# Blue-enriched LED light modulates biochemical and proteomic traits without affecting yield in indoor-grown cress microgreens

**DOI:** 10.3389/fpls.2026.1814329

**Published:** 2026-04-27

**Authors:** Andrea Ertani, Mariapia Esposito, Simonetta Caira, Jouhaina Riahi, Carla Colombani, Andrea Scaloni, Roberta Paradiso, Roberta Bulgari

**Affiliations:** 1 Department of Agricultural, Forest and Food Sciences (DISAFA), University of Turin, Grugliasco, Italy; 2 Institute for the Animal Production System in the Mediterranean Environment, National Research Council of Italy (CNR), Portici, Italy; 3 Department of Agricultural and Environmental Sciences – Production, Landscape, Agroenergy (DiSAA), University of Milan, Milan, Italy; 4 Department of Agricultural Sciences, University of Naples Federico II, Portici, Italy

**Keywords:** antioxidants, controlled-environment agriculture, *Lepidium sativum* L., proteomic profiling, secondary metabolism, soilless cultivation

## Abstract

Light spectral composition is a key driver of plant growth and quality in controlled-environment agriculture. This study evaluated the effects of two LED light spectra differing in blue, green, red, and far-red proportions on growth, nutritional traits, and proteomic profiles of indoor-grown cress (*Lepidium sativum* L.) microgreens. Plants were cultivated under two B:G:R:FR ratios (13:15:61:11 and 24:12:56:8) at identical photosynthetic photon flux density (255 μmol m^-2^ s^-1^). Growth parameters, including plantlet height, fresh and dry weight, dry matter percentage, and yield, did not differ significantly between treatments. Similarly, nitrate content, total chlorophylls, total carbon, total nitrogen, and the C/N ratio remained unchanged. In contrast, the blue-enriched and far-red-reduced spectrum significantly enhanced secondary metabolite accumulation, increasing anthocyanin concentration and phenolic index rising by 77% and 52%, respectively. Microgreen proteomics revealed a general plant reprogramming depending on LED light irradiation, mostly involving components associated with photosynthesis, protein physical control/biosynthesis/homeostasis/modification, multi-process regulation, RNA biosynthesis, vesicle trafficking, redox homeostasis or with unknown function, which showed over- or down-represented trends according to their nature. Notably, these molecular adjustments occurred without compromising growth or productivity. Overall, these results demonstrate that targeted manipulation of LED light quality can enhance the functional and nutritional quality of microgreens through proteomic reprogramming while maintaining yield, highlighting light spectrum modulation as a promising strategy for sustainable indoor and vertical farming systems. Repository data: PRIDE accession number PXD077094 (ProteomeXchange).

## 1 Introduction

Microgreens are increasingly recognized as a valuable horticultural crop, characterized by short production cycles and a high density of health-promoting compounds. These young edible plants, harvested at an early developmental stage (two to four true leaves), often contain higher concentrations of essential nutrients than their mature counterparts (Kyriacou et al., 2016; Falcinelli et al., 2025; Seth et al., 2025). Microgreens are rich in vitamins, minerals, and a wide range of bioactive compounds with antioxidant and health-promoting properties (Xiao et al., 2012; Mir et al., 2017; Turner et al., 2020). Their combination of concentrated nutrients, vibrant colours, and distinctive flavours has driven their increasing popularity in both functional and gourmet diets. Among the different species cultivated as microgreens, cress (*Lepidium sativum* L.) is particularly valued for its rapid growth, distinctive flavour profile, and high content of essential nutrients and secondary metabolites, including antioxidants such as anthocyanins and phenolic compounds (Keutgen et al., 2021).

The expansion of urban populations and the progressive reduction of arable land have stimulated interest in controlled-environment agriculture as a sustainable solution to meet the growing demand for fresh and nutritious foods. Indoor cultivation systems are particularly suitable for microgreen production, as they allow precise regulation of environmental factors such as temperature, humidity, and light. In this context, soilless cultivation systems represent an effective approach for indoor farming, supporting sustainable practices while enabling growers to optimize crop yield and quality (Dubey et al., 2024).

Light is among the most influential environmental factors shaping plant growth, metabolism, and nutritional quality. In indoor farming systems, light-emitting diodes (LEDs) are increasingly adopted owing to their energy efficiency, spectral flexibility, and precise controllability. Different light wavelengths, including blue (B), red (R), green (G), and far-red (FR), exert specific and well-documented effects on plant physiology (Massa et al., 2008; Samuolienė et al., 2013; Cocetta et al., 2017; Dou et al., 2017; Falcinelli et al., 2024; Farhangi et al., 2025; Heuvelink et al, 2025; Paradiso et al., 2025). Blue light is known to promote compact vegetative growth by enhancing stomatal conductance and limiting excessive stem elongation. It also stimulates the accumulation of secondary metabolites such as phenolic compounds, flavonoids, and anthocyanins, mainly through the activation of the phenylpropanoid-related pathway. Red light is highly efficient in driving photosynthesis and is generally associated with increased biomass accumulation. Far-red light, by altering the red/far-red ratio perceived by phytochromes, modulates plant morphology and shade-avoidance responses, including stem elongation and leaf expansion.

More recently, a crucial role of green light has been recognized in plant growth and canopy light distribution. Although traditionally considered less photosynthetically active, green light penetrates deeper into plant canopies than blue and red wavelengths, contributing to photosynthesis in lower leaf layers and influencing whole-plant carbon assimilation. In addition, green light has been shown to interact with blue-light signaling, modulating photomorphogenic responses and secondary metabolism (Smith et al., 2017; Wang et al., 2021; Paradiso et al., 2025).

By manipulating light spectral composition, growers can therefore fine-tune plant traits such as secondary metabolite accumulation, chlorophyll concentration, and nitrate levels, which are among the key indicators of nutritional value and sensory quality (Bian et al., 2015; Castañares, 2026). Accordingly, recent studies have demonstrated that targeted spectral strategies can enhance the functional quality of microgreens while maintaining, or even improving, productivity, highlighting light spectrum optimization as a powerful tool in controlled-environment agriculture (Zhang et al., 2020; Mir et al., 2024; Flores et al., 2024; Monterisi et al., 2025). Despite this growing interest, the integrated biochemical and proteomic responses of microgreens to specific light spectra remain incompletely understood. In particular, how changes in light quality can reshape metabolic priorities without compromising growth performance remains poorly defined. Although the effects of light spectral composition have been investigated in several microgreen species (Kyriacou et al., 2019; Zhang et al., 2020; Bantis et al., 2021; Liu et al., 2022; Pescarini et al., 2023; Mir et al., 2024; Borrelli et al., 2026; Cunha-Chiamolera et al., 2026), targeted studies focusing on cress (*Lepidium sativum* L.) remain limited. This underscores the need for species-specific investigations under controlled-environment conditions, where light quality and other growth parameters can be precisely regulated. The selected light spectra were chosen to represent two contrasting, yet agronomically realistic, lighting strategies commonly adopted in indoor and vertical farming systems and are further supported by evidence from previous studies (Sheikhi et al., 2024; Profico et al., 2025; Profico and Nicola 2025; Profico et al., 2026). Rather than exhaustively testing multiple spectral combinations, this work provides an in-depth characterization of plant responses under two application-relevant lighting conditions. Specifically, it aimed to evaluate the effects of two LED light spectra differing in their blue, green, red, and far-red proportions on the growth, nutritional traits, and proteomic profiles of indoor-grown cress microgreens cultivated in a controlled soilless system.

## 2 Materials and methods

### 2.1 Plant growth conditions, light treatments, and harvest

Cress (*Lepidium sativum* L.) was sown on March 18, 2024, at the Department of Agricultural, Forest and Food Sciences (DISAFA), University of Turin, Grugliasco, Italy. All materials were sterilized prior to use with a 5% sodium hypochlorite solution. The cultivation substrate consisted of hemp fiber mats placed in plastic trays (50 x 30 cm). For each tray, 35 g of seeds (Jelitto Staudensamen GmbH, Schwarmstedt, Germany) were evenly distributed on the substrate. A total of 6 trays were prepared with three biological replicates per treatment (one tray per shelf). After sowing, the substrate was irrigated with deionized water, and the trays were maintained in darkness at 22-25 °C until germination. Following emergence, the trays were transferred to an indoor cultivation system (approximately ~3 m³) and exposed to different LED light treatments at a total photosynthetic photon flux density (PPFD) of 255 µmol m² s^-1^.

Plants were grown under a 14 h light/10 h dark photoperiod, selected to reflect commonly adopted indoor cultivation practices and supported by previous studies (Liu et al., 2022; Sheikhi et al., 2024; Profico et al., 2025; Profico and Nicola 2025; Profico et al., 2026), with air temperature maintained at 22-25 °C and relative humidity at 50-70%. Two blue:green:red:far-red (B:G:R:FR) spectral ratios were tested: 13:15:61:11 (Shelf 1) and 24:12:56:8 (Shelf 2). Three days before harvest, microgreens were supplied with a nutrient solution composed of (mmol L^-1^): 6 N (40/60 N-NO_3_^-^/N-NH_4_^+^), 2 P, 6 K, 2 Mg, and 2.5 Ca. Prior to this, the hemp substrate had been kept moist with water only. Microgreens were harvested 10 days after sowing by cutting the shoots approximately 5 mm above the substrate and were immediately processed for subsequent analyses.

### 2.2 Plantlet growth

At harvest, plantlet height (H) was measured from the substrate surface (hemp substrate) to the tip of the tallest leaf, and fresh weight (FW) was determined for each tray. Dry weight (DW) was obtained after oven-drying the samples at 65 °C, for 24 h, until constant weight. Dry matter content (DM) was calculated as the ratio between DW and FW and expressed as percentage (DM%).

### 2.3 Biochemical and nutritional traits

#### 2.3.1 Total chlorophylls and carotenoids

Total chlorophylls (*a*+*b*) and carotenoids were determined according to Lichtenthaler (1987). Briefly, 50 mg of microgreens were extracted with 100% (v/v) methanol and incubated at 4 °C, for 24 h, in the dark. After extraction, absorbance was measured at 665.2 nm (chlorophyll *a*), 652.4 nm (chlorophyll *b*), and 470 nm (carotenoids), using a UV–Vis spectrophotometer (Cary 60 UV-Vis, Agilent Technologies, Santa Clara, CA, USA). Pigment concentrations were calculated using Lichtenthaler’s equations and expressed on a FW basis:


Chl a = 16.72 × A665.2 – 9.16 × A652.4

Chl b = 34.09 × A652.4 – 15.28 × A665.2

Total carotenoids = (1000 × A470 – 1.63 × Chl a – 104.96 × Chl b)/221

#### 2.3.2 Phenolic index and anthocyanins concentration

The phenolic index was determined on 50 mg of cress microgreens. Samples were extracted with 3 mL of methanol acidified with 1% (v/v) hydrochloric acid and incubated at 4 °C, for 24 h, in the dark. Absorbance was measured at 320 nm using a spectrophotometer and results expressed as absorbance units at 320 nm per gram of FW (ABS320 nm g^−1^ FW), according to Ke and Saltveit (1989). Anthocyanins concentration was determined spectrophotometrically, using the same methanolic extract. Absorbance was measured at 535 nm, and results were expressed as cyanidin-3-glucoside equivalents, following the method described by Klein and Hagen (1961).

#### 2.3.3 Nitrate, total carbon (C) and total nitrogen (N) content, and C/N ratio

Nitrate content was determined according to the salicylic acid method described by Cataldo et al. (1975). Briefly, 1 g of microgreens was homogenized in 3 mL of distilled water and centrifuged at 4000 x *g* for 15 min, at room temperature, using a benchtop centrifuge (ROTANTA 460R, Tuttlingen, Germany). An aliquot (20 μL) of the supernatant was mixed with 80 μL of 5% salicylic acid in sulphuric acid. After incubation at room temperature for 15 min, 3 mL of 1.5 N NaOH were added. Once cooled at room temperature, absorbance was measured at 410 nm using a spectrophotometer. Nitrate content was calculated based on a potassium nitrate (KNO_3_) standard calibration curve (0, 1, 2.5, 5, 7.5, 10 mM).

Total carbon (C) and total nitrogen (N) were determined in oven-dried microgreens (65 °C) by dry combustion method using an elemental analyser (ThermoQuest NA1500, Carlo Erba, Milan, Italy). Dried samples were finely ground using an oscillating mill (model MM 400, Retsch, GmbH, Retsch-Allee, Haan). Aliquots of 3–5 mg were weighted into tin (Sn) capsules and analyzed. Carbon and nitrogen concentrations were quantified based on the peak areas using an atropine calibration standard. The C/N ratio was subsequently calculated.

### 2.4 Proteomics analysis

#### 2.4.1 Protein extraction and sample preparation

From each of the three trays per treatment, 3 biological replicates of leaf tissues were collected, yielding a total of samples (5 for Shelf 1 and 5 for Shelf 2). For each replicate, approximately 100 mg of fresh leaf tissue was flash-frozen in liquid nitrogen and ground into a fine powder. Protein extraction was performed using a trichloroacetic acid (TCA)/acetone precipitation protocol. Briefly, powdered samples were homogenized in 4 mL of extraction buffer containing 10% (w/v) trichloroacetic acid (TCA) and 0.07% (v/v) dithiothreitol (DTT) in cold acetone. Homogenates were mixed thoroughly, stored overnight at –20 °C, centrifuged at 10,000 × g for 1 h at 4 °C, and supernatants were discarded. Pellets were washed twice with 4 mL of cold rinse solution (0.07% v/v DTT in acetone). After each wash, samples were incubated at –20 °C for 1 h, centrifuged at 10,000 × g for 30 min at 4 °C, and supernatants were discarded. Final pellets were air-dried under a laminar flow hood for 10 min. Dried pellets were resuspended in 1 mL of solubilization buffer containing 8 M urea and 50 mM triethylammonium bicarbonate, pH 8.5, supplemented with protease inhibitors (1 mM phenylmethylsulfonyl fluoride, PMSF, and protease inhibitor cocktail; Sigma-Aldrich). Samples were vortexed and incubated overnight at 30 °C under gentle agitation. Following incubation, samples were centrifuged at 12,000 × g for 5 min at 4 °C, and supernatants were collected.

Protein reduction was performed by adding 100 µL of 100 mM DTT to each sample and incubating at 60 °C for 1 h. Alkylation was carried out by adding 300 µL of 120 mM iodoacetamide and incubating the sample for 1 h, at room temperature, in the dark. Proteins were subsequently precipitated by adding cold acetone at a 5:1 (v/v) ratio and incubating overnight at –20 °C. Samples were centrifuged at 12,000 × g for 5 min at 4 °C, and supernatants were discarded. Protein concentration was determined using the Bio-Rad Protein Assay (Bio-Rad, Hercules, CA, USA) according to the manufacturer’s instructions. For enzymatic digestion, 100 µg of protein from each sample was digested with sequencing-grade trypsin (2 µL, 1 mg/mL) and incubated overnight at 37 °C. Digestion was stopped by cooling the samples on ice for 10 min. The resulting peptide mixtures were dried, resuspended in 50 µL of 50% (v/v) acetonitrile containing 0.1% (v/v) trifluoroacetic acid, and desalted using ZipTip™ C18 pipette tips prior to LC–MS/MS analysis.

#### 2.4.2 NanoLC-ESI-Q-Orbitrap-MS/MS analysis

Label-free proteomic analysis was performed with 3 technical replicates on the selected samples using a Thermo Scientific Orbitrap Exploris 240 mass spectrometer coupled online to an UltiMate 3000 RSLC nano-HPLC system (Dionex, Sunnyvale, CA, USA) via a Nanoflex ion source (Thermo Fisher Scientific). Peptides were loaded onto an Acclaim PepMapTM RSLC C18 column (150 mm × 75 μm ID, 2 μm particles, 100 Å pore size) (Thermo-Fisher Scientific) and separated at flow rate of 300 nL/min using a binary solvent system consisting of solvent A (water with 0.1% v/v formic acid) and solvent B (acetonitrile/water/formic acid, 80/19.92/0.08 v/v/v) in 1 min. The column was held at 6% solvent B for 8 min, increased to 31% over 90 min, increased to 50% over 5 min, increased to 95% over 5 min, remained at 95% for 8 min, and finally returned to 6% for equilibrating step. The mass spectrometer was operated in data-dependent acquisition (DDA) mode. Full MS scans were acquired in the Orbitrap over an *m/z* range 375–1500 at a resolution of 70,000. MS/MS spectra were collected for the ten most intense precursor ions with a normalized collision energy of 32%. MS/MS spectra were recorded at a resolution of 17,500 over a *m/z* range of 110–2000, with an automatic gain (ACG) control target of 100,000, a maximum injection time of 120 ms. Dynamic exclusion was enabled with a duration of 45 s.

#### 2.4.3 Bioinformatic analysis

Raw mass spectrometry data were processed using Proteome Discoverer (PD) vs. 3.1 (Thermo Scientific), with Mascot v2.6.1 (Matrix Science, UK) as the search engine. Peptide identification was performed against the UniProtKB *Brassica napus* protein database, due to the absence of the annotated *L. sativum* L. genome. Mass tolerance was set to ±10 ppm for precursor ions and ±0.05 Da for fragment ions. Carbamidomethylation of cysteine was specified as a fixed modification, while oxidation of methionine, deamidation of asparagine and glutamine, and pyroglutamate formation from glutamine at peptide N-terminus were set as variable modifications. Trypsin was selected as the proteolytic enzyme, allowing up to two missed cleavages. Peptide-spectrum matches were filtered using a Mascot ion score threshold of 30. Protein identification required the detection of at least one unique peptide, and only high-confidence identifications were retained with a false discovery rate (FDR) <1%, estimated using a target–decoy strategy. Proteomic analysis was performed using stringent filtering criteria in Proteome Discoverer (Master = “Master,” Protein FDR Confidence = High, and Unique peptides ≠ 0). Label-free quantification was performed based on precursor ion intensities. Statistical significance was assessed using a p-value < 0.05. Proteins with an abundance ratio <0.66 or >1.50 were considered differentially represented proteins (DRPs) and selected for downstream analyses. Due to the poor annotation of the *B. napus* genome and the large number of components firstly identified as uncharacterized proteins, the corresponding accession numbers were further searched in the UniProtKB database and associated with specific molecular species based on the direct dataset output and/or the retrieved entries having a sequence identity >90% from highly related organisms.

Heatmap visualization was generated within PD using Euclidean distance and average linkage clustering to highlight quantitative patterns among DRPs across samples. Principal component analysis (PCA) was performed using DRPs as variables, with sample groups projected in a two-dimensional space to assess clustering and variance among treatments.

### 2.5 Statistical analysis

The experiment was arranged according to a completely randomized design. Growth parameters (H, FW, yield, DW, DM%) as well as biochemical and nutritional traits were analyzed using GraphPad Prism 10 software (GraphPad Software, La Jolla, CA, USA; www.graphpad.com). Data are presented as mean value ± standard error (SE). Differences between the two light treatments were evaluated using Student’s t-test, and statistical significance was accepted at p < 0.05. Details regarding replication and statistical outcomes are reported in the corresponding tables and figure legends.

## 3 Results

### 3.1 Plantlet growth

Two plant groups were grown as reported in the experimental section; they experienced two LED light treatments that differed for blue:green:red:far-red (B:G:R:FR) spectral ratios, being 13:15:61:11 (Shelf 1) and 24:12:56:8 (Shelf 2). Growth and yield parameters of cress microgreens are reported in **Table 1**. No statistically significant differences were observed between treatments for any of the measured parameters. Microgreens height was comparable between Shelf 1 (4.12 ± 0.13 cm) and Shelf 2 (4.24 ± 0.09 cm). Similarly, FW per tray did not differ significantly between Shelf 1 (94.88 ± 1.02 g) and Shelf 2 (97.77 ± 2.46 g). Consequently, yield values were also similar between the two treatments, averaging approximately 1985 g m^-2^. Dry weight values showed no significant differences between Shelf 1 (5.14 ± 0.11 g) and Shelf 2 (6.18 ± 0.41 g). Likewise, DM % was comparable between treatments, with values of 5.42 ± 0.001% for Shelf 1 and 6.34 ± 0.005% for Shelf 2. Overall, the two light regimes resulted in similar growth and yield performance of cress microgreens.

**Table 1:** Microgreens height (H), fresh weight (FW) per 50 x 30 cm tray, yield, dry weight (DW), and dry matter content (DM) of cress microgreens cultivated under two LED light treatments with B:G:R:FR ratios of 13:15:61:11 (Shelf 1) and 24:12:56:8 (Shelf 2).

Light treatment	H(cm)	FW(g per tray)	Yield(g/m^2^)	DW(g)	DM(%)
Shelf 1	4.12 ± 0.13	94.88 ± 1.02	1990.00 ± 24.06	5.14 ± 0.11	5.42 ± 0.001
Shelf 2	4.24 ± 0.09	97.77 ± 2.46	1980.00 ± 21.31	6.18 ± 0.41	6.34 ± 0.005

Data are presented as means ± SE (n = 3), except for H (n = 18). Different letters within the same column indicate statistically significant differences (*p* < 0.05), where present.

### 3.2 Biochemical and nutritional traits

To assess the effect of the two distinct light treatments (Shelf 1 and Shelf 2) on the biochemical composition and nutritional traits of cress microgreens, several parameters were evaluated, including total chlorophylls, carotenoids, phenolic index, anthocyanin concentration, nitrate content, total carbon, total nitrogen, and the C/N ratio.

Cress microgreens grown under Shelf 1 and Shelf 2 exhibited comparable total chlorophyll concentrations, with an average value of 3.80 µg/mg FW (**Table 2**). Carotenoid concentrations were very low and often below the detection limit in both the treatments (data not shown).

**Table 2:** Total chlorophyll and anthocyanin concentrations, and phenolic index of cress microgreens grown under two LED light treatments with B:G:R:FR ratios of 13:15:61:11 (Shelf 1) and 24:12:56:8 (Shelf 2).

Light treatment	Total chlorophyll (µg/mg FW)	Anthocyanins (mg/100 g FW)	Phenolic index (ABS320 nm/g FW)
Shelf 1	3.97 ± 0.35	4.20 ± 0.26 b	13.78 ± 1.00 b
Shelf 2	3.64 ± 0.26	7.46 ± 0.47 a	20.99 ± 0.80 a

Data are presented as means ± SE (n = 9). Means followed by different letters within the same column indicate statistically significant differences (p < 0.05).

Anthocyanins concentration was significantly greater in Shelf 2 (7.46 mg/100 g FW) than in Shelf 1 (4.20 mg/100 g FW) (**Table 2**). Shelf 2 showed also a markedly higher phenolic index (20.99 ABS320 nm/g FW) compared to Shelf 1 (13.78 ABS320 nm/g FW), with a statistically significant difference (**Table 2**).

### 3.3 Nitrate content, total C (%), total N (%), and C/N ratio

Light treatments did not significantly influence the total carbon (%) of cress microgreens, with an average value of 44.68 across treatments (**Table 3**). Similarly, nitrate content and total nitrogen (%) were comparable between Shelf 1 and Shelf 2, with an average nitrate level of 10.36 mg/kg FW (**Table 3**). Overall, no statistically significant differences were observed between treatments for any of the measured parameters in cress microgreens, including C%, nitrate content, N%, and C/N ratio.

**Table 3:** Total carbon (C %), nitrate content, total nitrogen (N %), and C/N ratio of cress microgreens grown under two LED light treatments with B:G:R:FR ratios of 13:15:61:11 (Shelf 1) and 24:12:56:8 (Shelf 2).

Light treatment	C (%)	Nitrate (mg/kg FW)	N (%)	C/N
Shelf 1	45.80 ± 2.06	10.17 ± 2.14	5.93 ± 0.22	7.64 ± 0.30
Shelf 2	43.56 ± 0.03	10.56 ± 1.80	5.67 ± 0.11	7.74 ± 0.12

Data are presented as means ± SE. For nitrate, n = 9; for C%, N%, and C/N, n = 3. Means followed by different letters within the same column indicate statistically significant differences (p < 0.05), where present.

### 3.4 Cress proteome response to differential light exposure

**Figure 1:**
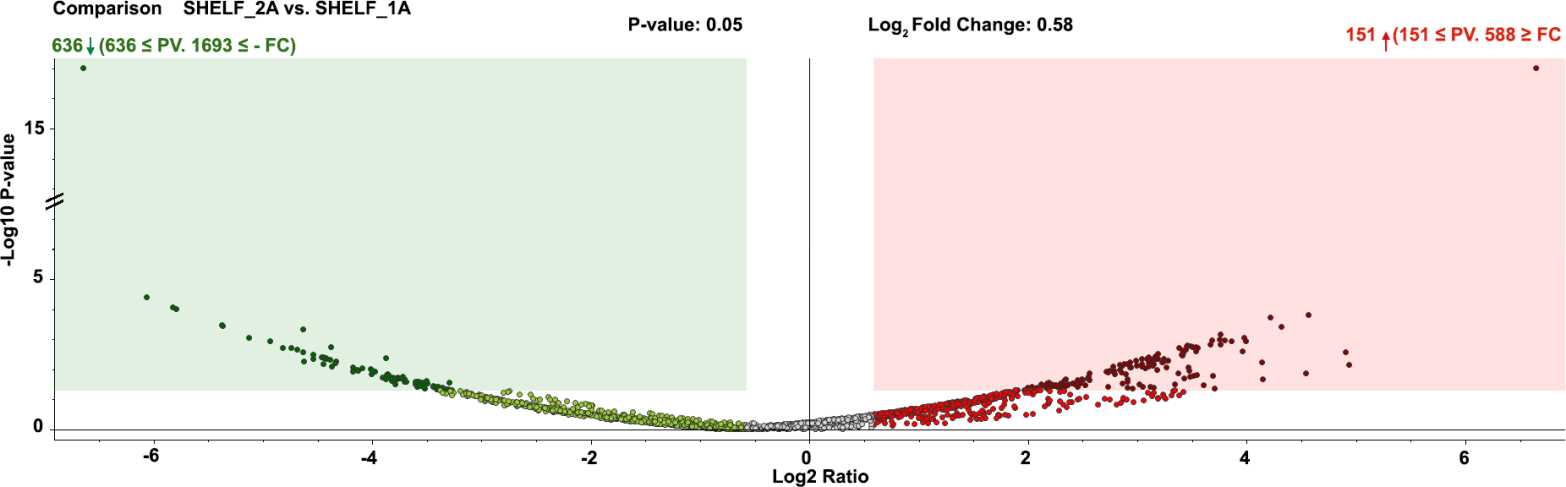
Volcano plot of differential protein representation between Shelf 2 and Shelf 1. The X-axis represents log_2_ fold change (positive = over-representation; negative = down-representation), and the Y-axis shows –log_10_ FDR-adjusted p-value. Red dots denote significantly over-represented proteins, green dots indicate significantly down-represented proteins, and grey dots represent non-significant changes.

Proteomic analysis identified 3,161 proteins in cress microgreen samples. Protein identification results between the two light treatments are reported in **Supplementary Table 1**. Based on selected abundance ratios reported in the experimental section, proteomic ANOVA analysis of cress microgreens exposed to two distinct LED spectra (Shelf 1 and Shelf 2) identified a total of 787 differentially represented proteins (DRPs) between treatments (adjusted *p* < 0.05). Among that, 151 were over-represented and 636 down-represented under Shelf 2 compared to Shelf 1. The overall distribution of DRPs is illustrated in the volcano plot shown in **Figure 1**, where log_2_ fold change is plotted against the –log_10_ FDR-adjusted p-value. Proteins showing significant over-representation or down-representation are clearly distinguished from those not significantly affected by the light treatments.

**Figure 2:**
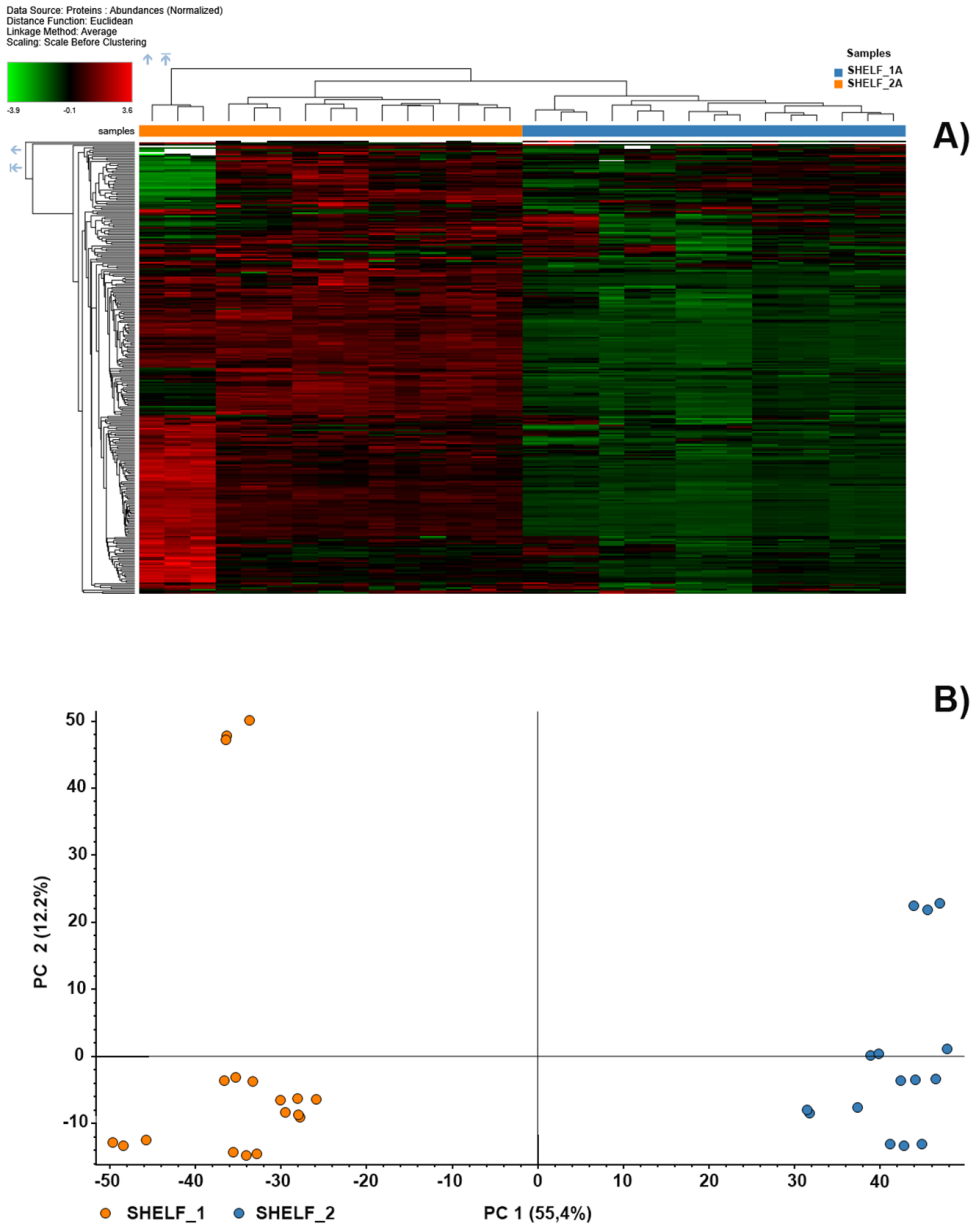
Comparative proteomic analysis of cress plants grown under two artificial B:G:R:FR light spectra: Shelf 1 = 13:15:61:11; Shelf 2 = 24:12:56:8. **(A)** Heatmap of normalized protein abundances across samples from both light treatments. Hierarchical clustering was performed using Euclidean distance and complete linkage after data scaling. The color gradient ranges from green (low abundance, –4.2) to red (high abundance, 5.5), illustrating representation variation. **(B)** Principal Component Analysis (PCA) of proteomic profiles. PC1 and PC2 explain 12.2% and 55.4% of total variance, respectively. Samples cluster according to light treatment, indicating distinct proteomic signatures.

Hierarchical clustering of normalized protein abundances revealed differences in protein abundance patterns between samples grown under the two light treatments (**Figure 2A**). The heatmap displays variation in protein abundance across samples, with representation values spanning the observed normalized range. Principal Component Analysis (PCA) showed a separation of samples according to light treatment along the first two principal components (**Figure 2B**), which together accounted for 67.6% of the total variance (PC1: 12.2%; PC2: 55.4%). Shelf 1 and Shelf 2 occupied distinct regions of the PCA space, although variability among biological 3 replicates was observed within each treatment.

A detailed examination of the variably represented proteome showed a subset of components strongly induced under the Shelf 2 condition, with abundance ratios ranging from 1.5 up to 100 (FDR < 0.05; **Supplementary Table 1**). Among the most markedly over-represented proteins, worth mentioning are cytochrome b5 family protein (A0A078FCT5; ratio = 100), large subunit ribosomal protein L6e (A0A078JKN0; ratio = 23.622), late embryogenesis abundant protein group 3 (A0A078JTB5; ratio = 15.563), and ribulose bisphosphate carboxylase large subunit (A0A078I3R9; ratio = 13.565), along with additional not assigned components, such as unknown methyltransferase (A0A816R5K5; ratio = 30.65), uncharacterized protein HID58_063886 (A0ABQ7Z8E8; ratio = 30.042), and hypothetical protein LOC106412569 (A0A816J6H8; ratio = 15.791).

**Figure 3:**
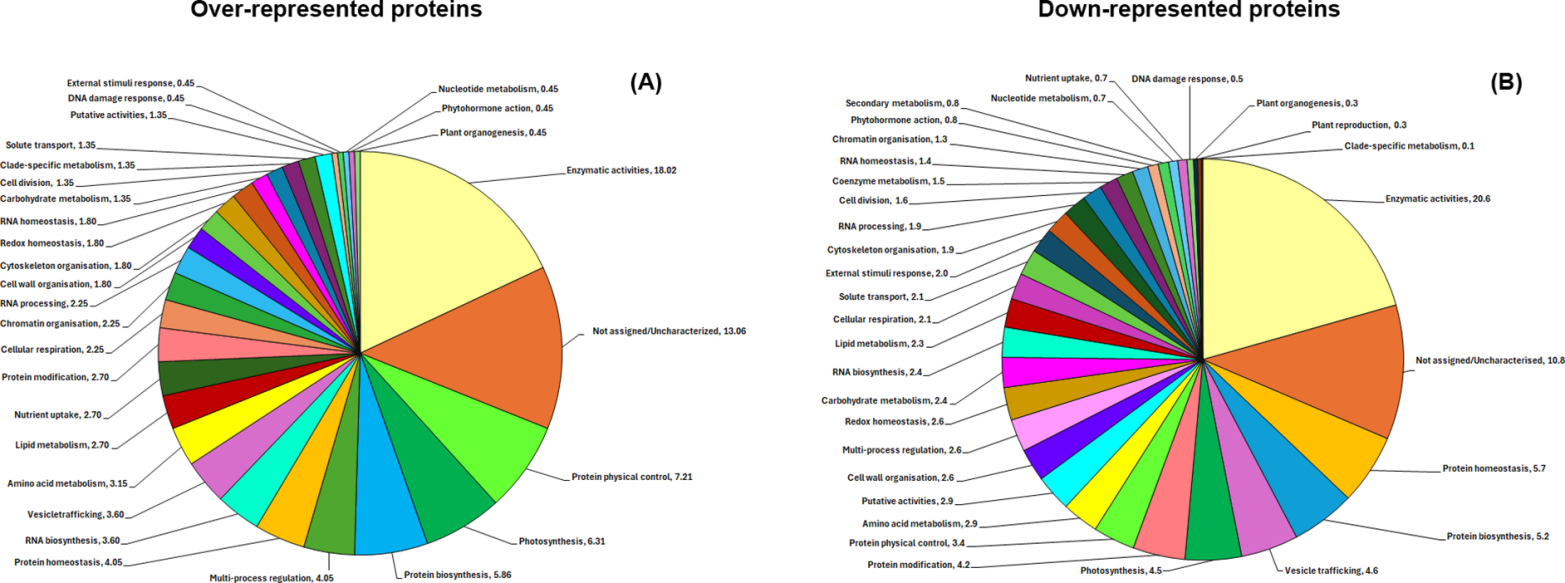
Functional distribution of over-represented **(A)** and down-represented **(B)** proteins in the Shelf 2 condition, as determined in cress microgreens exposed to two artificial B:G:R:FR light spectra. Identified protein species were assigned to specific functional classes using the Mercator software.

Over-represented proteins were indexed by a functional assignment obtained by Mercator software analysis (**Supplementary Table 2**). They were associated with one/multiple function(s), except 22 that were not linked to a component name and/or a known functional role. According to their identity, these proteins were related to: i) *protein physical control* (9.3%); ii) *photosynthesis* (8.8%); iii) *not assigned* (7.1%); iv) *protein biosynthesis* (7.1%); v) *multi-process regulation* (4.9%); vi) *protein homeostasis* (4.9%); vii) *uncharacterized context* (4.9%); viii) *RNA biosynthesis* (4.4%); ix) *vesicle trafficking* (4.4%); x) *amino acid metabolism* (3.8%), and other functions (**Figure 3A**). Within these functional families, bioinformatic analysis indicated enrichment of specific protein form control related, light related, stress-responsive signaling or metabolic activity subgroups (**Supplementary Table 2**), including: i) *protein chaperone activities*; ii) *photophosphorylation*; iii) *Calvin cycle*; iv) *organellar translation machinery and ribosome biogenesis*; v) *14-3–3 regulatory system*; vi) *calcium homeostasis*; vii) *glutamate and aspartate group amino acid biosynthesis*; viii); *DNA-binding transcriptional regulation;* ix) *phenolics biosynthesis*. Detailed information on over-represented components associated with above-reported subgroups is reported in **Supplementary Material**. Some of these proteins and related functional families/subfamilies have already been reported as variably represented in proteomic studies on blue light-treated leaves from *Scrophularia kakudensis* (Manivannan et al., 2021), *Arabidopsis thaliana* (Yavari et al., 2022), *Phyllostachys edulis* (Zhang et al., 2024), soybean hypocotyls (Wang et al., 2023, 2025), and *P. edulis* seedlings (Li et al., 2023).

Among the most markedly down-represented proteins worth mentioning are those associated with photosynthetic and chloroplast-related functions, including ribulose bisphosphate carboxylase large chain (rbcL) (A0A482K1T8; ratio = 0.01), photosystem I P700 chlorophyll a apoprotein A1 (A0A1B1Y0E3; ratio = 0.01), and photosystem II D2 protein (A0A816JB71; ratio = 0.01), suggesting an impairment of the photosynthetic machinery under the Shelf 2 condition (**Supplementary Table 3**).

Down-represented proteins were also subjected to Mercator-dependent functional assignment (**Supplementary Table 3**). They were associated with one/multiple function(s), except 97 species that were not linked to a component name and/or a known function. In general, decreased proteins were associated with: i) *uncharacterized context* (7.5%); ii) *protein homeostasis* (7.1%); iii) *protein biosynthesis* (6.5%); iv) *photosynthesis* (6.0%); v) *vesicle trafficking* (5.7%); vi) *protein modification* (5.2%); vii) *not assigned* (5.1%); viii) *protein physical control* (4.5%); ix) *redox homeostasis* (4.4%); x) *amino acid metabolism* (3.9%), and other functions (**Figure 3B**). Within these functional families, bioinformatic analysis showed enrichment of specific light related, protein form control related, stress-responsive, or metabolic activity subgroups, (**Supplementary Table 2**) including: i) *photophosphorylation;* ii) *Calvin cycle;* iii) *light response;* iv) *proteolysis;* v) *ubiquitin-proteasome system;* vi) *protein phosphorylation/dephosphorylation;* vii) *organellar translation machinery and ribosome biogenesis;* viii) *protein chaperone activities;* ix) *redox stress response;* x) *glutamate and aspartate group amino acid biosynthesis;* (xi) *phenolics biosynthesis*.

Collectively, above-reported results demonstrate that exposure to the Shelf 2 light spectrum triggered a widespread reprogramming of the cress leaf proteome that mostly involved components associated with photosynthesis, protein physical control-biosynthesis-homeostasis-modification, multi-process regulation, RNA biosynthesis, vesicle trafficking, redox homeostasis or with unknown function, which showed over- (less) or down-represented (more) trends according to their identity, but did not correspond to significant changes in plant growth parameters, and content of nitrate, total chlorophylls, total carbon, total nitrogen, and the resulting C/N ratio.

## 4 Discussion

### 4.1 Growth performance, antioxidants, and physiological stability under contrasting light spectra

This study demonstrates that targeted manipulation of LED light spectral composition can induce extensive biochemical reprogramming in cress microgreens without compromising plantlet growth or yield. A blue-enriched and far-red–reduced spectrum promoted the accumulation of antioxidant-related metabolites while preserving key physiological traits, highlighting a decoupling between primary growth processes and secondary metabolism. The absence of statistically significant differences between the two light treatments regarding growth parameters indicates that both lighting regimes were equally effective in supporting the growth and biomass production of cress microgreens. Although numerical differences were observed for some parameters, such as fresh weight, dry weight, and dry matter percentage, these variations did not reach statistical significance and therefore do not support definitive conclusions. In particular, differences in dry matter percentage should not be directly interpreted as differences in dry matter accumulation. Since dry matter percentage represents the complement of tissue water content, variations in DM% may also reflect differences in tissue hydration status rather than true changes in biomass synthesis. This distinction is especially relevant in microgreens, where water content can vary substantially depending on environmental conditions, without necessarily affecting overall productivity. Taken together, these results suggest that the two lighting conditions provide comparable physiological support for cress microgreens cultivation, with no measurable advantage of one shelf over the other in terms of growth or yield under the experimental conditions tested.

Total chlorophylls concentration was comparable between Shelf 1 and Shelf 2, while carotenoids were generally below the detection limit in both treatments. This indicates that photosynthetic pigment stability was maintained across contrasting light spectra. The significantly higher anthocyanin concentration and phenolic index observed in microgreens grown under Shelf 2 indicate that the corresponding light spectrum was more effective in stimulating the secondary metabolism. Light quality is known to play a key role in regulating the biosynthesis of phenolic compounds, including anthocyanins, through the activation of specific photoreceptors and downstream metabolic pathways. The increase in anthocyanin content (+78%) and phenolic index (+52%) under the Shelf 2 condition suggests an enhanced allocation of metabolic resources toward the production of antioxidant compounds. These responses are consistent with previous findings reporting the sensitivity of secondary metabolite accumulation to variations in light spectral composition, particularly in leafy vegetables and microgreens (Alrifai et al., 2021; Mahesh et al., 2025).

### 4.2 Nitrate content and elemental composition

The lack of significant differences in nitrate content between light treatments indicates that light spectral composition did not affect nitrate accumulation in cress microgreen tissues within the conditions applied in this study. Importantly, nitrate values measured in both treatments were several orders of magnitude lower than the maximum levels established by the European Commission for leafy vegetables (EU Regulation n° 1258/2011, confirmed by EU Regulation 917/2023), confirming a favourable nutritional and safety profile. It should be noted that nitrate accumulation is generally not a concern in microgreens, as they typically contain lower levels than adult plants (Bulgari et al., 2017). The regulation cited above, although referring to certain leafy vegetables, is commonly used in microgreen studies to assess food safety. Similarly, the comparable carbon and nitrogen contents, as well as the nearly identical C/N ratios, suggest that the balance between carbon assimilation and nitrogen uptake was not altered by the light environment. This finding supports the notion that both lighting regimes maintained a consistent physiological status in terms of primary metabolism.

### 4.3 Proteomic modulation and reprogramming under blue enriched light

The asymmetry in the number of proteins identified under the two lighting conditions indicated that light spectral composition exerts a strong influence on the cress microgreen proteome. Shelf 1 was associated with a broader set of uniquely detected proteins, suggesting the occurrence of a wider range of metabolic and signaling processes. Conversely, the more limited number of proteins uniquely detected under Shelf 2 pointed toward a more specific proteomic adjustment, potentially reflecting photomorphogenic regulation rather than broad metabolic activation. These trends are consistent with the established role of light quality, and in particular the relative proportions of red, blue, and far-red wavelengths, in shaping plant proteomic landscapes through photoreceptor-mediated signaling (Trivellini et al., 2023). Multivariate analyses further highlighted these spectral effects. Heatmap clustering and PCA revealed a clear separation between samples from the two light regimes. Shelf 1 samples showed greater dispersion, indicating higher proteomic variability, whereas Shelf 2 samples clustered more tightly, suggesting a more uniform response to the blue enriched spectrum. A few deviating replicates were observed, reflecting the inherent biological variability typical of plant systems and underscoring the value of multivariate approaches when interpreting complex proteomic datasets.

Beyond these global patterns, the large number of DRPs between Shelf 1 and Shelf 2 showed that light quality induced extensive proteomic reprogramming. The predominance of down-represented proteins under Shelf 2 indicates broad suppression of protein abundance in response to the blue enriched, far red–depleted spectrum. A similar quantitative trend was observed in blue light–treated leaves from *P. edulis* (Zhang et al., 2024). In contrast, only a smaller subset of proteins was over-represented, many of which are associated with *protein physical control*, *photosynthesis*, *protein biosynthesis*, *multi-process regulation*, *protein homeostasis*, *uncharacterized context*, *RNA biosynthesis*, *vesicle trafficking*, *amino acid metabolism*, *redox stress response* and *phenolics biosynthesis* pathways (**Supplementary Table 2**). This selective over-representation is consistent with the known influence of blue light on stress related signaling, photomorphogenesis, and redox regulation, often accompanied by reduced investment in growth related and photosynthetic functions (Trivellini et al., 2023). Similar patterns have been reported in Brassica and related species, where blue enriched light enhances stress tolerance but can reduce biomass accumulation or photosynthetic efficiency (Zhang et al., 2021; Liu et al., 2020; Xu et al., 2021; Wang et al., 2021; Li et al., 2019). Overall, these results indicate that different light spectra trigger distinct regulatory mechanisms at the proteomic level. Furthermore, Shelf 2 condition induced a targeted stress adaptation profile characterised by a widespread down-representation of specific components associated with *uncharacterized context*, *protein homeostasis*, *protein biosynthesis*, *photosynthesis*, *vesicle trafficking*, *protein modification*, *protein physical control*, *redox homeostasis* and *amino acid metabolism* pathways (**Supplementary Table 3**). Collectively, the combined evidence from unique molecular counts, clustering behaviour, and DRP profiles supports a model in which blue enriched lighting promoted a coordinated shift in the cress proteome toward defence, redox buffering, and photomorphogenic adjustments that was dependent on quantitative changes of a large number of components in percentage belonging to the same (restricted) functional classes, more than being associated with altered levels of few proteins related to very numerous biological roles.

### 4.4 Pathway level metabolic and functional reprogramming under blue enriched light

In Shelf 2 condition, the enrichment of selected pathways related to over-represented proteins involved in amino acid biosynthesis, nitrogen assimilation/metabolism, carbohydrate metabolism, secondary metabolite biosynthesis and redox regulation indicated a coordinated but protein specific metabolic response to the blue enriched spectrum. Partial activation of the pentose phosphate and glutathione-ascorbate metabolism pathways suggested an increased requirement for reducing power and antioxidant capacity, two essential components of cellular redox homeostasis under stress (**Supplementary Table 2**). The putative involvement of phenylpropanoid biosynthesis and cofactor related pathways further reflected the modulation of specific secondary metabolic processes commonly associated with oxidative stress mitigation and cellular protection. In fact, the observed over-representation of dihydroflavonol reductase 1, a key enzyme in the flavonoid biosynthetic pathway, specifically for the production of anthocyanins and proanthocyanidins, which plays a crucial role in pigmentation and light stress response (Ju et al., 1997; Gollop et al., 2002), and the parallel down-representation of cinnamoyl-CoA reductase 1 and a Fe2OG dioxygenase domain-containing protein similar to feruloyl-CoA 6’-hydroxylase respectively involved in the competitive formation of early cinnamaldehyde and coumarine intermediates prodrome to final biosynthesis of monolignols and lignin, was here tentatively associated with the plant response to selectively direct the latter pathways toward the specific accumulation of anthocyanins in Shelf 2 condition, as also experimentally observed (**Supplementary Tables 2** and **3**). Similar adjustments were documented in stress responsive proteomes of *Brassica napus*, where enhanced activity in carbohydrate, amino acid, secondary metabolite and redox related pathways helped sustaining growth while limiting oxidative damage during abiotic stress (Shu et al., 2022; Raza et al., 2021; Kanehisa et al., 2023).

In parallel, the extensive down-representation of proteins associated with photosynthesis and chloroplast functions under Shelf 2 condition indicated a strong suppression of energetically demanding primary metabolic pathways. The repression of key photosynthetic components involved in i) photosystems I and II (chlorophyll a-b binding protein isoforms Q2I0E4, A0A078ICK1 and A0A078GLT1; post-illumination chlorophyll fluorescence increase protein; thylakoid lumenal 17.9 kDa protein; beta-galactosidase protein; photosystem IP 700 chlorophyll a apoprotein A1; photosystem I subunit O; high chlorophyll fluorescence phenotype 173 protein; uncharacterized protein A0A816XSM9; chloroplastic zeaxanthin epoxidase; histone acetyltransferase A0A816TP41; CASP-like protein; photosystem II D2 protein; chloroplastic protein MET1; maintenance PSII under high light 1-like protein); ii) photophosphorylation/respiration (kinesin-like protein; chloroplastic ATP-synthase subunit a; dihydrodipicolinate reductase N-terminal domain-containing protein; chlororespiratory reduction 41 protein; RuBisCO large subunit-binding protein subunit alpha; chloroplastic NAD(P)H-quinone oxidoreductase subunit M; photosynthetic NDH subunit of subcomplex B5; plastocyanin isoforms A0A816ZC43 and A0A816QW44; cytochrome b6-f complex subunit 4; hypothetical protein A0A078FEV0; chloroplastic rhodanese-like domain-containing protein 4); iii) Calvin cycle enzyme complexes (phosphoribulokinase isoforms A0A078HT68 and A0ABQ7ZHQ8; ribose-5-phosphate isomerase; 4a-hydroxytetrahydrobiopterinde hydratase; trichome birefringence-like N-terminal domain-containing protein; ribosomal RNA-processing protein 42; uncharacterized protein A0ABQ8D036; RuBisCO large subunit-binding protein subunit alpha; sedoheptulose-1,7-bisphosphatase); iv) CAM/C4 photosynthetic enzymes (phosphoenolpyruvate carboxylase), suggested a general impairment of the photosynthetic apparatus and a marked decrease of the corresponding capacity (**Supplementary Table 3**). This phenomenon was already observed in blue light–treated leaves from *P. edulis* (Zhang et al., 2024). Regarding light stimulus response, worth mentioning is the observed down-representation of blue light photoreceptor-cryptochrome photoreceptor CRY and phytochrome B-like. The first protein has already been reported having quantitative levels negatively regulated by blue light in *A. thaliana* (Ahmad et al., 1998; Lin et al., 1998). When etiolated Arabidopsis seedlings were exposed to 20 to 30 mol m^-2^ sec^-1^ blue light, CRY2 protein levels declined more than 10-fold within 1 h (Ahmad et al., 1998; Lin et al., 1998). It was demonstrated that protein degradation mechanisms are responsible for the blue light–dependent regulation of the CRY2 protein abundance (Ahmad et al., 1998; Lin et al., 1998). Regarding phytochrome B-like, no evidence of a relation between red or blue light irradiation and protein concentration has been reported. Conversely, this protein was demonstrated being associated with radiation-induced autophosphorylation events (Christie et al., 2015).

On the other hand, enriched pathways among down-represented proteins—such as those related to cofactor and amino acid metabolism, and redox associated processes—highlighted specific and significant adjustments across both primary and secondary metabolic processes. Reduced representation of glutathione and ascorbate related proteins may reflect a strategic shift away from biosynthetically expensive antioxidant pathways, consistent with a reallocation of resources toward essential stress adaptation mechanisms. In this context, the blue irradiation set up used in Shelf 2 condition did seem not strong enough to massively activate apoplastic NADPH oxidases toward the generation of reactive oxygen species (ROS), as well as to promote induction of antioxidant defense enzymes including superoxide dismutases, ascorbate peroxidases, and catalases (**Supplementary Table 3**), as already observed under very high intensity of light exposition (Chibani et al., 2025). All above-reported proteins showed a general down-representation under the experimental conditions used in this study and corresponded to an unaltered plant physiological status ensuring optimal organism growth and productivity.

Additionally, massive down-representation of proteins involved in the mRNA surveillance pathway (29 in number) and polypeptide chain physical control/biosynthesis/homeostasis/modification processes (180 in number) pointed to a potential modulation of post-transcriptional regulation, translational and post-translational control under altered spectral environments (**Supplementary Table 3**), again reflecting a general downshift in energy-demanding functions. Collectively, these patterns indicated a shift from growth-oriented to survival-oriented metabolic states when plants are exposed to blue enriched, far red–depleted conditions. Similar spectral driven shifts have been reported in *B. napus* and related species, confirming that alterations in light quality can trigger profound and functionally coordinated proteomic remodeling (Grubb et al., 2025; Kanehisa et al., 2023).

### 4.5 Functional integration of light-quality responses in cress microgreens

In the present experiment, LED spectral modulation enhanced antioxidant-related traits without affecting growth or yield, supporting its potential to improve nutraceutical quality in controlled-environment agriculture (Azizi et al., 2025; Park et al., 2020). Interestingly, this metabolic shift occurred in the absence of a strong over-representation of multiple phenylpropanoid biosynthetic enzymes. In fact, only a parallel over-representation of dihydroflavonol reductase 1, and down-regulation of cinnamoyl-CoA reductase 1 and a Fe2OG dioxygenase domain-containing protein was observed. This finding suggests that the increased accumulation of phenolic compounds may be driven by the metabolic flux redistribution and post-translational regulation rather than changes in enzyme abundances alone. Such a mechanism is consistent with previous studies indicating that blue light enhances antioxidant capacity predominantly through photoreceptor-mediated signaling, redox modulation and stress-related responses, rather than through direct transcriptional or translational control of phenylpropanoid enzymes (Trivellini et al., 2023; Chibani et al., 2025).

Under Shelf 2 condition, a broad number of proteins associated with photosynthesis, ribosomal structure, and chloroplast functions were down-represented. This indicates a strategic reallocation of cellular resources from energy-intensive anabolic processes toward stress signaling and metabolic adjustment. Importantly, this extensive proteomic remodeling occurred without detectable changes in total chlorophyll content, nitrate content, or growth performance, suggesting that core physiological functions such as pigment maintenance and nitrogen assimilation were preserved. The slight increase in dry matter percentage may reflect alterations in tissue composition, potentially due to a higher relative contribution of structural or stress-responsive proteins rather than ribosomal components. Such a redistribution is consistent with a shift in carbon and nitrogen allocation toward protective and adaptive pathways, as reported under abiotic stress conditions (Cao et al., 2025; Roeber et al., 2021). The stability of nitrate content and C/N ratio further supports that nitrogen assimilation remained largely unaffected, even as amino acid metabolism and protein representation patterns were highly modified (Sathee et al., 2021). This condition was associated with the occurrence of a balance between over-represented and down-represented isoforms of glutamine synthase, augmented levels of aspartate aminotransferase, and repressed quantitative values of multifunctional fusion protein and Mn-dependent ADP-ribose/CDP-alcohol diphosphatase protein in Shelf 2 condition (**Supplementary Tables 2** and **3**), all already linked to nitrogen assimilation in plants (Kishorekumar et al., 2020), which probably contributed to determine an unaltered concentration of this element.

The maintenance of growth and yield despite major extensive proteomic remodeling highlights the remarkable physiological plasticity of microgreens in response to spectral cues. This apparent decoupling between primary growth processes from secondary metabolism underscores the potential of light-quality as a non-invasive strategy to enhance nutritional and functional traits without compromising productivity. From an applied perspective, these findings are particularly relevant for vertical farming and controlled-environment agriculture, where tailored light spectra may be exploited to simultaneously sustain yield and improve crop quality. Future studies should assess whether these proteomic adjustments persist during later developmental stages and will evaluate the extent to which these responses are conserved across different species. Moreover, integrating spectral optimization with nutrient management and other environmental controls may further refine cultivation strategies for sustainable indoor production. Expanding this approach to additional crops will help clarify whether the trade-offs observed between photosynthetic investment and antioxidant enhancement represent a general adaptive response or a species-specific trait (Azizi et al., 2025; Paradiso and Proietti, 2021).

## 5 Conclusions

This study demonstrates that targeted manipulation of LED light spectra, specifically increasing blue light and reducing far-red light, can substantially reshape the biochemical and proteomic profile of cress microgreens, without compromising plantlet growth and yield. Under blue-enriched conditions, anthocyanin concentration and phenolic index increased markedly, while chlorophylls and nitrate levels remained stable, indicating that nutritional enhancement can be achieved without loss of productivity.

Proteomic analysis revealed a coordinated reprogramming of cellular priorities, characterized by the over- or down-representation of specific components mostly associated with photosynthesis, protein physical control/biosynthesis/homeostasis/modification, multi-process regulation, RNA biosynthesis, vesicle trafficking, redox homeostasis or with unknown function, whose nature and representativity was dependent on the irradiation condition. The large number of DRPs between Shelf 1 and Shelf 2 showed that light quality induced extensive proteomic reprogramming. The predominance of down-represented proteins under Shelf 2 indicates broad suppression of protein abundance in response to the blue enriched, far red–depleted spectrum. In general, Shelf 2 condition promoted quantitative protein changes of a large number of components in percentage belonging to the same (restricted) functional classes, more than being associated with altered levels of few macromolecules related to very numerous biological roles. This metabolic flexibility likely underlies the apparent decoupling between protein abundance and metabolite accumulation, emphasizing the role of flux redistribution and post-translational regulation.

Overall, these findings highlight spectrum-tailored LED lighting as a promising and sustainable strategy to enhance the functional quality of microgreens in controlled-environment agriculture. Future studies will assess the persistence of these responses at later developmental stages, explore species-specific variability, and integrate spectral optimization with nutrient and environmental management. Combining biochemical analyses with proteomics, transcriptomics, metabolomics, and phosphoproteomic approaches will be essential to fully elucidate the regulatory networks underlying light-driven metabolic adaptation. Evaluating economic feasibility and consumer-relevant quality traits will further support the translation of spectral strategies into practical indoor farming applications.

## Data Availability

The mass spectrometry proteomics data have been deposited to the ProteomeXchange Consortium via the PRIDE partner repository with the dataset identifier PXD077094. The raw data supporting the conclusions of this article will be made available by the authors, without undue reservation.
