# Supplementary material for: Blue-enriched LED light modulates biochemical and proteomic traits without affecting yield in indoor-grown cress microgreens
Source: Front Plant Sci. 2026 Apr 27;17:1814329. doi: 10.3389/fpls.2026.1814329 (PMC13158100; doi:10.3389/fpls.2026.1814329)
Supplement: Supplementary file 1 [file Table1.docx]

**Blue-enriched LED light modulates biochemical and proteomic traits without affecting yield in indoor-grown cress microgreens**

**Andrea Ertani^1^, Mariapia Esposito^2^, Simonetta Caira^2^, Jouhaina Riahi^1^, Carla Colombani^3^, Andrea Scaloni^2^, Roberta Paradiso^4^, Roberta Bulgari^3^***

^1^ University of Turin, Department of Agricultural, Forest and Food Sciences (DISAFA), Largo Paolo Braccini 2, 10095 Grugliasco (Turin), Italy

^2^ Institute for the Animal Production System in the Mediterranean Environment, National Research Council of Italy (CNR), Piazzale Enrico Fermi 1, 80055 Portici (Naples), Italy

^3^ University of Milan, Department of Agricultural and Environmental Sciences – Production, Landscape, Agroenergy (DiSAA), Via Celoria 2, 20133 Milan, Italy

^4^ University of Naples Federico II, Department of Agricultural Sciences, Piazza Carlo di Borbone 1, 80055 Portici (Naples), Italy

*** Correspondence:** [roberta.bulgari@unimi.it](mailto:roberta.bulgari@unimi.it)

***SUPPLEMENTARY MATERIAL***

***Proteomic changes associated with exposure to different light sources***

When over-represented proteins under the Shelf 2 condition were indexed by a functional assignment obtained by Mercator software analysis, they were associated with one/multiple function(s), except 22 that were not linked to a component name and/or a known function. According to their identity and numerosity, these proteins were related in order to *protein physical control*, *photosynthesis*, *not assigned*, *protein biosynthesis*, *multi-process regulation*, *protein homeostasis*, *uncharacterized context*, *RNA biosynthesis*, *vesicle trafficking*, *amino acid metabolism* and other functional groups (Figure 3A). Within these families, bioinformatic analysis indicated specific enriched metabolic and stress-responsive activity subgroups (Supplementary Table S2), which included individual over-represented proteins as follow: i) *protein chaperone activities* (chaperone protein dnaJ 3-like; heat shock 70 kDa protein 15; small monomeric GTPase; TPR repeat-containing thioredoxin; STI1 domain-containing protein; co-chaperone protein p23-1; mitochondrial chaperonin CPN60; chaperonin HSP60; and chloroplast heat shock protein 70); ii) *photophosphorylation* (chaperonin HSP60; uncharacterized protein A0ABQ8CGP7; chloroplastic chlorophyll a-b binding protein; uncharacterized protein A0ABQ8A2S7; chloroplastic photosystem I reaction center subunit IV A; uncharacterized LOC106347363 protein; chloroplastic protein curvature thylakoid 1A; photosystem II reaction center protein; chloroplastic photosystem II 10 kDa polypeptide); iii) *Calvin cycle* (fructose-bisphosphate aldolase; chaperonin HSP60; ribulose bisphosphate carboxylase large chain; chloroplastic ribulose bisphosphate carboxylase small subunit); iv) *organellar translation machinery and ribosome biogenesis* (tyrosine-tRNA ligase; large ribosomal subunit proteins bL9c and L6e; universal ribosomal proteins uL24 and uL11; rRNA 2'-O-methyltransferase fibrillarin 2; small ribosomal subunit protein uS9c; uncharacterized proteins A0ABQ7XJ37, A0ABQ8C4D6, A0ABQ8CHS6 and A0ABQ8A2Z8); v) *14-3-3 regulatory system* (14-3-3-like protein GF14 kappa; 14-3-3 family protein; 14-3-3-like protein GF14 psi; 14-3-3 domain-containing protein); vi) *calcium homeostasis* (uncharacterized protein A0ABQ7ZMD4; plasma membrane-associated cation-binding protein 1 isoforms A0ABQ7XBQ1 and A0ABQ8EII9); vii) *glutamate and aspartate group amino acid biosynthesis* (uncharacterized proteins A0ABQ8BBJ8 and A0ABQ7XXW7; glutamate decarboxylase; nitrogen regulatory protein P-II; 5-methyltetrahydropteroyltriglutamate-homocysteine S-methyltransferase); viii); *DNA-binding transcriptional regulation* (transcriptional factor B3 family protein 1; ferredoxin-thioredoxin reductase catalytic chain; uncharacterized proteins A0ABQ7ZG33, A0ABQ7XJ37 and A0ABQ7Y9V6); ix) *redox stress response* (uncharacterized proteins A0ABQ8E8Q4 and A0ABQ8BKU7; peroxidase A0A816I7N5; catalase A0A816NI65 and peptide-methionine S-oxide reductase); x) *phenolics biosynthesis* (dihydroflavonol reductase 1).

When down-represented proteins under the Shelf 2 condition were indexed by a functional assignment obtained by Mercator software analysis, they were associated with one/multiple function(s), except 97 that were not linked to a component name and/or a known function. According to their identity and numerosity, these proteins were related in order to *uncharacterized context*, *protein homeostasis*, *protein biosynthesis*, *photosynthesis*, *vesicle trafficking*, *protein modification*, *not assigned*, *protein physical control*, *redox homeostasis*, *amino acid metabolism* and other functional groups (Figure 3B). Within these families, bioinformatic analysis indicated specific enriched metabolic and stress-responsive activity subgroups (Supplementary Table S2), which included individual down-represented proteins as follow: i) *photophosphorylation* (kinesin-like protein; chloroplastic ATP-synthase subunit a; dihydrodipicolinate reductase N-terminal domain-containing protein; chlororespiratory reduction 41 protein; RuBisCO large subunit-binding protein subunit alpha; chloroplastic NAD(P)H-quinone oxidoreductase subunit M; photosynthetic NDH subunit of subcomplex B5; plastocyanin isoforms A0A816ZC43 and A0A816QW44; protein kinase domain-containing protein; cytochrome b6-f complex subunit 4; hypothetical protein A0A078FEV0; chloroplastic rhodanese-like domain-containing protein 4; protein kinase domain-containing protein; chlorophyll a-b binding protein isoforms Q2I0E4, A0A078ICK1 and A0A078GLT1; post-illumination chlorophyll fluorescence increase protein; thylakoid lumenal 17.9 kDa protein; beta-galactosidase protein; photosystem IP 700 chlorophyll a apoprotein A1; photosystem I subunit O; glutathione transferase protein; casein kinase II subunit beta protein; high chlorophyll fluorescence phenotype 173 protein; glutathione transferase protein A0ABQ7Y0V4; uncharacterized protein A0A816XSM9; chloroplastic zeaxanthin epoxidase; histone acetyltransferase A0A816TP41; CASP-like protein; photosystem II D2 protein; peptidase M3A/M3B catalytic domain-containing protein; chloroplastic protein MET1; maintenance PSII under high light 1-like protein); ii) *Calvin cycle* (phosphoribulokinase isoforms A0A078HT68 and A0ABQ7ZHQ8; ribose-5-phosphate isomerase; 4a-Hydroxytetrahydrobiopterinde hydratase; trichome birefringence-like N-terminal domain-containing protein; RuBisCO large subunit-binding protein subunit alpha; ribosomal RNA-processing protein 42; uncharacterized protein A0ABQ8D036; sedoheptulose-1,7-bisphosphatase); iii) *light response* (cupin type-1 domain-containing protein; protein kinase domain-containing protein; scarecrow-like protein 5; uncharacterized protein A0ABQ8E4D6; phytochrome B-like; blue light photoreceptor-cryptochrome photoreceptor CRY; non-specific Ser/Thr protein kinase); iv) *proteolysis* (prolyl endopeptidase; peptidase A1 domain-containing protein; peptidase A1 family; legumain protein; cathepsin B-like protease 2; peptidase C1 family; cysteine protease XCP2; ATP-dependent zinc metalloprotease FTSH7; leucine aminopeptidase; uncharacterized proteins A0ABQ7X1J6 and A0ABQ8AIR4; chaperone protein Clp C2; ATP-dependent Clp protease ATP-binding subunit CLPT1 and proteolytic subunits A0A078HEJ2 and A0ABQ7YQD0; subtilisin-like protease SBT1.3; peptidase S8 family; carboxypeptidase protein isoforms A0ABQ8BA80 and A0ABQ8D7Z8); v) *ubiquitin-proteasome system* (regulatory particle non-ATPase 13 protein; AAA+ ATPase domain-containing protein; 26S proteasome non-ATPase regulatory subunit 13 homolog B-like; proteasome subunits 4, beta and alpha type-4-A-like; proteasome endopeptidase complex; plant UBX domain-containing protein10-like; peptide-N(4)-(N-acetyl-beta-glucosaminyl)asparagine amidase; phospholipase A-2-activating protein; defective in cullin neddylation protein; SKP1-like protein; cullin-associated NEDD8-dissociated protein 1; protein kinase domain-containing protein; DEK-C domain-containing protein; pre-mRNA-processing factor 19; WD repeat-containing protein DWA2; ubiquitin-like domain-containing protein; RING-type E3 ubiquitin transferase; pre-mRNA-processing factor 19; pectate lyase; ubiquitin-fold modifier 1; COP9 signalosome complex subunits 2, 3 and 5; OTU domain-containing protein; ubiquitinyl hydrolase 1 protein isoforms A0ABQ8EQY2 and A0ABQ8E4T5); vi) *protein phosphorylation/dephosphorylation* (ABC1 atypical kinase-like domain-containing protein isoforms A0ABQ8DP47 and A0ABQ8DP47; non-specific Ser/Thr protein kinase isoforms A0A078H5I3, A0A816ZBX6, A0A816RME9, A0A816UHF7 and A0ABQ8A7S2; protein-Ser/Thr phospatase A0ABQ8B5I2, A0ABQ7ZR20 and A0A078IYJ1; cyclin-dependent kinase; casein kinase II subunit alpha and beta protein; Mn-dependent ADP-ribose/CDP-alcohol diphosphatase protein; HTHcro/C1-type domain-containing protein; protein kinase domain-containing protein isoforms A0ABQ7X6P1, A0ABQ8EDM9, A0ABQ7ZNS7 and A0ABQ7X6P1; receptor-like Ser/Thr-protein kinase and EF-hand domain-containing proteins A0ABQ8A7D1 and A0ABQ8D2H7); vii) *organellar translation machinery and ribosome biogenesis* (glycine-tRNA ligase; methionine-tRNA ligase protein; serine-tRNA ligase protein; thioredoxin domain-containing protein; Sas10C-terminal domain-containing protein; large ribosomal subunit proteins uL4c and uL4m; chloroplastic import inner membrane translocase subunit HP30-1; uncharacterized proteins A0ABQ8B7M0, A0ABQ8A6U5 and A0ABQ7YIA5; 50S ribosomal proteins L22 and 23; folate gamma-glutamyl hydrolase protein, histone chaperone ASF1-like; small ribosomal subunit protein S1 and bS18c; 30S ribosomal protein 3; glutamine amidotransferase type-2 domain-containing protein; phosphoribosylformyl glycinamidine synthase; 60S acidic ribosomal proteins P1 and L28-1; plectin/eS10N-terminal domain-containing protein; 40S ribosomal proteins S19 and S6; receptor for activated C kinase 1B; universal ribosomal protein uS5 family); viii) *protein chaperone activities* (SHSP domain-containing protein isoforms A0ABQ8DP32 and A0A817B1J5, heat shock 70 kDa protein 16, SUN domain-containing protein, uncharacterized protein A0ABQ7XRY7, trichome birefringence-like N-terminal domain-containing protein, J domain-containing protein, nascent polypeptide-associated complex subunit alpha-like protein 1, 3 and beta); ix) *redox stress response* (inositol-1-monophosphatase; L-gulonolactoneoxidase protein; L-ascorbate peroxidase; glutathione reductase; monodehydroascorbate reductase (NADH) protein A0ABQ8C4F8 and A0A078FGS0; peroxidase proteins A0ABQ8A8C9, Q4PJU0, A0ABQ8BAP4 and A0A078FAL2; catalase isoforms A0A816R4K3, A0ABQ8CMJ5, A0ABQ8EGF2 and A0ABQ8B7K2; superoxide dismutase isoforms A0A816JH76 and A0A078HHJ9); x) *glutamate and aspartate group amino acid biosynthesis* (asparagine synthetase [glutamine-hydrolyzing]; bifunctional aspartokinase/homoserine dehydrogenase 1; acetolactate synthase I/III small subunit; diaminopimelate epimerase; 4-hydroxy-tetrahydrodipicolinate synthase protein; LL-diaminopimelate aminotransferase; nucleoside phosphorylase domain-containing protein; non-specific Ser/Thr protein kinase; threonine synthase protein; acetylglutamate kinase; histidine biosynthesis bifunctional protein hisIE; imidazoleglycerol-phosphate synthase; ribose-phosphate diphosphokinase); xi) *phenolics biosynthesis* (cinnamoyl-CoA reductase 1; glutathione transferase; chalcone-flavonone isomerase family protein; Fe2OG dioxygenase domain-containing protein).

**Supplementary Table S1** - Protein identification and differentially represented proteins (DRPs) in cress microgreens grown under Shelf 1 and Shelf 2 LED treatments. Proteomic ANOVA identified 787 DRPs (adjusted p < 0.05), with 151 over‑represented (pink) and 636 down‑represented (green) in Shelf 2 relative to Shelf 1. The table includes protein accession, description, key identification metrics, basic protein properties, Mascot data, gene symbol, and the Shelf 2A/Shelf 1A abundance ratio with corresponding p- and adjusted p-values.

| Accession | Description | Exp. q-value: Combined | Sum PEP Score | Coverage [%] | # Peptides | # PSMs | # Unique Peptides | # AAs | MW [kDa] | calc. pI | Score Mascot: Mascot | # Peptides (by Search Engine): Mascot | Gene Symbol | # Razor Peptides | Abundance Ratio: (SHELF_2A) / (SHELF_1A) | Abundance Ratio P-Value: (SHELF_2A) / (SHELF_1A) | Abundance Ratio Adj. P-Value: (SHELF_2A) / (SHELF_1A) |
| --- | --- | --- | --- | --- | --- | --- | --- | --- | --- | --- | --- | --- | --- | --- | --- | --- | --- |
| A0A078F495 | Protein disulfide-isomerase OS=Brassica napus OX=3708 GN=BnaA06g15500D PE=3 SV=1 | 0 | 5.944 | 5 | 2 | 35 | 1 | 498 | 55.5 | 5.35 | 344 | 2 | BnaA06g15500D | 0 | 10.765 | 0.02048781 | 0.090313268 |
| A0A078F6Y1 | 14-3-3-like protein GF14 kappa, (rape) hypothetical protein OS=Brassica napus OX=3708 GN=BnaA06g24300D PE=3 SV=1 | 0 | 33.02 | 44 | 10 | 166 | 4 | 248 | 28 | 4.89 | 1817 | 10 | BnaA06g24300D | 5 | 4.569 | 0.028681204 | 0.118805498 |
| A0A078F7A7 | Heat shock protein 70 family, chloroplast, BnaC08g11440D protein OS=Brassica napus OX=3708 GN=BnaC08g11440D PE=3 SV=1 | 0 | 55.006 | 25 | 15 | 187 | 4 | 621 | 66.7 | 4.97 | 1862 | 15 | BnaC08g11440D | 0 | 5.83 | 0.020822655 | 0.091676155 |
| A0A078F8S4 | Pathogenesis-related protein Bet v I family, (rape) hypothetical protein OS=Brassica napus OX=3708 GN=BnaC02g20340D PE=4 SV=1 | 0 | 2.9 | 7 | 2 | 17 | 2 | 229 | 25.3 | 5.48 | 128 | 2 | BnaC02g20340D | 0 | 12.714 | 0.00153354 | 0.008219285 |
| A0A078F9R8 | Ferritin OS=Brassica napus OX=3708 GN=BnaA03g00260D PE=3 SV=1 | 0 | 17.116 | 19 | 3 | 29 | 2 | 254 | 28.2 | 5.94 | 260 | 3 | BnaA03g00260D; Fer1 | 0 | 5.399 | 0.03128764 | 0.128165081 |
| A0A078FBJ6 | Tubulin-specific chaperone A OS=Brassica napus OX=3708 GN=BnaA05g12000D PE=3 SV=1 | 0 | 1.891 | 9 | 1 | 10 | 1 | 110 | 12.4 | 5.24 | 101 | 1 | BnaA05g12000D | 0 | 10.603 | 0.003278595 | 0.016887914 |
| A0A078FCT5 | Cytochrome b5 family protein, BnaC04g12100D protein OS=Brassica napus OX=3708 GN=BnaC04g12100D PE=3 SV=1 | 0.004 | 1.188 | 10 | 1 | 1 | 1 | 156 | 17.4 | 5.34 | 0 | 1 | BnaC04g12100D | 0 | 100 | 1E-17 | 5.5977E-17 |
| A0A078FDK2 | Peptide-methionine (S)-S-oxide reductase OS=Brassica napus OX=3708 GN=BnaA03g47160D PE=3 SV=1 | 0 | 9.696 | 14 | 3 | 20 | 3 | 254 | 28.3 | 7.72 | 297 | 3 | BnaA03g47160D | 0 | 4.766 | 0.033726649 | 0.136460468 |
| A0A078FIU2 | Nucleoside diphosphate kinase OS=Brassica napus OX=3708 GN=BnaA09g17360D PE=3 SV=1 | 0 | 16.024 | 20 | 2 | 91 | 2 | 148 | 16.4 | 6.79 | 1212 | 2 | Bc-NDPK1; BcNDK I; BnaA09g17360D | 0 | 4.11 | 0.038302512 | 0.151569017 |
| A0A078G4M4 | Large ribosomal subunit protein bL9c OS=Brassica napus OX=3708 GN=BnaC01g24010D PE=3 SV=1 | 0 | 2.652 | 5 | 1 | 3 | 1 | 196 | 22 | 9.8 | 59 | 1 | BnaC01g24010D | 0 | 6.847 | 0.008016076 | 0.03852175 |
| A0A078G5W3 | Trichohyalin-like, (rape) hypothetical protein OS=Brassica napus OX=3708 GN=BnaC01g35980D PE=4 SV=1 | 0.001 | 1.441 | 1 | 1 | 7 | 1 | 680 | 78.6 | 7.39 | 0 | 1 | BnaC01g35980D | 0 | 8.598 | 0.007942982 | 0.038187621 |
| A0A078GLC7 | E3 ubiquitin-protein ligase RBBP6, (rape) hypothetical protein OS=Brassica napus OX=3708 GN=BnaC09g20200D PE=4 SV=1 | 0.003 | 1.287 | 2 | 1 | 2 | 1 | 875 | 97.6 | 8.51 | 0 | 1 | BnaC09g20200D | 0 | 8.847 | 0.007428082 | 0.03583272 |
| A0A078GP99 | Universal ribosomal protein uL11 family, BnaA02g10390D protein OS=Brassica napus OX=3708 GN=BnaA02g10390D PE=3 SV=1 | 0 | 5.31 | 13 | 2 | 31 | 2 | 167 | 18.3 | 9.61 | 243 | 2 | BnaA02g10390D | 0 | 7.173 | 0.010660518 | 0.050007351 |
| A0A078GUK1 | Universal ribosomal protein uL24 family, (rape) hypothetical protein OS=Brassica napus OX=3708 GN=BnaA02g09540D PE=3 SV=1 | 0 | 5.68 | 12 | 2 | 11 | 2 | 201 | 22.3 | 9.86 | 45 | 2 | BnaA02g09540D | 0 | 7.455 | 0.015054069 | 0.06840089 |
| A0A078GVL1 | 14-3-3 family protein, BnaCnng05840D protein OS=Brassica napus OX=3708 GN=BnaCnng05840D PE=3 SV=1 | 0 | 45.276 | 40 | 10 | 184 | 3 | 261 | 29.4 | 4.78 | 2422 | 10 | BnaCnng05840D | 1 | 6.92 | 0.014152215 | 0.064783947 |
| A0A078GYM1 | Cathepsin H OS=Brassica napus OX=3708 GN=BnaA10g13210D PE=3 SV=1 | 0 | 15.306 | 16 | 4 | 52 | 3 | 359 | 39.4 | 5.68 | 256 | 4 | BnaA10g13210D | 1 | 4.229 | 0.040535432 | 0.158098514 |
| A0A078GZH9 | Chaperonin (HSP60) (rape) hypothetical protein OS=Brassica napus OX=3708 GN=BnaA04g16190D PE=3 SV=1 | 0 | 49.831 | 28 | 15 | 188 | 1 | 585 | 61.8 | 5.24 | 4055 | 15 | BnaA04g16190D | 0 | 8.514 | 0.004579061 | 0.022957445 |
| A0A078H0E3 | Phytepsin, (rape) hypothetical protein OS=Brassica napus OX=3708 GN=BnaC08g15160D PE=3 SV=1 | 0 | 42.511 | 24 | 9 | 134 | 1 | 595 | 64.1 | 6.18 | 2865 | 9 | BnaC08g15160D | 0 | 4.592 | 0.035400825 | 0.142695425 |
| A0A078H163 | Pyruvate dehydrogenase E1 component subunit alpha OS=Brassica napus OX=3708 GN=BnaA09g51510D PE=4 SV=1 | 0 | 15.47 | 14 | 5 | 21 | 1 | 425 | 46.9 | 7.71 | 230 | 5 | BnaA09g51510D | 0 | 7.297 | 0.02976982 | 0.122677791 |
| A0A078H697 | Uncharacterized LOC106345859, BnaA07g04430D protein OS=Brassica napus OX=3708 GN=BnaA07g04430D PE=4 SV=1 | 0.003 | 1.296 | 11 | 1 | 3 | 1 | 127 | 14 | 8.43 | 64 | 1 | BnaA07g04430D | 0 | 11.076 | 0.008528052 | 0.040826428 |
| A0A078H7S5 | Malate dehydrogenase OS=Brassica napus OX=3708 GN=BnaA09g16400D PE=3 SV=1 | 0 | 32.234 | 37 | 11 | 134 | 1 | 332 | 35.5 | 6.55 | 1440 | 11 | BnaA09g16400D | 0 | 8.25 | 0.00454654 | 0.022806252 |
| A0A078H8W8 | Transmembrane emp24 domain-containing protein p24 beta3, (rape) hypothetical protein OS=Brassica napus OX=3708 GN=BnaA01g24430D PE=3 SV=1 | 0.004 | 1.196 | 5 | 1 | 2 | 1 | 218 | 24.7 | 6.44 | 37 | 1 | BnaA01g24430D | 0 | 5.444 | 0.030405181 | 0.124717115 |
| A0A078HKF7 | Fructose-bisphosphate aldolase OS=Brassica napus OX=3708 GN=BnaC02g33660D PE=3 SV=1 | 0 | 22.74 | 25 | 6 | 61 | 4 | 389 | 42.3 | 8.32 | 720 | 6 | BnaC02g33660D | 0 | 8.26 | 0.038495131 | 0.151826554 |
| A0A078HZ79 | Chlorophyll a-b binding protein, chloroplastic OS=Brassica napus OX=3708 GN=BnaA07g07570D PE=3 SV=1 | 0 | 35.556 | 56 | 3 | 81 | 1 | 99 | 10.5 | 4.72 | 2335 | 3 | BnaA07g07570D | 0 | 11.249 | 0.0017313 | 0.009231908 |
| A0A078I1G2 | ATP synthase subunit delta', mitochondrial OS=Brassica napus OX=3708 GN=BnaAnng07210D PE=3 SV=1 | 0 | 1.873 | 5 | 1 | 7 | 1 | 203 | 21.5 | 6.54 | 56 | 1 | BnaAnng07210D | 0 | 10.871 | 0.002214314 | 0.01164948 |
| A0A078I3R9 | Ribulose bisphosphate carboxylase large chain, BnaCnng12440D protein OS=Brassica napus OX=3708 GN=BnaCnng12440D PE=4 SV=1 | 0.009 | 1.038 | 10 | 1 | 10 | 1 | 72 | 8.4 | 9.52 | 82 | 1 | BnaCnng12440D | 0 | 13.565 | 0.000714066 | 0.003894377 |
| A0A078II13 | Epithiospecifier protein-like, BnaAnng10080D protein OS=Brassica napus OX=3708 GN=BnaAnng10080D PE=4 SV=1 | 0 | 2.572 | 4 | 2 | 9 | 2 | 343 | 37.9 | 6.39 | 26 | 2 | BnaAnng10080D | 0 | 9.139 | 0.002986764 | 0.015429213 |
| A0A078IJL7 | rRNA 2'-O-methyltransferase fibrillarin 2, BnaA03g47570D protein OS=Brassica napus OX=3708 GN=BnaA03g47570D PE=3 SV=1 | 0 | 4.288 | 11 | 3 | 16 | 2 | 313 | 33.1 | 10.08 | 118 | 3 | BnaA03g47570D | 0 | 5.572 | 0.02979923 | 0.122677791 |
| A0A078ILM3 | Uncharacterized LOC106347363, (rape) hypothetical protein OS=Brassica napus OX=3708 GN=BnaA06g15210D PE=4 SV=1 | 0 | 1.782 | 6 | 1 | 7 | 1 | 125 | 13.4 | 10.33 | 105 | 1 | BnaA06g15210D | 0 | 9.089 | 0.010219786 | 0.048140156 |
| A0A078IYV5 | (S)-2-Hydroxy-acid oxidase OS=Brassica napus OX=3708 GN=BnaA03g58240D PE=3 SV=1 | 0 | 19.35 | 19 | 7 | 150 | 2 | 368 | 40.6 | 8.59 | 1233 | 7 | BnaA03g58240D | 0 | 5.893 | 0.01340718 | 0.061795882 |
| A0A078J5R3 | Sorbitol dehydrogenase, (rape) hypothetical protein OS=Brassica napus OX=3708 GN=BnaCnng32750D PE=3 SV=1 | 0.003 | 1.27 | 3 | 1 | 3 | 1 | 364 | 39 | 6.77 | 0 | 1 | BnaCnng32750D | 0 | 8.887 | 0.041108469 | 0.159376203 |
| A0A078JCL4 | Dehydration-responsive protein RD22-like, (rape) hypothetical protein OS=Brassica napus OX=3708 GN=BnaA06g39340D PE=4 SV=1 | 0.007 | 1.081 | 2 | 1 | 2 | 1 | 388 | 41.6 | 9.28 | 0 | 1 | BnaA06g39340D | 0 | 11.163 | 0.017924988 | 0.080439087 |
| A0A078JCZ3 | Small ribosomal subunit protein uS9c OS=Brassica napus OX=3708 GN=BnaAnng19390D PE=3 SV=1 | 0 | 4.974 | 12 | 2 | 17 | 2 | 212 | 22.9 | 10.26 | 247 | 2 | BnaAnng19390D | 0 | 4.641 | 0.031469005 | 0.128637513 |
| A0A078JKN0 | Large subunit ribosomal protein L6e, (rape) hypothetical protein OS=Brassica napus OX=3708 GN=BnaAnng22110D PE=3 SV=1 | 0 | 2.789 | 6 | 2 | 4 | 2 | 233 | 25.9 | 10.2 | 32 | 2 | BnaAnng22110D | 0 | 23.622 | 0.0001561 | 0.000867906 |
| A0A078JNV6 | Nitrogen regulatory protein P-II, (rape) hypothetical protein OS=Brassica napus OX=3708 GN=BnaCnng57780D PE=4 SV=1 | 0.004 | 1.214 | 20 | 1 | 9 | 1 | 54 | 5.9 | 7.53 | 60 | 1 | BnaCnng57780D | 0 | 7.531 | 0.006893299 | 0.033380703 |
| A0A078JQI2 | Ribulose bisphosphate carboxylase small subunit, chloroplastic OS=Brassica napus OX=3708 GN=BnaCnng55860D PE=3 SV=1 | 0 | 25.213 | 32 | 5 | 165 | 1 | 181 | 20.3 | 8.05 | 2616 | 5 | BnaCnng55860D; RBCS | 0 | 7.375 | 0.006643904 | 0.032289339 |
| A0A078JTB5 | Late embryogenesis abundant protein, group 3, (rape) hypothetical protein OS=Brassica napus OX=3708 GN=BnaAnng35040D PE=4 SV=1 | 0 | 1.514 | 3 | 1 | 13 | 1 | 297 | 32.3 | 5.73 | 153 | 1 | BnaAnng35040D | 0 | 15.563 | 0.002561024 | 0.013394585 |
| A0A1B1XZH5 | Photosystem II reaction center protein L OS=Brassica napus var. napus OX=138011 GN=psbL PE=3 SV=1 | 0 | 11.683 | 37 | 1 | 28 | 1 | 38 | 4.5 | 4.5 | 910 | 1 | psbL | 0 | 7.632 | 0.008578671 | 0.041059587 |
| A0A816HVR2 | Malic enzyme OS=Brassica napus OX=3708 GN=DARMORV10_C03P05810.1 PE=3 SV=1 | 0 | 29.931 | 7 | 8 | 119 | 3 | 1496 | 165.2 | 7.65 | 986 | 8 | DARMORV10_C03P05810.1 | 0 | 8.607 | 0.004109912 | 0.020903679 |
| A0A816I3S1 | Nitrogen regulatory protein P-II, (rape) hypothetical protein OS=Brassica napus OX=3708 GN=DARMORV10_C03P38310.1 PE=3 SV=1 | 0 | 4.03 | 9 | 2 | 15 | 2 | 197 | 21.5 | 9.55 | 61 | 2 | DARMORV10_C03P38310.1 | 0 | 11.715 | 0.001913684 | 0.010115055 |
| A0A816I7N5 | Peroxidase OS=Brassica napus OX=3708 GN=DARMORV10_C03P34140.1 PE=3 SV=1 | 0 | 8.795 | 9 | 2 | 37 | 2 | 352 | 38.9 | 7.9 | 227 | 2 | DARMORV10_C03P34140.1 | 0 | 7.253 | 0.012725664 | 0.058971785 |
| A0A816I7Y6 | Protein CURVATURE THYLAKOID 1A, chloroplastic, (rape) hypothetical protein OS=Brassica napus OX=3708 GN=DARMORV10_C03P38930.1 PE=4 SV=1 | 0 | 8.997 | 13 | 3 | 27 | 3 | 205 | 22.7 | 9.8 | 285 | 3 | DARMORV10_C03P38930.1 | 0 | 5.038 | 0.024991184 | 0.106083814 |
| A0A816I9B4 | Protein LONGIFOLIA 2, (rape) hypothetical protein OS=Brassica napus OX=3708 GN=DARMORV10_C03P39940.1 PE=4 SV=1 | 0 | 1.866 | 3 | 1 | 1 | 1 | 864 | 97.1 | 9.5 | 0 | 1 | DARMORV10_C03P39940.1 | 0 | 100 | 1E-17 | 5.5977E-17 |
| A0A816I9U9 | T-complex protein 1 subunit alpha OS=Brassica napus OX=3708 GN=DARMORV10_C03P50870.1 PE=3 SV=1 | 0 | 14.937 | 9 | 6 | 49 | 1 | 958 | 104.9 | 6.28 | 625 | 6 | DARMORV10_C03P50870.1 | 0 | 6.883 | 0.011414473 | 0.053415446 |
| A0A816IAK2 | Chaperonin CPN60, mitochondrial, (rape) hypothetical protein OS=Brassica napus OX=3708 GN=DARMORV10_C03P53460.1 PE=3 SV=1 | 0 | 19.333 | 16 | 9 | 85 | 2 | 577 | 61.3 | 5.76 | 614 | 9 | DARMORV10_C03P53460.1 | 0 | 7.503 | 0.026760128 | 0.111973446 |
| A0A816IES6 | FACT complex subunit OS=Brassica napus OX=3708 GN=DARMORV10_C03P35150.1 PE=3 SV=1 | 0.004 | 1.203 | 1 | 1 | 1 | 1 | 1177 | 132.5 | 6.05 | 26 | 1 | DARMORV10_C03P35150.1 | 0 | 100 | 1E-17 | 5.5977E-17 |
| A0A816IJ81 | Co-chaperone protein p23-1 OS=Brassica napus OX=3708 GN=DARMORV10_C09P01990.1 PE=3 SV=1 | 0 | 3.987 | 2 | 2 | 32 | 2 | 852 | 96.2 | 6.1 | 503 | 2 | DARMORV10_C09P01990.1 | 0 | 6.715 | 0.011590695 | 0.053992482 |
| A0A816J6H8 | Uncharacterized LOC106412569 (rape) hypothetical protein OS=Brassica napus OX=3708 GN=DARMORV10_C09P66120.1 PE=4 SV=1 | 0.007 | 1.087 | 5 | 1 | 2 | 1 | 358 | 39.1 | 7.46 | 31 | 1 | DARMORV10_C09P66120.1 | 0 | 15.791 | 0.000909003 | 0.004947454 |
| A0A816J847 | Protein OCTOPUS-like, (rape) hypothetical protein OS=Brassica napus OX=3708 GN=DARMORV10_C09P74550.1 PE=4 SV=1 | 0.006 | 1.135 | 4 | 1 | 2 | 1 | 544 | 60.4 | 8.92 | 0 | 1 | DARMORV10_C09P74550.1 | 0 | 100 | 1E-17 | 5.5977E-17 |
| A0A816JC23 | Aldo-keto reductase family 4 member C10, (rape) hypothetical protein OS=Brassica napus OX=3708 GN=DARMORV10_C04P10080.1 PE=3 SV=1 | 0.002 | 1.35 | 3 | 1 | 7 | 1 | 314 | 35.1 | 5.9 | 0 | 1 | DARMORV10_C04P10080.1 | 0 | 8.862 | 0.006647712 | 0.032289339 |
| A0A816JD42 | Ras-related protein RABA4a, (rape) hypothetical protein OS=Brassica napus OX=3708 GN=DARMORV10_C09P67060.1 PE=3 SV=1 | 0 | 5.076 | 14 | 3 | 30 | 1 | 223 | 24.4 | 5.99 | 188 | 3 | 80A08_30 | 0 | 5.129 | 0.033867135 | 0.136977157 |
| A0A816JS97 | COP1-interactive protein 1, (rape) hypothetical protein OS=Brassica napus OX=3708 GN=DARMORV10_C04P17390.1 PE=4 SV=1 | 0 | 19.014 | 6 | 8 | 61 | 2 | 1457 | 162.7 | 4.82 | 717 | 8 | DARMORV10_C04P17390.1 | 0 | 5.368 | 0.02956888 | 0.122034275 |
| A0A816JSF1 | Ferredoxin-thioredoxin reductase catalytic chain, chloroplastic OS=Brassica napus OX=3708 GN=DARMORV10_C04P46660.1 PE=3 SV=1 | 0.005 | 1.176 | 3 | 1 | 4 | 1 | 296 | 33.7 | 9.57 | 45 | 1 | DARMORV10_C04P46660.1 | 0 | 10.658 | 0.003536865 | 0.018126752 |
| A0A816JSP8 | Glutamate decarboxylase OS=Brassica napus OX=3708 GN=DARMORV10_C04P16840.1 PE=3 SV=1 | 0 | 41.766 | 18 | 6 | 98 | 2 | 494 | 55.9 | 6.57 | 1802 | 6 | DARMORV10_C04P16840.1 | 0 | 4.864 | 0.03591128 | 0.143994558 |
| A0A816JVR0 | Histone H1.2, (rape) hypothetical protein OS=Brassica napus OX=3708 GN=DARMORV10_C04P59660.1 PE=3 SV=1 | 0 | 3.319 | 4 | 1 | 30 | 1 | 272 | 28.4 | 10.93 | 916 | 1 | DARMORV10_C04P59660.1 | 0 | 5.906 | 0.023293872 | 0.099609076 |
| A0A816JWT6 | Uncharacterized protein At2g37660, chloroplastic, (rape) hypothetical protein OS=Brassica napus OX=3708 GN=DARMORV10_C04P66100.1 PE=3 SV=1 | 0 | 21.543 | 21 | 5 | 71 | 4 | 329 | 35.5 | 8.92 | 592 | 5 | DARMORV10_C04P66100.1 | 1 | 3.742 | 0.04900542 | 0.185229414 |
| A0A816K122 | Ribulose bisphosphate carboxylase small subunit, chloroplastic OS=Brassica napus OX=3708 GN=RBCS PE=3 SV=1 | 0 | 23.524 | 43 | 6 | 112 | 3 | 181 | 20.3 | 7.71 | 1877 | 6 | RBCS | 1 | 7.648 | 0.005884414 | 0.02892664 |
| A0A816K9F9 | Probable calreticulin, (rape) hypothetical protein OS=Brassica napus OX=3708 GN=DARMORV10_C02P37650.1 PE=3 SV=1 | 0 | 4.351 | 10 | 2 | 15 | 1 | 182 | 20.5 | 6.93 | 191 | 2 | DARMORV10_C02P37650.1 | 0 | 13.54 | 0.001547917 | 0.008290121 |
| A0A816KF65 | Small monomeric GTPase OS=Brassica napus OX=3708 GN=DARMORV10_C05P07200.1 PE=3 SV=1 | 0 | 35.249 | 26 | 14 | 153 | 2 | 616 | 70.2 | 4.79 | 1555 | 14 | DARMORV10_C05P07200.1 | 2 | 4.751 | 0.032875737 | 0.133496551 |
| A0A816KNP2 | K(+) efflux antiporter 1, chloroplastic-like, (rape) hypothetical protein OS=Brassica napus OX=3708 GN=DARMORV10_C05P00270.1 PE=3 SV=1 | 0 | 1.638 | 1 | 1 | 1 | 1 | 1155 | 124.1 | 5.07 | 0 | 1 | DARMORV10_C05P00270.1 | 0 | 3.904 | 0.049021654 | 0.185229414 |
| A0A816L182 | Myosin-6, (rape) hypothetical protein OS=Brassica napus OX=3708 GN=DARMORV10_C05P03650.1 PE=4 SV=1 | 0.004 | 1.222 | 1 | 1 | 4 | 1 | 824 | 92.9 | 5.34 | 31 | 1 | DARMORV10_C05P03650.1 | 0 | 8.429 | 0.009379272 | 0.044356441 |
| A0A816LDM1 | Probable DUF7081 domain-containing protein, (rape) hypothetical protein OS=Brassica napus OX=3708 GN=DARMORV10_C05P40720.1 PE=4 SV=1 | 0 | 5.895 | 7 | 4 | 14 | 3 | 694 | 78.5 | 6.92 | 97 | 4 | DARMORV10_C05P40720.1 | 1 | 4.919 | 0.040299564 | 0.157372795 |
| A0A816LK77 | TPR repeat-containing thioredoxin TDX OS=Brassica napus OX=3708 GN=DARMORV10_C05P48420.1 PE=3 SV=1 | 0.008 | 1.078 | 2 | 1 | 1 | 1 | 378 | 42.5 | 5.72 | 25 | 1 | DARMORV10_C05P48420.1 | 0 | 11.098 | 0.011301669 | 0.052933805 |
| A0A816N162 | Exocyst subunit Exo70 family protein OS=Brassica napus OX=3708 GN=DARMORV10_C07P44770.1 PE=3 SV=1 | 0.003 | 1.303 | 1 | 1 | 1 | 1 | 952 | 102.6 | 5.63 | 30 | 1 | DARMORV10_C07P44770.1 | 0 | 11.443 | 0.016244604 | 0.073251131 |
| A0A816N2A6 | Probable polygalacturonase, (rape) hypothetical protein OS=Brassica napus OX=3708 GN=DARMORV10_C07P52750.1 PE=3 SV=1 | 0 | 2.108 | 3 | 1 | 5 | 1 | 493 | 54.4 | 6.43 | 58 | 1 | DARMORV10_C07P52750.1 | 0 | 100 | 1E-17 | 5.5977E-17 |
| A0A816NI65 | Catalase (Fragment) OS=Brassica napus OX=3708 GN=DARMORV10_C07P60920.1 PE=3 SV=1 | 0 | 53.651 | 38 | 13 | 263 | 1 | 471 | 54.4 | 6.83 | 2819 | 13 | DARMORV10_C07P60920.1 | 1 | 5.671 | 0.022703736 | 0.097985024 |
| A0A816P5U2 | Dihydroflavonol reductase 1, (rape) hypothetical protein (Fragment) OS=Brassica napus OX=3708 GN=DARMORV10_A09P34160.1 PE=3 SV=1 | 0.006 | 1.129 | 3 | 1 | 2 | 1 | 346 | 38.4 | 5.73 | 17 | 1 | DARMORV10_A09P34160.1 | 0 | 5.614 | 0.021912201 | 0.095356345 |
| A0A816PJ92 | Probable transcriptional factor B3 family protein 1, (rape) hypothetical protein OS=Brassica napus OX=3708 GN=DARMORV10_A09P56140.1 PE=4 SV=1 | 0.001 | 1.419 | 1 | 1 | 7 | 1 | 1158 | 131.1 | 7.52 | 73 | 1 | DARMORV10_A09P56140.1 | 0 | 8.713 | 0.005602534 | 0.027667918 |
| A0A816PTZ9 | Probable 6-phosphofructo-2-kinase/fructose-2, 6-bisphosphatase, (rape) hypothetical protein OS=Brassica napus OX=3708 GN=DARMORV10_A09P66600.1 PE=4 SV=1 | 0 | 4.707 | 4 | 3 | 26 | 1 | 795 | 87.3 | 7.59 | 220 | 3 | DARMORV10_A09P66600.1 | 0 | 10.142 | 0.031941498 | 0.130122131 |
| A0A816Q4L2 | RNA-binding protein 8A (Fragment) OS=Brassica napus OX=3708 GN=DARMORV10_C06P13130.1 PE=3 SV=1 | 0 | 5.419 | 9 | 2 | 21 | 2 | 221 | 24.5 | 6 | 608 | 2 | DARMORV10_C06P13130.1 | 0 | 4.606 | 0.039591522 | 0.1551229 |
| A0A816R2X7 | Protein-serine/threonine phosphatase OS=Brassica napus OX=3708 GN=DARMORV10_C01P07180.1 PE=3 SV=1 | 0 | 7.24 | 16 | 3 | 17 | 3 | 308 | 32.9 | 4.92 | 126 | 3 | DARMORV10_C01P07180.1 | 0 | 7.994 | 0.007859929 | 0.037847767 |
| A0A816R5K5 | Methyltransferase OS=Brassica napus OX=3708 GN=DARMORV10_C01P11880.1 PE=3 SV=1 | 0 | 1.653 | 3 | 1 | 1 | 1 | 620 | 70.2 | 8.37 | 0 | 1 | DARMORV10_C01P11880.1 | 0 | 30.65 | 0.007283872 | 0.035160803 |
| A0A816R943 | Photosystem I reaction center subunit IV A, chloroplastic, (rape) hypothetical protein OS=Brassica napus OX=3708 GN=DARMORV10_C01P10750.1 PE=3 SV=1 | 0 | 29.958 | 29 | 5 | 48 | 5 | 143 | 15 | 9.92 | 1351 | 5 | DARMORV10_C01P10750.1 | 0 | 4.518 | 0.029205389 | 0.120860001 |
| A0A816S1D1 | Uncharacterized protein At4g28440, (rape) hypothetical protein OS=Brassica napus OX=3708 GN=DARMORV10_A06P07410.1 PE=4 SV=1 | 0 | 2.347 | 9 | 1 | 14 | 1 | 142 | 15.6 | 8.13 | 0 | 1 | DARMORV10_A06P07410.1 | 0 | 8.227 | 0.008380359 | 0.040137312 |
| A0A816S7X4 | Probable myosin, (rape) hypothetical protein OS=Brassica napus OX=3708 GN=DARMORV10_A06P05650.1 PE=3 SV=1 | 0 | 2.446 | 1 | 2 | 10 | 1 | 1780 | 202.8 | 8.94 | 0 | 2 | DARMORV10_A06P05650.1 | 1 | 5.465 | 0.023247742 | 0.099609076 |
| A0A816UDQ8 | Plasma membrane ATPase OS=Brassica napus OX=3708 GN=DARMORV10_C08P17790.1 PE=3 SV=1 | 0 | 5.669 | 3 | 3 | 19 | 1 | 944 | 103.7 | 6.32 | 81 | 3 | DARMORV10_C08P17790.1 | 0 | 100 | 1E-17 | 5.5977E-17 |
| A0A816UDU9 | Importin subunit alpha OS=Brassica napus OX=3708 GN=DARMORV10_C08P12580.1 PE=3 SV=1 | 0 | 4.6 | 6 | 3 | 28 | 2 | 543 | 59.9 | 5.17 | 108 | 3 | DARMORV10_C08P12580.1 | 0 | 4.529 | 0.042534527 | 0.163955718 |
| A0A816VBH8 | Chaperone protein dnaJ 3-like, (rape) hypothetical protein OS=Brassica napus OX=3708 GN=DARMORV10_A03P10570.1 PE=3 SV=1 | 0 | 4.902 | 8 | 3 | 17 | 1 | 408 | 45.5 | 7.05 | 126 | 3 | DNAJ | 0 | 4.823 | 0.03785265 | 0.150316267 |
| A0A816WHH5 | 5-Methyltetrahydropteroyltriglutamate--homocysteine S-methyltransferase OS=Brassica napus OX=3708 GN=DARMORV10_A03P33420.1 PE=3 SV=1 | 0 | 88.505 | 32 | 22 | 412 | 6 | 765 | 84.9 | 6.61 | 6356 | 22 | DARMORV10_A03P33420.1 | 1 | 8.873 | 0.004669121 | 0.023365231 |
| A0A816XUS5 | Myrosinase 2-like (Fragment) OS=Brassica napus OX=3708 GN=DARMORV10_A01P22620.1 PE=3 SV=1 | 0 | 2.127 | 2 | 1 | 32 | 1 | 542 | 62.3 | 8.13 | 501 | 1 | DARMORV10_A01P22620.1 | 0 | 11.022 | 0.001600033 | 0.008558539 |
| A0A816YN54 | Non-specific lipid transfer protein GPI-anchored 1, (rape) hypothetical protein OS=Brassica napus OX=3708 GN=DARMORV10_A07P11530.1 PE=3 SV=1 | 0 | 3.006 | 8 | 2 | 14 | 2 | 196 | 20.1 | 7.62 | 74 | 2 | DARMORV10_A07P11530.1 | 0 | 11.585 | 0.002571335 | 0.013438676 |
| A0A816ZKS5 | Uncharacterized BNAA07G30660D, (rape) hypothetical protein OS=Brassica napus OX=3708 GN=DARMORV10_A07P39160.1 PE=4 SV=1 | 0.006 | 1.124 | 2 | 1 | 1 | 1 | 314 | 36 | 4.86 | 46 | 1 | DARMORV10_A07P39160.1 | 0 | 10.723 | 0.002855396 | 0.014796961 |
| A0A816ZXB2 | (rape) hypothetical protein OS=Brassica napus OX=3708 GN=DARMORV10_A08P04600.1 PE=4 SV=1 | 0.003 | 1.302 | 9 | 1 | 6 | 1 | 103 | 12.3 | 10.52 | 46 | 1 | DARMORV10_A08P04600.1 | 0 | 10.431 | 0.001941612 | 0.010257609 |
| A0A817AFS0 | Heat shock 70 kDa protein 15 (rape) hypothetical protein OS=Brassica napus OX=3708 GN=DARMORV10_A04P04090.1 PE=3 SV=1 | 0 | 26.51 | 10 | 7 | 90 | 1 | 814 | 90 | 5.29 | 2027 | 7 | DARMORV10_A04P04090.1 | 0 | 7.298 | 0.00758379 | 0.036575614 |
| A0A817AJF9 | Late embryogenesis abundant protein At3g53040-like, (rape) hypothetical protein OS=Brassica napus OX=3708 GN=DARMORV10_A04P06430.1 PE=4 SV=1 | 0 | 4.638 | 6 | 3 | 26 | 3 | 451 | 48.5 | 5.26 | 205 | 3 | DARMORV10_A04P06430.1 | 0 | 17.648 | 0.005914197 | 0.029066378 |
| A0A817AJH6 | 14-3-3-like protein GF14 psi, (rape) hypothetical protein OS=Brassica napus OX=3708 GN=DARMORV10_A04P11450.1 PE=3 SV=1 | 0 | 51.982 | 43 | 10 | 223 | 3 | 257 | 28.9 | 4.77 | 3025 | 10 | DARMORV10_A04P11450.1 | 0 | 8.617 | 0.006385961 | 0.031243179 |
| A0A817BD13 | Peptidyl-prolyl cis-trans isomerase (Fragment) OS=Brassica napus OX=3708 GN=DARMORV10_A10P15550.1 PE=3 SV=1 | 0 | 2.123 | 5 | 1 | 6 | 1 | 233 | 25.2 | 8.82 | 94 | 1 | DARMORV10_A10P15550.1 | 0 | 9.121 | 0.004458842 | 0.02243238 |
| A0ABQ7X2K8 | Uncharacterized protein (Fragment) OS=Brassica napus OX=3708 GN=HID58_095721 PE=4 SV=1 | 0 | 8.22 | 11 | 3 | 32 | 2 | 351 | 39 | 8 | 469 | 3 | HID58_095721 | 0 | 9.479 | 0.004450264 | 0.022421879 |
| A0ABQ7X484 | Probable ferredoxin-NADP(+) reductase, Uncharacterized protein OS=Brassica napus OX=3708 GN=HID58_092470 PE=4 SV=1 | 0 | 26.57 | 8 | 6 | 53 | 4 | 989 | 109.7 | 7.69 | 899 | 6 | HID58_092470 | 0 | 6.845 | 0.011620574 | 0.054096385 |
| A0ABQ7XBQ1 | Probable plasma membrane-associated cation-binding protein 1, Uncharacterized protein OS=Brassica napus OX=3708 GN=HID58_093241 PE=4 SV=1 | 0 | 7.207 | 12 | 3 | 30 | 1 | 232 | 25.3 | 5.07 | 781 | 3 | HID58_093241 | 0 | 15.956 | 0.001137111 | 0.006159253 |
| A0ABQ7XCG7 | Probable photosystem II 10 kDa polypeptide, chloroplastic, Uncharacterized protein OS=Brassica napus OX=3708 GN=HID58_093684 PE=4 SV=1 | 0.001 | 1.41 | 5 | 1 | 19 | 1 | 147 | 15.7 | 9.55 | 247 | 1 | HID58_093684 | 0 | 18.582 | 0.000191401 | 0.001060324 |
| A0ABQ7XJ37 | Uncharacterized protein OS=Brassica napus OX=3708 GN=HID58_024504 PE=4 SV=1 | 0 | 6.784 | 3 | 2 | 12 | 2 | 576 | 66.4 | 9.31 | 263 | 2 | HID58_024504 | 0 | 7.535 | 0.022064637 | 0.095563023 |
| A0ABQ7XPQ2 | Uncharacterized protein OS=Brassica napus OX=3708 GN=HID58_085166 PE=4 SV=1 | 0 | 2.161 | 7 | 2 | 7 | 1 | 284 | 30.9 | 6.84 | 39 | 2 | HID58_085166 | 0 | 6.56 | 0.012287826 | 0.057066222 |
| A0ABQ7XWC2 | Uncharacterized protein OS=Brassica napus OX=3708 GN=HID58_087622 PE=4 SV=1 | 0 | 12.03 | 5 | 2 | 21 | 2 | 807 | 89.4 | 8.68 | 343 | 2 | HID58_087622 | 0 | 4.839 | 0.035743367 | 0.143428626 |
| A0ABQ7XX08 | Probable aspartate aminotransferase, Uncharacterized protein OS=Brassica napus OX=3708 GN=HID58_088682 PE=4 SV=1 | 0 | 3.923 | 5 | 2 | 55 | 1 | 429 | 46.9 | 7.12 | 879 | 2 | HID58_088682 | 0 | 8.123 | 0.031412658 | 0.128505237 |
| A0ABQ7XXW7 | Uncharacterized protein (Fragment) OS=Brassica napus OX=3708 GN=HID58_088555 PE=4 SV=1 | 0 | 30.56 | 4 | 7 | 165 | 1 | 1797 | 201.3 | 8.98 | 2634 | 7 | HID58_088555 | 0 | 7.172 | 0.007283118 | 0.035160803 |
| A0ABQ7Y453 | Uncharacterized protein (Fragment) OS=Brassica napus OX=3708 GN=HID58_080171 PE=4 SV=1 | 0.004 | 1.22 | 8 | 1 | 5 | 1 | 104 | 12.2 | 5.27 | 58 | 1 | HID58_080171 | 0 | 4.704 | 0.035461312 | 0.142885482 |
| A0ABQ7Y9V6 | Uncharacterized protein OS=Brassica napus OX=3708 GN=HID58_082204 PE=4 SV=1 | 0 | 1.763 | 2 | 1 | 7 | 1 | 581 | 63.8 | 8.88 | 0 | 1 | HID58_082204 | 0 | 4.123 | 0.049988231 | 0.188582363 |
| A0ABQ7YHK3 | Uncharacterized protein OS=Brassica napus OX=3708 GN=HID58_074716 PE=4 SV=1 | 0 | 75.67 | 26 | 17 | 248 | 1 | 1004 | 109.6 | 7.27 | 2432 | 17 | HID58_074716 | 0 | 8.971 | 0.004655229 | 0.0233284 |
| A0ABQ7YPL7 | Probable hemimethylated DNA-binding domain-containing protein, Uncharacterized protein OS=Brassica napus OX=3708 GN=HID58_076599 PE=4 SV=1 | 0 | 1.958 | 3 | 1 | 4 | 1 | 344 | 39.2 | 5.54 | 28 | 1 | HID58_076599 | 0 | 8.692 | 0.00509536 | 0.025350214 |
| A0ABQ7YRS2 | Uncharacterized protein OS=Brassica napus OX=3708 GN=HID58_077940 PE=4 SV=1 | 0 | 4.886 | 5 | 4 | 20 | 2 | 705 | 79.9 | 9.58 | 132 | 4 | HID58_077940 | 0 | 5.305 | 0.030243925 | 0.124222126 |
| A0ABQ7YW91 | Probable glutamine synthetase, Uncharacterized protein OS=Brassica napus OX=3708 GN=HID58_069571 PE=4 SV=1 | 0 | 3.486 | 6 | 2 | 19 | 1 | 360 | 39.6 | 6.87 | 122 | 2 | HID58_069571 | 0 | 14.521 | 0.001155998 | 0.006255236 |
| A0ABQ7YYN6 | Uncharacterized protein OS=Brassica napus OX=3708 GN=HID58_070400 PE=4 SV=1 | 0 | 4.482 | 8 | 3 | 15 | 1 | 261 | 30.3 | 6.8 | 119 | 3 | HID58_070400 | 0 | 17.732 | 0.021549967 | 0.094104891 |
| A0ABQ7YZ18 | Probable TSK-associating protein 1-like, Uncharacterized protein OS=Brassica napus OX=3708 GN=HID58_070541 PE=4 SV=1 | 0 | 7.943 | 8 | 4 | 64 | 2 | 722 | 80.8 | 4.64 | 144 | 4 | HID58_070541 | 0 | 5.482 | 0.038855896 | 0.152916097 |
| A0ABQ7Z624 | Probable vacuolar protein sorting-associated protein 52 A, Uncharacterized protein OS=Brassica napus OX=3708 GN=HID58_073017 PE=4 SV=1 | 0 | 2.277 | 2 | 1 | 12 | 1 | 725 | 82.4 | 5.52 | 61 | 1 | HID58_073017 | 0 | 4.93 | 0.041493114 | 0.160547932 |
| A0ABQ7Z8E8 | Uncharacterized protein OS=Brassica napus OX=3708 GN=HID58_063886 PE=4 SV=1 | 0.004 | 1.221 | 2 | 1 | 3 | 1 | 439 | 48.8 | 6.9 | 27 | 1 | HID58_063886 | 0 | 30.042 | 0.002642538 | 0.013743764 |
| A0ABQ7Z8Y3 | Probable single-stranded DNA binding protein Ssb-like OB fold domain-containing protein, Uncharacterized protein (Fragment) OS=Brassica napus OX=3708 GN=HID58_063926 PE=4 SV=1 | 0.006 | 1.112 | 6 | 1 | 3 | 1 | 145 | 16.3 | 9.1 | 42 | 1 | HID58_063926 | 0 | 13.08 | 0.042643914 | 0.164200142 |
| A0ABQ7Z9W3 | Probable elongation factor 1-gamma 2, Uncharacterized protein (Fragment) OS=Brassica napus OX=3708 GN=HID58_064414 PE=4 SV=1 | 0 | 51.2 | 26 | 8 | 134 | 2 | 435 | 48.8 | 5.5 | 1350 | 8 | HID58_064414 | 0 | 4.729 | 0.037188942 | 0.148312795 |
| A0ABQ7ZE94 | Probable RuBisCO large subunit-binding protein subunit beta, chloroplastic, Uncharacterized protein OS=Brassica napus OX=3708 GN=HID58_065929 PE=4 SV=1 | 0 | 13.157 | 5 | 4 | 75 | 1 | 1023 | 114.6 | 5.49 | 960 | 4 | HID58_065929 | 0 | 5.586 | 0.026888164 | 0.11246527 |
| A0ABQ7ZG33 | Uncharacterized protein OS=Brassica napus OX=3708 GN=HID58_066592 PE=4 SV=1 | 0 | 8.824 | 11 | 3 | 61 | 2 | 351 | 38.8 | 9.35 | 497 | 3 | HID58_066592 | 0 | 8.684 | 0.004963911 | 0.024736441 |
| A0ABQ7ZMD4 | Uncharacterized protein OS=Brassica napus OX=3708 GN=HID58_068752 PE=4 SV=1 | 0.004 | 1.243 | 2 | 1 | 1 | 1 | 363 | 40.7 | 5.21 | 0 | 1 | HID58_068752 | 0 | 6.633 | 0.012664802 | 0.058715141 |
| A0ABQ7ZYL1 | Probable eukaryotic translation initiation factor 4C, Uncharacterized protein OS=Brassica napus OX=3708 GN=HID58_061446 PE=4 SV=1 | 0 | 2.592 | 6 | 1 | 2 | 1 | 178 | 20 | 5.17 | 49 | 1 | HID58_061446 | 0 | 100 | 1E-17 | 5.5977E-17 |
| A0ABQ8A2S7 | Uncharacterized protein OS=Brassica napus OX=3708 GN=HID58_062910 PE=4 SV=1 | 0.005 | 1.172 | 2 | 1 | 4 | 1 | 451 | 50 | 8.38 | 0 | 1 | HID58_062910 | 0 | 5.761 | 0.033252668 | 0.13495041 |
| A0ABQ8A2Z8 | Uncharacterized protein OS=Brassica napus OX=3708 GN=HID58_063002 PE=4 SV=1 | 0 | 28.45 | 18 | 9 | 86 | 4 | 460 | 50.5 | 10.42 | 1139 | 9 | HID58_063002 | 4 | 4.358 | 0.032690697 | 0.132946717 |
| A0ABQ8A862 | Uncharacterized protein OS=Brassica napus OX=3708 GN=HID58_050857 PE=4 SV=1 | 0.004 | 1.239 | 5 | 1 | 4 | 1 | 198 | 22.5 | 6.4 | 26 | 1 | HID58_050857 | 0 | 11.353 | 0.001839233 | 0.00975039 |
| A0ABQ8A9J9 | Probable RRM domain-containing protein, Uncharacterized protein OS=Brassica napus OX=3708 GN=HID58_051625 PE=4 SV=1 | 0.004 | 1.187 | 2 | 1 | 5 | 1 | 628 | 71.9 | 5.92 | 0 | 1 | HID58_051625 | 0 | 10.343 | 0.013324978 | 0.061471059 |
| A0ABQ8AI34 | Probable peptidylprolyl isomerase, Uncharacterized protein OS=Brassica napus OX=3708 GN=HID58_054381 PE=4 SV=1 | 0 | 3.223 | 14 | 2 | 14 | 2 | 160 | 16.9 | 9.38 | 171 | 2 | HID58_054381 | 0 | 4.979 | 0.034004845 | 0.137378548 |
| A0ABQ8B5W1 | Uncharacterized protein OS=Brassica napus OX=3708 GN=HID58_049733 PE=4 SV=1 | 0.006 | 1.16 | 2 | 1 | 1 | 1 | 593 | 66.1 | 5.2 | 0 | 1 | HID58_049733 | 0 | 6.652 | 0.038216148 | 0.151395014 |
| A0ABQ8BA75 | Probable SRP54-type proteins GTP-binding domain-containing protein, Uncharacterized protein OS=Brassica napus OX=3708 GN=HID58_040683 PE=4 SV=1 | 0.002 | 1.333 | 1 | 1 | 2 | 1 | 632 | 69.8 | 7.91 | 22 | 1 | HID58_040683 | 0 | 19.917 | 0.00037442 | 0.002056142 |
| A0ABQ8BBJ8 | Uncharacterized protein OS=Brassica napus OX=3708 GN=HID58_041698 PE=4 SV=1 | 0 | 13.831 | 5 | 6 | 45 | 6 | 1781 | 197.3 | 8.59 | 770 | 6 | HID58_041698 | 0 | 4.91 | 0.03001663 | 0.123430501 |
| A0ABQ8BK95 | Probable tyrosine-tRNA ligase, Uncharacterized protein OS=Brassica napus OX=3708 GN=HID58_044743 PE=4 SV=1 | 0 | 4.874 | 7 | 3 | 10 | 3 | 503 | 55.8 | 6.95 | 49 | 3 | HID58_044743 | 0 | 23.332 | 0.013919774 | 0.063815344 |
| A0ABQ8BKU7 | Uncharacterized protein OS=Brassica napus OX=3708 GN=HID58_037284 PE=4 SV=1 | 0.001 | 1.377 | 1 | 1 | 13 | 1 | 930 | 106.6 | 8.9 | 117 | 1 | HID58_037284 | 0 | 100 | 1E-17 | 5.5977E-17 |
| A0ABQ8BNC0 | Probable RanBD1 domain-containing protein, Uncharacterized protein OS=Brassica napus OX=3708 GN=HID58_037655 PE=4 SV=1 | 0 | 1.646 | 4 | 1 | 4 | 1 | 283 | 31.9 | 5.17 | 43 | 1 | HID58_037655 | 0 | 11.616 | 0.001845646 | 0.009774718 |
| A0ABQ8BP75 | Probable PP2A regulatory subunit TAP46, Uncharacterized protein (Fragment) OS=Brassica napus OX=3708 GN=HID58_038149 PE=4 SV=1 | 0.004 | 1.207 | 2 | 1 | 3 | 1 | 423 | 47.9 | 5.21 | 0 | 1 | HID58_038149 | 0 | 7.505 | 0.040972037 | 0.159106346 |
| A0ABQ8BVW9 | Probable Ras-related protein Rab11D, Uncharacterized protein (Fragment) OS=Brassica napus OX=3708 GN=HID58_032276 PE=4 SV=1 | 0 | 3.949 | 15 | 3 | 15 | 2 | 229 | 25.1 | 6.24 | 162 | 3 | HID58_032276 | 0 | 5.066 | 0.038358591 | 0.151706883 |
| A0ABQ8BVX5 | Uncharacterized protein (Fragment) OS=Brassica napus OX=3708 GN=HID58_032234 PE=4 SV=1 | 0 | 1.742 | 7 | 1 | 5 | 1 | 177 | 20.1 | 5.39 | 97 | 1 | HID58_032234 | 0 | 12.194 | 0.033641157 | 0.136165984 |
| A0ABQ8C4D6 | Uncharacterized protein OS=Brassica napus OX=3708 GN=HID58_035227 PE=4 SV=1 | 0 | 3.536 | 8 | 2 | 12 | 1 | 558 | 61.2 | 8.32 | 112 | 2 | HID58_035227 | 1 | 8.71 | 0.038034553 | 0.150731347 |
| A0ABQ8C4M1 | Uncharacterized protein (Fragment) OS=Brassica napus OX=3708 GN=HID58_035317 PE=4 SV=1 | 0 | 3.259 | 2 | 1 | 49 | 1 | 410 | 43.2 | 6.27 | 589 | 1 | HID58_035317 | 0 | 9.606 | 0.008623667 | 0.041100518 |
| A0ABQ8C7G1 | Uncharacterized protein OS=Brassica napus OX=3708 GN=HID58_036330 PE=4 SV=1 | 0.007 | 1.105 | 1 | 1 | 1 | 1 | 776 | 87.6 | 6.3 | 0 | 1 | HID58_036330 | 0 | 9.364 | 0.006017785 | 0.029555144 |
| A0ABQ8C958 | Uncharacterized protein OS=Brassica napus OX=3708 GN=HID58_036927 PE=4 SV=1 | 0 | 1.959 | 1 | 1 | 6 | 1 | 852 | 81.3 | 5.52 | 83 | 1 | HID58_036927 | 0 | 12.83 | 0.001085604 | 0.005884726 |
| A0ABQ8CDP1 | Probable STI1 domain-containing protein, Uncharacterized protein OS=Brassica napus OX=3708 GN=HID58_029640 PE=4 SV=1 | 0 | 25.498 | 15 | 5 | 64 | 3 | 469 | 49.7 | 5.02 | 1935 | 5 | HID58_029640 | 2 | 5.328 | 0.030667866 | 0.125722407 |
| A0ABQ8CGH1 | Probable carboxypeptidase, Uncharacterized protein OS=Brassica napus OX=3708 GN=HID58_029906 PE=4 SV=1 | 0 | 9.785 | 8 | 4 | 21 | 3 | 678 | 75.5 | 6.1 | 65 | 4 | HID58_029906 | 1 | 7.68 | 0.008685428 | 0.04134889 |
| A0ABQ8CGP7 | Uncharacterized protein OS=Brassica napus OX=3708 GN=HID58_030700 PE=4 SV=1 | 0 | 18.063 | 8 | 4 | 43 | 4 | 696 | 78.8 | 8.9 | 672 | 4 | HID58_030700 | 0 | 3.999 | 0.040827025 | 0.158888757 |
| A0ABQ8CHS6 | Uncharacterized protein (Fragment) OS=Brassica napus OX=3708 GN=HID58_031080 PE=4 SV=1 | 0 | 3.234 | 2 | 2 | 11 | 2 | 1029 | 115.5 | 9.58 | 139 | 2 | HID58_031080 | 0 | 4.621 | 0.038956924 | 0.152916097 |
| A0ABQ8CJ07 | Uncharacterized protein OS=Brassica napus OX=3708 GN=HID58_030984 PE=4 SV=1 | 0.006 | 1.113 | 2 | 1 | 3 | 1 | 899 | 99.8 | 6.95 | 0 | 1 | HID58_030984 | 0 | 10.648 | 0.002365269 | 0.012391927 |
| A0ABQ8CP86 | Probable 14-3-3 domain-containing protein, Uncharacterized protein OS=Brassica napus OX=3708 GN=HID58_025850 PE=4 SV=1 | 0 | 17.679 | 13 | 5 | 133 | 1 | 304 | 34.2 | 4.87 | 818 | 5 | HID58_025850 | 0 | 13.459 | 0.001018725 | 0.005537606 |
| A0ABQ8CPU6 | Probable glucose-6-phosphate 1-epimerase, Uncharacterized protein OS=Brassica napus OX=3708 GN=HID58_026060 PE=4 SV=1 | 0.001 | 1.427 | 3 | 1 | 2 | 1 | 308 | 33.9 | 6.64 | 52 | 1 | HID58_026060 | 0 | 8.996 | 0.003797247 | 0.019368581 |
| A0ABQ8D9A6 | Uncharacterized protein OS=Brassica napus OX=3708 GN=HID58_018225 PE=4 SV=1 | 0 | 11.644 | 13 | 5 | 52 | 2 | 431 | 46.7 | 8.12 | 781 | 5 | HID58_018225 | 2 | 5.156 | 0.032242486 | 0.131223549 |
| A0ABQ8DIX1 | Uncharacterized protein OS=Brassica napus OX=3708 GN=HID58_014990 PE=4 SV=1 | 0 | 3.019 | 5 | 2 | 12 | 2 | 357 | 39.8 | 4.88 | 120 | 2 | HID58_014990 | 0 | 9.458 | 0.005583329 | 0.027592153 |
| A0ABQ8DQV7 | Probable NAD(P)H dehydrogenase (quinone), Uncharacterized protein OS=Brassica napus OX=3708 GN=HID58_008869 PE=4 SV=1 | 0 | 3.536 | 5 | 1 | 7 | 1 | 243 | 26.1 | 5.44 | 127 | 1 | HID58_008869 | 0 | 100 | 1E-17 | 5.5977E-17 |
| A0ABQ8E309 | Uncharacterized protein OS=Brassica napus OX=3708 GN=HID58_013121 PE=4 SV=1 | 0.006 | 1.123 | 6 | 1 | 1 | 1 | 178 | 19.7 | 4.96 | 0 | 1 | HID58_013121 | 0 | 11.661 | 0.015664006 | 0.070946589 |
| A0ABQ8E497 | Probable TPX2 C-terminal domain-containing protein, Uncharacterized protein OS=Brassica napus OX=3708 GN=HID58_013572 PE=4 SV=1 | 0 | 2.143 | 2 | 1 | 17 | 1 | 329 | 37.2 | 10.11 | 309 | 1 | HID58_013572 | 0 | 10.957 | 0.001668601 | 0.008903083 |
| A0ABQ8E8Q4 | Uncharacterized protein OS=Brassica napus OX=3708 GN=HID58_004476 PE=4 SV=1 | 0 | 3.155 | 2 | 2 | 3 | 1 | 1213 | 135.6 | 7.77 | 37 | 2 | HID58_004476 | 0 | 7.755 | 0.043485234 | 0.167139301 |
| A0ABQ8EGF8 | Probable peptidyl-prolyl cis-trans isomerase, Uncharacterized protein OS=Brassica napus OX=3708 GN=HID58_000332 PE=4 SV=1 | 0 | 7.966 | 13 | 3 | 38 | 2 | 197 | 20.8 | 9.03 | 405 | 3 | HID58_000332 | 1 | 7.915 | 0.005239447 | 0.026036845 |
| A0ABQ8EHX7 | Probable 26S proteasome non-ATPase regulatory subunit 2 homolog, Uncharacterized protein OS=Brassica napus OX=3708 GN=HID58_000823 PE=4 SV=1 | 0 | 40.964 | 20 | 12 | 120 | 1 | 871 | 95.4 | 5.15 | 1168 | 12 | HID58_000823 | 0 | 4.479 | 0.038296678 | 0.151569017 |
| A0ABQ8EII9 | Probable plasma-membrane associated cation-binding protein 1, Uncharacterized protein OS=Brassica napus OX=3708 GN=HID58_001076 PE=4 SV=1 | 0 | 7.65 | 12 | 3 | 25 | 1 | 249 | 27.3 | 4.86 | 766 | 3 | HID58_001076 | 2 | 5.882 | 0.017829293 | 0.080059952 |
| A0ABQ8EPH6 | Uncharacterized protein (Fragment) OS=Brassica napus OX=3708 GN=HID58_003224 PE=4 SV=1 | 0 | 6.034 | 1 | 2 | 77 | 2 | 1237 | 134 | 6.47 | 757 | 2 | HID58_003224 | 0 | 13.917 | 0.001056517 | 0.005732855 |
| A0ABQ8EPM1 | Probable TSA1-like protein, Uncharacterized protein OS=Brassica napus OX=3708 GN=HID58_003259 PE=4 SV=1 | 0 | 32.358 | 15 | 12 | 178 | 9 | 774 | 85.4 | 4.84 | 993 | 12 | HID58_003259 | 5 | 8.216 | 0.00665815 | 0.032289339 |
| F8K8R8 | Cytochrome b OS=Brassica napus OX=3708 GN=cob PE=3 SV=1 | 0.009 | 1.043 | 2 | 1 | 2 | 1 | 393 | 44.1 | 7.27 | 38 | 1 | cob | 0 | 4.378 | 0.033587925 | 0.136130521 |
| O04852 | Glutamine synthetase OS=Brassica napus OX=3708 GN=BnaCnng23850D PE=3 SV=1 | 0 | 26.919 | 26 | 6 | 101 | 3 | 356 | 39.3 | 5.48 | 870 | 6 | gln; GS1_1 | 4 | 5.077 | 0.021229267 | 0.092742248 |
| Q43744 | Malate dehydrogenase, mitochondrial OS=Brassica napus OX=3708 GN=MDH PE=2 SV=1 | 0 | 37.701 | 30 | 8 | 140 | 2 | 341 | 35.7 | 8.68 | 1901 | 8 | MDH | 0 | 9.715 | 0.005086225 | 0.025316524 |
| A0A068FAY3 | LL-diaminopimelate aminotransferase, (rape) hypothetical protein OS=Brassica napus OX=3708 GN=DARMORV10_A01P04640.1 PE=3 SV=1 | 0 | 14.27 | 15 | 5 | 72 | 2 | 458 | 50.1 | 6.77 | 1005 | 5 | DARMORV10_A01P04640.1 | 0 | 0.01 | 1E-17 | 5.5977E-17 |
| A0A076VK12 | DNA-directed RNA polymerase subunit beta OS=Brassica napus OX=3708 GN=rpoB PE=3 SV=1 | 0 | 7.661 | 6 | 5 | 15 | 5 | 1072 | 121 | 8.5 | 64 | 5 | rpoB | 0 | 0.01 | 1E-17 | 5.5977E-17 |
| A0A078E3L6 | Trafficking protein particle complex subunit OS=Brassica napus OX=3708 GN=BnaA09g15790D PE=3 SV=1 | 0 | 7.796 | 20 | 3 | 18 | 3 | 186 | 20.7 | 4.46 | 301 | 3 | BnaA09g15790D; LOC108823959 | 0 | 0.01 | 1E-17 | 5.5977E-17 |
| A0A078F013 | Ethanolamine-phosphate cytidylyltransferase OS=Brassica napus OX=3708 GN=BnaA03g18010D PE=3 SV=1 | 0 | 2.59 | 3 | 1 | 6 | 1 | 439 | 48.8 | 7.94 | 26 | 1 | BnaA03g18010D | 0 | 0.01 | 1E-17 | 5.5977E-17 |
| A0A078F117 | (rape) hypothetical protein OS=Brassica napus OX=3708 GN=BnaA06g15610D PE=4 SV=1 | 0.003 | 1.254 | 6 | 1 | 3 | 1 | 291 | 31.6 | 9.07 | 0 | 1 | BnaA06g15610D | 0 | 0.01 | 1E-17 | 5.5977E-17 |
| A0A078F1R5 | Defective in cullin neddylation protein OS=Brassica napus OX=3708 GN=BnaA05g26410D PE=4 SV=1 | 0 | 3.903 | 10 | 2 | 13 | 2 | 239 | 27.4 | 5.01 | 92 | 2 | BnaA05g26410D | 0 | 0.01 | 1E-17 | 5.5977E-17 |
| A0A078F366 | Procollagen-proline 4-dioxygenase OS=Brassica napus OX=3708 GN=BnaC03g47960D PE=3 SV=1 | 0 | 2.53 | 3 | 1 | 7 | 1 | 293 | 32.9 | 8.22 | 29 | 1 | BnaC03g47960D | 0 | 0.01 | 1E-17 | 5.5977E-17 |
| A0A078F5A7 | protein MET1, chloroplastic, (rape) hypothetical protein OS=Brassica napus OX=3708 GN=BnaA05g13750D PE=4 SV=1 | 0 | 23.785 | 29 | 7 | 51 | 7 | 329 | 37.1 | 7.15 | 672 | 7 | BnaA05g13750D | 0 | 0.07 | 0.020104521 | 0.088769768 |
| A0A078F5T2 | Leucine aminopeptidase OS=Brassica napus OX=3708 GN=BnaC09g43560D PE=3 SV=1 | 0.002 | 1.351 | 2 | 1 | 8 | 1 | 614 | 68.8 | 5.31 | 42 | 1 | BnaC09g43560D | 0 | 0.01 | 1E-17 | 5.5977E-17 |
| A0A078F604 | universal stress protein YxiE, BnaA05g27200D protein OS=Brassica napus OX=3708 GN=BnaA05g27200D PE=4 SV=1 | 0 | 2.489 | 10 | 1 | 3 | 1 | 200 | 21.8 | 6.2 | 22 | 1 | BnaA05g27200D | 0 | 0.01 | 1E-17 | 5.5977E-17 |
| A0A078F6W4 | Phospholipase C, (rape) hypothetical protein OS=Brassica napus OX=3708 GN=BnaC05g05030D PE=3 SV=1 | 0 | 2.224 | 2 | 1 | 15 | 1 | 529 | 59.5 | 6.81 | 111 | 1 | BnaC05g05030D | 0 | 0.01 | 1E-17 | 5.5977E-17 |
| A0A078F736 | Starch synthase OS=Brassica napus OX=3708 GN=BnaC08g11320D PE=3 SV=1 | 0 | 2.593 | 1 | 1 | 9 | 1 | 1020 | 115.7 | 5.69 | 89 | 1 | BnaC08g11320D | 0 | 0.01 | 1E-17 | 5.5977E-17 |
| A0A078F790 | ras-related protein RABC2a, BnaCnng02760D protein OS=Brassica napus OX=3708 GN=BnaCnng02760D PE=3 SV=1 | 0 | 4.458 | 11 | 2 | 16 | 1 | 210 | 23.3 | 7.01 | 57 | 2 | BnaCnng02760D | 0 | 0.01 | 1E-17 | 5.5977E-17 |
| A0A078FA30 | Probable [Acyl-CoA-binding domain-containing protein](https://www.uniprot.org/uniprotkb/A0A8X7P3R1/entry), (rape) hypothetical protein OS=Brassica napus OX=3708 GN=BnaA03g01260D PE=4 SV=1 | 0 | 2.051 | 2 | 1 | 2 | 1 | 503 | 55.1 | 5.96 | 16 | 1 | BnaA03g01260D | 0 | 0.01 | 1E-17 | 5.5977E-17 |
| A0A078FA32 | protein TIC110, chloroplastic, BnaC05g04790D protein OS=Brassica napus OX=3708 GN=BnaC05g04790D PE=4 SV=1 | 0 | 20.569 | 12 | 9 | 62 | 1 | 1005 | 111 | 5.86 | 483 | 9 | BnaC05g04790D | 0 | 0.01 | 1E-17 | 5.5977E-17 |
| A0A078FAF5 | protein SEMI-ROLLED LEAF 2, (rape) hypothetical protein OS=Brassica napus OX=3708 GN=BnaC08g44520D PE=4 SV=1 | 0.007 | 1.094 | 2 | 1 | 1 | 1 | 942 | 104.9 | 5.63 | 0 | 1 | BnaC08g44520D | 0 | 0.046 | 0.004081298 | 0.020763069 |
| A0A078FAL2 | Peroxidase OS=Brassica napus OX=3708 GN=BnaC08g44140D PE=3 SV=1 | 0 | 6.671 | 9 | 2 | 12 | 2 | 326 | 35.1 | 8.29 | 126 | 2 | BnaC08g44140D | 0 | 0.01 | 1E-17 | 5.5977E-17 |
| A0A078FBB6 | **Probable Nucleoplasmin-like domain-containing protein,** BnaA02g05840D protein OS=Brassica napus OX=3708 GN=BnaA02g05840D PE=3 SV=1 | 0 | 2.056 | 3 | 1 | 2 | 1 | 348 | 36.9 | 4.87 | 32 | 1 | BnaA02g05840D | 0 | 0.01 | 1E-17 | 5.5977E-17 |
| A0A078FBT8 | Trafficking protein particle complex subunit OS=Brassica napus OX=3708 GN=BnaCnng03550D PE=3 SV=1 | 0 | 4.023 | 16 | 2 | 7 | 2 | 141 | 16 | 5.76 | 26 | 2 | BnaCnng03550D | 0 | 0.01 | 1E-17 | 5.5977E-17 |
| A0A078FDV7 | Large ribosomal subunit protein uL4c OS=Brassica napus OX=3708 GN=BnaC05g05110D PE=3 SV=1 | 0 | 5.258 | 5 | 1 | 39 | 1 | 288 | 31.2 | 9.19 | 474 | 1 | BnaC05g05110D | 0 | 0.077 | 0.021075235 | 0.092295145 |
| A0A078FDW8 | protein TIC 56, chloroplastic, (rape) hypothetical protein OS=Brassica napus OX=3708 GN=BnaA03g00280D PE=4 SV=1 | 0 | 3.655 | 2 | 1 | 14 | 1 | 519 | 61 | 6.98 | 147 | 1 | BnaA03g00280D | 0 | 0.01 | 1E-17 | 5.5977E-17 |
| A0A078FEV0 | (rape) hypothetical protein OS=Brassica napus OX=3708 GN=BnaA09g40450D PE=3 SV=1 | 0 | 3.358 | 17 | 1 | 2 | 1 | 123 | 12.8 | 9.29 | 98 | 1 | BnaA09g40450D | 0 | 0.01 | 1E-17 | 5.5977E-17 |
| A0A078FF11 | nucleolin-like, (rape) hypothetical protein OS=Brassica napus OX=3708 GN=BnaA09g40800D PE=4 SV=1 | 0 | 2.21 | 6 | 1 | 2 | 1 | 304 | 33.4 | 5.22 | 48 | 1 | BnaA09g40800D | 0 | 0.01 | 1E-17 | 5.5977E-17 |
| A0A078FGS0 | Monodehydroascorbate reductase (NADH) OS=Brassica napus OX=3708 GN=BnaA03g00990D PE=3 SV=1 | 0 | 5.268 | 10 | 3 | 11 | 2 | 435 | 47 | 5.17 | 214 | 3 | BnaA03g00990D | 0 | 0.01 | 1E-17 | 5.5977E-17 |
| A0A078FGZ3 | histidine biosynthesis bifunctional protein hisIE, chloroplastic, BnaA09g24740D protein OS=Brassica napus OX=3708 GN=BnaA09g24740D PE=3 SV=1 | 0 | 2.418 | 5 | 1 | 11 | 1 | 282 | 31.3 | 6.15 | 75 | 1 | BnaA09g24740D | 0 | 0.01 | 1E-17 | 5.5977E-17 |
| A0A078FI85 | cold shock protein 2 , (rape) hypothetical protein OS=Brassica napus OX=3708 GN=BnaC03g57650D PE=3 SV=1 | 0 | 4.931 | 12 | 1 | 8 | 1 | 177 | 17.3 | 6.76 | 117 | 1 | BnaC03g57650D | 0 | 0.01 | 1E-17 | 5.5977E-17 |
| A0A078FJF5 | Eukaryotic translation initiation factor 3 subunit D OS=Brassica napus OX=3708 GN=BnaA09g17080D PE=3 SV=1 | 0.006 | 1.112 | 3 | 1 | 1 | 1 | 585 | 65.8 | 5.63 | 15 | 1 | BnaA09g17080D | 0 | 0.01 | 1E-17 | 5.5977E-17 |
| A0A078FKB8 | photosynthetic NDH subunit of subcomplex B 5, chloroplastic, BnaA02g22320D protein OS=Brassica napus OX=3708 GN=BnaA02g22320D PE=4 SV=1 | 0 | 1.799 | 5 | 1 | 5 | 1 | 222 | 24.8 | 4.68 | 23 | 1 | BnaA02g22320D | 0 | 0.01 | 1E-17 | 5.5977E-17 |
| A0A078FLI1 | protein THYLAKOID FORMATION 1, chloroplastic, BnaA09g43540D protein OS=Brassica napus OX=3708 GN=BnaA09g43540D PE=3 SV=1 | 0 | 10.962 | 13 | 3 | 25 | 3 | 296 | 33.3 | 9.17 | 137 | 3 | BnaA09g43540D | 0 | 0.018 | 9.82361E-05 | 0.00054647 |
| A0A078FPR3 | Caffeic O-methyltransferase 1-5, BnaA10g07270D protein OS=Brassica napus OX=3708 GN=BnaA10g07270D PE=4 SV=1 | 0 | 6.933 | 6 | 2 | 33 | 2 | 364 | 40 | 5.68 | 182 | 2 | BnaA10g07270D | 0 | 0.087 | 0.028865848 | 0.119524136 |
| A0A078FSK6 | UPF0301 protein CHU_1773, BnaC06g07740D protein OS=Brassica napus OX=3708 GN=BnaC06g07740D PE=4 SV=1 | 0 | 2.463 | 4 | 1 | 4 | 1 | 327 | 36.3 | 7.08 | 45 | 1 | BnaC06g07740D | 0 | 0.01 | 1E-17 | 5.5977E-17 |
| A0A078FTA9 | NADH dehydrogenase [ubiquinone] 1 alpha subcomplex subunit 13 OS=Brassica napus OX=3708 GN=BnaA08g27970D PE=3 SV=1 | 0 | 13.663 | 38 | 4 | 54 | 1 | 143 | 16.1 | 9.36 | 546 | 4 | BnaA08g27970D; LOC108807801 | 0 | 0.077 | 0.025948619 | 0.109767112 |
| A0A078FVZ3 | polyadenylate-binding protein RBP47B-like, (rape) hypothetical protein OS=Brassica napus OX=3708 GN=BnaA01g26250D PE=3 SV=1 | 0 | 1.706 | 3 | 1 | 13 | 1 | 434 | 47.8 | 6.67 | 148 | 1 | BnaA01g26250D | 0 | 0.01 | 1E-17 | 5.5977E-17 |
| A0A078FXS4 | probable peroxygenase 3, BnaA05g10200D protein OS=Brassica napus OX=3708 GN=BnaA05g10200D PE=3 SV=1 | 0 | 2.553 | 5 | 1 | 3 | 1 | 239 | 26.9 | 5.66 | 0 | 1 | BnaA05g10200D | 0 | 0.01 | 1E-17 | 5.5977E-17 |
| A0A078FZJ3 | fasciclin-like arabinogalactan protein 9, BnaC05g02150D protein OS=Brassica napus OX=3708 GN=BnaC05g02150D PE=3 SV=1 | 0.004 | 1.203 | 10 | 1 | 1 | 1 | 250 | 26.4 | 8.1 | 0 | 1 | BnaC05g02150D | 0 | 0.01 | 1E-17 | 5.5977E-17 |
| A0A078G0V8 | MIP/aquaporin (TC 1.A.8) family, (rape) hypothetical protein OS=Brassica napus OX=3708 GN=BnaA01g28120D PE=3 SV=1 | 0 | 8.597 | 21 | 2 | 5 | 1 | 249 | 25 | 5.64 | 136 | 2 | BnaA01g28120D | 1 | 0.01 | 1E-17 | 5.5977E-17 |
| A0A078G1Y5 | Pre-mRNA-processing factor 19 OS=Brassica napus OX=3708 GN=BnaC04g11150D PE=3 SV=1 | 0 | 3.828 | 4 | 1 | 15 | 1 | 609 | 65.9 | 6.95 | 249 | 1 | BnaC04g11150D | 0 | 0.01 | 1E-17 | 5.5977E-17 |
| A0A078G325 | rhodanese-like domain-containing protein 14, chloroplastic, BnaC01g20350D protein OS=Brassica napus OX=3708 GN=BnaC01g20350D PE=4 SV=1 | 0 | 3.796 | 10 | 2 | 18 | 2 | 227 | 25.3 | 7.34 | 64 | 2 | BnaC01g20350D | 0 | 0.01 | 1E-17 | 5.5977E-17 |
| A0A078G3Q8 | ribose-5-phosphate isomerase OS=Brassica napus OX=3708 GN=BnaA01g33410D PE=3 SV=1 | 0 | 39.369 | 35 | 6 | 123 | 2 | 275 | 29.2 | 6.25 | 2394 | 6 | BnaA01g33410D | 0 | 0.04 | 0.002675509 | 0.013911866 |
| A0A078G3X4 | peptide-N4-(N-acetyl-beta-glucosaminyl)asparagine amidase A-like, (rape) hypothetical protein OS=Brassica napus OX=3708 GN=BnaA01g29080D PE=4 SV=1 | 0 | 2.185 | 2 | 1 | 13 | 1 | 597 | 67.5 | 8 | 62 | 1 | BnaA01g29080D | 0 | 0.01 | 1E-17 | 5.5977E-17 |
| A0A078G4J9 | tRNA nucleotidyltransferase/poly(A) polymerase family, BnaA09g30470D protein OS=Brassica napus OX=3708 GN=BnaA09g30470D PE=3 SV=1 | 0 | 1.85 | 2 | 1 | 1 | 1 | 601 | 68.4 | 6.95 | 0 | 1 | BnaA09g30470D | 0 | 0.01 | 1E-17 | 5.5977E-17 |
| A0A078G4L8 | COP9 signalosome complex subunit 5°, BnaA09g30270D protein OS=Brassica napus OX=3708 GN=BnaA09g30270D PE=3 SV=1 | 0 | 4.177 | 7 | 2 | 6 | 2 | 356 | 39.8 | 5.08 | 47 | 2 | BnaA09g30270D | 0 | 0.01 | 1E-17 | 5.5977E-17 |
| A0A078G5U4 | probable sarcosine oxidase, (rape) hypothetical protein OS=Brassica napus OX=3708 GN=BnaC04g36290D PE=3 SV=1 | 0 | 3.426 | 3 | 1 | 5 | 1 | 413 | 45.3 | 6.8 | 22 | 1 | BnaC04g36290D | 0 | 0.01 | 1E-17 | 5.5977E-17 |
| A0A078G6R5 | Probable SAP domain-containing protein**,** BnaA01g33620D protein OS=Brassica napus OX=3708 GN=BnaA01g33620D PE=4 SV=1 | 0 | 2.465 | 1 | 1 | 7 | 1 | 907 | 102.6 | 5 | 42 | 1 | BnaA01g33620D | 0 | 0.01 | 1E-17 | 5.5977E-17 |
| A0A078G6T8 | Histone deacetylase OS=Brassica napus OX=3708 GN=BnaA01g33500D PE=3 SV=1 | 0 | 1.606 | 3 | 1 | 4 | 1 | 502 | 56 | 5.39 | 23 | 1 | BnaA01g33500D | 0 | 0.01 | 1E-17 | 5.5977E-17 |
| A0A078G6U3 | H/ACA ribonucleoprotein complex subunit OS=Brassica napus OX=3708 GN=BnaA01g33870D PE=3 SV=1 | 0 | 4.733 | 6 | 1 | 15 | 1 | 203 | 20.9 | 11.44 | 306 | 1 | BnaA01g33870D | 0 | 0.01 | 1E-17 | 5.5977E-17 |
| A0A078G743 | Nicalin OS=Brassica napus OX=3708 GN=BnaC03g54000D PE=3 SV=1 | 0 | 4.267 | 5 | 2 | 16 | 2 | 565 | 62.2 | 6.55 | 130 | 2 | BnaC03g54000D | 0 | 0.01 | 1E-17 | 5.5977E-17 |
| A0A078G7U2 | Beta-galactosidase OS=Brassica napus OX=3708 GN=BnaA02g08480D PE=3 SV=1 | 0 | 4.963 | 4 | 2 | 15 | 1 | 722 | 80.9 | 8.82 | 23 | 2 | BnaA02g08480D | 0 | 0.01 | 1E-17 | 5.5977E-17 |
| A0A078G8E2 | probable 3-hydroxyisobutyrate dehydrogenase-like 1, mitochondrial, BnaC06g01480D protein OS=Brassica napus OX=3708 GN=BnaC06g01480D PE=4 SV=1 | 0 | 13.325 | 14 | 4 | 40 | 4 | 335 | 35.3 | 8.84 | 229 | 4 | BnaC06g01480D | 0 | 0.075 | 0.019921254 | 0.088033126 |
| A0A078G901 | Protein farnesyltransferase/geranylgeranyltransferase type-1 subunit alpha OS=Brassica napus OX=3708 GN=BnaA01g18430D PE=3 SV=1 | 0.004 | 1.234 | 4 | 1 | 3 | 1 | 325 | 37.5 | 5.39 | 0 | 1 | BnaA01g18430D | 0 | 0.01 | 1E-17 | 5.5977E-17 |
| A0A078G971 | protein WVD2-like 2, (rape) hypothetical protein OS=Brassica napus OX=3708 GN=BnaC03g29720D PE=3 SV=1 | 0.007 | 1.107 | 8 | 1 | 1 | 1 | 309 | 34.5 | 9.76 | 0 | 1 | BnaC03g29720D | 0 | 0.01 | 1E-17 | 5.5977E-17 |
| A0A078G9F7 | 60S ribosomal protein L28-1, BnaA09g44030D protein OS=Brassica napus OX=3708 GN=BnaA09g44030D PE=3 SV=1 | 0 | 4.101 | 20 | 2 | 18 | 2 | 143 | 16 | 10.48 | 276 | 2 | BnaA09g44030D | 0 | 0.01 | 1E-17 | 5.5977E-17 |
| A0A078GAE5 | outer envelope pore protein 37, chloroplastic-like, (rape) hypothetical protein OS=Brassica napus OX=3708 GN=BnaC04g49380D PE=4 SV=1 | 0 | 5.48 | 8 | 2 | 17 | 2 | 343 | 39 | 8.98 | 284 | 2 | BnaC04g49380D | 0 | 0.01 | 1E-17 | 5.5977E-17 |
| A0A078GAH7 | BRISC and BRCA1-A complex member 2 OS=Brassica napus OX=3708 GN=BnaA06g37130D PE=3 SV=1 | 0 | 3.961 | 3 | 1 | 19 | 1 | 378 | 43.2 | 5.4 | 217 | 1 | BnaA06g37130D | 0 | 0.01 | 1E-17 | 5.5977E-17 |
| A0A078GBE9 | Protein transport protein SEC23 OS=Brassica napus OX=3708 GN=BnaA09g00370D PE=3 SV=1 | 0 | 1.76 | 1 | 1 | 5 | 1 | 874 | 95.5 | 6.98 | 31 | 1 | BnaA09g00370D | 0 | 0.01 | 1E-17 | 5.5977E-17 |
| A0A078GC86 | Peptide-methionine (R)-S-oxide reductase OS=Brassica napus OX=3708 GN=BnaA06g00780D PE=3 SV=1 | 0 | 3.014 | 5 | 1 | 9 | 1 | 204 | 22.8 | 8.91 | 70 | 1 | BnaA06g00780D | 0 | 0.01 | 1E-17 | 5.5977E-17 |
| A0A078GCG6 | Small GTPase superfamily. Arf family, BnaC07g31560D protein OS=Brassica napus OX=3708 GN=BnaC07g31560D PE=3 SV=1 | 0 | 7.666 | 20 | 3 | 21 | 3 | 184 | 20.6 | 7.8 | 182 | 3 | BnaC07g31560D; LOC108852083 | 0 | 0.048 | 0.001780682 | 0.009489292 |
| A0A078GCW3 | histone chaperone ASF1-like, (rape) hypothetical protein OS=Brassica napus OX=3708 GN=BnaA07g02500D PE=4 SV=1 | 0.004 | 1.235 | 11 | 1 | 1 | 1 | 149 | 16.8 | 5.05 | 0 | 1 | BnaA07g02500D | 0 | 0.01 | 1E-17 | 5.5977E-17 |
| A0A078GF16 | Heme oxygenase (biliverdin-producing) OS=Brassica napus OX=3708 GN=BnaC06g17920D PE=3 SV=1 | 0 | 5.468 | 5 | 1 | 15 | 1 | 281 | 32.2 | 6.71 | 617 | 1 | BnaC06g17920D | 0 | 0.01 | 1E-17 | 5.5977E-17 |
| A0A078GFE6 | Probable Chlororespiratory reduction 41**,** BnaC06g04300D protein OS=Brassica napus OX=3708 GN=BnaC06g04300D PE=4 SV=1 | 0 | 2.312 | 7 | 1 | 4 | 1 | 212 | 24.3 | 7.93 | 0 | 1 | BnaC06g04300D | 0 | 0.01 | 1E-17 | 5.5977E-17 |
| A0A078GIZ4 | TRAFAC class myosin-kinesin ATPase superfamily, BnaA05g19230D protein OS=Brassica napus OX=3708 GN=BnaA05g19230D PE=3 SV=1 | 0 | 2.426 | 1 | 1 | 10 | 1 | 1549 | 174.7 | 8.97 | 122 | 1 | BnaA05g19230D | 0 | 0.01 | 1E-17 | 5.5977E-17 |
| A0A078GJA7 | Delta(24)-sterol reductase OS=Brassica napus OX=3708 GN=BnaA05g19350D PE=4 SV=1 | 0.008 | 1.061 | 2 | 1 | 1 | 1 | 561 | 65.1 | 7.81 | 33 | 1 | BnaA05g19350D | 0 | 0.01 | 1E-17 | 5.5977E-17 |
| A0A078GJU5 | Malate dehydrogenase OS=Brassica napus OX=3708 GN=BnaA04g13230D PE=3 SV=1 | 0 | 14.686 | 10 | 2 | 42 | 1 | 353 | 37.1 | 7.93 | 441 | 2 | BnaA04g13230D | 0 | 0.029 | 0.000900905 | 0.004905867 |
| A0A078GLT1 | Chlorophyll a-b binding protein, chloroplastic OS=Brassica napus OX=3708 GN=BnaA09g26570D PE=3 SV=1 | 0 | 58.487 | 52 | 6 | 130 | 2 | 267 | 28.3 | 5.44 | 2992 | 6 | BnaA09g26570D | 2 | 0.01 | 1E-17 | 5.5977E-17 |
| A0A078GM84 | 4a-Hydroxytetrahydrobiopterin dehydratase OS=Brassica napus OX=3708 GN=BnaA09g26630D PE=3 SV=1 | 0.003 | 1.256 | 6 | 1 | 4 | 1 | 184 | 20.6 | 9.55 | 43 | 1 | BnaA09g26630D | 0 | 0.01 | 1E-17 | 5.5977E-17 |
| A0A078GNQ7 | Probable Myb-like domain-containing protein, BnaA01g06840D protein OS=Brassica napus OX=3708 GN=BnaA01g06840D PE=4 SV=1 | 0.006 | 1.115 | 16 | 1 | 1 | 1 | 131 | 14.8 | 5.99 | 0 | 1 | BnaA01g06840D | 0 | 0.01 | 1E-17 | 5.5977E-17 |
| A0A078GNX8 | selenoprotein H, (rape) hypothetical protein OS=Brassica napus OX=3708 GN=BnaA01g05890D PE=4 SV=1 | 0 | 2.209 | 7 | 1 | 3 | 1 | 179 | 19.8 | 9.48 | 72 | 1 | BnaA01g05890D | 0 | 0.01 | 1E-17 | 5.5977E-17 |
| A0A078GNY7 | stress-response A/B barrel domain-containing protein At5g22580, BnaC09g36390D protein OS=Brassica napus OX=3708 GN=BnaC09g36390D PE=4 SV=1 | 0 | 14.059 | 30 | 3 | 45 | 3 | 111 | 12.3 | 5.43 | 806 | 3 | BnaC09g36390D | 0 | 0.082 | 0.02595458 | 0.109767112 |
| A0A078GPA7 | ultraviolet-B receptor UVR8, BnaC09g41800D protein OS=Brassica napus OX=3708 GN=BnaC09g41800D PE=4 SV=1 | 0.007 | 1.088 | 2 | 1 | 3 | 1 | 398 | 42.9 | 5.49 | 0 | 1 | BnaC09g41800D | 0 | 0.01 | 1E-17 | 5.5977E-17 |
| A0A078GRF3 | phosphoglycerate mutase-like protein AT74, BnaA05g31980D protein OS=Brassica napus OX=3708 GN=BnaA05g31980D PE=4 SV=1 | 0 | 2.178 | 3 | 1 | 11 | 1 | 317 | 37.1 | 5.07 | 178 | 1 | BnaA05g31980D | 0 | 0.01 | 1E-17 | 5.5977E-17 |
| A0A078GUF2 | protein POST-ILLUMINATION CHLOROPHYLL FLUORESCENCE INCREASE, chloroplastic, (rape) hypothetical protein OS=Brassica napus OX=3708 GN=BnaC05g37470D PE=4 SV=1 | 0 | 13.06 | 12 | 2 | 26 | 2 | 267 | 29.6 | 6.09 | 330 | 2 | BnaC05g37470D | 0 | 0.067 | 0.019214755 | 0.085421944 |
| A0A078GUH2 | (rape) hypothetical protein OS=Brassica napus OX=3708 GN=BnaA06g25710D PE=4 SV=1 | 0 | 1.874 | 7 | 1 | 11 | 1 | 252 | 28.1 | 8.1 | 29 | 1 | BnaA06g25710D | 0 | 0.01 | 1E-17 | 5.5977E-17 |
| A0A078GVN7 | Probable Nucleoplasmin-like domain-containing protein, (rape) hypothetical protein OS=Brassica napus OX=3708 GN=BnaA04g16080D PE=4 SV=1 | 0.004 | 1.196 | 10 | 1 | 1 | 1 | 220 | 24.2 | 4.88 | 0 | 1 | BnaA04g16080D | 0 | 0.01 | 1E-17 | 5.5977E-17 |
| A0A078H0Q1 | Pyruvate kinase OS=Brassica napus OX=3708 GN=BnaC05g28630D PE=3 SV=1 | 0 | 8.76 | 7 | 3 | 26 | 1 | 571 | 62.9 | 7.64 | 79 | 3 | BnaC05g28630D | 0 | 0.01 | 1E-17 | 5.5977E-17 |
| A0A078H1Z4 | Inorganic diphosphatase OS=Brassica napus OX=3708 GN=BnaC04g27080D PE=3 SV=1 | 0 | 4.435 | 6 | 2 | 7 | 1 | 515 | 58.7 | 5.6 | 20 | 2 | BnaC04g27080D | 0 | 0.01 | 1E-17 | 5.5977E-17 |
| A0A078H2A9 | Eukaryotic translation initiation factor 3 subunit E OS=Brassica napus OX=3708 GN=BnaC04g24310D PE=3 SV=1 | 0 | 11.199 | 12 | 4 | 31 | 1 | 441 | 51.7 | 5.77 | 335 | 4 | BnaC04g24310D | 0 | 0.01 | 1E-17 | 5.5977E-17 |
| A0A078H436 | THO complex subunit 4A, BnaA10g12880D protein OS=Brassica napus OX=3708 GN=BnaA10g12880D PE=4 SV=1 | 0 | 1.695 | 5 | 1 | 2 | 1 | 239 | 25.5 | 9.8 | 0 | 1 | BnaA10g12880D | 0 | 0.01 | 1E-17 | 5.5977E-17 |
| A0A078H4X4 | stem-specific protein TSJT1-like, BnaA09g16620D protein OS=Brassica napus OX=3708 GN=BnaA09g16620D PE=4 SV=1 | 0 | 2.639 | 12 | 2 | 15 | 2 | 251 | 27.5 | 6.52 | 128 | 2 | BnaA09g16620D | 0 | 0.01 | 1E-17 | 5.5977E-17 |
| A0A078H5I3 | Non-specific serine/threonine protein kinase OS=Brassica napus OX=3708 GN=BnaA02g07840D PE=3 SV=1 | 0 | 17.983 | 9 | 6 | 48 | 1 | 919 | 103.3 | 7.5 | 376 | 6 | BnaA02g07840D | 0 | 0.01 | 1E-17 | 5.5977E-17 |
| A0A078H631 | Calcium-transporting ATPase OS=Brassica napus OX=3708 GN=BnaC02g11860D PE=3 SV=1 | 0.004 | 1.192 | 1 | 1 | 1 | 1 | 1122 | 121.5 | 7.88 | 0 | 1 | BnaC02g11860D | 0 | 0.037 | 0.001967074 | 0.010369115 |
| A0A078H649 | cinnamoyl-CoA reductase 1, (rape) hypothetical protein OS=Brassica napus OX=3708 GN=BnaA10g11850D PE=4 SV=1 | 0.007 | 1.109 | 3 | 1 | 1 | 1 | 323 | 35.3 | 5.67 | 0 | 1 | BnaA10g11850D | 0 | 0.01 | 1E-17 | 5.5977E-17 |
| A0A078H6W1 | Pectate lyase OS=Brassica napus OX=3708 GN=BnaA01g14130D PE=3 SV=1 | 0 | 2.071 | 4 | 1 | 4 | 1 | 406 | 44.7 | 7.72 | 37 | 1 | BnaA01g14130D | 0 | 0.01 | 1E-17 | 5.5977E-17 |
| A0A078H7J7 | dihydrofolate synthetase, (rape) hypothetical protein OS=Brassica napus OX=3708 GN=BnaA06g36040D PE=3 SV=1 | 0 | 1.624 | 2 | 1 | 10 | 1 | 527 | 56.3 | 6.7 | 141 | 1 | BnaA06g36040D | 0 | 0.01 | 1E-17 | 5.5977E-17 |
| A0A078HAE4 | Adenylosuccinate synthetase, chloroplastic OS=Brassica napus OX=3708 GN=BnaCnng07310D PE=3 SV=1 | 0 | 21.086 | 19 | 6 | 70 | 3 | 490 | 53.4 | 7.71 | 910 | 6 | BnaCnng07310D; PURA | 3 | 0.082 | 0.032025616 | 0.130415224 |
| A0A078HBL4 | guanylate-binding protein 2, (rape) hypothetical protein OS=Brassica napus OX=3708 GN=BnaA09g17790D PE=3 SV=1 | 0 | 13.271 | 6 | 6 | 45 | 6 | 1075 | 121.9 | 6.84 | 291 | 6 | BnaA09g17790D | 0 | 0.036 | 0.001955248 | 0.010316931 |
| A0A078HCC1 | protein HIGH CHLOROPHYLL FLUORESCENCE PHENOTYPE 173, chloroplastic, (rape) hypothetical protein OS=Brassica napus OX=3708 GN=BnaA06g11240D PE=4 SV=1 | 0 | 1.654 | 2 | 1 | 4 | 1 | 606 | 66.7 | 9.1 | 37 | 1 | BnaA06g11240D | 0 | 0.01 | 1E-17 | 5.5977E-17 |
| A0A078HDD3 | Probable plastid-lipid-associated protein 8, chloroplasticBnaC09g37690D protein OS=Brassica napus OX=3708 GN=BnaC09g37690D PE=3 SV=1 | 0 | 2.964 | 5 | 1 | 8 | 1 | 240 | 26.5 | 9.19 | 122 | 1 | BnaC09g37690D | 0 | 0.01 | 1E-17 | 5.5977E-17 |
| A0A078HEI6 | BnaA03g11110D protein OS=Brassica napus OX=3708 GN=BnaA03g11110D PE=3 SV=1 | 0 | 14.489 | 13 | 2 | 38 | 2 | 147 | 16.4 | 4.64 | 672 | 2 | BnaA03g11110D | 0 | 0.01 | 1E-17 | 5.5977E-17 |
| A0A078HEJ2 | ATP-dependent Clp protease proteolytic subunit OS=Brassica napus OX=3708 GN=BnaC06g27560D PE=3 SV=1 | 0 | 7.533 | 5 | 1 | 10 | 1 | 313 | 34.3 | 8.12 | 43 | 1 | BnaC06g27560D | 0 | 0.01 | 1E-17 | 5.5977E-17 |
| A0A078HHJ9 | Superoxide dismutase OS=Brassica napus OX=3708 GN=BnaA01g31350D PE=3 SV=1 | 0 | 18.24 | 39 | 4 | 45 | 1 | 180 | 19.9 | 8.5 | 1425 | 4 | BnaA01g31350D | 0 | 0.01 | 1E-17 | 5.5977E-17 |
| A0A078HII6 | Glucose-6-phosphate 1-epimerase OS=Brassica napus OX=3708 GN=BnaA10g11380D PE=3 SV=1 | 0 | 22.031 | 26 | 7 | 55 | 7 | 313 | 35.5 | 6.14 | 756 | 7 | BnaA10g11380D | 0 | 0.083 | 0.033949477 | 0.137258378 |
| A0A078HMH2 | 6-Phosphogluconate dehydrogenase, decarboxylating OS=Brassica napus OX=3708 GN=BnaA04g11280D PE=3 SV=1 | 0 | 19.243 | 18 | 6 | 78 | 1 | 487 | 53.3 | 5.67 | 1441 | 6 | BnaA04g11280D | 0 | 0.01 | 1E-17 | 5.5977E-17 |
| A0A078HMT6 | Acetolactate synthase I/III small subunit, (rape) hypothetical protein OS=Brassica napus OX=3708 GN=BnaC02g06500D PE=3 SV=1 | 0 | 2.064 | 2 | 1 | 8 | 1 | 475 | 51.9 | 7.37 | 107 | 1 | BnaC02g06500D | 0 | 0.01 | 1E-17 | 5.5977E-17 |
| A0A078HNC1 | Thioglucosidase OS=Brassica napus OX=3708 GN=BnaA02g17600D PE=3 SV=1 | 0 | 6.958 | 5 | 2 | 35 | 2 | 550 | 63.9 | 6.02 | 243 | 2 | BnaA02g17600D | 0 | 0.057 | 0.010505059 | 0.049407902 |
| A0A078HPA5 | Uncharacterized protein (rape) hypothetical protein OS=Brassica napus OX=3708 GN=BnaA03g50510D PE=4 SV=1 | 0 | 2.953 | 3 | 1 | 1 | 1 | 702 | 76.2 | 8.62 | 0 | 1 | BnaA03g50510D | 0 | 0.01 | 1E-17 | 5.5977E-17 |
| A0A078HRQ0 | 26S proteasome non-ATPase regulatory subunit 13 homolog B-like, (rape) hypothetical protein OS=Brassica napus OX=3708 GN=BnaAnng05690D PE=3 SV=1 | 0 | 5.956 | 9 | 3 | 21 | 2 | 386 | 44.1 | 5.39 | 259 | 3 | BnaAnng05690D | 0 | 0.055 | 0.012060843 | 0.056085016 |
| A0A078HT68 | Phosphoribulokinase OS=Brassica napus OX=3708 GN=BnaA08g07000D PE=3 SV=1 | 0 | 62.287 | 36 | 11 | 279 | 1 | 401 | 44.8 | 5.87 | 3930 | 11 | BnaA08g07000D | 0 | 0.01 | 1E-17 | 5.5977E-17 |
| A0A078HTJ8 | BnaA09g38330D protein OS=Brassica napus OX=3708 GN=BnaA09g38330D PE=3 SV=1 | 0 | 2.621 | 11 | 1 | 3 | 1 | 145 | 16.2 | 9.82 | 67 | 1 | BnaA09g38330D | 0 | 0.01 | 1E-17 | 5.5977E-17 |
| A0A078HTU1 | Peptidase C1 family, (rape) hypothetical protein OS=Brassica napus OX=3708 GN=BnaA01g05320D PE=3 SV=1 | 0 | 5.715 | 4 | 1 | 4 | 1 | 367 | 39.9 | 6.61 | 96 | 1 | BnaA01g05320D | 0 | 0.01 | 1E-17 | 5.5977E-17 |
| A0A078HX36 | GDSL esterase/lipase, BnaC05g46600D protein OS=Brassica napus OX=3708 GN=BnaC05g46600D PE=3 SV=1 | 0.004 | 1.252 | 6 | 1 | 1 | 1 | 380 | 42.2 | 6.92 | 25 | 1 | BnaC05g46600D | 0 | 0.01 | 1E-17 | 5.5977E-17 |
| A0A078HXM7 | tRNA-dihydrouridine(47) synthase [NAD(P)(+)] OS=Brassica napus OX=3708 GN=BnaC01g00040D PE=3 SV=1 | 0 | 2.844 | 4 | 2 | 7 | 2 | 667 | 74.9 | 6.99 | 24 | 2 | BnaC01g00040D | 0 | 0.01 | 1E-17 | 5.5977E-17 |
| A0A078HZF7 | Mitochondrial processing peptidase OS=Brassica napus OX=3708 GN=BnaC05g48550D PE=3 SV=1 | 0 | 59.163 | 37 | 14 | 123 | 1 | 529 | 58.8 | 6.71 | 1033 | 14 | BnaC05g48550D | 0 | 0.01 | 1E-17 | 5.5977E-17 |
| A0A078I1I6 | Acyl-coenzyme A oxidase OS=Brassica napus OX=3708 GN=BnaA01g18160D PE=3 SV=1 | 0 | 12.68 | 9 | 5 | 46 | 3 | 664 | 74.3 | 8.06 | 325 | 5 | BnaA01g18160D | 2 | 0.087 | 0.042439489 | 0.16394328 |
| A0A078I1N1 | Xaa-Pro dipeptidase OS=Brassica napus OX=3708 GN=BnaA01g07350D PE=3 SV=1 | 0 | 5.655 | 2 | 1 | 22 | 1 | 493 | 54.9 | 6.1 | 384 | 1 | BnaA01g07350D | 0 | 0.01 | 1E-17 | 5.5977E-17 |
| A0A078I266 | Mitochondrial carrier (TC 2.A.29) family, (rape) hypothetical protein OS=Brassica napus OX=3708 GN=BnaA02g04450D PE=3 SV=1 | 0 | 10.609 | 14 | 3 | 40 | 3 | 299 | 32 | 9.23 | 652 | 3 | BnaA02g04450D | 0 | 0.048 | 0.008289699 | 0.039729738 |
| A0A078I5S9 | Tubulin beta chain OS=Brassica napus OX=3708 GN=BnaA05g12490D PE=3 SV=1 | 0 | 90.123 | 41 | 14 | 364 | 1 | 449 | 50.6 | 4.83 | 3793 | 14 | BnaA05g12490D | 0 | 0.01 | 1E-17 | 5.5977E-17 |
| A0A078I6C5 | Importin subunit beta-1, (rape) hypothetical protein OS=Brassica napus OX=3708 GN=BnaA05g29380D PE=3 SV=1 | 0 | 6.914 | 6 | 3 | 22 | 3 | 873 | 96.4 | 4.75 | 255 | 3 | BnaA05g29380D | 0 | 0.01 | 1E-17 | 5.5977E-17 |
| A0A078I7K6 | Chalcone-flavonone isomerase family protein OS=Brassica napus OX=3708 GN=BnaC06g06390D PE=3 SV=1 | 0 | 1.779 | 4 | 1 | 3 | 1 | 292 | 31 | 9.22 | 30 | 1 | BnaC06g06390D | 0 | 0.01 | 1E-17 | 5.5977E-17 |
| A0A078I7M4 | Universal ribosomal protein uS5 family, (rape) hypothetical protein OS=Brassica napus OX=3708 GN=BnaC01g28170D PE=3 SV=1 | 0 | 25.251 | 26 | 6 | 83 | 3 | 276 | 30 | 10.15 | 974 | 6 | BnaC01g28170D | 4 | 0.047 | 0.004374888 | 0.02215149 |
| A0A078ICK1 | Chlorophyll a-b binding protein, chloroplastic OS=Brassica napus OX=3708 GN=BnaA01g22670D PE=3 SV=1 | 0 | 23.223 | 18 | 5 | 106 | 1 | 273 | 29.2 | 8.25 | 1130 | 5 | BnaA01g22670D | 0 | 0.069 | 0.015183238 | 0.068914727 |
| A0A078ICM7 | UDP-glucose 4-epimerase OS=Brassica napus OX=3708 GN=BnaA01g13540D PE=3 SV=1 | 0 | 2.805 | 8 | 2 | 5 | 1 | 348 | 38.3 | 6.62 | 35 | 2 | BnaA01g13540D | 0 | 0.01 | 1E-17 | 5.5977E-17 |
| A0A078IEW0 | Casein kinase II subunit alpha OS=Brassica napus OX=3708 GN=BnaA09g42220D PE=3 SV=1 | 0 | 6.491 | 8 | 3 | 8 | 1 | 421 | 48.7 | 9.39 | 90 | 3 | BnaA09g42220D | 0 | 0.01 | 1E-17 | 5.5977E-17 |
| A0A078IGJ3 | Ribonuclease P OS=Brassica napus OX=3708 GN=BnaA05g10710D PE=3 SV=1 | 0.005 | 1.182 | 2 | 1 | 3 | 1 | 580 | 66 | 8.91 | 33 | 1 | BnaA05g10710D | 0 | 0.01 | 1E-17 | 5.5977E-17 |
| A0A078IGW4 | BnaCnng16630D protein OS=Brassica napus OX=3708 GN=BnaCnng16630D PE=4 SV=1 | 0.002 | 1.339 | 1 | 1 | 8 | 1 | 1261 | 140.9 | 6.25 | 33 | 1 | BnaCnng16630D | 0 | 0.01 | 1E-17 | 5.5977E-17 |
| A0A078IH14 | D-3-phosphoglycerate dehydrogenase OS=Brassica napus OX=3708 GN=BnaA03g50990D PE=3 SV=1 | 0 | 18.4 | 23 | 9 | 75 | 1 | 598 | 62.7 | 6.68 | 727 | 9 | BnaA03g50990D | 0 | 0.01 | 1E-17 | 5.5977E-17 |
| A0A078IJ78 | Probable W2 domain-containing protein**,** BnaC06g42190D protein OS=Brassica napus OX=3708 GN=BnaC06g42190D PE=3 SV=1 | 0 | 6.031 | 4 | 2 | 12 | 2 | 598 | 67.7 | 5.8 | 264 | 2 | BnaC06g42190D | 0 | 0.01 | 1E-17 | 5.5977E-17 |
| A0A078IKQ3 | bifunctional aspartokinase/homoserine dehydrogenase 1, chloroplastic, (rape) hypothetical protein OS=Brassica napus OX=3708 GN=BnaCnng21170D PE=3 SV=1 | 0 | 2.496 | 2 | 2 | 3 | 2 | 911 | 99 | 7.21 | 0 | 2 | BnaCnng21170D | 0 | 0.01 | 1E-17 | 5.5977E-17 |
| A0A078IKT4 | NAD(P)H-quinone oxidoreductase subunit M, chloroplastic OS=Brassica napus OX=3708 GN=BnaC03g61160D PE=3 SV=1 | 0 | 4.684 | 10 | 1 | 2 | 1 | 220 | 25.1 | 4.78 | 27 | 1 | BnaC03g61160D | 0 | 0.01 | 1E-17 | 5.5977E-17 |
| A0A078IM29 | Translocase of chloroplast OS=Brassica napus OX=3708 GN=BnaA03g55090D PE=3 SV=1 | 0 | 2.755 | 10 | 2 | 2 | 1 | 313 | 34.9 | 9.35 | 0 | 2 | BnaA03g55090D | 0 | 0.01 | 1E-17 | 5.5977E-17 |
| A0A078IRF5 | Phosphoenolpyruvate carboxykinase (ATP) OS=Brassica napus OX=3708 GN=BnaC02g43920D PE=3 SV=1 | 0 | 13.301 | 4 | 2 | 29 | 1 | 668 | 72.9 | 6.54 | 633 | 2 | BnaC02g43920D | 0 | 0.01 | 1E-17 | 5.5977E-17 |
| A0A078ISB1 | Actin-related protein 7 OS=Brassica napus OX=3708 GN=BnaA09g38930D PE=3 SV=1 | 0 | 1.847 | 2 | 1 | 8 | 1 | 403 | 44.3 | 4.83 | 32 | 1 | BnaA09g38930D | 0 | 0.01 | 1E-17 | 5.5977E-17 |
| A0A078ITY5 | Protein ECERIFERUM 26-like, BnaAnng11500D protein OS=Brassica napus OX=3708 GN=BnaAnng11500D PE=3 SV=1 | 0 | 3.633 | 3 | 1 | 2 | 1 | 436 | 48.3 | 6.84 | 71 | 1 | BnaAnng11500D | 0 | 0.01 | 1E-17 | 5.5977E-17 |
| A0A078IW34 | Probable Neprosin domain-containing protein, BnaCnng24580D protein OS=Brassica napus OX=3708 GN=BnaCnng24580D PE=4 SV=1 | 0 | 2.033 | 2 | 1 | 4 | 1 | 494 | 55.1 | 6.15 | 27 | 1 | BnaCnng24580D | 0 | 0.01 | 1E-17 | 5.5977E-17 |
| A0A078IWZ9 | 2-keto-3-deoxy-L-rhamnonate aldolase-like, (rape) hypothetical protein OS=Brassica napus OX=3708 GN=BnaCnng25300D PE=3 SV=1 | 0 | 4.612 | 5 | 1 | 7 | 1 | 359 | 38.5 | 5.72 | 118 | 1 | BnaCnng25300D | 0 | 0.01 | 1E-17 | 5.5977E-17 |
| A0A078IYJ1 | Serine/threonine-protein phosphatase OS=Brassica napus OX=3708 GN=BnaA05g34330D PE=3 SV=1 | 0 | 6.227 | 9 | 2 | 11 | 1 | 313 | 35.7 | 5.22 | 43 | 2 | BnaA05g34330D; LOC108808940 | 0 | 0.01 | 1E-17 | 5.5977E-17 |
| A0A078IYK4 | Spermidine/spermine synthase family, (rape) hypothetical protein OS=Brassica napus OX=3708 GN=BnaA08g20290D PE=3 SV=1 | 0 | 19.147 | 24 | 7 | 46 | 1 | 333 | 36.5 | 5.06 | 638 | 7 | BnaA08g20290D | 0 | 0.01 | 1E-17 | 5.5977E-17 |
| A0A078IZC5 | Pyruvate decarboxylase OS=Brassica napus OX=3708 GN=BnaAnng13920D PE=3 SV=1 | 0 | 6.907 | 4 | 2 | 10 | 1 | 607 | 65.6 | 6.39 | 67 | 2 | BnaAnng13920D | 0 | 0.01 | 1E-17 | 5.5977E-17 |
| A0A078IZQ8 | Blue light photoreceptor, OS=Brassica napus OX=3708 GN=BnaAnng13370D PE=3 SV=1 | 0 | 1.589 | 2 | 1 | 3 | 1 | 713 | 80.2 | 5.27 | 0 | 1 | BnaAnng13370D | 0 | 0.01 | 1E-17 | 5.5977E-17 |
| A0A078J0Q2 | Thioredoxin-dependent peroxiredoxin OS=Brassica napus OX=3708 GN=BnaC03g73430D PE=3 SV=1 | 0 | 43.777 | 30 | 6 | 134 | 1 | 277 | 30.9 | 5.73 | 1581 | 6 | BnaC03g73430D | 0 | 0.01 | 1E-17 | 5.5977E-17 |
| A0A078J5J3 | Thioglucosidase OS=Brassica napus OX=3708 GN=BnaC01g43700D PE=3 SV=1 | 0 | 29.179 | 10 | 5 | 177 | 3 | 528 | 60.2 | 6.87 | 1897 | 5 | BnaC01g43700D | 6 | 0.039 | 0.002265527 | 0.011895544 |
| A0A078J5L4 | Fructose-bisphosphatase OS=Brassica napus OX=3708 GN=BnaAnng15110D PE=3 SV=1 | 0 | 22.294 | 18 | 5 | 65 | 1 | 406 | 44.1 | 6.3 | 556 | 5 | BnaAnng15110D | 0 | 0.01 | 1E-17 | 5.5977E-17 |
| A0A078J5Y0 | Beta-ureidopropionase, OS=Brassica napus OX=3708 GN=BnaAnng15120D PE=3 SV=1 | 0 | 2.043 | 4 | 1 | 4 | 1 | 408 | 45.4 | 6.47 | 34 | 1 | BnaAnng15120D | 0 | 0.01 | 1E-17 | 5.5977E-17 |
| A0A078J5Z2 | Pyruvate kinase, OS=Brassica napus OX=3708 GN=BnaCnng32880D PE=3 SV=1 | 0 | 41.152 | 31 | 11 | 92 | 2 | 527 | 57.4 | 6.9 | 926 | 11 | BnaCnng32880D | 0 | 0.01 | 1E-17 | 5.5977E-17 |
| A0A078J6L2 | Thioglucosidase, OS=Brassica napus OX=3708 GN=BnaCnng39140D PE=3 SV=1 | 0 | 9.965 | 8 | 3 | 32 | 1 | 524 | 60.2 | 6.87 | 276 | 3 | BnaCnng39140D | 0 | 0.01 | 1E-17 | 5.5977E-17 |
| A0A078J712 | Peptidase A1 family, (rape) hypothetical protein OS=Brassica napus OX=3708 GN=BnaA03g55560D PE=3 SV=1 | 0 | 3.189 | 3 | 1 | 15 | 1 | 440 | 47 | 9.1 | 369 | 1 | BnaA03g55560D | 0 | 0.01 | 1E-17 | 5.5977E-17 |
| A0A078J7J7 | Haloacid dehalogenase-like hydrolase domain-containing protein 3, BnaCnng36750D protein OS=Brassica napus OX=3708 GN=BnaCnng36750D PE=4 SV=1 | 0.001 | 1.374 | 3 | 1 | 3 | 1 | 257 | 28.9 | 8.02 | 32 | 1 | BnaCnng36750D | 0 | 0.01 | 1E-17 | 5.5977E-17 |
| A0A078JDI8 | Peptidase S8 family, BnaA02g06800D protein OS=Brassica napus OX=3708 GN=BnaA02g06800D PE=3 SV=1 | 0 | 1.798 | 2 | 1 | 3 | 1 | 763 | 82 | 8.07 | 34 | 1 | BnaA02g06800D | 0 | 0.01 | 1E-17 | 5.5977E-17 |
| A0A078JFG1 | Protein EMBRYO DEFECTIVE 514, BnaCnng45980D protein OS=Brassica napus OX=3708 GN=BnaCnng45980D PE=4 SV=1 | 0 | 1.914 | 5 | 1 | 7 | 1 | 203 | 22.3 | 7.97 | 34 | 1 | BnaCnng45980D | 0 | 0.01 | 1E-17 | 5.5977E-17 |
| A0A078JH18 | ABC transporter B family member 28, BnaCnng49290D protein OS=Brassica napus OX=3708 GN=BnaCnng49290D PE=4 SV=1 | 0 | 7.027 | 4 | 2 | 10 | 2 | 734 | 80 | 9.09 | 189 | 2 | BnaCnng49290D | 0 | 0.01 | 1E-17 | 5.5977E-17 |
| A0A078JHB7 | Nascent polypeptide-associated complex subunit beta OS=Brassica napus OX=3708 GN=BnaAnng20100D PE=3 SV=1 | 0 | 17.629 | 18 | 4 | 38 | 2 | 230 | 25.1 | 8.81 | 674 | 4 | BnaAnng20100D | 0 | 0.01 | 1E-17 | 5.5977E-17 |
| A0A078JHM6 | Probable [Plectin/eS10 N-terminal domain-containing protein](https://www.uniprot.org/uniprotkb/A0A3P6BHD3/entry) (rape) hypothetical protein OS=Brassica napus OX=3708 GN=BnaA08g30820D PE=3 SV=1 | 0 | 5.206 | 14 | 2 | 14 | 2 | 257 | 28.4 | 9.54 | 86 | 2 | BnaA08g30820D | 0 | 0.069 | 0.014969673 | 0.068075159 |
| A0A078JLJ1 | Indole-3-glycerol-phosphate synthase OS=Brassica napus OX=3708 GN=BnaA03g57920D PE=3 SV=1 | 0 | 7.314 | 10 | 3 | 15 | 3 | 402 | 44.1 | 8.34 | 128 | 3 | BnaA03g57920D | 0 | 0.01 | 1E-17 | 5.5977E-17 |
| A0A078JRA0 | Probable Helitron helicase-like domain-containing protein**,** BnaCnng57090D protein OS=Brassica napus OX=3708 GN=BnaCnng57090D PE=4 SV=1 | 0 | 1.611 | 2 | 1 | 1 | 1 | 433 | 49.6 | 9.33 | 38 | 1 | BnaCnng57090D | 0 | 0.047 | 0.004420729 | 0.022288797 |
| A0A078JVB3 | Glutathione transferase OS=Brassica napus OX=3708 GN=BnaAnng37730D PE=3 SV=1 | 0 | 19.714 | 15 | 3 | 12 | 2 | 264 | 29.5 | 8.07 | 195 | 3 | BnaAnng37730D | 0 | 0.01 | 1E-17 | 5.5977E-17 |
| A0A078K063 | protein RALF-like 34, (rape) hypothetical protein OS=Brassica napus OX=3708 GN=BnaAnng39730D PE=3 SV=1 | 0 | 1.523 | 9 | 1 | 4 | 1 | 132 | 15 | 6.77 | 76 | 1 | BnaAnng39730D | 0 | 0.01 | 1E-17 | 5.5977E-17 |
| A0A1B1XZB1 | 50S ribosomal protein L23, chloroplastic OS=Brassica napus var. napus OX=138011 GN=rpl23 PE=3 SV=1 | 0 | 2.119 | 16 | 1 | 10 | 1 | 93 | 10.8 | 10.65 | 42 | 1 | rpl23; rpl23-A; rpl23-B | 0 | 0.01 | 1E-17 | 5.5977E-17 |
| A0A1B1XZH7 | DNA-directed RNA polymerase subunit alpha OS=Brassica napus var. napus OX=138011 GN=rpoA PE=3 SV=1 | 0 | 1.921 | 6 | 1 | 2 | 1 | 327 | 37.8 | 7.84 | 35 | 1 | rpoA | 0 | 0.01 | 1E-17 | 5.5977E-17 |
| A0A1B1XZQ3 | Small ribosomal subunit protein bS18c OS=Brassica napus var. napus OX=138011 GN=rps18 PE=3 SV=1 | 0 | 2.207 | 12 | 1 | 7 | 1 | 101 | 12 | 12.16 | 25 | 1 | rps18 | 0 | 0.01 | 1E-17 | 5.5977E-17 |
| A0A1B1Y0C1 | Cytochrome b6-f complex subunit 4 OS=Brassica napus var. napus OX=138011 GN=petD PE=3 SV=1 | 0.001 | 1.365 | 9 | 1 | 1 | 1 | 160 | 17.4 | 7.18 | 32 | 1 | petD | 0 | 0.01 | 1E-17 | 5.5977E-17 |
| A0A1B1Y0D5 | ATP synthase subunit a, chloroplastic OS=Brassica napus var. napus OX=138011 GN=atpI PE=3 SV=1 | 0 | 4.625 | 10 | 1 | 9 | 1 | 249 | 27.2 | 5.02 | 95 | 1 | atpI | 0 | 0.01 | 1E-17 | 5.5977E-17 |
| A0A1B1Y0E3 | Photosystem I P700 chlorophyll a apoprotein A1 OS=Brassica napus var. napus OX=138011 GN=psaA PE=3 SV=1 | 0 | 2.022 | 2 | 1 | 3 | 1 | 750 | 83.1 | 7.11 | 39 | 1 | psaA | 0 | 0.01 | 1E-17 | 5.5977E-17 |
| A0A482K1T8 | Ribulose bisphosphate carboxylase large chain (Fragment) OS=Brassica napus OX=3708 GN=rbcL PE=3 SV=1 | 0 | 75.275 | 46 | 12 | 426 | 7 | 187 | 20.7 | 8.84 | 3541 | 12 | rbcL | 0 | 0.01 | 1E-17 | 5.5977E-17 |
| A0A7T7BWD1 | NADH-ubiquinone oxidoreductase chain 1 OS=Brassica napus OX=3708 GN=nad1 PE=3 SV=1 | 0.002 | 1.342 | 4 | 1 | 1 | 1 | 325 | 35.8 | 9.48 | 0 | 1 | nad1 | 0 | 0.01 | 1E-17 | 5.5977E-17 |
| A0A816HX21 | Importin-5, (rape) hypothetical protein OS=Brassica napus OX=3708 GN=DARMORV10_C03P11210.1 PE=4 SV=1 | 0 | 16.707 | 8 | 7 | 42 | 1 | 1115 | 123.6 | 4.83 | 246 | 7 | DARMORV10_C03P11210.1 | 1 | 0.01 | 1E-17 | 5.5977E-17 |
| A0A816HYZ4 | 4a-Hydroxytetrahydrobiopterin dehydratase OS=Brassica napus OX=3708 GN=DARMORV10_C03P19210.1 PE=3 SV=1 | 0 | 3.8 | 6 | 1 | 3 | 1 | 239 | 26.5 | 8.27 | 94 | 1 | DARMORV10_C03P19210.1 | 0 | 0.01 | 1E-17 | 5.5977E-17 |
| A0A816I220 | Probable Kinesin motor domain-containing protein, (rape) hypothetical protein OS=Brassica napus OX=3708 GN=DARMORV10_C03P05120.1 PE=3 SV=1 | 0 | 9.377 | 3 | 3 | 38 | 3 | 1257 | 139.3 | 6.02 | 531 | 3 | DARMORV10_C03P05120.1 | 0 | 0.097 | 0.045245707 | 0.173433037 |
| A0A816I295 | alpha-D-xyloside xylohydrolase OS=Brassica napus OX=3708 GN=DARMORV10_C03P05920.1 PE=3 SV=1 | 0 | 2.063 | 1 | 1 | 4 | 1 | 899 | 100.5 | 5.73 | 51 | 1 | DARMORV10_C03P05920.1 | 0 | 0.01 | 1E-17 | 5.5977E-17 |
| A0A816I4Y0 | ras-related protein RABE1e, (rape) hypothetical protein OS=Brassica napus OX=3708 GN=DARMORV10_C03P01550.1 PE=3 SV=1 | 0 | 6.885 | 16 | 3 | 63 | 1 | 223 | 24.8 | 7.83 | 516 | 3 | DARMORV10_C03P01550.1 | 0 | 0.01 | 1E-17 | 5.5977E-17 |
| A0A816I4Z1 | NADH dehydrogenase [ubiquinone] flavoprotein 1, mitochondrial OS=Brassica napus OX=3708 GN=DARMORV10_C03P04400.1 PE=3 SV=1 | 0 | 32.144 | 28 | 11 | 86 | 11 | 486 | 53.4 | 8.27 | 674 | 11 | DARMORV10_C03P04400.1 | 0 | 0.046 | 0.004201477 | 0.021328892 |
| A0A816I526 | Eukaryotic translation initiation factor NCBP OS=Brassica napus OX=3708 GN=DARMORV10_C03P10090.1 PE=3 SV=1 | 0 | 2.055 | 5 | 1 | 13 | 1 | 218 | 25.3 | 7.15 | 82 | 1 | DARMORV10_C03P10090.1 | 0 | 0.01 | 1E-17 | 5.5977E-17 |
| A0A816I555 | Pyruvate kinase OS=Brassica napus OX=3708 GN=DARMORV10_C03P15820.1 PE=3 SV=1 | 0 | 20.481 | 21 | 7 | 72 | 1 | 498 | 54.5 | 6.13 | 763 | 7 | DARMORV10_C03P15820.1 | 0 | 0.01 | 1E-17 | 5.5977E-17 |
| A0A816I5Y0 | Probable Dihydrodipicolinate reductase N-terminal domain-containing protein (rape) hypothetical protein OS=Brassica napus OX=3708 GN=DARMORV10_C03P18520.1 PE=3 SV=1 | 0.008 | 1.073 | 3 | 1 | 1 | 1 | 334 | 36.2 | 7.39 | 32 | 1 | DARMORV10_C03P18520.1 | 0 | 0.01 | 1E-17 | 5.5977E-17 |
| A0A816I6U6 | GDSL esterase/lipase LTL1-like, (rape) hypothetical protein OS=Brassica napus OX=3708 GN=DARMORV10_C03P40760.1 PE=3 SV=1 | 0 | 3.437 | 7 | 2 | 6 | 2 | 368 | 40.5 | 5.68 | 16 | 2 | DARMORV10_C03P40760.1 | 0 | 0.01 | 1E-17 | 5.5977E-17 |
| A0A816I942 | auxin-induced in root cultures protein 12-like, (rape) hypothetical protein OS=Brassica napus OX=3708 GN=DARMORV10_C03P42830.1 PE=4 SV=1 | 0.006 | 1.165 | 3 | 1 | 6 | 1 | 265 | 27.5 | 9.29 | 0 | 1 | DARMORV10_C03P42830.1 | 0 | 0.01 | 1E-17 | 5.5977E-17 |
| A0A816I965 | translationally-controlled tumor protein homolog, (rape) hypothetical protein OS=Brassica napus OX=3708 GN=DARMORV10_C03P48370.1 PE=3 SV=1 | 0 | 10.495 | 23 | 3 | 66 | 1 | 168 | 19 | 4.67 | 647 | 3 | DARMORV10_C03P48370.1 | 0 | 0.01 | 1E-17 | 5.5977E-17 |
| A0A816I9N6 | 2-Oxoglutarate dehydrogenase, mitochondrial OS=Brassica napus OX=3708 GN=DARMORV10_C03P59910.1 PE=3 SV=1 | 0 | 56.594 | 17 | 14 | 152 | 7 | 1015 | 114.9 | 6.92 | 1849 | 14 | DARMORV10_C03P59910.1 | 0 | 0.086 | 0.030340817 | 0.124524619 |
| A0A816IA79 | proteasome subunit alpha type-4-A-like, (rape) hypothetical protein OS=Brassica napus OX=3708 GN=DARMORV10_C03P52170.1 PE=3 SV=1 | 0 | 9.774 | 11 | 1 | 16 | 1 | 178 | 19.9 | 6.38 | 672 | 1 | DARMORV10_C03P52170.1 | 0 | 0.01 | 1E-17 | 5.5977E-17 |
| A0A816IBF3 | Calcium-transporting ATPase OS=Brassica napus OX=3708 GN=DARMORV10_C03P51530.1 PE=3 SV=1 | 0 | 2.744 | 1 | 1 | 8 | 1 | 1438 | 157.2 | 6.74 | 79 | 1 | DARMORV10_C03P51530.1 | 0 | 0.01 | 1E-17 | 5.5977E-17 |
| A0A816IDT1 | Dr1-associated corepressor, (rape) hypothetical protein (Fragment) OS=Brassica napus OX=3708 GN=DARMORV10_C03P45620.1 PE=4 SV=1 | 0 | 2.084 | 3 | 1 | 9 | 1 | 320 | 35.8 | 5.33 | 39 | 1 | DARMORV10_C03P45620.1 | 0 | 0.01 | 1E-17 | 5.5977E-17 |
| A0A816IE71 | AT-hook motif nuclear-localized protein OS=Brassica napus OX=3708 GN=DARMORV10_C03P33150.1 PE=4 SV=1 | 0 | 2.627 | 6 | 1 | 8 | 1 | 263 | 27.7 | 7.9 | 71 | 1 | DARMORV10_C03P33150.1 | 0 | 0.01 | 1E-17 | 5.5977E-17 |
| A0A816IE78 | Purple acid phosphatase OS=Brassica napus OX=3708 GN=DARMORV10_C03P57040.1 PE=3 SV=1 | 0 | 2.647 | 4 | 2 | 6 | 1 | 470 | 54.6 | 7.65 | 30 | 2 | DARMORV10_C03P57040.1 | 0 | 0.01 | 1E-17 | 5.5977E-17 |
| A0A816IH85 | putative hydrolase C777.06c, (rape) hypothetical protein OS=Brassica napus OX=3708 GN=DARMORV10_C03P46600.1 PE=4 SV=1 | 0 | 2.596 | 4 | 1 | 3 | 1 | 358 | 40 | 7.52 | 25 | 1 | DARMORV10_C03P46600.1 | 0 | 0.095 | 0.047368789 | 0.179789585 |
| A0A816II30 | Protein-L-isoaspartate O-methyltransferase (Fragment) OS=Brassica napus OX=3708 GN=DARMORV10_C03P66480.1 PE=3 SV=1 | 0 | 5.766 | 6 | 1 | 9 | 1 | 282 | 30.4 | 7.06 | 227 | 1 | DARMORV10_C03P66480.1 | 0 | 0.01 | 1E-17 | 5.5977E-17 |
| A0A816IM64 | Plasma membrane ATPase OS=Brassica napus OX=3708 GN=DARMORV10_C03P88830.1 PE=3 SV=1 | 0 | 19.446 | 21 | 11 | 61 | 1 | 660 | 72.9 | 9.09 | 206 | 11 | DARMORV10_C03P88830.1 | 0 | 0.01 | 1E-17 | 5.5977E-17 |
| A0A816IME9 | ATP-dependent zinc metalloprotease FTSH 7, chloroplastic (rape) hypothetical protein OS=Brassica napus OX=3708 GN=DARMORV10_C03P71290.1 PE=3 SV=1 | 0.003 | 1.266 | 1 | 1 | 1 | 1 | 812 | 89 | 8.95 | 29 | 1 | DARMORV10_C03P71290.1 | 0 | 0.01 | 1E-17 | 5.5977E-17 |
| A0A816ING8 | Protein DETOXIFICATION OS=Brassica napus OX=3708 GN=DARMORV10_C03P77090.1 PE=3 SV=1 | 0.003 | 1.302 | 0 | 1 | 9 | 1 | 1976 | 216 | 6.13 | 0 | 1 | DARMORV10_C03P77090.1 | 0 | 0.01 | 1E-17 | 5.5977E-17 |
| A0A816IRH3 | Rhodanese-like domain-containing protein 4, chloroplastic (rape) hypothetical protein OS=Brassica napus OX=3708 GN=DARMORV10_C09P00870.1 PE=4 SV=1 | 0 | 8.652 | 5 | 3 | 38 | 1 | 498 | 53.2 | 5.66 | 163 | 3 | DARMORV10_C09P00870.1 | 0 | 0.01 | 1E-17 | 5.5977E-17 |
| A0A816IRI9 | Nuclear transport factor 2, (rape) hypothetical protein OS=Brassica napus OX=3708 GN=DARMORV10_C09P08020.1 PE=4 SV=1 | 0 | 6.255 | 6 | 2 | 23 | 2 | 520 | 56 | 5.94 | 143 | 2 | DARMORV10_C09P08020.1 | 0 | 0.068 | 0.020997138 | 0.092103721 |
| A0A816IZ13 | Uncharacterized protein (rape) hypothetical protein OS=Brassica napus OX=3708 GN=DARMORV10_C09P36020.1 PE=4 SV=1 | 0 | 5.466 | 10 | 1 | 27 | 1 | 142 | 15.8 | 6.73 | 394 | 1 | DARMORV10_C09P36020.1 | 0 | 0.01 | 1E-17 | 5.5977E-17 |
| A0A816IZT2 | Uncharacterized protein (rape) hypothetical protein OS=Brassica napus OX=3708 GN=DARMORV10_C09P45650.1 PE=4 SV=1 | 0 | 9.213 | 34 | 1 | 29 | 1 | 44 | 5.2 | 6.19 | 386 | 1 | DARMORV10_C09P45650.1 | 0 | 0.01 | 1E-17 | 5.5977E-17 |
| A0A816IZV9 | Probable glucan 1,3-alpha-glucosidase OS=Brassica napus OX=3708 GN=DARMORV10_C09P09530.1 PE=3 SV=1 | 0 | 20.602 | 8 | 5 | 31 | 2 | 910 | 103 | 5.59 | 274 | 5 | DARMORV10_C09P09530.1 | 4 | 0.083 | 0.03815208 | 0.151169152 |
| A0A816J0G2 | Zeaxanthin epoxidase, chloroplastic OS=Brassica napus OX=3708 GN=DARMORV10_C09P11330.1 PE=4 SV=1 | 0 | 8.313 | 5 | 4 | 30 | 4 | 908 | 101.9 | 7.06 | 145 | 4 | DARMORV10_C09P11330.1 | 0 | 0.092 | 0.037520221 | 0.149189702 |
| A0A816J613 | Tubulin beta chain OS=Brassica napus OX=3708 GN=DARMORV10_C09P64620.1 PE=3 SV=1 | 0 | 63.24 | 34 | 13 | 274 | 3 | 481 | 54.6 | 5.17 | 2538 | 13 | DARMORV10_C09P64620.1 | 0 | 0.01 | 1E-17 | 5.5977E-17 |
| A0A816J6H1 | Protein MAINTENANCE OF PSII UNDER HIGH LIGHT 1-like (rape) hypothetical protein OS=Brassica napus OX=3708 GN=DARMORV10_C09P69150.1 PE=4 SV=1 | 0 | 4.62 | 5 | 1 | 17 | 1 | 278 | 29.6 | 6.06 | 152 | 1 | DARMORV10_C09P69150.1 | 0 | 0.01 | 1E-17 | 5.5977E-17 |
| A0A816J6W7 | Conserved oligomeric Golgi complex subunit 1 OS=Brassica napus OX=3708 GN=DARMORV10_C09P60740.1 PE=3 SV=1 | 0.007 | 1.096 | 1 | 1 | 1 | 1 | 1479 | 165 | 6.04 | 39 | 1 | DARMORV10_C09P60740.1 | 0 | 0.01 | 1E-17 | 5.5977E-17 |
| A0A816J9K0 | Probable Plastid lipid-associated protein/fibrillin conserved domain-containing protein, (rape) hypothetical protein OS=Brassica napus OX=3708 GN=DARMORV10_C04P01080.1 PE=3 SV=1 | 0 | 1.694 | 1 | 1 | 4 | 1 | 897 | 101.3 | 5.87 | 0 | 1 | DARMORV10_C04P01080.1 | 0 | 0.01 | 1E-17 | 5.5977E-17 |
| A0A816JAL7 | Subtilisin-like protease SBT1.3, (rape) hypothetical protein OS=Brassica napus OX=3708 GN=DARMORV10_C09P41180.1 PE=3 SV=1 | 0.001 | 1.389 | 1 | 1 | 5 | 1 | 775 | 83.8 | 9.1 | 33 | 1 | DARMORV10_C09P41180.1 | 0 | 0.01 | 1E-17 | 5.5977E-17 |
| A0A816JAU4 | Probable Hydroxyproline O-arabinosyltransferase-like domain-containing protein, (rape) hypothetical protein OS=Brassica napus OX=3708 GN=DARMORV10_C09P63600.1 PE=3 SV=1 | 0 | 16.347 | 8 | 7 | 134 | 7 | 781 | 87 | 9.52 | 1289 | 7 | DARMORV10_C09P63600.1 | 0 | 0.095 | 0.038472483 | 0.151793106 |
| A0A816JB47 | Probable BEACH-type PH domain-containing protein, (rape) hypothetical protein OS=Brassica napus OX=3708 GN=DARMORV10_C04P06480.1 PE=4 SV=1 | 0.002 | 1.357 | 0 | 1 | 9 | 1 | 3081 | 337.1 | 6.04 | 48 | 1 | DARMORV10_C04P06480.1 | 0 | 0.01 | 1E-17 | 5.5977E-17 |
| A0A816JB71 | Photosystem II D2 protein OS=Brassica napus OX=3708 GN=DARMORV10_C09P40430.1 PE=3 SV=1 | 0 | 32.481 | 24 | 6 | 70 | 1 | 359 | 40.3 | 6.15 | 821 | 6 | psbD | 0 | 0.01 | 1E-17 | 5.5977E-17 |
| A0A816JCK2 | cathepsin B-like protease 2, (rape) hypothetical protein OS=Brassica napus OX=3708 GN=DARMORV10_C04P03330.1 PE=3 SV=1 | 0 | 7.57 | 9 | 2 | 13 | 1 | 393 | 43.9 | 7.27 | 186 | 2 | DARMORV10_C04P03330.1 | 0 | 0.01 | 1E-17 | 5.5977E-17 |
| A0A816JD15 | Uncharacterized protein. (rape) hypothetical protein OS=Brassica napus OX=3708 GN=DARMORV10_C09P46830.1 PE=4 SV=1 | 0 | 2.873 | 8 | 1 | 4 | 1 | 249 | 27.9 | 7.24 | 86 | 1 | DARMORV10_C09P46830.1 | 0 | 0.01 | 1E-17 | 5.5977E-17 |
| A0A816JH76 | Superoxide dismutase OS=Brassica napus OX=3708 GN=DARMORV10_C09P58880.1 PE=3 SV=1 | 0 | 2.298 | 7 | 1 | 5 | 1 | 180 | 19.1 | 8.16 | 0 | 1 | DARMORV10_C09P58880.1 | 0 | 0.01 | 1E-17 | 5.5977E-17 |
| A0A816JK99 | Cysteine synthase OS=Brassica napus OX=3708 GN=DARMORV10_C04P70750.1 PE=3 SV=1 | 0 | 41.231 | 25 | 9 | 146 | 1 | 515 | 54.6 | 7.64 | 2123 | 9 | DARMORV10_C04P70750.1 | 0 | 0.01 | 1E-17 | 5.5977E-17 |
| A0A816JSD3 | Uncharacterized protein (rape) hypothetical protein OS=Brassica napus OX=3708 GN=DARMORV10_C04P51900.1 PE=4 SV=1 | 0.003 | 1.294 | 3 | 1 | 1 | 1 | 409 | 46.7 | 8.4 | 0 | 1 | DARMORV10_C04P51900.1 | 0 | 0.01 | 1E-17 | 5.5977E-17 |
| A0A816JT56 | Eukaryotic translation initiation factor 3 subunit J OS=Brassica napus OX=3708 GN=DARMORV10_C04P49430.1 PE=3 SV=1 | 0 | 7.364 | 6 | 1 | 36 | 1 | 248 | 27.6 | 5.06 | 309 | 1 | DARMORV10_C04P49430.1 | 0 | 0.01 | 1E-17 | 5.5977E-17 |
| A0A816JTM6 | ATP synthase subunit alpha OS=Brassica napus OX=3708 GN=DARMORV10_C04P19640.1 PE=3 SV=1 | 0 | 72.357 | 42 | 21 | 238 | 2 | 506 | 55.1 | 7.36 | 2076 | 21 | DARMORV10_C04P19640.1 | 0 | 0.01 | 1E-17 | 5.5977E-17 |
| A0A816JUZ4 | Probable RuBisCO large subunit-binding protein subunit alpha, chloroplastic, (rape) hypothetical protein OS=Brassica napus OX=3708 GN=DARMORV10_C04P23540.1 PE=3 SV=1 | 0 | 31.639 | 22 | 10 | 128 | 1 | 518 | 54.7 | 5.59 | 2389 | 10 | DARMORV10_C04P23540.1 | 0 | 0.01 | 1E-17 | 5.5977E-17 |
| A0A816JVZ8 | Probable peroxygenase 3 (rape) hypothetical protein OS=Brassica napus OX=3708 GN=DARMORV10_C04P62820.1 PE=3 SV=1 | 0 | 2.809 | 5 | 1 | 15 | 1 | 244 | 27.5 | 5.25 | 83 | 1 | DARMORV10_C04P62820.1 | 0 | 0.01 | 1E-17 | 5.5977E-17 |
| A0A816JW56 | (rape) hypothetical protein OS=Brassica napus OX=3708 GN=DARMORV10_C04P64000.1 PE=4 SV=1 | 0 | 4.19 | 6 | 1 | 16 | 1 | 214 | 24.7 | 7.4 | 179 | 1 | DARMORV10_C04P64000.1 | 0 | 0.01 | 1E-17 | 5.5977E-17 |
| A0A816JYJ5 | Phospholipase A1 OS=Brassica napus OX=3708 GN=DARMORV10_C04P69860.1 PE=3 SV=1 | 0 | 2.516 | 4 | 1 | 6 | 1 | 413 | 46.3 | 5.8 | 112 | 1 | DARMORV10_C04P69860.1 | 0 | 0.01 | 1E-17 | 5.5977E-17 |
| A0A816K0C0 | Probable Acyl-coenzyme A oxidase 4, peroxisomal (rape) hypothetical protein OS=Brassica napus OX=3708 GN=DARMORV10_C04P41140.1 PE=3 SV=1 | 0 | 9.272 | 8 | 3 | 25 | 3 | 479 | 52.4 | 9.07 | 116 | 3 | DARMORV10_C04P41140.1 | 0 | 0.086 | 0.028255037 | 0.117221479 |
| A0A816K1G9 | UDP-arabinopyranose mutase OS=Brassica napus OX=3708 GN=DARMORV10_C02P07880.1 PE=3 SV=1 | 0 | 28.954 | 24 | 7 | 97 | 1 | 363 | 41.1 | 6.13 | 921 | 7 | DARMORV10_C02P07880.1 | 0 | 0.01 | 1E-17 | 5.5977E-17 |
| A0A816K281 | Glutathione transferase (Fragment) OS=Brassica napus OX=3708 GN=DARMORV10_C02P08840.1 PE=3 SV=1 | 0.008 | 1.078 | 5 | 1 | 2 | 1 | 429 | 47.8 | 6.73 | 20 | 1 | DARMORV10_C02P08840.1 | 0 | 0.01 | 1E-17 | 5.5977E-17 |
| A0A816KDK9 | Expansin OS=Brassica napus OX=3708 GN=DARMORV10_C02P51120.1 PE=3 SV=1 | 0 | 5.354 | 8 | 1 | 5 | 1 | 253 | 27.2 | 9.61 | 102 | 1 | DARMORV10_C02P51120.1 | 0 | 0.01 | 1E-17 | 5.5977E-17 |
| A0A816KKL8 | Proteasome subunit beta (Fragment) OS=Brassica napus OX=3708 GN=DARMORV10_C02P34590.1 PE=3 SV=1 | 0 | 5.076 | 15 | 2 | 14 | 1 | 205 | 23 | 6.55 | 71 | 2 | DARMORV10_C02P34590.1 | 0 | 0.01 | 1E-17 | 5.5977E-17 |
| A0A816KQY7 | photosystem I subunit O, (rape) hypothetical protein OS=Brassica napus OX=3708 GN=DARMORV10_C05P06520.1 PE=4 SV=1 | 0 | 6.764 | 17 | 2 | 4 | 2 | 145 | 15.6 | 9.89 | 83 | 2 | DARMORV10_C05P06520.1 | 0 | 0.01 | 1E-17 | 5.5977E-17 |
| A0A816KUU9 | Protoporphyrinogen oxidase OS=Brassica napus OX=3708 GN=DARMORV10_C05P01130.1 PE=3 SV=1 | 0 | 2.221 | 3 | 1 | 7 | 1 | 538 | 57.4 | 9.03 | 36 | 1 | DARMORV10_C05P01130.1 | 0 | 0.01 | 1E-17 | 5.5977E-17 |
| A0A816KW56 | butanoate--CoA ligase AAE1, (rape) hypothetical protein OS=Brassica napus OX=3708 GN=DARMORV10_C05P19130.1 PE=3 SV=1 | 0.001 | 1.442 | 2 | 1 | 11 | 1 | 576 | 63.2 | 8.81 | 55 | 1 | DARMORV10_C05P19130.1 | 0 | 0.057 | 0.011522901 | 0.053723394 |
| A0A816KW83 | Probable ENTH domain-containing protein (rape) hypothetical protein OS=Brassica napus OX=3708 GN=DARMORV10_C05P19300.1 PE=4 SV=1 | 0 | 9.796 | 3 | 3 | 18 | 2 | 1172 | 126.7 | 5.74 | 30 | 3 | DARMORV10_C05P19300.1 | 1 | 0.01 | 1E-17 | 5.5977E-17 |
| A0A816KXJ7 | Probable Fe2OG dioxygenase domain-containing protein (rape) hypothetical protein OS=Brassica napus OX=3708 GN=DARMORV10_C02P60220.1 PE=3 SV=1 | 0 | 2.596 | 4 | 1 | 14 | 1 | 341 | 39.2 | 5.94 | 93 | 1 | DARMORV10_C02P60220.1 | 0 | 0.01 | 1E-17 | 5.5977E-17 |
| A0A816KXY7 | Expansin OS=Brassica napus OX=3708 GN=DARMORV10_C05P24190.1 PE=3 SV=1 | 0 | 13.407 | 11 | 2 | 15 | 1 | 258 | 27.6 | 8.56 | 548 | 2 | DARMORV10_C05P24190.1 | 0 | 0.01 | 1E-17 | 5.5977E-17 |
| A0A816L2D0 | UDP-glucose 6-dehydrogenase OS=Brassica napus OX=3708 GN=DARMORV10_C05P24470.1 PE=3 SV=1 | 0 | 3.915 | 3 | 2 | 11 | 1 | 935 | 104.2 | 5.14 | 182 | 2 | DARMORV10_C05P24470.1 | 0 | 0.01 | 1E-17 | 5.5977E-17 |
| A0A816L3K6 | RNA helicase OS=Brassica napus OX=3708 GN=DARMORV10_C05P38490.1 PE=3 SV=1 | 0 | 1.512 | 1 | 1 | 1 | 1 | 1045 | 119.1 | 5.69 | 0 | 1 | DARMORV10_C05P38490.1 | 0 | 0.01 | 1E-17 | 5.5977E-17 |
| A0A816L5G1 | Uncharacterized protein, (rape) hypothetical protein OS=Brassica napus OX=3708 GN=DARMORV10_C05P07850.1 PE=3 SV=1 | 0 | 3.686 | 3 | 1 | 2 | 1 | 580 | 65.4 | 8.34 | 53 | 1 | DARMORV10_C05P07850.1 | 0 | 0.01 | 1E-17 | 5.5977E-17 |
| A0A816L981 | Phosphoenolpyruvate carboxylase OS=Brassica napus OX=3708 GN=DARMORV10_C05P52230.1 PE=3 SV=1 | 0 | 28.746 | 16 | 13 | 81 | 1 | 967 | 110.2 | 6.23 | 557 | 13 | DARMORV10_C05P52230.1 | 0 | 0.01 | 1E-17 | 5.5977E-17 |
| A0A816L9H0 | phytochrome B-like, (rape) hypothetical protein OS=Brassica napus OX=3708 GN=DARMORV10_C05P49390.1 PE=3 SV=1 | 0 | 2.715 | 2 | 2 | 8 | 2 | 1191 | 131.4 | 6 | 52 | 2 | DARMORV10_C05P49390.1 | 0 | 0.01 | 1E-17 | 5.5977E-17 |
| A0A816LHQ9 | (rape) hypothetical protein OS=Brassica napus OX=3708 GN=DARMORV10_C05P60840.1 PE=3 SV=1 | 0.001 | 1.416 | 1 | 1 | 2 | 1 | 1133 | 131.3 | 6.04 | 16 | 1 | DARMORV10_C05P60840.1 | 0 | 0.01 | 1E-17 | 5.5977E-17 |
| A0A816MES8 | Cyanate hydratase OS=Brassica napus OX=3708 GN=CYN PE=3 SV=1 | 0 | 3.549 | 13 | 1 | 3 | 1 | 168 | 18.7 | 6.01 | 52 | 1 | CYN | 0 | 0.01 | 1E-17 | 5.5977E-17 |
| A0A816MFY0 | berberine bridge enzyme-like 13, (rape) hypothetical protein OS=Brassica napus OX=3708 GN=DARMORV10_C07P14920.1 PE=3 SV=1 | 0 | 1.966 | 2 | 1 | 1 | 1 | 552 | 62.2 | 9.45 | 30 | 1 | DARMORV10_C07P14920.1 | 0 | 0.01 | 1E-17 | 5.5977E-17 |
| A0A816MTW1 | Cullin-associated NEDD8-dissociated protein 1 OS=Brassica napus OX=3708 GN=DARMORV10_C07P33270.1 PE=3 SV=1 | 0 | 8.063 | 4 | 3 | 9 | 3 | 1253 | 139.1 | 6.21 | 94 | 3 | DARMORV10_C07P33270.1 | 0 | 0.01 | 1E-17 | 5.5977E-17 |
| A0A816MYX1 | Probable USP domain-containing protein, (rape) hypothetical protein OS=Brassica napus OX=3708 GN=DARMORV10_C07P51810.1 PE=3 SV=1 | 0 | 1.53 | 3 | 1 | 4 | 1 | 487 | 57 | 7.59 | 0 | 1 | DARMORV10_C07P51810.1 | 0 | 0.01 | 1E-17 | 5.5977E-17 |
| A0A816N0S2 | DNA mismatch repair protein MSH3 OS=Brassica napus OX=3708 GN=DARMORV10_C07P54390.1 PE=3 SV=1 | 0 | 1.817 | 1 | 1 | 5 | 1 | 1864 | 208.7 | 6.74 | 18 | 1 | DARMORV10_C07P54390.1 | 0 | 0.01 | 1E-17 | 5.5977E-17 |
| A0A816NHJ6 | probable polyamine oxidase 5, (rape) hypothetical protein OS=Brassica napus OX=3708 GN=DARMORV10_C07P57070.1 PE=3 SV=1 | 0.002 | 1.352 | 2 | 1 | 3 | 1 | 561 | 61.3 | 5.35 | 0 | 1 | DARMORV10_C07P57070.1 | 0 | 0.01 | 1E-17 | 5.5977E-17 |
| A0A816NP02 | splicing factor 3B subunit 1-like, (rape) hypothetical protein OS=Brassica napus OX=3708 GN=DARMORV10_A09P08590.1 PE=3 SV=1 | 0 | 1.802 | 1 | 1 | 6 | 1 | 1268 | 141.1 | 5.99 | 76 | 1 | DARMORV10_A09P08590.1 | 0 | 0.01 | 1E-17 | 5.5977E-17 |
| A0A816NUK8 | (rape) hypothetical protein OS=Brassica napus OX=3708 GN=DARMORV10_A09P17610.1 PE=4 SV=1 | 0.004 | 1.197 | 5 | 1 | 2 | 1 | 250 | 28.7 | 6.52 | 0 | 1 | DARMORV10_A09P17610.1 | 0 | 0.01 | 1E-17 | 5.5977E-17 |
| A0A816NXT9 | Probable MOSC domain-containing protein (rape) hypothetical protein (Fragment) OS=Brassica napus OX=3708 GN=DARMORV10_A09P21270.1 PE=4 SV=1 | 0 | 4.481 | 6 | 3 | 22 | 2 | 674 | 76.7 | 7.21 | 163 | 3 | DARMORV10_A09P21270.1 | 0 | 0.064 | 0.01204156 | 0.056031821 |
| A0A816PCI0 | Dual specificity protein phosphatase 4 OS=Brassica napus OX=3708 GN=DARMORV10_A09P45250.1 PE=4 SV=1 | 0 | 3.965 | 4 | 2 | 20 | 1 | 760 | 84.8 | 6.73 | 65 | 2 | DARMORV10_A09P45250.1 | 0 | 0.01 | 1E-17 | 5.5977E-17 |
| A0A816PDC5 | Diaminopimelate epimerase OS=Brassica napus OX=3708 GN=DARMORV10_A09P46600.1 PE=3 SV=1 | 0 | 6.783 | 11 | 3 | 16 | 1 | 361 | 38.6 | 5.85 | 66 | 3 | DARMORV10_A09P46600.1 | 0 | 0.01 | 1E-17 | 5.5977E-17 |
| A0A816PL51 | Large ribosomal subunit protein uL4m OS=Brassica napus OX=3708 GN=DARMORV10_A09P59050.1 PE=3 SV=1 | 0 | 21.874 | 7 | 3 | 135 | 1 | 541 | 60.4 | 9.67 | 2163 | 3 | DARMORV10_A09P59050.1 | 3 | 0.033 | 0.001197139 | 0.00645289 |
| A0A816Q204 | receptor for activated C kinase 1B, (rape) hypothetical protein OS=Brassica napus OX=3708 GN=DARMORV10_C06P03270.1 PE=3 SV=1 | 0 | 5.464 | 7 | 2 | 27 | 1 | 329 | 35.7 | 6.64 | 98 | 2 | DARMORV10_C06P03270.1 | 0 | 0.01 | 1E-17 | 5.5977E-17 |
| A0A816Q7E3 | Dihydrolipoamide acetyltransferase component of pyruvate dehydrogenase complex OS=Brassica napus OX=3708 GN=DARMORV10_C06P11500.1 PE=3 SV=1 | 0 | 3.015 | 3 | 1 | 17 | 1 | 456 | 47.8 | 8.6 | 114 | 1 | DARMORV10_C06P11500.1 | 0 | 0.01 | 1E-17 | 5.5977E-17 |
| A0A816QFX6 | Ubiquitin-fold modifier 1 OS=Brassica napus OX=3708 GN=DARMORV10_A07P27870.1 PE=3 SV=1 | 0 | 5.832 | 31 | 1 | 12 | 1 | 90 | 9.6 | 9.31 | 28 | 1 | DARMORV10_A07P27870.1 | 0 | 0.01 | 1E-17 | 5.5977E-17 |
| A0A816QGY5 | Glycine-tRNA ligase OS=Brassica napus OX=3708 GN=DARMORV10_C06P32790.1 PE=3 SV=1 | 0 | 14.179 | 6 | 5 | 28 | 5 | 1044 | 116.9 | 6.01 | 101 | 5 | DARMORV10_C06P32790.1 | 0 | 0.01 | 1E-17 | 5.5977E-17 |
| A0A816QPB2 | probable choline kinase 1, (rape) hypothetical protein OS=Brassica napus OX=3708 GN=DARMORV10_C06P45740.1 PE=3 SV=1 | 0 | 3.54 | 3 | 1 | 13 | 1 | 346 | 40.3 | 4.96 | 328 | 1 | DARMORV10_C06P45740.1 | 0 | 0.01 | 1E-17 | 5.5977E-17 |
| A0A816QSX1 | WD repeat-containing protein DWA2, (rape) hypothetical protein OS=Brassica napus OX=3708 GN=DARMORV10_C06P50230.1 PE=4 SV=1 | 0 | 3.095 | 5 | 1 | 5 | 1 | 350 | 38.8 | 5.43 | 35 | 1 | DARMORV10_C06P50230.1 | 0 | 0.01 | 1E-17 | 5.5977E-17 |
| A0A816QU66 | EP1-like glycoprotein 2, (rape) hypothetical protein OS=Brassica napus OX=3708 GN=DARMORV10_C06P52730.1 PE=4 SV=1 | 0 | 2.287 | 6 | 2 | 2 | 1 | 454 | 50.2 | 8.16 | 0 | 2 | DARMORV10_C06P52730.1 | 0 | 0.01 | 1E-17 | 5.5977E-17 |
| A0A816QW44 | Plastocyanin OS=Brassica napus OX=3708 GN=DARMORV10_C06P50040.1 PE=3 SV=1 | 0 | 17.487 | 16 | 2 | 79 | 2 | 170 | 17.4 | 5.48 | 826 | 2 | DARMORV10_C06P50040.1 | 0 | 0.01 | 1E-17 | 5.5977E-17 |
| A0A816R3B4 | Adenylosuccinate lyase OS=Brassica napus OX=3708 GN=DARMORV10_C01P12350.1 PE=3 SV=1 | 0 | 13.388 | 4 | 2 | 28 | 2 | 855 | 95.9 | 6.15 | 900 | 2 | DARMORV10_C01P12350.1 | 0 | 0.01 | 1E-17 | 5.5977E-17 |
| A0A816R4J7 | alpha-aminoadipic semialdehyde synthase, (rape) hypothetical protein OS=Brassica napus OX=3708 GN=DARMORV10_C01P05470.1 PE=3 SV=1 | 0 | 1.869 | 1 | 1 | 8 | 1 | 1060 | 117 | 5.86 | 43 | 1 | DARMORV10_C01P05470.1 | 0 | 0.01 | 1E-17 | 5.5977E-17 |
| A0A816R4K1 | ectonucleotide pyrophosphatase/phosphodiesterase family member 3, (rape) hypothetical protein OS=Brassica napus OX=3708 GN=DARMORV10_C01P09770.1 PE=4 SV=1 | 0 | 3.982 | 3 | 1 | 9 | 1 | 459 | 51.6 | 8.16 | 29 | 1 | DARMORV10_C01P09770.1 | 0 | 0.01 | 1E-17 | 5.5977E-17 |
| A0A816R4K3 | Catalase OS=Brassica napus OX=3708 GN=DARMORV10_C01P03770.1 PE=3 SV=1 | 0 | 55.252 | 38 | 13 | 245 | 1 | 480 | 55.4 | 6.83 | 2455 | 13 | DARMORV10_C01P03770.1 | 0 | 0.01 | 1E-17 | 5.5977E-17 |
| A0A816R5V7 | Probable Arf-GAP domain-containing protein (rape) hypothetical protein OS=Brassica napus OX=3708 GN=DARMORV10_C01P11720.1 PE=4 SV=1 | 0 | 1.774 | 3 | 1 | 3 | 1 | 426 | 46 | 8.24 | 47 | 1 | DARMORV10_C01P11720.1 | 0 | 0.01 | 1E-17 | 5.5977E-17 |
| A0A816RAG3 | thylakoid lumenal 17.9 kDa protein, chloroplastic, (rape) hypothetical protein OS=Brassica napus OX=3708 GN=DARMORV10_C01P20210.1 PE=4 SV=1 | 0 | 3.194 | 6 | 1 | 2 | 1 | 245 | 26.7 | 8.03 | 45 | 1 | DARMORV10_C01P20210.1 | 0 | 0.01 | 1E-17 | 5.5977E-17 |
| A0A816RG60 | (rape) hypothetical protein OS=Brassica napus OX=3708 GN=DARMORV10_C01P23680.1 PE=3 SV=1 | 0 | 2.744 | 1 | 1 | 15 | 1 | 819 | 92.3 | 9.44 | 304 | 1 | DARMORV10_C01P23680.1 | 0 | 0.01 | 1E-17 | 5.5977E-17 |
| A0A816RJI3 | Probable SAM domain-containing protein (rape) hypothetical protein OS=Brassica napus OX=3708 GN=DARMORV10_C01P31480.1 PE=4 SV=1 | 0.005 | 1.184 | 4 | 1 | 5 | 1 | 274 | 30.3 | 5.86 | 0 | 1 | DARMORV10_C01P31480.1 | 0 | 0.01 | 1E-17 | 5.5977E-17 |
| A0A816RLF7 | RING-type E3 ubiquitin transferase OS=Brassica napus OX=3708 GN=DARMORV10_C01P33340.1 PE=4 SV=1 | 0.007 | 1.103 | 3 | 1 | 2 | 1 | 661 | 71.7 | 5.24 | 0 | 1 | DARMORV10_C01P33340.1 | 0 | 0.01 | 1E-17 | 5.5977E-17 |
| A0A816RME9 | Non-specific serine/threonine protein kinase OS=Brassica napus OX=3708 GN=DARMORV10_C01P43710.1 PE=3 SV=1 | 0 | 4.82 | 5 | 2 | 20 | 1 | 548 | 62.1 | 8.82 | 440 | 2 | DARMORV10_C01P43710.1 | 0 | 0.01 | 1E-17 | 5.5977E-17 |
| A0A816RXC9 | Probable Gamma-aminobutyric acid transaminase, (rape) hypothetical protein OS=Brassica napus OX=3708 GN=DARMORV10_C01P42030.1 PE=3 SV=1 | 0 | 9.284 | 11 | 4 | 26 | 3 | 560 | 62.3 | 8.79 | 50 | 4 | DARMORV10_C01P42030.1 | 0 | 0.01 | 1E-17 | 5.5977E-17 |
| A0A816RXT9 | Probable Serine aminopeptidase S33 domain-containing protein, (rape) hypothetical protein (Fragment) OS=Brassica napus OX=3708 GN=DARMORV10_A06P01640.1 PE=4 SV=1 | 0 | 2.886 | 4 | 1 | 9 | 1 | 366 | 41.2 | 7.94 | 84 | 1 | DARMORV10_A06P01640.1 | 0 | 0.01 | 1E-17 | 5.5977E-17 |
| A0A816S5P1 | Prolyl endopeptidase OS=Brassica napus OX=3708 GN=DARMORV10_A06P03180.1 PE=3 SV=1 | 0 | 5.075 | 5 | 3 | 14 | 1 | 769 | 86.9 | 6.58 | 52 | 3 | DARMORV10_A06P03180.1 | 0 | 0.01 | 1E-17 | 5.5977E-17 |
| A0A816SAR4 | scarecrow-like protein 5, (rape) hypothetical protein OS=Brassica napus OX=3708 GN=DARMORV10_A06P03020.1 PE=3 SV=1 | 0 | 1.518 | 2 | 1 | 4 | 1 | 526 | 58.5 | 6.19 | 44 | 1 | DARMORV10_A06P03020.1 | 0 | 0.01 | 1E-17 | 5.5977E-17 |
| A0A816SMV0 | Probable Beta-glucosidase, (rape) hypothetical protein OS=Brassica napus OX=3708 GN=DARMORV10_A06P20480.1 PE=3 SV=1 | 0 | 2.007 | 1 | 1 | 1 | 1 | 1210 | 133.1 | 5.33 | 43 | 1 | DARMORV10_A06P20480.1 | 0 | 0.01 | 1E-17 | 5.5977E-17 |
| A0A816SPT8 | Oligopeptidase A OS=Brassica napus OX=3708 GN=DARMORV10_A06P32460.1 PE=3 SV=1 | 0 | 6.316 | 4 | 3 | 14 | 2 | 823 | 91.9 | 6.43 | 161 | 3 | DARMORV10_A06P32460.1 | 0 | 0.01 | 1E-17 | 5.5977E-17 |
| A0A816SRJ3 | Asparagine synthetase [glutamine-hydrolyzing] OS=Brassica napus OX=3708 GN=DARMORV10_A06P27160.1 PE=4 SV=1 | 0 | 4.171 | 3 | 1 | 13 | 1 | 613 | 69.1 | 6.51 | 313 | 1 | DARMORV10_A06P27160.1 | 0 | 0.01 | 1E-17 | 5.5977E-17 |
| A0A816SW15 | Probable Metalloenzyme domain-containing protein, (rape) hypothetical protein OS=Brassica napus OX=3708 GN=DARMORV10_A06P39620.1 PE=3 SV=1 | 0 | 4.524 | 8 | 3 | 15 | 1 | 505 | 54 | 5.36 | 37 | 3 | DARMORV10_A06P39620.1 | 0 | 0.01 | 1E-17 | 5.5977E-17 |
| A0A816SXD7 | Ribose-phosphate diphosphokinase OS=Brassica napus OX=3708 GN=DARMORV10_A05P03440.1 PE=3 SV=1 | 0 | 5.024 | 11 | 2 | 9 | 1 | 343 | 38.2 | 6.38 | 62 | 2 | DARMORV10_A05P03440.1 | 0 | 0.01 | 1E-17 | 5.5977E-17 |
| A0A816SYL0 | beta-D-xylosidase 1, (rape) hypothetical protein OS=Brassica napus OX=3708 GN=DARMORV10_A06P38380.1 PE=3 SV=1 | 0 | 72.332 | 21 | 12 | 207 | 6 | 772 | 83.5 | 8.87 | 3950 | 12 | DARMORV10_A06P38380.1 | 9 | 0.069 | 0.015757248 | 0.07126347 |
| A0A816T135 | Probable NAD(P)-binding domain-containing protein (rape) hypothetical protein OS=Brassica napus OX=3708 GN=DARMORV10_A05P08310.1 PE=3 SV=1 | 0 | 12.784 | 15 | 3 | 42 | 2 | 322 | 34.6 | 7.24 | 592 | 3 | DARMORV10_A05P08310.1 | 0 | 0.063 | 0.01143063 | 0.053467702 |
| A0A816TCN8 | vacuolar protein-sorting-associated protein 37 homolog 2, (rape) hypothetical protein OS=Brassica napus OX=3708 GN=DARMORV10_A05P09200.1 PE=3 SV=1 | 0.001 | 1.402 | 3 | 1 | 4 | 1 | 252 | 28.7 | 7.39 | 29 | 1 | DARMORV10_A05P09200.1 | 0 | 0.01 | 1E-17 | 5.5977E-17 |
| A0A816TP41 | Histone acetyltransferase OS=Brassica napus OX=3708 GN=DARMORV10_A05P25290.1 PE=3 SV=1 | 0 | 2.21 | 2 | 1 | 3 | 1 | 743 | 79.8 | 8.59 | 22 | 1 | DARMORV10_A05P25290.1 | 0 | 0.01 | 1E-17 | 5.5977E-17 |
| A0A816TUJ8 | heme-binding-like protein At3g10130, chloroplastic, (rape) hypothetical protein OS=Brassica napus OX=3708 GN=DARMORV10_A05P38960.1 PE=3 SV=1 | 0.008 | 1.065 | 4 | 1 | 2 | 1 | 328 | 37.1 | 9.29 | 30 | 1 | DARMORV10_A05P38960.1 | 0 | 0.01 | 1E-17 | 5.5977E-17 |
| A0A816TYJ5 | Probable C2 NT-type domain-containing protein, (rape) hypothetical protein OS=Brassica napus OX=3708 GN=DARMORV10_C08P09250.1 PE=4 SV=1 | 0 | 17.148 | 7 | 4 | 34 | 4 | 842 | 93.4 | 5.2 | 261 | 4 | DARMORV10_C08P09250.1 | 0 | 0.01 | 1E-17 | 5.5977E-17 |
| A0A816U1P6 | Inositol-1-monophosphatase OS=Brassica napus OX=3708 GN=DARMORV10_A05P44240.1 PE=3 SV=1 | 0 | 4.86 | 7 | 1 | 7 | 1 | 300 | 32.3 | 5.5 | 54 | 1 | DARMORV10_A05P44240.1 | 0 | 0.01 | 1E-17 | 5.5977E-17 |
| A0A816U1R5 | Probable ABC transporter domain-containing protein (rape) hypothetical protein OS=Brassica napus OX=3708 GN=DARMORV10_C08P00830.1 PE=3 SV=1 | 0 | 1.636 | 1 | 1 | 2 | 1 | 586 | 65.6 | 7.91 | 26 | 1 | DARMORV10_C08P00830.1 | 0 | 0.01 | 1E-17 | 5.5977E-17 |
| A0A816U3Z1 | Probable Apple domain-containing protein, (rape) hypothetical protein OS=Brassica napus OX=3708 GN=DARMORV10_C08P17640.1 PE=4 SV=1 | 0 | 1.511 | 10 | 1 | 1 | 1 | 200 | 22.2 | 8.56 | 0 | 1 | DARMORV10_C08P17640.1 | 0 | 0.01 | 1E-17 | 5.5977E-17 |
| A0A816U4A0 | PITH domain-containing protein At3g04780-like, (rape) hypothetical protein OS=Brassica napus OX=3708 GN=DARMORV10_A05P42780.1 PE=3 SV=1 | 0 | 3.507 | 10 | 1 | 5 | 1 | 176 | 19.6 | 4.96 | 107 | 1 | DARMORV10_A05P42780.1 | 0 | 0.01 | 1E-17 | 5.5977E-17 |
| A0A816UGE8 | Guanine nucleotide-binding protein alpha subunit OS=Brassica napus OX=3708 GN=DARMORV10_C08P38230.1 PE=3 SV=1 | 0 | 1.563 | 2 | 1 | 1 | 1 | 420 | 48.9 | 6.84 | 0 | 1 | DARMORV10_C08P38230.1 | 0 | 0.01 | 1E-17 | 5.5977E-17 |
| A0A816UHF7 | Cyclin-dependent kinase OS=Brassica napus OX=3708 GN=DARMORV10_C08P23780.1 PE=3 SV=1 | 0.002 | 1.311 | 3 | 1 | 6 | 1 | 435 | 50.6 | 9.07 | 22 | 1 | DARMORV10_C08P23780.1 | 0 | 0.01 | 1E-17 | 5.5977E-17 |
| A0A816ULC5 | Acetylglutamate kinase OS=Brassica napus OX=3708 GN=DARMORV10_C08P33110.1 PE=3 SV=1 | 0.006 | 1.169 | 4 | 1 | 3 | 1 | 345 | 36.3 | 8.5 | 0 | 1 | DARMORV10_C08P33110.1 | 0 | 0.01 | 1E-17 | 5.5977E-17 |
| A0A816ULE3 | Adenylosuccinate synthetase, chloroplastic OS=Brassica napus OX=3708 GN=PURA PE=3 SV=1 | 0 | 18.209 | 16 | 5 | 51 | 2 | 491 | 53.2 | 6.7 | 491 | 5 | PURA | 0 | 0.049 | 0.006253257 | 0.030676461 |
| A0A816ULY3 | Probable HTH cro/C1-type domain-containing protein, (rape) hypothetical protein OS=Brassica napus OX=3708 GN=DARMORV10_C08P34010.1 PE=3 SV=1 | 0 | 3.517 | 3 | 1 | 6 | 1 | 630 | 69.6 | 9.31 | 72 | 1 | DARMORV10_C08P34010.1 | 0 | 0.01 | 1E-17 | 5.5977E-17 |
| A0A816UMB6 | Non-specific serine/threonine protein kinase OS=Brassica napus OX=3708 GN=DARMORV10_C08P37950.1 PE=4 SV=1 | 0 | 2.97 | 2 | 1 | 8 | 1 | 720 | 78.2 | 6.14 | 26 | 1 | DARMORV10_C08P37950.1 | 0 | 0.01 | 1E-17 | 5.5977E-17 |
| A0A816UPL9 | chaperone protein ClpC2, chloroplastic, (rape) hypothetical protein OS=Brassica napus OX=3708 GN=DARMORV10_C08P23840.1 PE=3 SV=1 | 0 | 111.526 | 32 | 28 | 403 | 2 | 932 | 103.4 | 6.42 | 3987 | 28 | DARMORV10_C08P23840.1 | 0 | 0.01 | 1E-17 | 5.5977E-17 |
| A0A816UQ20 | Glutathione reductase OS=Brassica napus OX=3708 GN=DARMORV10_C08P29810.1 PE=3 SV=1 | 0 | 10.967 | 12 | 4 | 26 | 1 | 561 | 60.5 | 8.28 | 108 | 4 | DARMORV10_C08P29810.1 | 0 | 0.01 | 1E-17 | 5.5977E-17 |
| A0A816UW50 | Pectate lyase OS=Brassica napus OX=3708 GN=DARMORV10_C08P45750.1 PE=3 SV=1 | 0 | 4.905 | 3 | 1 | 11 | 1 | 661 | 74.3 | 7.18 | 38 | 1 | DARMORV10_C08P45750.1 | 0 | 0.01 | 1E-17 | 5.5977E-17 |
| A0A816UX43 | Phenylalanine-tRNA ligase OS=Brassica napus OX=3708 GN=DARMORV10_C08P41200.1 PE=3 SV=1 | 0.003 | 1.301 | 2 | 1 | 1 | 1 | 453 | 51.9 | 7.36 | 20 | 1 | DARMORV10_C08P41200.1 | 0 | 0.01 | 1E-17 | 5.5977E-17 |
| A0A816UYL5 | stem-specific protein TSJT1, (rape) hypothetical protein OS=Brassica napus OX=3708 GN=DARMORV10_A03P09370.1 PE=4 SV=1 | 0 | 3.25 | 5 | 1 | 11 | 1 | 251 | 27.3 | 6.9 | 207 | 1 | DARMORV10_A03P09370.1 | 0 | 0.01 | 1E-17 | 5.5977E-17 |
| A0A816UZ41 | Probable TauD/TfdA-like domain-containing protein, (rape) hypothetical protein OS=Brassica napus OX=3708 GN=DARMORV10_A03P09860.1 PE=4 SV=1 | 0 | 3.744 | 4 | 1 | 14 | 1 | 392 | 45.1 | 6.67 | 132 | 1 | DARMORV10_A03P09860.1 | 0 | 0.01 | 1E-17 | 5.5977E-17 |
| A0A816V877 | chloroplastic import inner membrane translocase subunit HP30-1, (rape) hypothetical protein OS=Brassica napus OX=3708 GN=DARMORV10_C08P24540.1 PE=3 SV=1 | 0 | 2.591 | 5 | 1 | 12 | 1 | 266 | 28.6 | 9.28 | 79 | 1 | DARMORV10_C08P24540.1 | 0 | 0.01 | 1E-17 | 5.5977E-17 |
| A0A816VAQ7 | transport inhibitor response 1-like protein , (rape) hypothetical protein OS=Brassica napus OX=3708 GN=DARMORV10_A03P27600.1 PE=4 SV=1 | 0 | 2.729 | 2 | 1 | 4 | 1 | 646 | 72.5 | 6.37 | 55 | 1 | DARMORV10_A03P27600.1 | 0 | 0.01 | 1E-17 | 5.5977E-17 |
| A0A816VAT7 | (rape) hypothetical protein OS=Brassica napus OX=3708 GN=DARMORV10_A03P16060.1 PE=4 SV=1 | 0.006 | 1.16 | 1 | 1 | 6 | 1 | 1272 | 138 | 6.06 | 29 | 1 | DARMORV10_A03P16060.1 | 0 | 0.01 | 1E-17 | 5.5977E-17 |
| A0A816VBP6 | Diphosphomevalonate decarboxylase OS=Brassica napus OX=3708 GN=DARMORV10_C08P29490.1 PE=3 SV=1 | 0 | 2.109 | 2 | 1 | 3 | 1 | 458 | 50.5 | 6.29 | 0 | 1 | DARMORV10_C08P29490.1 | 0 | 0.01 | 1E-17 | 5.5977E-17 |
| A0A816VIK8 | Probable F-box domain-containing protein (rape) hypothetical protein OS=Brassica napus OX=3708 GN=DARMORV10_A03P40440.1 PE=4 SV=1 | 0 | 1.537 | 1 | 1 | 8 | 1 | 1192 | 132.7 | 6.61 | 87 | 1 | DARMORV10_A03P40440.1 | 0 | 0.01 | 1E-17 | 5.5977E-17 |
| A0A816VY53 | Succinate--CoA ligase [ADP-forming] subunit alpha, mitochondrial OS=Brassica napus OX=3708 GN=DARMORV10_A03P46310.1 PE=3 SV=1 | 0 | 21.044 | 22 | 5 | 38 | 2 | 346 | 35.9 | 8.76 | 688 | 5 | DARMORV10_A03P46310.1 | 0 | 0.01 | 1E-17 | 5.5977E-17 |
| A0A816W090 | Protein-serine/threonine kinase (Fragment) OS=Brassica napus OX=3708 GN=DARMORV10_A03P34710.1 PE=3 SV=1 | 0.007 | 1.088 | 2 | 1 | 6 | 1 | 439 | 50 | 8.12 | 30 | 1 | DARMORV10_A03P34710.1 | 0 | 0.01 | 1E-17 | 5.5977E-17 |
| A0A816W1Z3 | DNA gyrase subunit A, chloroplastic/mitochondrial OS=Brassica napus OX=3708 GN=DARMORV10_A03P36700.1 PE=3 SV=1 | 0 | 3.741 | 3 | 2 | 5 | 2 | 940 | 103.1 | 7.23 | 16 | 2 | DARMORV10_A03P36700.1 | 0 | 0.01 | 1E-17 | 5.5977E-17 |
| A0A816WCD4 | ATP-dependent Clp protease ATP-binding subunit CLPT1, chloroplastic-like, (rape) hypothetical protein (Fragment) OS=Brassica napus OX=3708 GN=DARMORV10_A03P55940.1 PE=4 SV=1 | 0 | 2.997 | 9 | 1 | 4 | 1 | 252 | 27.4 | 9.61 | 42 | 1 | DARMORV10_A03P55940.1 | 0 | 0.01 | 1E-17 | 5.5977E-17 |
| A0A816WDI1 | Sm protein F OS=Brassica napus OX=3708 GN=DARMORV10_A03P59660.1 PE=3 SV=1 | 0.009 | 1.042 | 11 | 1 | 2 | 1 | 150 | 17 | 7.33 | 14 | 1 | DARMORV10_A03P59660.1 | 0 | 0.01 | 1E-17 | 5.5977E-17 |
| A0A816WDV3 | COP9 signalosome complex subunit 2 OS=Brassica napus OX=3708 GN=DARMORV10_A03P26810.1 PE=3 SV=1 | 0 | 2.428 | 2 | 1 | 3 | 1 | 871 | 99.5 | 5.49 | 27 | 1 | DARMORV10_A03P26810.1 | 0 | 0.01 | 1E-17 | 5.5977E-17 |
| A0A816WFF6 | plant UBX domain-containing protein 10-like, (rape) hypothetical protein OS=Brassica napus OX=3708 GN=DARMORV10_A03P29290.1 PE=4 SV=1 | 0 | 2.128 | 2 | 1 | 12 | 1 | 479 | 53.1 | 5 | 118 | 1 | DARMORV10_A03P29290.1 | 0 | 0.01 | 1E-17 | 5.5977E-17 |
| A0A816WJK6 | nascent polypeptide-associated complex subunit alpha-like protein 1, (rape) hypothetical protein OS=Brassica napus OX=3708 GN=DARMORV10_A03P37610.1 PE=4 SV=1 | 0 | 15.799 | 20 | 3 | 48 | 2 | 201 | 21.7 | 4.54 | 1073 | 3 | DARMORV10_A03P37610.1 | 0 | 0.074 | 0.019562075 | 0.086857885 |
| A0A816X2K7 | Exocyst complex component OS=Brassica napus OX=3708 GN=DARMORV10_A02P24620.1 PE=3 SV=1 | 0 | 2.486 | 2 | 1 | 4 | 1 | 795 | 89.9 | 6.38 | 66 | 1 | DARMORV10_A02P24620.1 | 0 | 0.01 | 1E-17 | 5.5977E-17 |
| A0A816XC64 | embryo-specific protein ATS3B-like, (rape) hypothetical protein OS=Brassica napus OX=3708 GN=DARMORV10_A02P42180.1 PE=4 SV=1 | 0 | 4.347 | 10 | 1 | 15 | 1 | 185 | 20.4 | 6.49 | 66 | 1 | DARMORV10_A02P42180.1 | 0 | 0.01 | 1E-17 | 5.5977E-17 |
| A0A816XIR4 | (rape) hypothetical protein OS=Brassica napus OX=3708 GN=DARMORV10_A01P06180.1 PE=3 SV=1 | 0 | 2.827 | 2 | 1 | 5 | 1 | 588 | 66.7 | 8.75 | 22 | 1 | DARMORV10_A01P06180.1 | 0 | 0.01 | 1E-17 | 5.5977E-17 |
| A0A816XKQ3 | Probable DUF642 domain-containing protein, (rape) hypothetical protein OS=Brassica napus OX=3708 GN=DARMORV10_A01P05910.1 PE=4 SV=1 | 0 | 6.829 | 11 | 4 | 27 | 2 | 365 | 39.8 | 8.46 | 165 | 4 | DARMORV10_A01P05910.1 | 0 | 0.01 | 1E-17 | 5.5977E-17 |
| A0A816XM00 | 11S globulin OS=Brassica napus OX=3708 GN=DARMORV10_A01P09420.1 PE=3 SV=1 | 0 | 12.064 | 7 | 3 | 49 | 1 | 509 | 56.5 | 7.97 | 570 | 3 | DARMORV10_A01P09420.1 | 0 | 0.015 | 4.11759E-05 | 0.00022995 |
| A0A816XSM9 | Uncharacterized protein (rape) hypothetical protein OS=Brassica napus OX=3708 GN=DARMORV10_A01P18990.1 PE=4 SV=1 | 0.006 | 1.135 | 3 | 1 | 4 | 1 | 381 | 41.5 | 9.35 | 23 | 1 | DARMORV10_A01P18990.1 | 0 | 0.01 | 1E-17 | 5.5977E-17 |
| A0A816XWQ7 | Probable Imidazole glycerol-phosphate synthase, (rape) hypothetical protein OS=Brassica napus OX=3708 GN=DARMORV10_A01P18110.1 PE=3 SV=1 | 0 | 19.622 | 24 | 8 | 42 | 1 | 431 | 47.2 | 6.34 | 212 | 8 | DARMORV10_A01P18110.1 | 0 | 0.01 | 1E-17 | 5.5977E-17 |
| A0A816Y1W8 | Probable 6-phosphogluconolactonase OS=Brassica napus OX=3708 GN=DARMORV10_A01P24800.1 PE=3 SV=1 | 0.008 | 1.067 | 3 | 1 | 3 | 1 | 258 | 28.8 | 6.4 | 22 | 1 | DARMORV10_A01P24800.1 | 0 | 0.01 | 1E-17 | 5.5977E-17 |
| A0A816Y294 | (rape) hypothetical protein OS=Brassica napus OX=3708 GN=DARMORV10_A01P27560.1 PE=4 SV=1 | 0 | 3.149 | 9 | 1 | 6 | 1 | 231 | 26.3 | 9.45 | 209 | 1 | DARMORV10_A01P27560.1 | 0 | 0.01 | 1E-17 | 5.5977E-17 |
| A0A816Y6T0 | (rape) hypothetical protein OS=Brassica napus OX=3708 GN=DARMORV10_A01P38740.1 PE=4 SV=1 | 0.007 | 1.094 | 3 | 1 | 1 | 1 | 827 | 87.9 | 7.23 | 0 | 1 | DARMORV10_A01P38740.1 | 0 | 0.01 | 1E-17 | 5.5977E-17 |
| A0A816YPV8 | CCR4-NOT transcription complex subunit 1, (rape) hypothetical protein OS=Brassica napus OX=3708 GN=DARMORV10_A07P21180.1 PE=4 SV=1 | 0 | 8.323 | 2 | 4 | 22 | 1 | 2378 | 263.5 | 5.63 | 201 | 4 | DARMORV10_A07P21180.1 | 0 | 0.01 | 1E-17 | 5.5977E-17 |
| A0A816YTP4 | GDP-mannose 4,6-dehydratase OS=Brassica napus OX=3708 GN=DARMORV10_A07P17560.1 PE=3 SV=1 | 0 | 8.064 | 10 | 2 | 10 | 1 | 368 | 41.6 | 6.67 | 52 | 2 | DARMORV10_A07P17560.1 | 0 | 0.01 | 1E-17 | 5.5977E-17 |
| A0A816YVR1 | PRA1 family protein OS=Brassica napus OX=3708 GN=DARMORV10_A07P22750.1 PE=3 SV=1 | 0 | 1.933 | 7 | 1 | 1 | 1 | 211 | 22.7 | 8.92 | 0 | 1 | DARMORV10_A07P22750.1 | 0 | 0.01 | 1E-17 | 5.5977E-17 |
| A0A816YZ70 | Glucan endo-1,3-beta-D-glucosidase OS=Brassica napus OX=3708 GN=DARMORV10_A07P21140.1 PE=3 SV=1 | 0 | 3.411 | 4 | 1 | 15 | 1 | 428 | 45.5 | 6.99 | 310 | 1 | DARMORV10_A07P21140.1 | 0 | 0.01 | 1E-17 | 5.5977E-17 |
| A0A816Z3M4 | Probable Negatively light-regulated protein (rape) hypothetical protein OS=Brassica napus OX=3708 GN=DARMORV10_A07P35810.1 PE=3 SV=1 | 0 | 2.569 | 8 | 1 | 10 | 1 | 156 | 17 | 5.22 | 72 | 1 | DARMORV10_A07P35810.1 | 0 | 0.01 | 1E-17 | 5.5977E-17 |
| A0A816Z4P8 | Phosphoglycerate kinase OS=Brassica napus OX=3708 GN=DARMORV10_A07P27080.1 PE=3 SV=1 | 0 | 59.512 | 42 | 14 | 219 | 1 | 401 | 42.3 | 6 | 3950 | 14 | DARMORV10_A07P27080.1 | 0 | 0.01 | 1E-17 | 5.5977E-17 |
| A0A816ZAB2 | alpha-Amylase OS=Brassica napus OX=3708 GN=DARMORV10_A07P36260.1 PE=3 SV=1 | 0 | 4.405 | 4 | 3 | 16 | 3 | 900 | 100.7 | 5.94 | 37 | 3 | DARMORV10_A07P36260.1 | 0 | 0.01 | 1E-17 | 5.5977E-17 |
| A0A816ZAE6 | (rape) hypothetical protein OS=Brassica napus OX=3708 GN=DARMORV10_A07P37600.1 PE=3 SV=1 | 0 | 1.693 | 1 | 1 | 3 | 1 | 1228 | 137.4 | 8.97 | 0 | 1 | DARMORV10_A07P37600.1 | 0 | 0.01 | 1E-17 | 5.5977E-17 |
| A0A816ZBX6 | Non-specific serine/threonine protein kinase OS=Brassica napus OX=3708 GN=DARMORV10_A07P41530.1 PE=3 SV=1 | 0.002 | 1.34 | 3 | 1 | 1 | 1 | 555 | 63 | 7.58 | 0 | 1 | DARMORV10_A07P41530.1 | 0 | 0.01 | 1E-17 | 5.5977E-17 |
| A0A816ZC43 | Plastocyanin OS=Brassica napus OX=3708 GN=DARMORV10_A07P41660.1 PE=3 SV=1 | 0 | 2.585 | 18 | 1 | 5 | 1 | 170 | 17.4 | 5.22 | 32 | 1 | DARMORV10_A07P41660.1 | 0 | 0.01 | 1E-17 | 5.5977E-17 |
| A0A816ZCQ1 | Lactoylglutathione lyase OS=Brassica napus OX=3708 GN=DARMORV10_A07P33890.1 PE=3 SV=1 | 0 | 25.265 | 22 | 6 | 84 | 6 | 341 | 37.9 | 6.92 | 1453 | 6 | DARMORV10_A07P33890.1 | 0 | 0.092 | 0.035447756 | 0.142857725 |
| A0A816ZFK7 | trihelix transcription factor GT-2, (rape) hypothetical protein OS=Brassica napus OX=3708 GN=DARMORV10_A07P42480.1 PE=4 SV=1 | 0 | 1.714 | 2 | 1 | 3 | 1 | 575 | 65.4 | 7.61 | 23 | 1 | DARMORV10_A07P42480.1 | 0 | 0.01 | 1E-17 | 5.5977E-17 |
| A0A816ZIL8 | (rape) hypothetical protein OS=Brassica napus OX=3708 GN=DARMORV10_A07P44620.1 PE=3 SV=1 | 0 | 1.623 | 2 | 1 | 5 | 1 | 527 | 59.2 | 9.16 | 0 | 1 | DARMORV10_A07P44620.1 | 0 | 0.01 | 1E-17 | 5.5977E-17 |
| A0A817A5K5 | Valine-tRNA ligase OS=Brassica napus OX=3708 GN=DARMORV10_A08P27330.1 PE=3 SV=1 | 0 | 3.348 | 2 | 1 | 5 | 1 | 1059 | 120.6 | 6.24 | 0 | 1 | DARMORV10_A08P27330.1 | 0 | 0.01 | 1E-17 | 5.5977E-17 |
| A0A817AGF7 | V-type proton ATPase subunit C OS=Brassica napus OX=3708 GN=DARMORV10_A08P32590.1 PE=3 SV=1 | 0 | 13.051 | 21 | 6 | 38 | 1 | 394 | 45.1 | 6.37 | 444 | 6 | DARMORV10_A08P32590.1 | 0 | 0.01 | 1E-17 | 5.5977E-17 |
| A0A817AI73 | cysteine protease XCP2, (rape) hypothetical protein OS=Brassica napus OX=3708 GN=DARMORV10_A08P29200.1 PE=3 SV=1 | 0 | 2.968 | 4 | 1 | 13 | 1 | 359 | 40 | 5.35 | 121 | 1 | DARMORV10_A08P29200.1 | 0 | 0.01 | 1E-17 | 5.5977E-17 |
| A0A817AKH6 | Glyceraldehyde-3-phosphate dehydrogenase (phosphorylating) OS=Brassica napus OX=3708 GN=DARMORV10_A08P32290.1 PE=3 SV=1 | 0 | 62.684 | 26 | 14 | 270 | 2 | 656 | 73.6 | 6.68 | 5256 | 14 | DARMORV10_A08P32290.1 | 16 | 0.043 | 0.003295344 | 0.016937548 |
| A0A817APY9 | Glutaredoxin-dependent peroxiredoxin OS=Brassica napus OX=3708 GN=DARMORV10_A04P06550.1 PE=3 SV=1 | 0 | 27.25 | 35 | 6 | 73 | 1 | 229 | 24.2 | 8.78 | 1359 | 6 | DARMORV10_A04P06550.1 | 0 | 0.01 | 1E-17 | 5.5977E-17 |
| A0A817AWD8 | S-adenosylmethionine synthase OS=Brassica napus OX=3708 GN=DARMORV10_A04P28170.1 PE=3 SV=1 | 0 | 35.361 | 31 | 9 | 88 | 2 | 390 | 42.5 | 6.54 | 1655 | 9 | DARMORV10_A04P28170.1 | 0 | 0.01 | 1E-17 | 5.5977E-17 |
| A0A817AY91 | probable mitochondrial saccharopine dehydrogenase-like oxidoreductase At5g39410, (rape) hypothetical protein OS=Brassica napus OX=3708 GN=DARMORV10_A04P12100.1 PE=3 SV=1 | 0 | 2.562 | 4 | 1 | 5 | 1 | 456 | 50 | 8.65 | 22 | 1 | DARMORV10_A04P12100.1 | 0 | 0.01 | 1E-17 | 5.5977E-17 |
| A0A817B1J5 | Ptobable SHSP domain-containing protein (rape) hypothetical protein OS=Brassica napus OX=3708 GN=DARMORV10_A10P05120.1 PE=3 SV=1 | 0 | 2.618 | 4 | 1 | 5 | 1 | 292 | 32 | 8.28 | 73 | 1 | DARMORV10_A10P05120.1 | 0 | 0.01 | 1E-17 | 5.5977E-17 |
| A0A817B7F2 | 3-Hydroxyisobutyryl-CoA hydrolase OS=Brassica napus OX=3708 GN=DARMORV10_A04P22280.1 PE=3 SV=1 | 0 | 2.553 | 4 | 1 | 8 | 1 | 427 | 47.6 | 8.43 | 49 | 1 | DARMORV10_A04P22280.1 | 0 | 0.01 | 1E-17 | 5.5977E-17 |
| A0A817BBT4 | Protein REDUCED CHLOROPLAST COVERAGE 1, (rape) hypothetical protein OS=Brassica napus OX=3708 GN=DARMORV10_A10P00600.1 PE=4 SV=1 | 0 | 11.484 | 3 | 4 | 20 | 4 | 1776 | 195.4 | 6.28 | 413 | 4 | DARMORV10_A10P00600.1 | 0 | 0.05 | 0.005421869 | 0.026843763 |
| A0A817BF91 | Heparanase-like protein 1, (rape) hypothetical protein OS=Brassica napus OX=3708 GN=DARMORV10_A10P28690.1 PE=3 SV=1 | 0 | 7.713 | 2 | 1 | 23 | 1 | 570 | 63.6 | 8.68 | 353 | 1 | DARMORV10_A10P28690.1 | 0 | 0.01 | 1E-17 | 5.5977E-17 |
| A0A817BLH5 | Nascent polypeptide-associated complex subunit alpha-like protein 3, (rape) hypothetical protein (Fragment) OS=Brassica napus OX=3708 GN=DARMORV10_A10P24500.1 PE=4 SV=1 | 0 | 9.551 | 12 | 3 | 38 | 1 | 238 | 25.7 | 4.65 | 509 | 3 | DARMORV10_A10P24500.1 | 0 | 0.01 | 1E-17 | 5.5977E-17 |
| A0ABQ7WY43 | Probable thioglucosidase, OS=Brassica napus OX=3708 GN=HID58_091934 PE=4 SV=1 | 0 | 25.371 | 8 | 4 | 166 | 2 | 526 | 60.2 | 7.31 | 1802 | 4 | HID58_091934 | 0 | 0.083 | 0.026713804 | 0.111823286 |
| A0ABQ7WYF3 | Uncharacterized protein OS=Brassica napus OX=3708 GN=HID58_091849 PE=4 SV=1 | 0 | 23.797 | 9 | 6 | 52 | 2 | 607 | 65.2 | 6.58 | 663 | 6 | HID58_091849 | 0 | 0.01 | 1E-17 | 5.5977E-17 |
| A0ABQ7X000 | Probable Phospholipase A-2-activating protein OS=Brassica napus OX=3708 GN=HID58_092025 PE=4 SV=1 | 0 | 15.218 | 11 | 5 | 41 | 1 | 770 | 85.2 | 5.86 | 453 | 5 | HID58_092025 | 0 | 0.01 | 1E-17 | 5.5977E-17 |
| A0ABQ7X1J6 | Uncharacterized protein OS=Brassica napus OX=3708 GN=HID58_096058 PE=4 SV=1 | 0 | 14.141 | 11 | 6 | 27 | 1 | 905 | 101.6 | 6.2 | 154 | 6 | HID58_096058 | 0 | 0.01 | 1E-17 | 5.5977E-17 |
| A0ABQ7X6P1 | Uncharacterized protein OS=Brassica napus OX=3708 GN=HID58_094631 PE=4 SV=1 | 0 | 1.662 | 0 | 1 | 1 | 1 | 2264 | 249.4 | 5.99 | 44 | 1 | HID58_094631 | 0 | 0.01 | 1E-17 | 5.5977E-17 |
| A0ABQ7X6Z8 | Uncharacterized protein OS=Brassica napus OX=3708 GN=HID58_095211 PE=4 SV=1 | 0 | 2.787 | 2 | 1 | 9 | 1 | 1048 | 117 | 6.87 | 52 | 1 | HID58_095211 | 0 | 0.01 | 1E-17 | 5.5977E-17 |
| A0ABQ7X8F3 | Uncharacterized protein (Fragment) OS=Brassica napus OX=3708 GN=HID58_094155 PE=4 SV=1 | 0 | 5.415 | 4 | 2 | 25 | 2 | 579 | 65.4 | 5 | 69 | 2 | HID58_094155 | 0 | 0.01 | 1E-17 | 5.5977E-17 |
| A0ABQ7X9R9 | Uncharacterized protein OS=Brassica napus OX=3708 GN=HID58_091216 PE=4 SV=1 | 0.002 | 1.334 | 4 | 1 | 1 | 1 | 580 | 65.4 | 7.23 | 0 | 1 | HID58_091216 | 0 | 0.01 | 1E-17 | 5.5977E-17 |
| A0ABQ7XA93 | Uncharacterized protein OS=Brassica napus OX=3708 GN=HID58_093628 PE=4 SV=1 | 0.004 | 1.217 | 2 | 1 | 1 | 1 | 517 | 56.8 | 7.8 | 26 | 1 | HID58_093628 | 0 | 0.01 | 1E-17 | 5.5977E-17 |
| A0ABQ7XC24 | Uncharacterized protein OS=Brassica napus OX=3708 GN=HID58_093120 PE=4 SV=1 | 0 | 12.267 | 7 | 1 | 23 | 1 | 351 | 40.5 | 6.61 | 126 | 1 | HID58_093120 | 0 | 0.01 | 1E-17 | 5.5977E-17 |
| A0ABQ7XCP9 | Uncharacterized protein OS=Brassica napus OX=3708 GN=HID58_090784 PE=4 SV=1 | 0 | 2.549 | 3 | 1 | 9 | 1 | 430 | 46.8 | 8.73 | 35 | 1 | HID58_090784 | 0 | 0.01 | 1E-17 | 5.5977E-17 |
| A0ABQ7XET9 | Probable type I protein arginine methyltransferase OS=Brassica napus OX=3708 GN=HID58_069219 PE=4 SV=1 | 0 | 2.656 | 3 | 1 | 3 | 1 | 625 | 69.2 | 5.44 | 0 | 1 | HID58_069219 | 0 | 0.01 | 1E-17 | 5.5977E-17 |
| A0ABQ7XHK5 | Probable SKP1-like protein OS=Brassica napus OX=3708 GN=HID58_007992 PE=4 SV=1 | 0 | 3.326 | 10 | 1 | 2 | 1 | 188 | 20.8 | 4.7 | 69 | 1 | HID58_007992 | 0 | 0.01 | 1E-17 | 5.5977E-17 |
| A0ABQ7XIY7 | Uncharacterized protein OS=Brassica napus OX=3708 GN=HID58_024544 PE=4 SV=1 | 0 | 1.773 | 2 | 1 | 6 | 1 | 445 | 49.5 | 8.82 | 33 | 1 | HID58_024544 | 0 | 0.01 | 1E-17 | 5.5977E-17 |
| A0ABQ7XRY7 | Uncharacterized protein OS=Brassica napus OX=3708 GN=HID58_086928 PE=4 SV=1 | 0 | 6.23 | 4 | 1 | 9 | 1 | 390 | 43.6 | 4.92 | 138 | 1 | HID58_086928 | 0 | 0.01 | 1E-17 | 5.5977E-17 |
| A0ABQ7XU89 | Probable Vesicle-fusing ATPase OS=Brassica napus OX=3708 GN=HID58_086726 PE=4 SV=1 | 0 | 5.699 | 4 | 2 | 8 | 2 | 749 | 82.4 | 6.27 | 105 | 2 | HID58_086726 | 0 | 0.01 | 1E-17 | 5.5977E-17 |
| A0ABQ7XVN8 | Uncharacterized protein (Fragment) OS=Brassica napus OX=3708 GN=HID58_088187 PE=4 SV=1 | 0 | 7.685 | 4 | 3 | 24 | 2 | 820 | 92.7 | 4.88 | 316 | 3 | HID58_088187 | 1 | 0.068 | 0.00426112 | 0.021585647 |
| A0ABQ7XXM9 | Uncharacterized protein OS=Brassica napus OX=3708 GN=HID58_088966 PE=4 SV=1 | 0 | 11.82 | 8 | 5 | 26 | 2 | 925 | 100.5 | 6.55 | 126 | 5 | HID58_088966 | 0 | 0.062 | 0.010098475 | 0.047600116 |
| A0ABQ7XXS5 | Uncharacterized protein OS=Brassica napus OX=3708 GN=HID58_088999 PE=4 SV=1 | 0 | 19.758 | 16 | 7 | 96 | 1 | 549 | 60.4 | 6.55 | 791 | 7 | HID58_088999 | 0 | 0.01 | 1E-17 | 5.5977E-17 |
| A0ABQ7XY62 | Probable COP9 signalosome complex subunit 3 (Fragment) OS=Brassica napus OX=3708 GN=HID58_089144 PE=4 SV=1 | 0 | 7.595 | 4 | 3 | 23 | 3 | 931 | 103.8 | 6.86 | 211 | 3 | HID58_089144 | 0 | 0.01 | 1E-17 | 5.5977E-17 |
| A0ABQ7XZ46 | Uncharacterized protein (Fragment) OS=Brassica napus OX=3708 GN=HID58_089475 PE=4 SV=1 | 0 | 6.223 | 2 | 2 | 17 | 1 | 1009 | 113.7 | 7.06 | 252 | 2 | HID58_089475 | 0 | 0.01 | 1E-17 | 5.5977E-17 |
| A0ABQ7XZR0 | Probable D-lactate dehydrogenase (cytochrome) protein OS=Brassica napus OX=3708 GN=HID58_089688 PE=4 SV=1 | 0 | 1.792 | 4 | 1 | 3 | 1 | 569 | 62.1 | 7.83 | 0 | 1 | HID58_089688 | 0 | 0.01 | 1E-17 | 5.5977E-17 |
| A0ABQ7Y0K9 | Probable Expansin protein OS=Brassica napus OX=3708 GN=HID58_089998 PE=4 SV=1 | 0 | 6.836 | 10 | 2 | 13 | 1 | 287 | 30.8 | 9.63 | 85 | 2 | HID58_089998 | 0 | 0.01 | 1E-17 | 5.5977E-17 |
| A0ABQ7Y0V4 | Uncharacterized protein OS=Brassica napus OX=3708 GN=HID58_079029 PE=4 SV=1 | 0.004 | 1.213 | 1 | 1 | 7 | 1 | 1131 | 125.4 | 6.1 | 21 | 1 | HID58_079029 | 0 | 0.088 | 0.024633874 | 0.104857896 |
| A0ABQ7Y132 | Uncharacterized protein OS=Brassica napus OX=3708 GN=HID58_079121 PE=4 SV=1 | 0 | 3.43 | 5 | 1 | 15 | 1 | 250 | 28.1 | 8.16 | 265 | 1 | HID58_079121 | 0 | 0.043 | 0.004546776 | 0.022806252 |
| A0ABQ7Y2K6 | Uncharacterized protein (Fragment) OS=Brassica napus OX=3708 GN=HID58_079033 PE=4 SV=1 | 0 | 2.737 | 3 | 2 | 10 | 2 | 1022 | 113.6 | 5.57 | 32 | 2 | HID58_079033 | 0 | 0.01 | 1E-17 | 5.5977E-17 |
| A0ABQ7Y3X8 | Uncharacterized protein (Fragment) OS=Brassica napus OX=3708 GN=HID58_080096 PE=4 SV=1 | 0.008 | 1.078 | 2 | 1 | 2 | 1 | 551 | 61.9 | 6.14 | 0 | 1 | HID58_080096 | 0 | 0.01 | 1E-17 | 5.5977E-17 |
| A0ABQ7Y5R3 | Uncharacterized protein OS=Brassica napus OX=3708 GN=HID58_080739 PE=4 SV=1 | 0 | 36.231 | 23 | 8 | 60 | 1 | 465 | 51.8 | 7.46 | 1180 | 8 | HID58_080739 | 0 | 0.01 | 1E-17 | 5.5977E-17 |
| A0ABQ7Y7Z4 | Uncharacterized protein OS=Brassica napus OX=3708 GN=HID58_081487 PE=4 SV=1 | 0 | 2.734 | 2 | 1 | 1 | 1 | 828 | 92.7 | 7.46 | 53 | 1 | HID58_081487 | 0 | 0.01 | 1E-17 | 5.5977E-17 |
| A0ABQ7Y807 | Uncharacterized protein (Fragment) OS=Brassica napus OX=3708 GN=HID58_080431 PE=4 SV=1 | 0.003 | 1.296 | 1 | 1 | 2 | 1 | 2333 | 248.3 | 5.26 | 0 | 1 | HID58_080431 | 0 | 0.01 | 1E-17 | 5.5977E-17 |
| A0ABQ7Y8M4 | Uncharacterized protein OS=Brassica napus OX=3708 GN=HID58_081745 PE=4 SV=1 | 0 | 5.724 | 4 | 2 | 11 | 1 | 601 | 66.5 | 5.92 | 196 | 2 | HID58_081745 | 0 | 0.01 | 1E-17 | 5.5977E-17 |
| A0ABQ7Y9R7 | Probable NADP-dependent oxidoreductase domain-containing protein OS=Brassica napus OX=3708 GN=HID58_082162 PE=4 SV=1 | 0 | 5.197 | 13 | 2 | 12 | 2 | 311 | 34.5 | 6.54 | 74 | 2 | HID58_082162 | 0 | 0.01 | 1E-17 | 5.5977E-17 |
| A0ABQ7YAA3 | Uncharacterized protein OS=Brassica napus OX=3708 GN=HID58_082340 PE=4 SV=1 | 0 | 3.51 | 2 | 1 | 28 | 1 | 1650 | 180.2 | 6.39 | 197 | 1 | HID58_082340 | 0 | 0.01 | 1E-17 | 5.5977E-17 |
| A0ABQ7YAI0 | Probable Anaphase-promoting complex subunit 4 WD40 domain-containing protein OS=Brassica napus OX=3708 GN=HID58_082405 PE=4 SV=1 | 0.003 | 1.256 | 2 | 1 | 10 | 1 | 481 | 52.4 | 5.88 | 39 | 1 | HID58_082405 | 0 | 0.01 | 1E-17 | 5.5977E-17 |
| A0ABQ7YAN3 | Probable [galactinol--sucrose galactosyltransferase](https://www.uniprot.org/uniprotkb/A0A0D3DTZ1/entry) protein OS=Brassica napus OX=3708 GN=HID58_082460 PE=4 SV=1 | 0 | 3.458 | 2 | 1 | 4 | 1 | 784 | 86.2 | 6.21 | 123 | 1 | HID58_082460 | 0 | 0.01 | 1E-17 | 5.5977E-17 |
| A0ABQ7YAR3 | Probable Deoxyhypusine hydroxylase protein (Fragment) OS=Brassica napus OX=3708 GN=HID58_082509 PE=4 SV=1 | 0.008 | 1.054 | 2 | 1 | 3 | 1 | 345 | 37.8 | 4.94 | 34 | 1 | HID58_082509 | 0 | 0.01 | 1E-17 | 5.5977E-17 |
| A0ABQ7YBT9 | Uncharacterized protein (Fragment) OS=Brassica napus OX=3708 GN=HID58_082880 PE=4 SV=1 | 0 | 3.015 | 9 | 2 | 12 | 2 | 250 | 27.9 | 9.44 | 49 | 2 | HID58_082880 | 0 | 0.01 | 1E-17 | 5.5977E-17 |
| A0ABQ7YCD2 | Uncharacterized protein OS=Brassica napus OX=3708 GN=HID58_083049 PE=4 SV=1 | 0 | 2.729 | 3 | 2 | 20 | 1 | 685 | 75.1 | 6.2 | 38 | 2 | HID58_083049 | 0 | 0.01 | 1E-17 | 5.5977E-17 |
| A0ABQ7YDB2 | Uncharacterized protein OS=Brassica napus OX=3708 GN=HID58_082407 PE=4 SV=1 | 0 | 2.553 | 1 | 1 | 1 | 1 | 1305 | 150.3 | 7.85 | 40 | 1 | HID58_082407 | 0 | 0.01 | 1E-17 | 5.5977E-17 |
| A0ABQ7YDY3 | Uncharacterized protein OS=Brassica napus OX=3708 GN=HID58_083606 PE=4 SV=1 | 0 | 2.342 | 2 | 1 | 4 | 1 | 460 | 52.5 | 8.76 | 72 | 1 | HID58_083606 | 0 | 0.01 | 1E-17 | 5.5977E-17 |
| A0ABQ7YEX5 | Probable non-specific serine/threonine protein kinase protein OS=Brassica napus OX=3708 GN=HID58_083475 PE=4 SV=1 | 0.007 | 1.108 | 3 | 1 | 1 | 1 | 452 | 50.8 | 7.84 | 0 | 1 | HID58_083475 | 0 | 0.01 | 1E-17 | 5.5977E-17 |
| A0ABQ7YF46 | Probable Large ribosomal subunit protein uL4m OS=Brassica napus OX=3708 GN=HID58_073862 PE=4 SV=1 | 0.001 | 1.471 | 4 | 1 | 6 | 1 | 331 | 36.7 | 10.21 | 51 | 1 | HID58_073862 | 0 | 0.01 | 1E-17 | 5.5977E-17 |
| A0ABQ7YFN9 | Probable serine--tRNA ligase protein OS=Brassica napus OX=3708 GN=HID58_083608 PE=4 SV=1 | 0 | 2.282 | 3 | 1 | 5 | 1 | 541 | 60.1 | 6.24 | 24 | 1 | HID58_083608 | 0 | 0.01 | 1E-17 | 5.5977E-17 |
| A0ABQ7YGC9 | Uncharacterized protein OS=Brassica napus OX=3708 GN=HID58_074233 PE=4 SV=1 | 0 | 6.245 | 11 | 2 | 14 | 1 | 264 | 29.8 | 6.86 | 76 | 2 | HID58_074233 | 0 | 0.01 | 1E-17 | 5.5977E-17 |
| A0ABQ7YIA5 | Uncharacterized protein (Fragment) OS=Brassica napus OX=3708 GN=HID58_074050 PE=4 SV=1 | 0 | 6.18 | 3 | 3 | 32 | 1 | 1170 | 132.6 | 6.98 | 411 | 3 | HID58_074050 | 0 | 0.01 | 1E-17 | 5.5977E-17 |
| A0ABQ7YJP1 | Probable IST1-like protein OS=Brassica napus OX=3708 GN=HID58_075442 PE=4 SV=1 | 0 | 1.847 | 2 | 1 | 3 | 1 | 367 | 41.2 | 8.81 | 50 | 1 | HID58_075442 | 0 | 0.01 | 1E-17 | 5.5977E-17 |
| A0ABQ7YKZ5 | Uncharacterized protein OS=Brassica napus OX=3708 GN=HID58_075901 PE=4 SV=1 | 0 | 19.042 | 27 | 2 | 28 | 2 | 77 | 8.9 | 9.29 | 609 | 2 | HID58_075901 | 0 | 0.01 | 1E-17 | 5.5977E-17 |
| A0ABQ7YL95 | Probable sulfate adenylyltransferase OS=Brassica napus OX=3708 GN=HID58_076011 PE=4 SV=1 | 0 | 15.402 | 8 | 6 | 48 | 3 | 924 | 103.1 | 8.22 | 573 | 6 | HID58_076011 | 0 | 0.01 | 1E-17 | 5.5977E-17 |
| A0ABQ7YMN3 | Uncharacterized protein (Fragment) OS=Brassica napus OX=3708 GN=HID58_075879 PE=4 SV=1 | 0 | 1.497 | 1 | 1 | 1 | 1 | 904 | 100.7 | 7.03 | 18 | 1 | HID58_075879 | 0 | 0.01 | 1E-17 | 5.5977E-17 |
| A0ABQ7YPG5 | Probable Peptide-N(4)-(N-acetyl-beta-glucosaminyl)asparagine amidase OS=Brassica napus OX=3708 GN=HID58_077098 PE=4 SV=1 | 0.001 | 1.472 | 2 | 1 | 2 | 1 | 730 | 83.3 | 5.41 | 0 | 1 | HID58_077098 | 0 | 0.01 | 1E-17 | 5.5977E-17 |
| A0ABQ7YQD0 | Uncharacterized protein OS=Brassica napus OX=3708 GN=HID58_077446 PE=4 SV=1 | 0 | 4.455 | 4 | 1 | 12 | 1 | 561 | 60.6 | 9.51 | 26 | 1 | HID58_077446 | 0 | 0.01 | 1E-17 | 5.5977E-17 |
| A0ABQ7YQR9 | Probable Vacuolar protein 14 C-terminal Fig4-binding domain-containing protein OS=Brassica napus OX=3708 GN=HID58_076493 PE=4 SV=1 | 0 | 5.018 | 3 | 2 | 20 | 2 | 753 | 85.1 | 5.88 | 155 | 2 | HID58_076493 | 0 | 0.01 | 1E-17 | 5.5977E-17 |
| A0ABQ7YRR1 | Uncharacterized protein (Fragment) OS=Brassica napus OX=3708 GN=HID58_077824 PE=4 SV=1 | 0.004 | 1.19 | 3 | 1 | 1 | 1 | 789 | 85.9 | 7.85 | 35 | 1 | HID58_077824 | 0 | 0.01 | 1E-17 | 5.5977E-17 |
| A0ABQ7YSD4 | Uncharacterized protein OS=Brassica napus OX=3708 GN=HID58_078150 PE=4 SV=1 | 0 | 2.265 | 3 | 1 | 4 | 1 | 391 | 43.5 | 8.62 | 29 | 1 | HID58_078150 | 0 | 0.01 | 1E-17 | 5.5977E-17 |
| A0ABQ7YT00 | Uncharacterized protein (Fragment) OS=Brassica napus OX=3708 GN=HID58_077996 PE=4 SV=1 | 0 | 5.385 | 2 | 3 | 21 | 1 | 2107 | 240.4 | 6.25 | 116 | 3 | HID58_077996 | 0 | 0.01 | 1E-17 | 5.5977E-17 |
| A0ABQ7YU82 | Probable Putative gamma-glutamylcyclotransferase protein OS=Brassica napus OX=3708 GN=HID58_078514 PE=4 SV=1 | 0 | 2.726 | 8 | 1 | 3 | 1 | 185 | 21.5 | 6.07 | 0 | 1 | HID58_078514 | 0 | 0.01 | 1E-17 | 5.5977E-17 |
| A0ABQ7YV06 | Probable CBS domain-containing protein OS=Brassica napus OX=3708 GN=HID58_078794 PE=4 SV=1 | 0 | 1.702 | 5 | 1 | 2 | 1 | 236 | 25.3 | 8.59 | 0 | 1 | HID58_078794 | 0 | 0.01 | 1E-17 | 5.5977E-17 |
| A0ABQ7YVD1 | Probable Glucose-1-phosphate adenylyltransferase protein OS=Brassica napus OX=3708 GN=HID58_078865 PE=4 SV=1 | 0 | 2.436 | 4 | 2 | 7 | 2 | 640 | 72.1 | 9.04 | 0 | 2 | HID58_078865 | 0 | 0.01 | 1E-17 | 5.5977E-17 |
| A0ABQ7YVH3 | Uncharacterized protein OS=Brassica napus OX=3708 GN=HID58_078903 PE=4 SV=1 | 0 | 7.259 | 4 | 3 | 28 | 2 | 1106 | 125.2 | 9.09 | 233 | 3 | HID58_078903 | 0 | 0.01 | 1E-17 | 5.5977E-17 |
| A0ABQ7YXR3 | Uncharacterized protein OS=Brassica napus OX=3708 GN=HID58_070082 PE=4 SV=1 | 0.008 | 1.076 | 1 | 1 | 6 | 1 | 504 | 55.8 | 7.36 | 0 | 1 | HID58_070082 | 0 | 0.01 | 1E-17 | 5.5977E-17 |
| A0ABQ7YY04 | Probable RRM domain-containing protein OS=Brassica napus OX=3708 GN=HID58_070181 PE=4 SV=1 | 0 | 2.503 | 4 | 2 | 20 | 1 | 427 | 47.3 | 7.06 | 114 | 2 | HID58_070181 | 0 | 0.01 | 1E-17 | 5.5977E-17 |
| A0ABQ7YZ90 | Probable Protein kinase domain-containing protein OS=Brassica napus OX=3708 GN=HID58_070590 PE=4 SV=1 | 0.006 | 1.134 | 1 | 1 | 4 | 1 | 1099 | 121.5 | 6.6 | 0 | 1 | HID58_070590 | 0 | 0.01 | 1E-17 | 5.5977E-17 |
| A0ABQ7Z1C8 | Uncharacterized protein (Fragment) OS=Brassica napus OX=3708 GN=HID58_071353 PE=4 SV=1 | 0 | 2.861 | 3 | 1 | 11 | 1 | 582 | 65.1 | 9.39 | 35 | 1 | HID58_071353 | 0 | 0.01 | 1E-17 | 5.5977E-17 |
| A0ABQ7Z1P0 | Probable TBCC domain-containing protein 1 OS=Brassica napus OX=3708 GN=HID58_071419 PE=4 SV=1 | 0 | 2.411 | 1 | 1 | 5 | 1 | 953 | 106.1 | 5.69 | 59 | 1 | HID58_071419 | 0 | 0.01 | 1E-17 | 5.5977E-17 |
| A0ABQ7Z220 | Uncharacterized protein OS=Brassica napus OX=3708 GN=HID58_071580 PE=4 SV=1 | 0 | 3.26 | 2 | 1 | 8 | 1 | 770 | 84.8 | 6.13 | 135 | 1 | HID58_071580 | 0 | 0.01 | 1E-17 | 5.5977E-17 |
| A0ABQ7Z2P0 | Probable Nucleoprotein TPR/MLP1-2 domain-containing protein OS=Brassica napus OX=3708 GN=HID58_071822 PE=4 SV=1 | 0 | 2.472 | 1 | 1 | 4 | 1 | 2120 | 239.8 | 5.07 | 21 | 1 | HID58_071822 | 0 | 0.01 | 1E-17 | 5.5977E-17 |
| A0ABQ7Z3W1 | Probable [ATP-dependent RNA helicase](https://www.uniprot.org/uniprotkb/A0A078FDY9/entry) OS=Brassica napus OX=3708 GN=HID58_072186 PE=4 SV=1 | 0 | 18.051 | 31 | 4 | 106 | 1 | 207 | 22.9 | 5.67 | 1333 | 4 | HID58_072186 | 0 | 0.01 | 1E-17 | 5.5977E-17 |
| A0ABQ7Z4J3 | Uncharacterized protein (Fragment) OS=Brassica napus OX=3708 GN=HID58_072397 PE=4 SV=1 | 0 | 9.69 | 19 | 2 | 30 | 2 | 198 | 22.5 | 9.44 | 209 | 2 | HID58_072397 | 0 | 0.078 | 0.026179688 | 0.110559392 |
| A0ABQ7Z4J6 | Uncharacterized protein (Fragment) OS=Brassica napus OX=3708 GN=HID58_072465 PE=4 SV=1 | 0 | 6.345 | 2 | 1 | 7 | 1 | 1085 | 121.1 | 7.25 | 200 | 1 | HID58_072465 | 0 | 0.01 | 1E-17 | 5.5977E-17 |
| A0ABQ7Z517 | Probable [50S ribosomal protein L22, chloroplastic](https://www.uniprot.org/uniprotkb/A0A0D3CY38/entry) protein OS=Brassica napus OX=3708 GN=HID58_072661 PE=4 SV=1 | 0 | 2.975 | 66 | 2 | 13 | 1 | 35 | 3.9 | 7.18 | 166 | 2 | HID58_072661 | 0 | 0.01 | 1E-17 | 5.5977E-17 |
| A0ABQ7Z5U2 | Uncharacterized protein OS=Brassica napus OX=3708 GN=HID58_072875 PE=4 SV=1 | 0 | 2.604 | 3 | 2 | 9 | 1 | 854 | 93.7 | 7.12 | 28 | 2 | HID58_072875 | 0 | 0.01 | 1E-17 | 5.5977E-17 |
| A0ABQ7Z6R8 | Probable phosphoribosylformylglycinamidine synthase OS=Brassica napus OX=3708 GN=HID58_073223 PE=4 SV=1 | 0 | 31.223 | 9 | 9 | 55 | 1 | 1503 | 165.3 | 5.85 | 691 | 9 | HID58_073223 | 0 | 0.01 | 1E-17 | 5.5977E-17 |
| A0ABQ7Z6Z4 | Probable Thioredoxin-like fold domain-containing protein OS=Brassica napus OX=3708 GN=HID58_073347 PE=4 SV=1 | 0 | 1.982 | 9 | 1 | 3 | 1 | 239 | 26.9 | 9.74 | 0 | 1 | HID58_073347 | 0 | 0.01 | 1E-17 | 5.5977E-17 |
| A0ABQ7Z734 | Uncharacterized protein (Fragment) OS=Brassica napus OX=3708 GN=HID58_073396 PE=4 SV=1 | 0.009 | 1.041 | 2 | 1 | 1 | 1 | 2064 | 228.4 | 8.75 | 0 | 1 | HID58_073396 | 0 | 0.01 | 1E-17 | 5.5977E-17 |
| A0ABQ7Z7F7 | Probable Fibronectin type III-like domain-containing protein (Fragment) OS=Brassica napus OX=3708 GN=HID58_073517 PE=4 SV=1 | 0 | 11.84 | 7 | 4 | 31 | 4 | 781 | 85.3 | 8.51 | 283 | 4 | HID58_073517 | 0 | 0.094 | 0.046064541 | 0.175416916 |
| A0ABQ7Z7L6 | Uncharacterized protein (Fragment) OS=Brassica napus OX=3708 GN=HID58_073559 PE=4 SV=1 | 0 | 16.818 | 4 | 4 | 43 | 3 | 1511 | 166.9 | 9.2 | 360 | 4 | HID58_073559 | 1 | 0.01 | 1E-17 | 5.5977E-17 |
| A0ABQ7Z888 | Uncharacterized protein (Fragment) OS=Brassica napus OX=3708 GN=HID58_063833 PE=4 SV=1 | 0 | 2.006 | 2 | 1 | 5 | 1 | 674 | 76.1 | 7.88 | 37 | 1 | HID58_063833 | 0 | 0.01 | 1E-17 | 5.5977E-17 |
| A0ABQ7Z8D6 | Probable D-isomer specific 2-hydroxyacid dehydrogenase NAD-binding domain-containing protein OS=Brassica napus OX=3708 GN=HID58_063782 PE=4 SV=1 | 0 | 1.862 | 2 | 1 | 1 | 1 | 996 | 109.7 | 6.54 | 38 | 1 | HID58_063782 | 0 | 0.01 | 1E-17 | 5.5977E-17 |
| A0ABQ7Z9G2 | Probable Sas10 C-terminal domain-containing protein OS=Brassica napus OX=3708 GN=HID58_064260 PE=4 SV=1 | 0 | 1.765 | 1 | 1 | 3 | 1 | 1050 | 120.7 | 9.29 | 44 | 1 | HID58_064260 | 0 | 0.01 | 1E-17 | 5.5977E-17 |
| A0ABQ7Z9Y6 | Uncharacterized protein OS=Brassica napus OX=3708 GN=HID58_064428 PE=4 SV=1 | 0 | 3.42 | 3 | 2 | 16 | 2 | 847 | 95.9 | 6.62 | 23 | 2 | HID58_064428 | 0 | 0.01 | 1E-17 | 5.5977E-17 |
| A0ABQ7ZAF7 | Uncharacterized protein OS=Brassica napus OX=3708 GN=HID58_064591 PE=4 SV=1 | 0 | 5.355 | 3 | 1 | 22 | 1 | 599 | 67.8 | 4.81 | 97 | 1 | HID58_064591 | 0 | 0.01 | 1E-17 | 5.5977E-17 |
| A0ABQ7ZBN3 | Probable ABC transporter domain-containing protein OS=Brassica napus OX=3708 GN=HID58_064933 PE=4 SV=1 | 0 | 9.384 | 4 | 5 | 26 | 1 | 1464 | 165.4 | 8.19 | 157 | 5 | HID58_064933 | 0 | 0.01 | 1E-17 | 5.5977E-17 |
| A0ABQ7ZCV3 | Uncharacterized protein OS=Brassica napus OX=3708 GN=HID58_065460 PE=4 SV=1 | 0 | 1.632 | 2 | 1 | 5 | 1 | 747 | 83.5 | 8.6 | 19 | 1 | HID58_065460 | 0 | 0.01 | 1E-17 | 5.5977E-17 |
| A0ABQ7ZDY6 | Uncharacterized protein (Fragment) OS=Brassica napus OX=3708 GN=HID58_065823 PE=4 SV=1 | 0.005 | 1.186 | 11 | 1 | 1 | 1 | 139 | 15.4 | 8.19 | 0 | 1 | HID58_065823 | 0 | 0.1 | 0.046640318 | 0.17754641 |
| A0ABQ7ZEJ1 | Probable CobW C-terminal domain-containing protein (Fragment) OS=Brassica napus OX=3708 GN=HID58_065907 PE=4 SV=1 | 0 | 1.496 | 3 | 1 | 6 | 1 | 380 | 42.5 | 4.86 | 26 | 1 | HID58_065907 | 0 | 0.01 | 1E-17 | 5.5977E-17 |
| A0ABQ7ZHQ8 | Probable Phosphoribulokinase protein OS=Brassica napus OX=3708 GN=HID58_067145 PE=4 SV=1 | 0 | 67.29 | 34 | 11 | 293 | 1 | 404 | 45.4 | 5.74 | 4146 | 11 | HID58_067145 | 17 | 0.059 | 0.009309184 | 0.044132124 |
| A0ABQ7ZIU3 | Uncharacterized protein OS=Brassica napus OX=3708 GN=HID58_067334 PE=4 SV=1 | 0 | 18.739 | 11 | 6 | 28 | 1 | 732 | 81.8 | 6.23 | 593 | 6 | HID58_067334 | 0 | 0.01 | 1E-17 | 5.5977E-17 |
| A0ABQ7ZKJ6 | Uncharacterized protein OS=Brassica napus OX=3708 GN=HID58_067976 PE=4 SV=1 | 0 | 3.705 | 12 | 1 | 3 | 1 | 209 | 23.2 | 7.71 | 45 | 1 | HID58_067976 | 0 | 0.01 | 1E-17 | 5.5977E-17 |
| A0ABQ7ZKN4 | Uncharacterized protein OS=Brassica napus OX=3708 GN=HID58_068122 PE=4 SV=1 | 0 | 7.507 | 8 | 2 | 15 | 2 | 552 | 61.2 | 8.92 | 121 | 2 | HID58_068122 | 0 | 0.01 | 1E-17 | 5.5977E-17 |
| A0ABQ7ZL02 | Probable Protein ROOT HAIR DEFECTIVE 3 homolog OS=Brassica napus OX=3708 GN=HID58_068293 PE=4 SV=1 | 0 | 3.628 | 2 | 1 | 4 | 1 | 868 | 97.5 | 6.25 | 25 | 1 | HID58_068293 | 0 | 0.01 | 1E-17 | 5.5977E-17 |
| A0ABQ7ZLI8 | Uncharacterized protein OS=Brassica napus OX=3708 GN=HID58_068307 PE=4 SV=1 | 0 | 1.856 | 8 | 1 | 9 | 1 | 264 | 29.4 | 5.07 | 23 | 1 | HID58_068307 | 0 | 0.01 | 1E-17 | 5.5977E-17 |
| A0ABQ7ZM08 | Probable DNA gyrase subunit B OS=Brassica napus OX=3708 GN=HID58_019818 PE=4 SV=1 | 0 | 1.931 | 1 | 1 | 8 | 1 | 1241 | 137.4 | 8.95 | 93 | 1 | HID58_019818 | 0 | 0.01 | 1E-17 | 5.5977E-17 |
| A0ABQ7ZMP2 | Uncharacterized protein OS=Brassica napus OX=3708 GN=HID58_068901 PE=4 SV=1 | 0 | 13.64 | 5 | 2 | 17 | 2 | 719 | 79.7 | 6.54 | 535 | 2 | HID58_068901 | 0 | 0.01 | 1E-17 | 5.5977E-17 |
| A0ABQ7ZNP5 | Probable 2-alkenal reductase [NAD(P)(+)] (Fragment) OS=Brassica napus OX=3708 GN=HID58_069055 PE=4 SV=1 | 0 | 13.534 | 9 | 3 | 30 | 3 | 360 | 40.1 | 7.99 | 367 | 3 | HID58_069055 | 0 | 0.071 | 0.025011702 | 0.106086849 |
| A0ABQ7ZNS7 | Uncharacterized protein OS=Brassica napus OX=3708 GN=HID58_057950 PE=4 SV=1 | 0 | 6.112 | 5 | 2 | 6 | 1 | 606 | 66.3 | 8.66 | 80 | 2 | HID58_057950 | 0 | 0.01 | 1E-17 | 5.5977E-17 |
| A0ABQ7ZQ40 | Uncharacterized protein (Fragment) OS=Brassica napus OX=3708 GN=HID58_058470 PE=4 SV=1 | 0 | 6.673 | 5 | 2 | 21 | 2 | 461 | 51.4 | 7.17 | 458 | 2 | HID58_058470 | 0 | 0.01 | 1E-17 | 5.5977E-17 |
| A0ABQ7ZR20 | Probable Serine/threonine-protein phosphatase (Fragment) OS=Brassica napus OX=3708 GN=HID58_058583 PE=4 SV=1 | 0 | 3.342 | 7 | 2 | 10 | 1 | 325 | 36.8 | 5.45 | 43 | 2 | HID58_058583 | 0 | 0.01 | 1E-17 | 5.5977E-17 |
| A0ABQ7ZTK5 | Uncharacterized protein OS=Brassica napus OX=3708 GN=HID58_059657 PE=4 SV=1 | 0 | 4.844 | 6 | 2 | 6 | 2 | 656 | 73.1 | 6.27 | 37 | 2 | HID58_059657 | 0 | 0.01 | 1E-17 | 5.5977E-17 |
| A0ABQ7ZTQ4 | Probable Expansin OS=Brassica napus OX=3708 GN=HID58_059730 PE=4 SV=1 | 0 | 7.745 | 10 | 2 | 9 | 1 | 269 | 29.5 | 9.52 | 186 | 2 | HID58_059730 | 0 | 0.01 | 1E-17 | 5.5977E-17 |
| A0ABQ7ZU62 | Uncharacterized protein OS=Brassica napus OX=3708 GN=HID58_059881 PE=4 SV=1 | 0.002 | 1.313 | 1 | 1 | 3 | 1 | 986 | 110.2 | 5.3 | 34 | 1 | HID58_059881 | 0 | 0.01 | 1E-17 | 5.5977E-17 |
| A0ABQ7ZU83 | Uncharacterized protein OS=Brassica napus OX=3708 GN=HID58_059908 PE=4 SV=1 | 0.004 | 1.206 | 2 | 1 | 1 | 1 | 713 | 79.1 | 6.6 | 26 | 1 | HID58_059908 | 0 | 0.01 | 1E-17 | 5.5977E-17 |
| A0ABQ7ZVE1 | Uncharacterized protein OS=Brassica napus OX=3708 GN=HID58_060330 PE=4 SV=1 | 0.001 | 1.439 | 1 | 1 | 1 | 1 | 1075 | 120.5 | 6.92 | 0 | 1 | HID58_060330 | 0 | 0.01 | 1E-17 | 5.5977E-17 |
| A0ABQ7ZYD6 | Probable C2 domain-containing protein OS=Brassica napus OX=3708 GN=HID58_061366 PE=4 SV=1 | 0.001 | 1.405 | 1 | 1 | 8 | 1 | 912 | 102.4 | 5.59 | 43 | 1 | HID58_061366 | 0 | 0.01 | 1E-17 | 5.5977E-17 |
| A0ABQ8A0U7 | Uncharacterized protein (Fragment) OS=Brassica napus OX=3708 GN=HID58_062018 PE=4 SV=1 | 0 | 4.026 | 4 | 2 | 18 | 1 | 630 | 68.4 | 9.14 | 330 | 2 | HID58_062018 | 0 | 0.01 | 1E-17 | 5.5977E-17 |
| A0ABQ8A1A1 | Uncharacterized protein OS=Brassica napus OX=3708 GN=HID58_062382 PE=4 SV=1 | 0.002 | 1.348 | 2 | 1 | 1 | 1 | 862 | 97.2 | 9.13 | 0 | 1 | HID58_062382 | 0 | 0.01 | 1E-17 | 5.5977E-17 |
| A0ABQ8A247 | Probable Importin N-terminal domain-containing protein (Fragment) OS=Brassica napus OX=3708 GN=HID58_062685 PE=4 SV=1 | 0.002 | 1.312 | 1 | 1 | 8 | 1 | 1075 | 122.7 | 4.93 | 25 | 1 | HID58_062685 | 0 | 0.01 | 1E-17 | 5.5977E-17 |
| A0ABQ8A2Q2 | Uncharacterized protein OS=Brassica napus OX=3708 GN=HID58_062604 PE=4 SV=1 | 0 | 3.161 | 3 | 1 | 9 | 1 | 503 | 56.8 | 6.58 | 52 | 1 | HID58_062604 | 0 | 0.01 | 1E-17 | 5.5977E-17 |
| A0ABQ8A5K1 | Probable Coatomer subunit delta (Fragment) OS=Brassica napus OX=3708 GN=HID58_050200 PE=4 SV=1 | 0 | 11.445 | 3 | 2 | 17 | 2 | 1027 | 115.1 | 6.8 | 334 | 2 | HID58_050200 | 0 | 0.01 | 1E-17 | 5.5977E-17 |
| A0ABQ8A5Q5 | Uncharacterized protein OS=Brassica napus OX=3708 GN=HID58_063562 PE=4 SV=1 | 0 | 2.829 | 0 | 1 | 9 | 1 | 3605 | 404.6 | 7.71 | 121 | 1 | HID58_063562 | 0 | 0.084 | 0.026591401 | 0.111441528 |
| A0ABQ8A5W0 | Uncharacterized protein OS=Brassica napus OX=3708 GN=HID58_050288 PE=4 SV=1 | 0 | 8.642 | 5 | 4 | 37 | 1 | 785 | 88 | 7.64 | 320 | 4 | HID58_050288 | 0 | 0.01 | 1E-17 | 5.5977E-17 |
| A0ABQ8A6D7 | Uncharacterized protein OS=Brassica napus OX=3708 GN=HID58_050500 PE=4 SV=1 | 0.004 | 1.22 | 2 | 1 | 1 | 1 | 409 | 46.1 | 9.33 | 0 | 1 | HID58_050500 | 0 | 0.087 | 0.044735303 | 0.171574742 |
| A0ABQ8A6T3 | Probable Glycosyltransferase (Fragment) OS=Brassica napus OX=3708 GN=HID58_050671 PE=4 SV=1 | 0.006 | 1.12 | 2 | 1 | 8 | 1 | 500 | 56.3 | 6.19 | 0 | 1 | HID58_050671 | 0 | 0.01 | 1E-17 | 5.5977E-17 |
| A0ABQ8A6U5 | Uncharacterized protein OS=Brassica napus OX=3708 GN=HID58_050672 PE=4 SV=1 | 0 | 5.241 | 1 | 1 | 6 | 1 | 1873 | 212.6 | 8.6 | 71 | 1 | HID58_050672 | 0 | 0.01 | 1E-17 | 5.5977E-17 |
| A0ABQ8A7D1 | Uncharacterized protein OS=Brassica napus OX=3708 GN=HID58_050878 PE=4 SV=1 | 0 | 1.718 | 3 | 1 | 6 | 1 | 513 | 59.2 | 5.38 | 0 | 1 | HID58_050878 | 0 | 0.01 | 1E-17 | 5.5977E-17 |
| A0ABQ8A7S2 | Probable non-specific serine/threonine protein kinase OS=Brassica napus OX=3708 GN=HID58_050529 PE=4 SV=1 | 0 | 2.637 | 2 | 1 | 10 | 1 | 524 | 58.1 | 5.52 | 59 | 1 | HID58_050529 | 0 | 0.01 | 1E-17 | 5.5977E-17 |
| A0ABQ8A844 | Uncharacterized protein OS=Brassica napus OX=3708 GN=HID58_051146 PE=4 SV=1 | 0 | 2.622 | 9 | 1 | 15 | 1 | 107 | 12.7 | 9.7 | 157 | 1 | HID58_051146 | 0 | 0.01 | 1E-17 | 5.5977E-17 |
| A0ABQ8A8C9 | Uncharacterized protein (Fragment) OS=Brassica napus OX=3708 GN=HID58_051208 PE=4 SV=1 | 0 | 3.646 | 5 | 2 | 11 | 1 | 525 | 58.2 | 9.42 | 44 | 2 | HID58_051208 | 0 | 0.01 | 1E-17 | 5.5977E-17 |
| A0ABQ8AA27 | Uncharacterized protein OS=Brassica napus OX=3708 GN=HID58_051828 PE=4 SV=1 | 0 | 5.446 | 4 | 2 | 8 | 2 | 900 | 98.7 | 5.72 | 43 | 2 | HID58_051828 | 0 | 0.01 | 1E-17 | 5.5977E-17 |
| A0ABQ8AAG5 | Uncharacterized protein OS=Brassica napus OX=3708 GN=HID58_051955 PE=4 SV=1 | 0.009 | 1.045 | 1 | 1 | 1 | 1 | 1597 | 175.9 | 8.22 | 0 | 1 | HID58_051955 | 0 | 0.01 | 1E-17 | 5.5977E-17 |
| A0ABQ8AB60 | Probable MIF4G domain-containing protein OS=Brassica napus OX=3708 GN=HID58_052186 PE=4 SV=1 | 0.004 | 1.239 | 1 | 1 | 1 | 1 | 1190 | 134.8 | 5.74 | 15 | 1 | HID58_052186 | 0 | 0.01 | 1E-17 | 5.5977E-17 |
| A0ABQ8AE57 | Probable 40S ribosomal protein S19 OS=Brassica napus OX=3708 GN=HID58_053239 PE=4 SV=1 | 0 | 11.653 | 7 | 4 | 19 | 4 | 597 | 67.4 | 8.91 | 254 | 4 | HID58_053239 | 0 | 0.024 | 0.000344735 | 0.00190092 |
| A0ABQ8AE70 | Probable Thioredoxin domain-containing protein OS=Brassica napus OX=3708 GN=HID58_052972 PE=4 SV=1 | 0 | 6.685 | 5 | 2 | 11 | 2 | 532 | 58.2 | 4.89 | 177 | 2 | HID58_052972 | 0 | 0.01 | 1E-17 | 5.5977E-17 |
| A0ABQ8AG61 | Probable Glutamine synthetase protein OS=Brassica napus OX=3708 GN=HID58_053968 PE=4 SV=1 | 0 | 9.974 | 15 | 3 | 25 | 1 | 362 | 39.6 | 7.31 | 188 | 3 | HID58_053968 | 0 | 0.01 | 1E-17 | 5.5977E-17 |
| A0ABQ8AGU5 | Probable peptidylprolyl isomerase protein OS=Brassica napus OX=3708 GN=HID58_054087 PE=4 SV=1 | 0 | 7.943 | 9 | 1 | 22 | 1 | 173 | 19 | 9.83 | 425 | 1 | HID58_054087 | 0 | 0.01 | 1E-17 | 5.5977E-17 |
| A0ABQ8AH24 | Uncharacterized protein OS=Brassica napus OX=3708 GN=HID58_053739 PE=4 SV=1 | 0.001 | 1.416 | 1 | 1 | 3 | 1 | 1810 | 202.9 | 7.09 | 105 | 1 | HID58_053739 | 0 | 0.01 | 1E-17 | 5.5977E-17 |
| A0ABQ8AHJ6 | Probable Terpene cyclase/mutase family member protein OS=Brassica napus OX=3708 GN=HID58_054460 PE=4 SV=1 | 0 | 1.701 | 2 | 1 | 7 | 1 | 793 | 89.6 | 6.57 | 29 | 1 | HID58_054460 | 0 | 0.01 | 1E-17 | 5.5977E-17 |
| A0ABQ8AIR4 | Uncharacterized protein OS=Brassica napus OX=3708 GN=HID58_054762 PE=4 SV=1 | 0 | 3.935 | 1 | 2 | 26 | 2 | 1508 | 173 | 5.21 | 216 | 2 | HID58_054762 | 0 | 0.071 | 0.019418719 | 0.086257114 |
| A0ABQ8AJI9 | Probable Importin N-terminal domain-containing protein (Fragment) OS=Brassica napus OX=3708 GN=HID58_055113 PE=4 SV=1 | 0 | 1.684 | 1 | 1 | 2 | 1 | 1006 | 112.2 | 6 | 40 | 1 | HID58_055113 | 0 | 0.01 | 1E-17 | 5.5977E-17 |
| A0ABQ8AMS0 | Probable Nucleoside phosphorylase domain-containing protein OS=Brassica napus OX=3708 GN=HID58_056278 PE=4 SV=1 | 0 | 3.899 | 6 | 1 | 4 | 1 | 315 | 33.7 | 5.07 | 36 | 1 | HID58_056278 | 0 | 0.01 | 1E-17 | 5.5977E-17 |
| A0ABQ8AMX9 | Uncharacterized protein (Fragment) OS=Brassica napus OX=3708 GN=HID58_056002 PE=4 SV=1 | 0 | 3.429 | 20 | 2 | 8 | 1 | 143 | 15.9 | 7.91 | 76 | 2 | HID58_056002 | 0 | 0.01 | 1E-17 | 5.5977E-17 |
| A0ABQ8AQG1 | Probable MSP domain-containing protein OS=Brassica napus OX=3708 GN=HID58_056857 PE=4 SV=1 | 0.008 | 1.058 | 4 | 1 | 1 | 1 | 312 | 34.8 | 8.16 | 21 | 1 | HID58_056857 | 0 | 0.01 | 1E-17 | 5.5977E-17 |
| A0ABQ8AQJ3 | Probable 40S ribosomal protein S6 (Fragment) OS=Brassica napus OX=3708 GN=HID58_057241 PE=4 SV=1 | 0 | 4.543 | 15 | 3 | 6 | 1 | 273 | 31 | 10.64 | 141 | 3 | HID58_057241 | 0 | 0.01 | 1E-17 | 5.5977E-17 |
| A0ABQ8AQY6 | Probable Disease resistance protein (Fragment) OS=Brassica napus OX=3708 GN=HID58_057383 PE=4 SV=1 | 0 | 1.638 | 1 | 1 | 4 | 1 | 1151 | 130.6 | 6.61 | 107 | 1 | HID58_057383 | 0 | 0.01 | 1E-17 | 5.5977E-17 |
| A0ABQ8ASE2 | Probable RNA helicase protein OS=Brassica napus OX=3708 GN=HID58_057592 PE=4 SV=1 | 0 | 55.766 | 51 | 16 | 229 | 1 | 422 | 48 | 5.48 | 2058 | 16 | HID58_057592 | 1 | 0.01 | 1E-17 | 5.5977E-17 |
| A0ABQ8AVC4 | Uncharacterized protein (Fragment) OS=Brassica napus OX=3708 GN=HID58_046011 PE=4 SV=1 | 0 | 2.288 | 4 | 1 | 5 | 1 | 338 | 37.3 | 8.15 | 27 | 1 | HID58_046011 | 0 | 0.01 | 1E-17 | 5.5977E-17 |
| A0ABQ8AVZ6 | Probable peptidylprolyl isomerase protein OS=Brassica napus OX=3708 GN=HID58_046262 PE=4 SV=1 | 0 | 15.849 | 14 | 7 | 51 | 7 | 548 | 61.5 | 5.5 | 553 | 7 | HID58_046262 | 0 | 0.076 | 0.018246351 | 0.081693025 |
| A0ABQ8AWH0 | Probable Bifunctional inhibitor/plant lipid transfer protein/seed storage helical domain-containing protein (Fragment) OS=Brassica napus OX=3708 GN=HID58_046245 PE=4 SV=1 | 0 | 1.547 | 5 | 1 | 2 | 1 | 260 | 28.3 | 8.48 | 42 | 1 | HID58_046245 | 0 | 0.01 | 1E-17 | 5.5977E-17 |
| A0ABQ8B1L1 | Uncharacterized protein OS=Brassica napus OX=3708 GN=HID58_047896 PE=4 SV=1 | 0 | 1.89 | 3 | 1 | 5 | 1 | 467 | 52.9 | 7.12 | 18 | 1 | HID58_047896 | 0 | 0.01 | 1E-17 | 5.5977E-17 |
| A0ABQ8B1T5 | Uncharacterized protein OS=Brassica napus OX=3708 GN=HID58_048015 PE=4 SV=1 | 0 | 5.545 | 4 | 3 | 28 | 3 | 648 | 73.5 | 6.23 | 364 | 3 | HID58_048015 | 0 | 0.104 | 0.048504723 | 0.183859758 |
| A0ABQ8B4I4 | Uncharacterized protein OS=Brassica napus OX=3708 GN=HID58_049282 PE=4 SV=1 | 0 | 5.354 | 13 | 3 | 7 | 1 | 367 | 42.7 | 5.58 | 21 | 3 | HID58_049282 | 0 | 0.01 | 1E-17 | 5.5977E-17 |
| A0ABQ8B5I2 | Uncharacterized protein OS=Brassica napus OX=3708 GN=HID58_049629 PE=4 SV=1 | 0 | 3.602 | 1 | 1 | 3 | 1 | 1635 | 180.6 | 7.81 | 55 | 1 | HID58_049629 | 0 | 0.01 | 1E-17 | 5.5977E-17 |
| A0ABQ8B728 | Uncharacterized protein (Fragment) OS=Brassica napus OX=3708 GN=HID58_049816 PE=4 SV=1 | 0 | 2.233 | 8 | 1 | 1 | 1 | 152 | 17.2 | 9.41 | 15 | 1 | HID58_049816 | 0 | 0.01 | 1E-17 | 5.5977E-17 |
| A0ABQ8B7K2 | Probable catalase protein OS=Brassica napus OX=3708 GN=HID58_040264 PE=4 SV=1 | 0 | 36.246 | 41 | 9 | 179 | 1 | 308 | 36 | 6.93 | 1619 | 9 | HID58_040264 | 0 | 0.01 | 1E-17 | 5.5977E-17 |
| A0ABQ8B7M0 | Uncharacterized protein OS=Brassica napus OX=3708 GN=HID58_040307 PE=4 SV=1 | 0 | 9.156 | 4 | 1 | 27 | 1 | 340 | 37.9 | 5.03 | 630 | 1 | HID58_040307 | 0 | 0.01 | 1E-17 | 5.5977E-17 |
| A0ABQ8B7S2 | Probable DJ-1/PfpI domain-containing protein OS=Brassica napus OX=3708 GN=HID58_040355 PE=4 SV=1 | 0.005 | 1.182 | 2 | 1 | 1 | 1 | 472 | 50.6 | 9.1 | 27 | 1 | HID58_040355 | 0 | 0.01 | 1E-17 | 5.5977E-17 |
| A0ABQ8B7T6 | Probable TIP41-like protein OS=Brassica napus OX=3708 GN=HID58_040333 PE=4 SV=1 | 0 | 1.841 | 4 | 1 | 5 | 1 | 305 | 34.7 | 5.71 | 24 | 1 | HID58_040333 | 0 | 0.01 | 1E-17 | 5.5977E-17 |
| A0ABQ8B833 | Probable legumain protein OS=Brassica napus OX=3708 GN=HID58_040444 PE=4 SV=1 | 0 | 3.06 | 3 | 1 | 12 | 1 | 491 | 53.6 | 5.38 | 56 | 1 | HID58_040444 | 0 | 0.01 | 1E-17 | 5.5977E-17 |
| A0ABQ8B864 | Uncharacterized protein OS=Brassica napus OX=3708 GN=HID58_040494 PE=4 SV=1 | 0 | 3.232 | 3 | 2 | 7 | 2 | 1133 | 121.6 | 7.69 | 0 | 2 | HID58_040494 | 0 | 0.01 | 1E-17 | 5.5977E-17 |
| A0ABQ8B944 | Probable Serine hydroxymethyltransferase protein OS=Brassica napus OX=3708 GN=HID58_040515 PE=4 SV=1 | 0 | 11.591 | 2 | 2 | 36 | 2 | 1254 | 139.5 | 7.96 | 963 | 2 | HID58_040515 | 0 | 0.01 | 1E-17 | 5.5977E-17 |
| A0ABQ8BA54 | Probable N-acetyltransferase domain-containing protein OS=Brassica napus OX=3708 GN=HID58_041197 PE=4 SV=1 | 0.009 | 1.044 | 4 | 1 | 1 | 1 | 237 | 26.4 | 6.64 | 0 | 1 | HID58_041197 | 0 | 0.01 | 1E-17 | 5.5977E-17 |
| A0ABQ8BA80 | Uncharacterized protein OS=Brassica napus OX=3708 GN=HID58_040664 PE=4 SV=1 | 0 | 8.157 | 3 | 2 | 15 | 1 | 891 | 99.2 | 6.95 | 337 | 2 | HID58_040664 | 0 | 0.01 | 1E-17 | 5.5977E-17 |
| A0ABQ8BAK3 | Uncharacterized protein (Fragment) OS=Brassica napus OX=3708 GN=HID58_041350 PE=4 SV=1 | 0.003 | 1.279 | 1 | 1 | 2 | 1 | 1015 | 115.1 | 6.67 | 19 | 1 | HID58_041350 | 0 | 0.01 | 1E-17 | 5.5977E-17 |
| A0ABQ8BAP4 | Probable Peroxidase protein OS=Brassica napus OX=3708 GN=HID58_041374 PE=4 SV=1 | 0 | 3.378 | 10 | 2 | 6 | 2 | 351 | 39.8 | 8.22 | 39 | 2 | HID58_041374 | 0 | 0.01 | 1E-17 | 5.5977E-17 |
| A0ABQ8BBJ9 | Uncharacterized protein OS=Brassica napus OX=3708 GN=HID58_041695 PE=4 SV=1 | 0 | 2.402 | 1 | 1 | 12 | 1 | 1479 | 163.3 | 5.92 | 32 | 1 | HID58_041695 | 0 | 0.01 | 1E-17 | 5.5977E-17 |
| A0ABQ8BBK7 | Probable Manganese-dependent ADP-ribose/CDP-alcohol diphosphatase protein OS=Brassica napus OX=3708 GN=HID58_041689 PE=4 SV=1 | 0 | 4.762 | 1 | 1 | 8 | 1 | 1252 | 141.3 | 7.55 | 300 | 1 | HID58_041689 | 0 | 0.01 | 1E-17 | 5.5977E-17 |
| A0ABQ8BCE5 | Uncharacterized protein OS=Brassica napus OX=3708 GN=HID58_041429 PE=4 SV=1 | 0 | 2.968 | 5 | 1 | 3 | 1 | 274 | 31.3 | 8.69 | 0 | 1 | HID58_041429 | 0 | 0.01 | 1E-17 | 5.5977E-17 |
| A0ABQ8BCT4 | Probable Aminotransferase class I/classII large domain-containing protein OS=Brassica napus OX=3708 GN=HID58_041568 PE=4 SV=1 | 0 | 3.004 | 3 | 1 | 17 | 1 | 427 | 47.5 | 8.19 | 71 | 1 | HID58_041568 | 0 | 0.01 | 1E-17 | 5.5977E-17 |
| A0ABQ8BCX8 | Probable SUN domain-containing protein OS=Brassica napus OX=3708 GN=HID58_041613 PE=4 SV=1 | 0.001 | 1.401 | 1 | 1 | 3 | 1 | 1499 | 168.4 | 7.43 | 41 | 1 | HID58_041613 | 0 | 0.01 | 1E-17 | 5.5977E-17 |
| A0ABQ8BDP2 | Uncharacterized protein OS=Brassica napus OX=3708 GN=HID58_042447 PE=4 SV=1 | 0 | 74.85 | 42 | 19 | 425 | 1 | 392 | 41.5 | 6.89 | 5959 | 19 | HID58_042447 | 0 | 0.01 | 1E-17 | 5.5977E-17 |
| A0ABQ8BDR2 | Uncharacterized protein (Fragment) OS=Brassica napus OX=3708 GN=HID58_042105 PE=4 SV=1 | 0 | 14.377 | 34 | 6 | 51 | 1 | 230 | 25.5 | 7.42 | 554 | 6 | HID58_042105 | 0 | 0.01 | 1E-17 | 5.5977E-17 |
| A0ABQ8BGN1 | Uncharacterized protein OS=Brassica napus OX=3708 GN=HID58_043462 PE=4 SV=1 | 0 | 2.041 | 1 | 1 | 16 | 1 | 948 | 103 | 8.9 | 117 | 1 | HID58_043462 | 0 | 0.01 | 1E-17 | 5.5977E-17 |
| A0ABQ8BHT2 | Uncharacterized protein OS=Brassica napus OX=3708 GN=HID58_043873 PE=4 SV=1 | 0 | 2.401 | 7 | 1 | 4 | 1 | 185 | 21.4 | 7.17 | 43 | 1 | HID58_043873 | 0 | 0.094 | 0.037762872 | 0.150015355 |
| A0ABQ8BIF9 | Probable CREG-like beta-barrel domain-containing protein (Fragment) OS=Brassica napus OX=3708 GN=HID58_043548 PE=4 SV=1 | 0 | 2.525 | 4 | 1 | 5 | 1 | 403 | 44.3 | 6.61 | 45 | 1 | HID58_043548 | 0 | 0.01 | 1E-17 | 5.5977E-17 |
| A0ABQ8BJL2 | Uncharacterized protein OS=Brassica napus OX=3708 GN=HID58_044481 PE=4 SV=1 | 0 | 10.107 | 23 | 3 | 19 | 3 | 236 | 26.5 | 8.35 | 103 | 3 | HID58_044481 | 0 | 0.062 | 0.014627077 | 0.066786491 |
| A0ABQ8BKB9 | Uncharacterized protein OS=Brassica napus OX=3708 GN=HID58_044755 PE=4 SV=1 | 0.001 | 1.408 | 2 | 1 | 1 | 1 | 393 | 42.3 | 6.04 | 0 | 1 | HID58_044755 | 0 | 0.01 | 1E-17 | 5.5977E-17 |
| A0ABQ8BLL2 | Uncharacterized protein OS=Brassica napus OX=3708 GN=HID58_044731 PE=4 SV=1 | 0 | 3.426 | 27 | 1 | 3 | 1 | 71 | 8 | 4.7 | 23 | 1 | HID58_044731 | 0 | 0.01 | 1E-17 | 5.5977E-17 |
| A0ABQ8BLL9 | Uncharacterized protein OS=Brassica napus OX=3708 GN=HID58_044594 PE=4 SV=1 | 0 | 5.358 | 2 | 3 | 14 | 2 | 1594 | 180 | 7.27 | 47 | 3 | HID58_044594 | 1 | 0.01 | 1E-17 | 5.5977E-17 |
| A0ABQ8BM46 | Uncharacterized protein OS=Brassica napus OX=3708 GN=HID58_044789 PE=4 SV=1 | 0 | 3.701 | 6 | 1 | 10 | 1 | 295 | 33.7 | 6.99 | 66 | 1 | HID58_044789 | 0 | 0.01 | 1E-17 | 5.5977E-17 |
| A0ABQ8BPD3 | Uncharacterized protein (Fragment) OS=Brassica napus OX=3708 GN=HID58_037863 PE=4 SV=1 | 0 | 1.791 | 5 | 1 | 3 | 1 | 237 | 26.2 | 6.25 | 0 | 1 | HID58_037863 | 0 | 0.01 | 1E-17 | 5.5977E-17 |
| A0ABQ8BPQ0 | Probable glucan endo-1,3-beta-D-glucosidase protein OS=Brassica napus OX=3708 GN=HID58_038579 PE=4 SV=1 | 0 | 1.737 | 3 | 1 | 4 | 1 | 498 | 54.7 | 8.46 | 43 | 1 | HID58_038579 | 0 | 0.01 | 1E-17 | 5.5977E-17 |
| A0ABQ8BQS8 | Probable L-gulonolactone oxidase protein OS=Brassica napus OX=3708 GN=HID58_038437 PE=4 SV=1 | 0 | 3.493 | 5 | 2 | 3 | 1 | 587 | 65 | 7.81 | 0 | 2 | HID58_038437 | 0 | 0.01 | 1E-17 | 5.5977E-17 |
| A0ABQ8BRB7 | Probable Alkaline/neutral invertase protein OS=Brassica napus OX=3708 GN=HID58_038750 PE=4 SV=1 | 0 | 7.003 | 6 | 3 | 16 | 3 | 676 | 76.3 | 6.83 | 65 | 3 | HID58_038750 | 0 | 0.102 | 0.027596623 | 0.115113967 |
| A0ABQ8BRS4 | Uncharacterized protein OS=Brassica napus OX=3708 GN=HID58_039346 PE=4 SV=1 | 0 | 2.042 | 1 | 1 | 2 | 1 | 1126 | 126.8 | 6.16 | 25 | 1 | HID58_039346 | 0 | 0.01 | 1E-17 | 5.5977E-17 |
| A0ABQ8BT57 | Probable GST N-terminal domain-containing protein OS=Brassica napus OX=3708 GN=HID58_039811 PE=4 SV=1 | 0 | 2.373 | 4 | 1 | 3 | 1 | 366 | 40.6 | 8.25 | 115 | 1 | HID58_039811 | 0 | 0.01 | 1E-17 | 5.5977E-17 |
| A0ABQ8BTQ9 | Probable 60S acidic ribosomal protein P1 (Fragment) OS=Brassica napus OX=3708 GN=HID58_031542 PE=4 SV=1 | 0 | 1.835 | 33 | 1 | 3 | 1 | 114 | 11.4 | 4.32 | 0 | 1 | HID58_031542 | 0 | 0.01 | 1E-17 | 5.5977E-17 |
| A0ABQ8BVZ4 | Uncharacterized protein OS=Brassica napus OX=3708 GN=HID58_032300 PE=4 SV=1 | 0.009 | 1.045 | 10 | 1 | 1 | 1 | 143 | 16.3 | 5.39 | 0 | 1 | HID58_032300 | 0 | 0.01 | 1E-17 | 5.5977E-17 |
| A0ABQ8BWW7 | Probable Histone-binding protein RBBP4-like N-terminal domain-containing protein OS=Brassica napus OX=3708 GN=HID58_032611 PE=4 SV=1 | 0 | 3.089 | 4 | 1 | 14 | 1 | 506 | 55.5 | 5.99 | 22 | 1 | HID58_032611 | 0 | 0.01 | 1E-17 | 5.5977E-17 |
| A0ABQ8BX32 | Probable TOG domain-containing protein OS=Brassica napus OX=3708 GN=HID58_032639 PE=4 SV=1 | 0 | 4.451 | 2 | 2 | 10 | 2 | 1434 | 158.5 | 7.11 | 101 | 2 | HID58_032639 | 0 | 0.01 | 1E-17 | 5.5977E-17 |
| A0ABQ8BX98 | Uncharacterized protein OS=Brassica napus OX=3708 GN=HID58_032768 PE=4 SV=1 | 0 | 3.583 | 7 | 1 | 8 | 1 | 242 | 26 | 8.29 | 31 | 1 | HID58_032768 | 0 | 0.01 | 1E-17 | 5.5977E-17 |
| A0ABQ8BXJ8 | Uncharacterized protein OS=Brassica napus OX=3708 GN=HID58_032844 PE=4 SV=1 | 0 | 1.555 | 3 | 1 | 9 | 1 | 328 | 36 | 5.06 | 31 | 1 | HID58_032844 | 0 | 0.01 | 1E-17 | 5.5977E-17 |
| A0ABQ8BXP3 | Uncharacterized protein OS=Brassica napus OX=3708 GN=HID58_032830 PE=4 SV=1 | 0 | 3.129 | 1 | 1 | 2 | 1 | 2195 | 244.9 | 6.02 | 21 | 1 | HID58_032830 | 0 | 0.01 | 1E-17 | 5.5977E-17 |
| A0ABQ8C2B4 | Probable Coatomer subunit epsilon protein (Fragment) OS=Brassica napus OX=3708 GN=HID58_034494 PE=4 SV=1 | 0 | 3.749 | 5 | 1 | 9 | 1 | 309 | 34.6 | 5.5 | 119 | 1 | HID58_034494 | 0 | 0.01 | 1E-17 | 5.5977E-17 |
| A0ABQ8C4F8 | Uncharacterized protein OS=Brassica napus OX=3708 GN=HID58_035277 PE=4 SV=1 | 0 | 14.806 | 13 | 4 | 29 | 1 | 492 | 52.8 | 6.37 | 443 | 4 | HID58_035277 | 0 | 0.01 | 1E-17 | 5.5977E-17 |
| A0ABQ8C5C4 | Uncharacterized protein (Fragment) OS=Brassica napus OX=3708 GN=HID58_035590 PE=4 SV=1 | 0 | 3.768 | 2 | 1 | 10 | 1 | 832 | 92.8 | 7.97 | 144 | 1 | HID58_035590 | 0 | 0.01 | 1E-17 | 5.5977E-17 |
| A0ABQ8C5S3 | Probable Guanosine nucleotide diphosphate dissociation inhibitor protein OS=Brassica napus OX=3708 GN=HID58_035750 PE=4 SV=1 | 0 | 15.262 | 6 | 2 | 25 | 1 | 452 | 50.3 | 5.92 | 380 | 2 | HID58_035750 | 0 | 0.01 | 1E-17 | 5.5977E-17 |
| A0ABQ8C5S8 | Uncharacterized protein OS=Brassica napus OX=3708 GN=HID58_035136 PE=4 SV=1 | 0.004 | 1.244 | 1 | 1 | 3 | 1 | 811 | 92.4 | 9.07 | 34 | 1 | HID58_035136 | 0 | 0.01 | 1E-17 | 5.5977E-17 |
| A0ABQ8C736 | Uncharacterized protein OS=Brassica napus OX=3708 GN=HID58_036211 PE=4 SV=1 | 0 | 6.469 | 2 | 2 | 9 | 1 | 1405 | 156.1 | 6.52 | 130 | 2 | HID58_036211 | 1 | 0.01 | 1E-17 | 5.5977E-17 |
| A0ABQ8C8C1 | Uncharacterized protein OS=Brassica napus OX=3708 GN=HID58_036596 PE=4 SV=1 | 0 | 18.424 | 17 | 4 | 50 | 4 | 326 | 35.3 | 7.99 | 1288 | 4 | HID58_036596 | 0 | 0.055 | 0.008734831 | 0.041482704 |
| A0ABQ8C9A8 | Uncharacterized protein OS=Brassica napus OX=3708 GN=HID58_036973 PE=4 SV=1 | 0 | 2.588 | 2 | 1 | 3 | 1 | 630 | 69.9 | 8.56 | 66 | 1 | HID58_036973 | 0 | 0.01 | 1E-17 | 5.5977E-17 |
| A0ABQ8C9P0 | Uncharacterized protein OS=Brassica napus OX=3708 GN=HID58_036604 PE=4 SV=1 | 0 | 1.802 | 1 | 1 | 3 | 1 | 1301 | 143.5 | 5.24 | 0 | 1 | HID58_036604 | 0 | 0.01 | 1E-17 | 5.5977E-17 |
| A0ABQ8CAQ9 | Probable Signal recognition particle 54 kDa proteinUncharacterized protein (Fragment) OS=Brassica napus OX=3708 GN=HID58_028607 PE=4 SV=1 | 0 | 3.991 | 3 | 1 | 11 | 1 | 503 | 55.6 | 9.07 | 57 | 1 | HID58_028607 | 0 | 0.01 | 1E-17 | 5.5977E-17 |
| A0ABQ8CC64 | Probable 3-oxoacyl-[acyl-carrier-protein] reductase protein OS=Brassica napus OX=3708 GN=HID58_028387 PE=4 SV=1 | 0.001 | 1.391 | 4 | 1 | 4 | 1 | 263 | 28.6 | 8.13 | 29 | 1 | HID58_028387 | 0 | 0.01 | 1E-17 | 5.5977E-17 |
| A0ABQ8CCJ5 | Uncharacterized protein OS=Brassica napus OX=3708 GN=HID58_029245 PE=4 SV=1 | 0.006 | 1.128 | 4 | 1 | 3 | 1 | 208 | 22.9 | 4.98 | 0 | 1 | HID58_029245 | 0 | 0.01 | 1E-17 | 5.5977E-17 |
| A0ABQ8CCW0 | Probable Importin subunit alpha protein OS=Brassica napus OX=3708 GN=HID58_029340 PE=4 SV=1 | 0 | 14.137 | 13 | 4 | 26 | 2 | 538 | 59.2 | 5.1 | 199 | 4 | HID58_029340 | 0 | 0.01 | 1E-17 | 5.5977E-17 |
| A0ABQ8CDE1 | Probable SET domain-containing protein OS=Brassica napus OX=3708 GN=HID58_029548 PE=4 SV=1 | 0 | 1.633 | 1 | 1 | 1 | 1 | 777 | 89.3 | 6.58 | 33 | 1 | HID58_029548 | 0 | 0.01 | 1E-17 | 5.5977E-17 |
| A0ABQ8CFA8 | Probable Phosphotransferase protein OS=Brassica napus OX=3708 GN=HID58_030222 PE=4 SV=1 | 0 | 2.988 | 2 | 1 | 27 | 1 | 595 | 65 | 7.44 | 84 | 1 | HID58_030222 | 0 | 0.01 | 1E-17 | 5.5977E-17 |
| A0ABQ8CG13 | Probable Small ribosomal subunit protein eS1 OS=Brassica napus OX=3708 GN=HID58_029707 PE=4 SV=1 | 0 | 8.791 | 9 | 2 | 13 | 1 | 258 | 29.5 | 9.76 | 249 | 2 | HID58_029707 | 0 | 0.01 | 1E-17 | 5.5977E-17 |
| A0ABQ8CGW5 | Probable DEK-C domain-containing protein OS=Brassica napus OX=3708 GN=HID58_030056 PE=4 SV=1 | 0 | 2.893 | 1 | 1 | 11 | 1 | 1399 | 155.3 | 5.25 | 43 | 1 | HID58_030056 | 0 | 0.01 | 1E-17 | 5.5977E-17 |
| A0ABQ8CHV8 | Probable Glucose-6-phosphate 1-dehydrogenase protein (Fragment) OS=Brassica napus OX=3708 GN=HID58_031092 PE=4 SV=1 | 0 | 1.924 | 2 | 1 | 11 | 1 | 691 | 77.1 | 6.38 | 92 | 1 | HID58_031092 | 0 | 0.01 | 1E-17 | 5.5977E-17 |
| A0ABQ8CJQ7 | Probable Protein transport protein SEC23 (Fragment) OS=Brassica napus OX=3708 GN=HID58_031239 PE=4 SV=1 | 0 | 8.53 | 5 | 3 | 37 | 1 | 780 | 86.1 | 5.5 | 94 | 3 | HID58_031239 | 0 | 0.01 | 1E-17 | 5.5977E-17 |
| A0ABQ8CKA8 | Uncharacterized protein OS=Brassica napus OX=3708 GN=HID58_025123 PE=4 SV=1 | 0.008 | 1.068 | 6 | 1 | 1 | 1 | 402 | 43.8 | 4.56 | 0 | 1 | HID58_025123 | 0 | 0.01 | 1E-17 | 5.5977E-17 |
| A0ABQ8CKT8 | Probable Vacuolar protein sorting-associated protein 35 OS=Brassica napus OX=3708 GN=HID58_024843 PE=4 SV=1 | 0 | 2.694 | 2 | 1 | 4 | 1 | 808 | 91.8 | 5.43 | 79 | 1 | HID58_024843 | 0 | 0.01 | 1E-17 | 5.5977E-17 |
| A0ABQ8CLA4 | Probable HIT domain-containing protein OS=Brassica napus OX=3708 GN=HID58_025493 PE=4 SV=1 | 0.007 | 1.106 | 7 | 1 | 1 | 1 | 202 | 21.9 | 9.09 | 32 | 1 | HID58_025493 | 0 | 0.01 | 1E-17 | 5.5977E-17 |
| A0ABQ8CMJ5 | Probable Catalase OS=Brassica napus OX=3708 GN=HID58_025933 PE=4 SV=1 | 0 | 101.751 | 32 | 22 | 570 | 2 | 947 | 109.2 | 7.24 | 5592 | 22 | HID58_025933 | 3 | 0.01 | 1E-17 | 5.5977E-17 |
| A0ABQ8CPL0 | Probable Beta-galactosidase protein OS=Brassica napus OX=3708 GN=HID58_026674 PE=4 SV=1 | 0 | 32.913 | 8 | 5 | 82 | 2 | 1072 | 118.9 | 7.85 | 862 | 5 | HID58_026674 | 4 | 0.018 | 8.81376E-05 | 0.000490549 |
| A0ABQ8CPL1 | Probable RNA helicase protein OS=Brassica napus OX=3708 GN=HID58_026665 PE=4 SV=1 | 0 | 5.996 | 10 | 3 | 12 | 2 | 495 | 56.1 | 7.97 | 22 | 3 | HID58_026665 | 0 | 0.01 | 1E-17 | 5.5977E-17 |
| A0ABQ8CQD4 | Probable beta-ketoacyl-[acyl-carrier-protein] synthase I OS=Brassica napus OX=3708 GN=HID58_026944 PE=4 SV=1 | 0 | 4.65 | 4 | 1 | 8 | 1 | 571 | 60.9 | 8.19 | 29 | 1 | HID58_026944 | 0 | 0.01 | 1E-17 | 5.5977E-17 |
| A0ABQ8CQM1 | Uncharacterized protein OS=Brassica napus OX=3708 GN=HID58_027010 PE=4 SV=1 | 0.003 | 1.27 | 2 | 1 | 4 | 1 | 571 | 62.8 | 9.28 | 16 | 1 | HID58_027010 | 0 | 0.01 | 1E-17 | 5.5977E-17 |
| A0ABQ8CR66 | Probable Sedoheptulose-1,7-bisphosphatase, chloroplastic OS=Brassica napus OX=3708 GN=HID58_026476 PE=4 SV=1 | 0 | 51.761 | 30 | 10 | 191 | 1 | 394 | 42.6 | 6.4 | 2838 | 10 | HID58_026476 | 0 | 0.01 | 1E-17 | 5.5977E-17 |
| A0ABQ8CRN5 | Probable mitogen-activated protein kinase OS=Brassica napus OX=3708 GN=HID58_026626 PE=4 SV=1 | 0 | 3.895 | 4 | 2 | 4 | 1 | 502 | 57.3 | 7.14 | 27 | 2 | HID58_026626 | 0 | 0.01 | 1E-17 | 5.5977E-17 |
| A0ABQ8CSG4 | Probable RNA helicase protein OS=Brassica napus OX=3708 GN=HID58_027685 PE=4 SV=1 | 0 | 38.576 | 18 | 12 | 197 | 1 | 906 | 101.9 | 8.7 | 1520 | 12 | HID58_027685 | 0 | 0.01 | 1E-17 | 5.5977E-17 |
| A0ABQ8CTJ7 | Probable V-type proton ATPase catalytic subunit A protein OS=Brassica napus OX=3708 GN=HID58_028072 PE=4 SV=1 | 0 | 99.865 | 45 | 24 | 359 | 1 | 646 | 71.3 | 5.21 | 4832 | 24 | HID58_028072 | 0 | 0.074 | 0.018694412 | 0.08336813 |
| A0ABQ8CU80 | Probable Smr domain-containing protein OS=Brassica napus OX=3708 GN=HID58_027798 PE=4 SV=1 | 0 | 1.572 | 1 | 1 | 3 | 1 | 1106 | 123.6 | 6.43 | 27 | 1 | HID58_027798 | 0 | 0.01 | 1E-17 | 5.5977E-17 |
| A0ABQ8CUV5 | Uncharacterized protein (Fragment) OS=Brassica napus OX=3708 GN=HID58_027706 PE=4 SV=1 | 0.006 | 1.139 | 1 | 1 | 3 | 1 | 1219 | 133.5 | 5.64 | 29 | 1 | HID58_027706 | 0 | 0.01 | 1E-17 | 5.5977E-17 |
| A0ABQ8CV72 | Probable 3-isopropylmalate dehydrogenase, chloroplastic OS=Brassica napus OX=3708 GN=HID58_028158 PE=4 SV=1 | 0 | 5.653 | 8 | 2 | 16 | 1 | 411 | 44 | 6.57 | 59 | 2 | HID58_028158 | 0 | 0.01 | 1E-17 | 5.5977E-17 |
| A0ABQ8CV81 | Probable Vacuolar protein sorting-associated protein 41 homolog OS=Brassica napus OX=3708 GN=HID58_021034 PE=4 SV=1 | 0 | 1.751 | 1 | 1 | 7 | 1 | 996 | 111.5 | 5.26 | 0 | 1 | HID58_021034 | 0 | 0.01 | 1E-17 | 5.5977E-17 |
| A0ABQ8CVC4 | Uncharacterized protein OS=Brassica napus OX=3708 GN=HID58_020561 PE=4 SV=1 | 0 | 4.195 | 1 | 2 | 11 | 2 | 1909 | 216.5 | 6.77 | 102 | 2 | HID58_020561 | 0 | 0.01 | 1E-17 | 5.5977E-17 |
| A0ABQ8CVP8 | Uncharacterized protein OS=Brassica napus OX=3708 GN=HID58_020681 PE=4 SV=1 | 0 | 2.55 | 4 | 2 | 14 | 1 | 812 | 89.9 | 8.87 | 289 | 2 | HID58_020681 | 0 | 0.01 | 1E-17 | 5.5977E-17 |
| A0ABQ8CW06 | Uncharacterized protein OS=Brassica napus OX=3708 GN=HID58_021232 PE=4 SV=1 | 0.007 | 1.081 | 1 | 1 | 9 | 1 | 1851 | 207.4 | 6.29 | 51 | 1 | HID58_021232 | 0 | 0.01 | 1E-17 | 5.5977E-17 |
| A0ABQ8CW79 | Probable Heat shock 70 kDa protein 16 OS=Brassica napus OX=3708 GN=HID58_021306 PE=4 SV=1 | 0 | 1.878 | 2 | 1 | 5 | 1 | 764 | 84.9 | 6.13 | 0 | 1 | HID58_021306 | 0 | 0.01 | 1E-17 | 5.5977E-17 |
| A0ABQ8CWH4 | Uncharacterized protein OS=Brassica napus OX=3708 GN=HID58_021452 PE=4 SV=1 | 0 | 3.381 | 1 | 1 | 11 | 1 | 1089 | 119.6 | 8.05 | 125 | 1 | HID58_021452 | 0 | 0.01 | 1E-17 | 5.5977E-17 |
| A0ABQ8CXI3 | Uncharacterized protein OS=Brassica napus OX=3708 GN=HID58_021843 PE=4 SV=1 | 0 | 7.854 | 7 | 3 | 18 | 1 | 654 | 71.1 | 6.89 | 59 | 3 | HID58_021843 | 0 | 0.01 | 1E-17 | 5.5977E-17 |
| A0ABQ8CXJ8 | Uncharacterized protein (Fragment) OS=Brassica napus OX=3708 GN=HID58_021756 PE=4 SV=1 | 0 | 4.65 | 8 | 2 | 6 | 2 | 419 | 46.9 | 9.28 | 56 | 2 | HID58_021756 | 0 | 0.01 | 1E-17 | 5.5977E-17 |
| A0ABQ8CY15 | Probable Dehydrin protein OS=Brassica napus OX=3708 GN=HID58_022023 PE=4 SV=1 | 0 | 2.105 | 3 | 2 | 11 | 1 | 331 | 37.9 | 5.58 | 37 | 2 | HID58_022023 | 0 | 0.01 | 1E-17 | 5.5977E-17 |
| A0ABQ8CZ51 | Uncharacterized protein OS=Brassica napus OX=3708 GN=HID58_022382 PE=4 SV=1 | 0 | 2.652 | 1 | 1 | 3 | 1 | 1289 | 147.4 | 7.14 | 0 | 1 | HID58_022382 | 0 | 0.01 | 1E-17 | 5.5977E-17 |
| A0ABQ8CZP4 | Probable Pectinesterase protein OS=Brassica napus OX=3708 GN=HID58_022552 PE=4 SV=1 | 0 | 4.208 | 4 | 2 | 23 | 1 | 526 | 57.5 | 8.37 | 440 | 2 | HID58_022552 | 0 | 0.048 | 0.004759179 | 0.023804784 |
| A0ABQ8D036 | Uncharacterized protein OS=Brassica napus OX=3708 GN=HID58_022728 PE=4 SV=1 | 0 | 9.916 | 12 | 4 | 29 | 1 | 350 | 39.5 | 7.69 | 347 | 4 | HID58_022728 | 0 | 0.01 | 1E-17 | 5.5977E-17 |
| A0ABQ8D0M9 | Probable Golgi to ER traffic protein 4 homolog protein OS=Brassica napus OX=3708 GN=HID58_022937 PE=4 SV=1 | 0 | 1.78 | 3 | 1 | 12 | 1 | 334 | 38.3 | 5.77 | 86 | 1 | HID58_022937 | 0 | 0.01 | 1E-17 | 5.5977E-17 |
| A0ABQ8D1J9 | Uncharacterized protein OS=Brassica napus OX=3708 GN=HID58_022930 PE=4 SV=1 | 0 | 3.305 | 2 | 1 | 13 | 1 | 783 | 86.3 | 8.1 | 46 | 1 | HID58_022930 | 0 | 0.01 | 1E-17 | 5.5977E-17 |
| A0ABQ8D1T5 | Probable Nitronate monooxygenase domain-containing protein OS=Brassica napus OX=3708 GN=HID58_023020 PE=4 SV=1 | 0.001 | 1.432 | 3 | 1 | 17 | 1 | 340 | 36.8 | 5.91 | 68 | 1 | HID58_023020 | 0 | 0.01 | 1E-17 | 5.5977E-17 |
| A0ABQ8D233 | Uncharacterized protein OS=Brassica napus OX=3708 GN=HID58_023369 PE=4 SV=1 | 0.004 | 1.194 | 2 | 1 | 5 | 1 | 868 | 96.6 | 7.39 | 0 | 1 | HID58_023369 | 0 | 0.073 | 0.021147278 | 0.092421756 |
| A0ABQ8D2H7 | Probable EF-hand domain-containing protein OS=Brassica napus OX=3708 GN=HID58_023588 PE=4 SV=1 | 0 | 12.322 | 10 | 5 | 23 | 5 | 544 | 62.9 | 5.06 | 179 | 5 | HID58_023588 | 0 | 0.01 | 1E-17 | 5.5977E-17 |
| A0ABQ8D3M2 | Uncharacterized protein OS=Brassica napus OX=3708 GN=HID58_023971 PE=4 SV=1 | 0.005 | 1.176 | 1 | 1 | 2 | 1 | 1300 | 142.2 | 7.53 | 0 | 1 | HID58_023971 | 0 | 0.01 | 1E-17 | 5.5977E-17 |
| A0ABQ8D438 | Probable Casein kinase II subunit beta protein OS=Brassica napus OX=3708 GN=HID58_024158 PE=4 SV=1 | 0.001 | 1.426 | 2 | 1 | 4 | 1 | 529 | 59.1 | 5.1 | 0 | 1 | HID58_024158 | 0 | 0.01 | 1E-17 | 5.5977E-17 |
| A0ABQ8D481 | Probable RimM N-terminal domain-containing protein OS=Brassica napus OX=3708 GN=HID58_024203 PE=4 SV=1 | 0.006 | 1.164 | 2 | 1 | 2 | 1 | 656 | 74.2 | 5.5 | 33 | 1 | HID58_024203 | 0 | 0.01 | 1E-17 | 5.5977E-17 |
| A0ABQ8D4G0 | Uncharacterized protein OS=Brassica napus OX=3708 GN=HID58_023890 PE=4 SV=1 | 0 | 1.495 | 7 | 1 | 7 | 1 | 195 | 22.3 | 9.44 | 98 | 1 | HID58_023890 | 0 | 0.01 | 1E-17 | 5.5977E-17 |
| A0ABQ8D5Y0 | Uncharacterized protein OS=Brassica napus OX=3708 GN=HID58_023908 PE=4 SV=1 | 0 | 2.552 | 2 | 2 | 10 | 2 | 2512 | 287.3 | 7.58 | 39 | 2 | HID58_023908 | 0 | 0.01 | 1E-17 | 5.5977E-17 |
| A0ABQ8D626 | Uncharacterized protein (Fragment) OS=Brassica napus OX=3708 GN=HID58_017066 PE=4 SV=1 | 0 | 5.046 | 1 | 1 | 14 | 1 | 1106 | 120.1 | 8.05 | 227 | 1 | HID58_017066 | 0 | 0.01 | 1E-17 | 5.5977E-17 |
| A0ABQ8D6X3 | Probable Intron-binding protein aquarius OS=Brassica napus OX=3708 GN=HID58_017368 PE=4 SV=1 | 0 | 7.519 | 4 | 4 | 9 | 4 | 1502 | 171.6 | 5.53 | 54 | 4 | HID58_017368 | 0 | 0.01 | 1E-17 | 5.5977E-17 |
| A0ABQ8D7Z8 | Probable Carboxypeptidase protein (Fragment) OS=Brassica napus OX=3708 GN=HID58_017738 PE=4 SV=1 | 0 | 2.908 | 3 | 1 | 4 | 1 | 466 | 52.1 | 8.07 | 18 | 1 | HID58_017738 | 0 | 0.01 | 1E-17 | 5.5977E-17 |
| A0ABQ8D8A6 | Probable VOC domain-containing protein OS=Brassica napus OX=3708 GN=HID58_017861 PE=4 SV=1 | 0 | 3.617 | 12 | 1 | 8 | 1 | 143 | 16.1 | 6.07 | 120 | 1 | HID58_017861 | 0 | 0.01 | 1E-17 | 5.5977E-17 |
| A0ABQ8D8D2 | Uncharacterized protein OS=Brassica napus OX=3708 GN=HID58_017857 PE=4 SV=1 | 0 | 1.729 | 2 | 1 | 2 | 1 | 669 | 76.2 | 9.95 | 0 | 1 | HID58_017857 | 0 | 0.01 | 1E-17 | 5.5977E-17 |
| A0ABQ8D944 | Uncharacterized protein (Fragment) OS=Brassica napus OX=3708 GN=HID58_018161 PE=4 SV=1 | 0 | 1.753 | 3 | 1 | 5 | 1 | 427 | 49.1 | 9.1 | 28 | 1 | HID58_018161 | 0 | 0.01 | 1E-17 | 5.5977E-17 |
| A0ABQ8D969 | Uncharacterized protein OS=Brassica napus OX=3708 GN=HID58_017286 PE=4 SV=1 | 0.001 | 1.429 | 1 | 1 | 1 | 1 | 1094 | 126 | 5.44 | 33 | 1 | HID58_017286 | 0 | 0.01 | 1E-17 | 5.5977E-17 |
| A0ABQ8D9X0 | Uncharacterized protein OS=Brassica napus OX=3708 GN=HID58_018451 PE=4 SV=1 | 0 | 12.925 | 15 | 3 | 28 | 1 | 277 | 30 | 6.7 | 468 | 3 | HID58_018451 | 0 | 0.01 | 1E-17 | 5.5977E-17 |
| A0ABQ8DAD9 | Uncharacterized protein OS=Brassica napus OX=3708 GN=HID58_018577 PE=4 SV=1 | 0.001 | 1.46 | 5 | 1 | 10 | 1 | 264 | 29.6 | 8.7 | 0 | 1 | HID58_018577 | 0 | 0.072 | 0.031746751 | 0.12950112 |
| A0ABQ8DCN4 | Probable VHS domain-containing protein OS=Brassica napus OX=3708 GN=HID58_019390 PE=4 SV=1 | 0.002 | 1.317 | 2 | 1 | 4 | 1 | 752 | 81.8 | 5.26 | 33 | 1 | HID58_019390 | 0 | 0.01 | 1E-17 | 5.5977E-17 |
| A0ABQ8DDG1 | Uncharacterized protein (Fragment) OS=Brassica napus OX=3708 GN=HID58_019676 PE=4 SV=1 | 0.007 | 1.107 | 1 | 1 | 4 | 1 | 1238 | 138.8 | 9.17 | 41 | 1 | HID58_019676 | 0 | 0.01 | 1E-17 | 5.5977E-17 |
| A0ABQ8DDN7 | Uncharacterized protein OS=Brassica napus OX=3708 GN=HID58_019114 PE=4 SV=1 | 0 | 2.989 | 10 | 2 | 11 | 2 | 336 | 35.6 | 9.92 | 20 | 2 | HID58_019114 | 0 | 0.01 | 1E-17 | 5.5977E-17 |
| A0ABQ8DE51 | Probable Phosphoinositide phospholipase C protein OS=Brassica napus OX=3708 GN=HID58_019921 PE=4 SV=1 | 0.006 | 1.133 | 2 | 1 | 8 | 1 | 586 | 66.6 | 6.47 | 26 | 1 | HID58_019921 | 0 | 0.01 | 1E-17 | 5.5977E-17 |
| A0ABQ8DE63 | Uncharacterized protein (Fragment) OS=Brassica napus OX=3708 GN=HID58_019936 PE=4 SV=1 | 0 | 2.937 | 3 | 1 | 10 | 1 | 555 | 58.2 | 8.85 | 70 | 1 | HID58_019936 | 0 | 0.01 | 1E-17 | 5.5977E-17 |
| A0ABQ8DF70 | Probable UTP--glucose-1-phosphate uridylyltransferase protein OS=Brassica napus OX=3708 GN=HID58_020258 PE=4 SV=1 | 0 | 75.945 | 45 | 17 | 264 | 1 | 469 | 51.7 | 5.59 | 3865 | 17 | HID58_020258 | 0 | 0.098 | 0.041241107 | 0.159861513 |
| A0ABQ8DFA4 | Uncharacterized protein OS=Brassica napus OX=3708 GN=HID58_020315 PE=4 SV=1 | 0 | 10.936 | 2 | 4 | 43 | 1 | 1686 | 191.2 | 7.84 | 556 | 4 | HID58_020315 | 0 | 0.01 | 1E-17 | 5.5977E-17 |
| A0ABQ8DFC9 | Probable starch synthase protein OS=Brassica napus OX=3708 GN=HID58_020302 PE=4 SV=1 | 0 | 2.462 | 2 | 1 | 5 | 1 | 841 | 93 | 6.58 | 0 | 1 | HID58_020302 | 0 | 0.01 | 1E-17 | 5.5977E-17 |
| A0ABQ8DFF4 | Uncharacterized protein OS=Brassica napus OX=3708 GN=HID58_019446 PE=4 SV=1 | 0 | 1.655 | 1 | 1 | 8 | 1 | 1456 | 157.9 | 6.05 | 85 | 1 | HID58_019446 | 0 | 0.01 | 1E-17 | 5.5977E-17 |
| A0ABQ8DGQ0 | Probable methionine--tRNA ligase protein OS=Brassica napus OX=3708 GN=HID58_014229 PE=4 SV=1 | 0 | 8.652 | 10 | 5 | 15 | 2 | 643 | 71.3 | 7.46 | 72 | 5 | HID58_014229 | 0 | 0.01 | 1E-17 | 5.5977E-17 |
| A0ABQ8DGR3 | Uncharacterized protein OS=Brassica napus OX=3708 GN=HID58_020209 PE=4 SV=1 | 0.003 | 1.278 | 3 | 1 | 1 | 1 | 615 | 67.6 | 5.81 | 0 | 1 | HID58_020209 | 0 | 0.01 | 1E-17 | 5.5977E-17 |
| A0ABQ8DMK0 | Uncharacterized protein OS=Brassica napus OX=3708 GN=HID58_016313 PE=4 SV=1 | 0 | 2.382 | 1 | 1 | 6 | 1 | 1155 | 128.6 | 6.34 | 42 | 1 | HID58_016313 | 0 | 0.01 | 1E-17 | 5.5977E-17 |
| A0ABQ8DP32 | Uncharacterized protein OS=Brassica napus OX=3708 GN=HID58_008232 PE=4 SV=1 | 0 | 2.607 | 1 | 1 | 3 | 1 | 1255 | 138.3 | 7.9 | 35 | 1 | HID58_008232 | 0 | 0.01 | 1E-17 | 5.5977E-17 |
| A0ABQ8DP47 | Probable ABC1 atypical kinase-like domain-containing protein OS=Brassica napus OX=3708 GN=HID58_008258 PE=4 SV=1 | 0 | 4.702 | 6 | 3 | 8 | 3 | 580 | 65 | 7.03 | 38 | 3 | HID58_008258 | 0 | 0.04 | 0.000469987 | 0.002570414 |
| A0ABQ8DPB3 | Probable FAS1 domain-containing protein OS=Brassica napus OX=3708 GN=HID58_008307 PE=4 SV=1 | 0.002 | 1.36 | 3 | 1 | 10 | 1 | 459 | 50.6 | 7.39 | 106 | 1 | HID58_008307 | 0 | 0.01 | 1E-17 | 5.5977E-17 |
| A0ABQ8DPD6 | Probable GOLD domain-containing protein OS=Brassica napus OX=3708 GN=HID58_008069 PE=4 SV=1 | 0 | 5.359 | 8 | 3 | 8 | 3 | 505 | 58.2 | 5.17 | 40 | 3 | HID58_008069 | 0 | 0.01 | 1E-17 | 5.5977E-17 |
| A0ABQ8DQM7 | Probable Dynamin-type G domain-containing protein OS=Brassica napus OX=3708 GN=HID58_016547 PE=4 SV=1 | 0 | 5.637 | 3 | 2 | 18 | 1 | 631 | 70.2 | 8.37 | 179 | 2 | HID58_016547 | 0 | 0.01 | 1E-17 | 5.5977E-17 |
| A0ABQ8DR46 | Uncharacterized protein OS=Brassica napus OX=3708 GN=HID58_008941 PE=4 SV=1 | 0 | 26.529 | 9 | 5 | 67 | 1 | 820 | 90.7 | 8.5 | 783 | 5 | HID58_008941 | 0 | 0.046 | 0.003990223 | 0.020333529 |
| A0ABQ8DRQ9 | Probable Sialidase domain-containing protein (Fragment) OS=Brassica napus OX=3708 GN=HID58_009169 PE=4 SV=1 | 0 | 2.968 | 3 | 1 | 6 | 1 | 386 | 42.6 | 5.31 | 79 | 1 | HID58_009169 | 0 | 0.01 | 1E-17 | 5.5977E-17 |
| A0ABQ8DTZ3 | Probable Protein VACUOLELESS1 OS=Brassica napus OX=3708 GN=HID58_009946 PE=4 SV=1 | 0 | 1.55 | 1 | 1 | 1 | 1 | 864 | 97.1 | 5.81 | 0 | 1 | HID58_009946 | 0 | 0.01 | 1E-17 | 5.5977E-17 |
| A0ABQ8DUE0 | Probable S-formylglutathione hydrolase protein OS=Brassica napus OX=3708 GN=HID58_010134 PE=4 SV=1 | 0 | 7.204 | 9 | 2 | 26 | 1 | 312 | 35 | 6.28 | 281 | 2 | HID58_010134 | 0 | 0.01 | 1E-17 | 5.5977E-17 |
| A0ABQ8DUT8 | Uncharacterized protein (Fragment) OS=Brassica napus OX=3708 GN=HID58_010216 PE=4 SV=1 | 0 | 2.233 | 3 | 2 | 10 | 2 | 841 | 94.5 | 5.2 | 31 | 2 | HID58_010216 | 0 | 0.01 | 1E-17 | 5.5977E-17 |
| A0ABQ8DV52 | Probable Regulatory particle non-ATPase 13 protein (Fragment) OS=Brassica napus OX=3708 GN=HID58_010389 PE=4 SV=1 | 0 | 5.284 | 8 | 2 | 16 | 2 | 342 | 37.9 | 4.53 | 226 | 2 | HID58_010389 | 0 | 0.01 | 1E-17 | 5.5977E-17 |
| A0ABQ8DVT6 | Probable Plastid lipid-associated protein 1, chloroplastic OS=Brassica napus OX=3708 GN=HID58_010629 PE=4 SV=1 | 0 | 21.421 | 28 | 6 | 37 | 2 | 327 | 35.7 | 5.6 | 410 | 6 | HID58_010629 | 0 | 0.01 | 1E-17 | 5.5977E-17 |
| A0ABQ8DX31 | Probable Glyoxysomal fatty acid beta-oxidation multifunctional protein MFP-a (Fragment) OS=Brassica napus OX=3708 GN=HID58_011049 PE=4 SV=1 | 0 | 14.457 | 10 | 5 | 45 | 1 | 737 | 80.5 | 9.16 | 201 | 5 | HID58_011049 | 0 | 0.01 | 1E-17 | 5.5977E-17 |
| A0ABQ8DX70 | Probable Tryptophan--tRNA ligase, cytoplasmic protein (Fragment) OS=Brassica napus OX=3708 GN=HID58_010945 PE=4 SV=1 | 0 | 4.992 | 6 | 2 | 4 | 2 | 414 | 46.7 | 6.48 | 51 | 2 | HID58_010945 | 0 | 0.01 | 1E-17 | 5.5977E-17 |
| A0ABQ8DXN5 | Uncharacterized protein OS=Brassica napus OX=3708 GN=HID58_011223 PE=4 SV=1 | 0 | 4.665 | 2 | 2 | 50 | 1 | 1452 | 163.4 | 5.71 | 450 | 2 | HID58_011223 | 0 | 0.072 | 0.020161598 | 0.088911858 |
| A0ABQ8E050 | Pobable Pulmonary surfactant-associated protein B (Fragment) OS=Brassica napus OX=3708 GN=HID58_012102 PE=4 SV=1 | 0 | 2.423 | 9 | 1 | 3 | 1 | 218 | 24.9 | 5.6 | 61 | 1 | HID58_012102 | 0 | 0.01 | 1E-17 | 5.5977E-17 |
| A0ABQ8E0E6 | Probable DUF7906 domain-containing protein OS=Brassica napus OX=3708 GN=HID58_012196 PE=4 SV=1 | 0.006 | 1.156 | 2 | 1 | 4 | 1 | 837 | 94.4 | 6.49 | 0 | 1 | HID58_012196 | 0 | 0.01 | 1E-17 | 5.5977E-17 |
| A0ABQ8E205 | Uncharacterized protein OS=Brassica napus OX=3708 GN=HID58_011818 PE=4 SV=1 | 0.002 | 1.347 | 1 | 1 | 2 | 1 | 1114 | 126.7 | 5.53 | 0 | 1 | HID58_011818 | 0 | 0.01 | 1E-17 | 5.5977E-17 |
| A0ABQ8E2C1 | Uncharacterized protein OS=Brassica napus OX=3708 GN=HID58_012881 PE=4 SV=1 | 0 | 13.449 | 11 | 3 | 11 | 1 | 489 | 53.8 | 5.54 | 280 | 3 | HID58_012881 | 0 | 0.01 | 1E-17 | 5.5977E-17 |
| A0ABQ8E424 | Probable glucose-6-phosphate 1-epimerase protein OS=Brassica napus OX=3708 GN=HID58_012572 PE=4 SV=1 | 0.004 | 1.202 | 4 | 1 | 3 | 1 | 312 | 35.2 | 5.87 | 30 | 1 | HID58_012572 | 0 | 0.01 | 1E-17 | 5.5977E-17 |
| A0ABQ8E492 | Probable DUF1682 domain-containing protein OS=Brassica napus OX=3708 GN=HID58_012613 PE=4 SV=1 | 0 | 2.299 | 4 | 1 | 7 | 1 | 465 | 52.3 | 6.11 | 35 | 1 | HID58_012613 | 0 | 0.01 | 1E-17 | 5.5977E-17 |
| A0ABQ8E4D6 | Uncharacterized protein (Fragment) OS=Brassica napus OX=3708 GN=HID58_012677 PE=4 SV=1 | 0 | 3.025 | 1 | 1 | 10 | 1 | 1116 | 123.7 | 7.91 | 41 | 1 | HID58_012677 | 0 | 0.01 | 1E-17 | 5.5977E-17 |
| A0ABQ8E4Q1 | Probable Folylpolyglutamate synthase protein OS=Brassica napus OX=3708 GN=HID58_004058 PE=4 SV=1 | 0 | 3.029 | 2 | 2 | 2 | 2 | 980 | 109.6 | 6.67 | 54 | 2 | HID58_004058 | 0 | 0.024 | 0.000352866 | 0.001941758 |
| A0ABQ8E4T5 | Probable ubiquitinyl hydrolase 1 protein OS=Brassica napus OX=3708 GN=HID58_004075 PE=4 SV=1 | 0 | 23.86 | 13 | 10 | 67 | 1 | 1146 | 134.5 | 5.81 | 734 | 10 | HID58_004075 | 0 | 0.01 | 1E-17 | 5.5977E-17 |
| A0ABQ8E513 | Uncharacterized protein (Fragment) OS=Brassica napus OX=3708 GN=HID58_004182 PE=4 SV=1 | 0 | 45.888 | 9 | 6 | 108 | 1 | 1076 | 113.6 | 8.72 | 2353 | 6 | HID58_004182 | 0 | 0.01 | 1E-17 | 5.5977E-17 |
| A0ABQ8E5D1 | Uncharacterized protein OS=Brassica napus OX=3708 GN=HID58_004297 PE=4 SV=1 | 0 | 7.618 | 1 | 1 | 27 | 1 | 1154 | 129.9 | 5.94 | 690 | 1 | HID58_004297 | 0 | 0.01 | 1E-17 | 5.5977E-17 |
| A0ABQ8E6A0 | Uncharacterized protein OS=Brassica napus OX=3708 GN=HID58_004616 PE=4 SV=1 | 0 | 3.142 | 2 | 1 | 10 | 1 | 853 | 94.4 | 8.37 | 140 | 1 | HID58_004616 | 0 | 0.01 | 1E-17 | 5.5977E-17 |
| A0ABQ8E738 | Uncharacterized protein OS=Brassica napus OX=3708 GN=HID58_004898 PE=4 SV=1 | 0 | 2.361 | 2 | 1 | 8 | 1 | 551 | 57.1 | 9.01 | 23 | 1 | HID58_004898 | 0 | 0.01 | 1E-17 | 5.5977E-17 |
| A0ABQ8E8P8 | Probable Glutaredoxin-dependent peroxiredoxin protein OS=Brassica napus OX=3708 GN=HID58_005456 PE=4 SV=1 | 0 | 5.048 | 11 | 1 | 3 | 1 | 206 | 22.2 | 6.34 | 121 | 1 | HID58_005456 | 0 | 0.01 | 1E-17 | 5.5977E-17 |
| A0ABQ8EA23 | Uncharacterized protein (Fragment) OS=Brassica napus OX=3708 GN=HID58_005945 PE=4 SV=1 | 0 | 4.187 | 2 | 1 | 11 | 1 | 664 | 71.8 | 9.1 | 214 | 1 | HID58_005945 | 0 | 0.01 | 1E-17 | 5.5977E-17 |
| A0ABQ8EAH0 | Probable Proteasome subunit beta protein OS=Brassica napus OX=3708 GN=HID58_006114 PE=4 SV=1 | 0 | 9.621 | 23 | 3 | 31 | 2 | 204 | 22.8 | 5.68 | 82 | 3 | HID58_006114 | 1 | 0.01 | 1E-17 | 5.5977E-17 |
| A0ABQ8EAX0 | Probable BRCT domain-containing protein OS=Brassica napus OX=3708 GN=HID58_006230 PE=4 SV=1 | 0 | 6.558 | 4 | 4 | 20 | 1 | 1244 | 140.8 | 6.98 | 51 | 4 | HID58_006230 | 0 | 0.01 | 1E-17 | 5.5977E-17 |
| A0ABQ8EAY0 | Uncharacterized protein OS=Brassica napus OX=3708 GN=HID58_005419 PE=4 SV=1 | 0 | 4.276 | 2 | 1 | 5 | 1 | 790 | 90.5 | 7.43 | 54 | 1 | HID58_005419 | 0 | 0.01 | 1E-17 | 5.5977E-17 |
| A0ABQ8ED10 | Probable Complex III subunit VI (Fragment) OS=Brassica napus OX=3708 GN=HID58_006988 PE=4 SV=1 | 0.007 | 1.086 | 7 | 1 | 3 | 1 | 130 | 14.7 | 6.98 | 29 | 1 | HID58_006988 | 0 | 0.01 | 1E-17 | 5.5977E-17 |
| A0ABQ8ED27 | Probable GST C-terminal domain-containing protein (Fragment) OS=Brassica napus OX=3708 GN=HID58_006723 PE=4 SV=1 | 0 | 2.457 | 6 | 2 | 11 | 1 | 335 | 38.3 | 6.46 | 39 | 2 | HID58_006723 | 0 | 0.01 | 1E-17 | 5.5977E-17 |
| A0ABQ8EDM9 | Uncharacterized protein OS=Brassica napus OX=3708 GN=HID58_007238 PE=4 SV=1 | 0.005 | 1.182 | 1 | 1 | 1 | 1 | 1616 | 178.8 | 7.34 | 0 | 1 | HID58_007238 | 0 | 0.01 | 1E-17 | 5.5977E-17 |
| A0ABQ8EFX4 | Uncharacterized protein OS=Brassica napus OX=3708 GN=HID58_000231 PE=4 SV=1 | 0 | 1.538 | 2 | 1 | 1 | 1 | 532 | 59.9 | 6.99 | 29 | 1 | HID58_000231 | 0 | 0.01 | 1E-17 | 5.5977E-17 |
| A0ABQ8EGF2 | Probable Catalase protein OS=Brassica napus OX=3708 GN=HID58_000313 PE=4 SV=1 | 0 | 38.201 | 28 | 10 | 144 | 1 | 461 | 53.4 | 7.09 | 1350 | 10 | HID58_000313 | 0 | 0.01 | 1E-17 | 5.5977E-17 |
| A0ABQ8EHI4 | Probable threonine synthase protein OS=Brassica napus OX=3708 GN=HID58_000716 PE=4 SV=1 | 0 | 12.758 | 9 | 4 | 44 | 4 | 527 | 58 | 7.62 | 306 | 4 | HID58_000716 | 0 | 0.046 | 0.006660392 | 0.032289339 |
| A0ABQ8EHJ5 | Uncharacterized protein OS=Brassica napus OX=3708 GN=HID58_007684 PE=4 SV=1 | 0 | 8.418 | 14 | 1 | 9 | 1 | 121 | 13.7 | 10.21 | 77 | 1 | HID58_007684 | 0 | 0.01 | 1E-17 | 5.5977E-17 |
| A0ABQ8EI45 | Uncharacterized protein OS=Brassica napus OX=3708 GN=HID58_000978 PE=4 SV=1 | 0 | 1.917 | 3 | 1 | 1 | 1 | 480 | 54.9 | 5.88 | 0 | 1 | HID58_000978 | 0 | 0.01 | 1E-17 | 5.5977E-17 |
| A0ABQ8EID7 | Probable Methyltransferase protein OS=Brassica napus OX=3708 GN=HID58_000988 PE=4 SV=1 | 0 | 1.795 | 2 | 1 | 5 | 1 | 707 | 81 | 7.14 | 44 | 1 | HID58_000988 | 0 | 0.01 | 1E-17 | 5.5977E-17 |
| A0ABQ8EKW9 | Probable Pyruvate, phosphate dikinase OS=Brassica napus OX=3708 GN=HID58_001966 PE=4 SV=1 | 0 | 43.262 | 18 | 14 | 180 | 1 | 956 | 104.1 | 6.19 | 1458 | 14 | HID58_001966 | 0 | 0.01 | 1E-17 | 5.5977E-17 |
| A0ABQ8EQY2 | Uncharacterized protein OS=Brassica napus OX=3708 GN=HID58_003515 PE=4 SV=1 | 0 | 14.853 | 4 | 6 | 67 | 2 | 1659 | 192.7 | 5.72 | 1216 | 6 | HID58_003515 | 0 | 0.041 | 0.005630382 | 0.027792632 |
| P93657 | L-ascorbate peroxidase OS=Brassica napus OX=3708 GN=APX PE=2 SV=1 | 0 | 29.364 | 26 | 4 | 67 | 1 | 250 | 27.6 | 6.07 | 1634 | 4 | APX | 0 | 0.1 | 0.048745388 | 0.184576104 |
| Q2I0E4 | Chlorophyll a-b binding protein, chloroplastic (Fragment) OS=Brassica napus OX=3708 GN=LHB1B2 PE=2 SV=1 | 0 | 13.627 | 59 | 3 | 84 | 1 | 75 | 8.2 | 4.78 | 1843 | 3 | LHB1B2 | 0 | 0.01 | 1E-17 | 5.5977E-17 |
| Q42623 | Glutamine synthetase OS=Brassica napus OX=3708 GN=gln PE=2 SV=1 | 0 | 38.955 | 31 | 6 | 81 | 1 | 356 | 39.1 | 5.47 | 987 | 6 | gln; GLN1.1 | 1 | 0.01 | 1E-17 | 5.5977E-17 |
| Q42634 | Phosphoenolpyruvate carboxylase OS=Brassica napus OX=3708 GN=PE3-PEPCase PE=3 SV=1 | 0 | 66.83 | 30 | 25 | 191 | 11 | 964 | 109.6 | 6.42 | 1650 | 25 | PE3-PEPCase | 16 | 0.101 | 0.045531348 | 0.174070427 |
| Q4PJU0 | Peroxidase OS=Brassica napus OX=3708 GN=DARMORV10_C08P24120.1 PE=2 SV=1 | 0 | 6.117 | 10 | 3 | 24 | 1 | 354 | 38.8 | 6.24 | 144 | 3 | DARMORV10_C08P24120.1 | 0 | 0.01 | 1E-17 | 5.5977E-17 |
| Q6RJS1 | Eukaryotic translation initiation factor 5A OS=Brassica napus OX=3708 PE=2 SV=1 | 0 | 23.323 | 44 | 7 | 61 | 2 | 159 | 17.1 | 6.14 | 682 | 7 |  | 0 | 0.01 | 1E-17 | 5.5977E-17 |
| Q9M4R9 | Tubulin beta chain (Fragment) OS=Brassica napus OX=3708 PE=2 SV=1 | 0 | 65.507 | 47 | 12 | 254 | 1 | 323 | 36.1 | 6.02 | 3185 | 12 |  | 0 | 0.01 | 1E-17 | 5.5977E-17 |
|  |  |  |  |  |  |  |  |  |  |  |  |  |  |  |  |  |  |

**Supplementary Table S2** – Functional classification of over-represented proteins annotated with Mercator4. The table lists each protein’s functional BIN assignment, gene identifier, accession number and descriptive annotation.

| **NAME** | **Gene Name** | **Accession** | **Description** |
| --- | --- | --- | --- |
| Amino acid metabolism.amino acid degradation.cysteine degradation.mercaptopyruvate sulfurtransferase *(MST1/2) | HID58_095721 | A0ABQ7X2K8 | Uncharacterized protein (Fragment) OS=Brassica napus OX=3708 GN=HID58_095721 PE=4 SV=1 |
| Amino acid metabolism.aspartate group amino acid biosynthesis.aspartate-derived amino acids.methionine biosynthesis.L-homocysteine S-methyltransferase activities.methyl-tetrahydrofolate-dependent methionine synthase *(MS) | DARMORV10_A03P33420.1 | A0A816WHH5 | 5-Methyltetrahydropteroyltriglutamate--homocysteine S-methyltransferase OS=Brassica napus OX=3708 GN=DARMORV10_A03P33420.1 PE=3 SV=1 |
| Amino acid metabolism.aspartate group amino acid biosynthesis.aspartate-derived amino acids.methionine biosynthesis.L-homocysteine S-methyltransferase activities.methyl-tetrahydrofolate-dependent methionine synthase *(MS) | HID58_088555 | A0ABQ7XXW7 | Uncharacterized protein (Fragment) OS=Brassica napus OX=3708 GN=HID58_088555 PE=4 SV=1 |
| Amino acid metabolism.glutamate group amino acid biosynthesis.glutamate-derived amino acids.arginine biosynthesis.argininosuccinate synthetase | HID58_041698 | A0ABQ8BBJ8 | Uncharacterized protein OS=Brassica napus OX=3708 GN=HID58_041698 PE=4 SV=1 |
| Amino acid metabolism.glutamate group amino acid biosynthesis.glutamate-derived amino acids.gamma-aminobutyrate (GABA).glutamate decarboxylase *(GAD) | DARMORV10_C04P16840.1 | A0A816JSP8 | Glutamate decarboxylase OS=Brassica napus OX=3708 GN=DARMORV10_C04P16840.1 PE=3 SV=1 |
| Amino acid metabolism.glutamate group amino acid biosynthesis.glutamate-derived amino acids.ornithine biosynthesis.N-acetylglutamate kinase complex.regulatory PII nitrogen sensor protein *(GLB1) | BnaCnng57780D | A0A078JNV6 | Nitrogen regulatory protein P-II, (rape) hypothetical protein OS=Brassica napus OX=3708 GN=BnaCnng57780D PE=4 SV=1 |
| Amino acid metabolism.glutamate group amino acid biosynthesis.glutamate-derived amino acids.ornithine biosynthesis.N-acetylglutamate kinase complex.regulatory PII nitrogen sensor protein *(GLB1) | DARMORV10_C03P38310.1 | A0A816I3S1 | Nitrogen regulatory protein P-II, (rape) hypothetical protein OS=Brassica napus OX=3708 GN=DARMORV10_C03P38310.1 PE=3 SV=1 |
| Carbohydrate metabolism.plastidial glycolysis | HID58_026060 | A0ABQ8CPU6 | Glucose-6-phosphate 1-epimerase, Uncharacterized protein OS=Brassica napus OX=3708 GN=HID58_026060 PE=4 SV=1 |
| Carbohydrate metabolism.plastidial glycolysis.fructose-1,6-bisphosphate aldolase *(FBA1) | BnaC02g33660D | A0A078HKF7 | Fructose-bisphosphate aldolase OS=Brassica napus OX=3708 GN=BnaC02g33660D PE=3 SV=1 |
| Carbohydrate metabolism.sorbitol metabolism.sorbitol dehydrogenase | BnaCnng32750D | A0A078J5R3 | Sorbitol dehydrogenase, (rape) hypothetical protein OS=Brassica napus OX=3708 GN=BnaCnng32750D PE=3 SV=1 |
| Carbohydrate metabolism.sucrose metabolism.regulation of sucrose/starch partitioning.bifunctional 6-phosphofructo-2-kinase and fructose-2,6-bisphosphatase | DARMORV10_A09P66600.1 | A0A816PTZ9 | 6-phosphofructo-2-kinase/fructose-2, 6-bisphosphatase, (rape) hypothetical protein OS=Brassica napus OX=3708 GN=DARMORV10_A09P66600.1 PE=4 SV=1 |
| Cell division.cytokinesis.preprophase microtubule organization.TON1-TRM-PP2A (TTP) preprophase band formation complex.component *(TRM) | DARMORV10_C03P39940.1 | A0A816I9B4 | Protein LONGIFOLIA 2, (rape) hypothetical protein OS=Brassica napus OX=3708 GN=DARMORV10_C03P39940.1 PE=4 SV=1 |
| Cell division.meiotic recombination.meiotic crossover.class-I/ZMM crossover pathway.HEI10 regulatory E3 ubiquitin ligase activity.regulatory co-chaperone *(HCR3) | DARMORV10_A03P10570.1 | A0A816VBH8 | Chaperone protein dnaJ 3-like, (rape) hypothetical protein OS=Brassica napus OX=3708 GN=DARMORV10_A03P10570.1 PE=3 SV=1 |
| Cell wall organisation.cutin and suberin.cuticular lipid formation.alkane-forming pathway.electron supply module.electron shuttle hemoprotein *(Cyt-b5) | BnaC04g12100D | A0A078FCT5 | Cytochrome b5 family protein, BnaC04g12100D protein OS=Brassica napus OX=3708 GN=BnaC04g12100D PE=3 SV=1 |
| Cell wall organisation.cutin and suberin.cuticular lipid formation.alkane-forming pathway.electron supply module.electron shuttle hemoprotein *(Cyt-b5) | HID58_087622 | A0ABQ7XWC2 | Uncharacterized protein OS=Brassica napus OX=3708 GN=HID58_087622 PE=4 SV=1 |
| Cell wall organisation.cutin and suberin.export.cuticular lipid transfer accessory factor *(LTPG) | DARMORV10_A07P11530.1 | A0A816YN54 | Non-specific lipid transfer protein GPI-anchored 1, (rape) hypothetical protein OS=Brassica napus OX=3708 GN=DARMORV10_A07P11530.1 PE=3 SV=1 |
| Cell wall organisation.hemicellulose.xylan.modification and degradation.glucuronoxylan 4-O-methyltransferase *(GXM1/2/3) | HID58_031080 | A0ABQ8CHS6 | Uncharacterized protein (Fragment) OS=Brassica napus OX=3708 GN=HID58_031080 PE=4 SV=1 |
| Cellular respiration.oxidative phosphorylation.ATP synthase complex.peripheral MF1 subcomplex.subunit delta_ | BnaAnng07210D | A0A078I1G2 | ATP synthase subunit delta', mitochondrial OS=Brassica napus OX=3708 GN=BnaAnng07210D PE=3 SV=1 |
| Cellular respiration.oxidative phosphorylation.cytochrome c reductase complex.apocytochrome b component | cob | F8K8R8 | Cytochrome b OS=Brassica napus OX=3708 GN=cob PE=3 SV=1 |
| Cellular respiration.oxidative phosphorylation.NADH dehydrogenase complex | HID58_008869 | A0ABQ8DQV7 | NAD(P)H dehydrogenase (quinone), Uncharacterized protein OS=Brassica napus OX=3708 GN=HID58_008869 PE=4 SV=1 |
| Cellular respiration.oxidative phosphorylation.NADH dehydrogenase complex.non-core modules.component *(C1-Fd) | HID58_068752 | A0ABQ7ZMD4 | Uncharacterized protein OS=Brassica napus OX=3708 GN=HID58_068752 PE=4 SV=1 |
| Cellular respiration.tricarboxylic acid cycle.aconitase *(ACO) | HID58_074716 | A0ABQ7YHK3 | Uncharacterized protein OS=Brassica napus OX=3708 GN=HID58_074716 PE=4 SV=1 |
| Cellular respiration.tricarboxylic acid cycle.NAD-dependent malate dehydrogenase *(mMDH1/2) | MDH | Q43744 | Malate dehydrogenase, mitochondrial OS=Brassica napus OX=3708 GN=MDH PE=2 SV=1 |
| Chromatin organisation.chromatin structure.DNA bridging.linker histone *(H1) | DARMORV10_C04P59660.1 | A0A816JVR0 | Histone H1.2, (rape) hypothetical protein OS=Brassica napus OX=3708 GN=DARMORV10_C04P59660.1 PE=3 SV=1 |
| Chromatin organisation.chromatin structure.DNA wrapping.histone H2A-H2B dimer.histone *(H2A/HTA) | HID58_066592 | A0ABQ7ZG33 | Uncharacterized protein OS=Brassica napus OX=3708 GN=HID58_066592 PE=4 SV=1 |
| Chromatin organisation.chromatin structure.DNA wrapping.histone H2A-H2B dimer.histone *(H2A/HTA) | HID58_018225 | A0ABQ8D9A6 | Uncharacterized protein OS=Brassica napus OX=3708 GN=HID58_018225 PE=4 SV=1 |
| Chromatin organisation.histone chaperone activities.FACT histone chaperone complex.component *(SPT16) | DARMORV10_C03P35150.1 | A0A816IES6 | FACT complex subunit OS=Brassica napus OX=3708 GN=DARMORV10_C03P35150.1 PE=3 SV=1 |
| Chromatin organisation.post-translational histone modification.PRC1 bifunctional histone ubiquitination and methylation reader complex.associated factors.regulatory protein *(EMF1) | DARMORV10_C03P05810.1 | A0A816HVR2 | Malic enzyme OS=Brassica napus OX=3708 GN=DARMORV10_C03P05810.1 PE=3 SV=1 |
| Clade-specific metabolism.Brassicaceae.glucosinolate degradation.epithiospecifier protein *(ESP) | BnaAnng10080D | A0A078II13 | Epithiospecifier protein-like, BnaAnng10080D protein OS=Brassica napus OX=3708 GN=BnaAnng10080D PE=4 SV=1 |
| Clade-specific metabolism.Brassicaceae.glucosinolate degradation.myrosinase *(TGG) | DARMORV10_A01P22620.1 | A0A816XUS5 | Myrosinase 2-like (Fragment) OS=Brassica napus OX=3708 GN=DARMORV10_A01P22620.1 PE=3 SV=1 |
| Clade-specific metabolism.Brassicaceae.glucosinolate degradation.nitrilespecifier protein *(NSP) | HID58_003224 | A0ABQ8EPH6 | Uncharacterized protein (Fragment) OS=Brassica napus OX=3708 GN=HID58_003224 PE=4 SV=1 |
| Cytoskeleton organisation.actin and tubulin folding.post-CCT Tubulin folding pathway.TFC tubulin folding cofactors.cofactor *(TFC-A) | BnaA05g12000D | A0A078FBJ6 | Tubulin-specific chaperone A OS=Brassica napus OX=3708 GN=BnaA05g12000D PE=3 SV=1 |
| Cytoskeleton organisation.actin and tubulin folding.TRiC/CCT chaperonin folding complex.subunit alpha *(CCT1) | DARMORV10_C03P50870.1 | A0A816I9U9 | T-complex protein 1 subunit alpha OS=Brassica napus OX=3708 GN=DARMORV10_C03P50870.1 PE=3 SV=1 |
| Cytoskeleton organisation.microfilament network.actin organisation.regulatory protein *(PGSL1) | HID58_032234 | A0ABQ8BVX5 | Uncharacterized protein (Fragment) OS=Brassica napus OX=3708 GN=HID58_032234 PE=4 SV=1 |
| Cytoskeleton organisation.microfilament network.myosin microfilament-based motor protein activities.class XI myosin motor protein | DARMORV10_C05P03650.1 | A0A816L182 | Myosin-6, (rape) hypothetical protein OS=Brassica napus OX=3708 GN=DARMORV10_C05P03650.1 PE=4 SV=1 |
| Cytoskeleton organisation.microfilament network.myosin microfilament-based motor protein activities.class XI myosin motor protein | DARMORV10_A06P05650.1 | A0A816S7X4 | Myosin, (rape) hypothetical protein OS=Brassica napus OX=3708 GN=DARMORV10_A06P05650.1 PE=3 SV=1 |
| DNA damage response.nucleotide excision repair (NER).preincision complex formation.CAK kinase module.scaffold component *(TFB3) | HID58_029906 | A0ABQ8CGH1 | Carboxypeptidase, Uncharacterized protein OS=Brassica napus OX=3708 GN=HID58_029906 PE=4 SV=1 |
| External stimuli response.pathogen.defense mechanisms | BnaC02g20340D | A0A078F8S4 | Pathogenesis-related protein Bet v I family, (rape) hypothetical protein OS=Brassica napus OX=3708 GN=BnaC02g20340D PE=4 SV=1 |
| External stimuli response.pathogen.defense mechanisms.defense enzyme activities.pathogen polygalacturonase inhibitor *(PGIP1/2) | DARMORV10_C07P52750.1 | A0A816N2A6 | Polygalacturonase, (rape) hypothetical protein OS=Brassica napus OX=3708 GN=DARMORV10_C07P52750.1 PE=3 SV=1 |
| External stimuli response.pathogen.defense mechanisms.induction of callose deposition | DARMORV10_C04P17390.1 | A0A816JS97 | COP1-interactive protein 1, (rape) hypothetical protein OS=Brassica napus OX=3708 GN=DARMORV10_C04P17390.1 PE=4 SV=1 |
| External stimuli response.toxic compounds.reactive aldehyde detoxification.aldo-keto reductase *(AKR4C8/9) | DARMORV10_C04P10080.1 | A0A816JC23 | Aldo-keto reductase family 4 member C10, (rape) hypothetical protein OS=Brassica napus OX=3708 GN=DARMORV10_C04P10080.1 PE=3 SV=1 |
| Lipid metabolism.fatty acid metabolism.acetyl-CoA generation.plastidial pyruvate dehydrogenase complex.E1 pyruvate dehydrogenase subcomplex.subunit alpha | BnaA09g51510D | A0A078H163 | Pyruvate dehydrogenase E1 component subunit alpha OS=Brassica napus OX=3708 GN=BnaA09g51510D PE=4 SV=1 |
| Lipid metabolism.fatty acid metabolism.citrate shuttle.cytosolic NAD-dependent malate dehydrogenase | BnaA09g16400D | A0A078H7S5 | Malate dehydrogenase OS=Brassica napus OX=3708 GN=BnaA09g16400D PE=3 SV=1 |
| Lipid metabolism.fatty acid metabolism.citrate shuttle.cytosolic NADP-dependent malic enzyme | DARMORV10_C03P05810.1 | A0A816HVR2 | Malic enzyme OS=Brassica napus OX=3708 GN=DARMORV10_C03P05810.1 PE=3 SV=1 |
| Lipid metabolism.fatty acid metabolism.fatty acid degradation.glyoxylate cycle.aconitase | HID58_074716 | A0ABQ7YHK3 | Uncharacterized protein OS=Brassica napus OX=3708 GN=HID58_074716 PE=4 SV=1 |
| Lipid metabolism.fatty acid metabolism.fatty acid desaturation.electron supply.cytochrome electron shuttle hemoprotein *(Cyt-b5) | BnaC04g12100D | A0A078FCT5 | Cytochrome b5 family protein, BnaC04g12100D protein OS=Brassica napus OX=3708 GN=BnaC04g12100D PE=3 SV=1 |
| Lipid metabolism.fatty acid metabolism.fatty acid desaturation.electron supply.cytochrome electron shuttle hemoprotein *(Cyt-b5) | HID58_087622 | A0ABQ7XWC2 | Uncharacterized protein OS=Brassica napus OX=3708 GN=HID58_087622 PE=4 SV=1 |
| Multi-process regulation.14-3-3 regulatory system.14-3-3 phosphoprotein-binding protein *(GRF) | BnaA06g24300D | A0A078F6Y1 | 14-3-3-like protein GF14 kappa, (rape) hypothetical protein OS=Brassica napus OX=3708 GN=BnaA06g24300D PE=3 SV=1 |
| Multi-process regulation.14-3-3 regulatory system.14-3-3 phosphoprotein-binding protein *(GRF) | BnaCnng05840D | A0A078GVL1 | 14-3-3 family protein, BnaCnng05840D protein OS=Brassica napus OX=3708 GN=BnaCnng05840D PE=3 SV=1 |
| Multi-process regulation.14-3-3 regulatory system.14-3-3 phosphoprotein-binding protein *(GRF) | DARMORV10_A04P11450.1 | A0A817AJH6 | 14-3-3-like protein GF14 psi, (rape) hypothetical protein OS=Brassica napus OX=3708 GN=DARMORV10_A04P11450.1 PE=3 SV=1 |
| Multi-process regulation.14-3-3 regulatory system.14-3-3 phosphoprotein-binding protein *(GRF) | HID58_025850 | A0ABQ8CP86 | 14-3-3 domain-containing protein, Uncharacterized protein OS=Brassica napus OX=3708 GN=HID58_025850 PE=4 SV=1 |
| Multi-process regulation.calcium homeostasis.Ca2+-dependent signalling.CML-type calcium sensor activities.calcium sensor *(CML2/3/++) | HID58_068752 | A0ABQ7ZMD4 | Uncharacterized protein OS=Brassica napus OX=3708 GN=HID58_068752 PE=4 SV=1 |
| Multi-process regulation.calcium homeostasis.Ca2+-dependent signalling.phosphoinositide signalling effector *(PCaP1/2) | HID58_093241 | A0ABQ7XBQ1 | Plasma membrane-associated cation-binding protein 1, Uncharacterized protein OS=Brassica napus OX=3708 GN=HID58_093241 PE=4 SV=1 |
| Multi-process regulation.calcium homeostasis.Ca2+-dependent signalling.phosphoinositide signalling effector *(PCaP1/2) | HID58_001076 | A0ABQ8EII9 | Plasma-membrane associated cation-binding protein 1, Uncharacterized protein OS=Brassica napus OX=3708 GN=HID58_001076 PE=4 SV=1 |
| Multi-process regulation.ROP-GTPase regulatory system.RopGAP GTPase-activating activities.regulatory protein *(ARO) of RenGAP activity | HID58_092470 | A0ABQ7X484 | Ferredoxin-NADP(+) reductase, Uncharacterized protein OS=Brassica napus OX=3708 GN=HID58_092470 PE=4 SV=1 |
| Multi-process regulation.TOR (Target Of Rapamycin) signalling.TOR kinase substrates.TOR-dependent regulatory protein *(TAP46) of protein phosphatase complex | HID58_038149 | A0ABQ8BP75 | PP2A regulatory subunit TAP46, Uncharacterized protein (Fragment) OS=Brassica napus OX=3708 GN=HID58_038149 PE=4 SV=1 |
| not assigned.annotated | DARMORV10_C01P11880.1 | A0A816R5K5 | Methyltransferase OS=Brassica napus OX=3708 GN=DARMORV10_C01P11880.1 PE=3 SV=1 |
| not assigned.annotated | DARMORV10_A04P06430.1 | A0A817AJF9 | Late embryogenesis abundant protein At3g53040-like, (rape) hypothetical protein OS=Brassica napus OX=3708 GN=DARMORV10_A04P06430.1 PE=4 SV=1 |
| not assigned.annotated | HID58_080171 | A0ABQ7Y453 | Uncharacterized protein (Fragment) OS=Brassica napus OX=3708 GN=HID58_080171 PE=4 SV=1 |
| not assigned.annotated | HID58_070400 | A0ABQ7YYN6 | Uncharacterized protein OS=Brassica napus OX=3708 GN=HID58_070400 PE=4 SV=1 |
| not assigned.annotated | HID58_070541 | A0ABQ7YZ18 | TSK-associating protein 1-like, Uncharacterized protein OS=Brassica napus OX=3708 GN=HID58_070541 PE=4 SV=1 |
| not assigned.annotated | HID58_049733 | A0ABQ8B5W1 | Uncharacterized protein OS=Brassica napus OX=3708 GN=HID58_049733 PE=4 SV=1 |
| not assigned.annotated | HID58_036330 | A0ABQ8C7G1 | Uncharacterized protein OS=Brassica napus OX=3708 GN=HID58_036330 PE=4 SV=1 |
| not assigned.annotated | HID58_003259 | A0ABQ8EPM1 | TSA1-like protein, Uncharacterized protein OS=Brassica napus OX=3708 GN=HID58_003259 PE=4 SV=1 |
| not assigned.not annotated | BnaAnng35040D | A0A078JTB5 | Late embryogenesis abundant protein, group 3, (rape) hypothetical protein OS=Brassica napus OX=3708 GN=BnaAnng35040D PE=4 SV=1 |
| not assigned.not annotated | DARMORV10_C09P66120.1 | A0A816J6H8 | Uncharacterized LOC106412569 (rape) hypothetical protein OS=Brassica napus OX=3708 GN=DARMORV10_C09P66120.1 PE=4 SV=1 |
| not assigned.not annotated | DARMORV10_A07P39160.1 | A0A816ZKS5 | Uncharacterized BNAA07G30660D, (rape) hypothetical protein OS=Brassica napus OX=3708 GN=DARMORV10_A07P39160.1 PE=4 SV=1 |
| not assigned.not annotated | DARMORV10_A08P04600.1 | A0A816ZXB2 | (rape) hypothetical protein OS=Brassica napus OX=3708 GN=DARMORV10_A08P04600.1 PE=4 SV=1 |
| not assigned.not annotated | HID58_013572 | A0ABQ8E497 | TPX2 C-terminal domain-containing protein, Uncharacterized protein OS=Brassica napus OX=3708 GN=HID58_013572 PE=4 SV=1 |
| Nucleotide metabolism.deoxynucleotides.salvage pathway.nucleoside diphosphate kinase | BnaA09g17360D | A0A078FIU2 | Nucleoside diphosphate kinase OS=Brassica napus OX=3708 GN=BnaA09g17360D PE=3 SV=1 |
| Nutrient uptake.iron uptake.chelation-based strategy.Fe(III)-phytosiderophore uptake transporter *(YSL1) | HID58_087622 | A0ABQ7XWC2 | Uncharacterized protein OS=Brassica napus OX=3708 GN=HID58_087622 PE=4 SV=1 |
| Nutrient uptake.iron uptake.iron homeostasis.ILR3 transcriptional regulator activity.bHLH-IVc-class transcriptional regulator *(ILR3) | HID58_066592 | A0ABQ7ZG33 | Uncharacterized protein OS=Brassica napus OX=3708 GN=HID58_066592 PE=4 SV=1 |
| Nutrient uptake.iron uptake.iron homeostasis.iron storage protein *(FER) | BnaA03g00260D | A0A078F9R8 | Ferritin OS=Brassica napus OX=3708 GN=BnaA03g00260D PE=3 SV=1 |
| Nutrient uptake.nitrogen assimilation.ammonium assimilation.glutamine synthetase activities.cytosolic glutamine synthetase *(GLN1) | HID58_069571 | A0ABQ7YW91 | Glutamine synthetase, Uncharacterized protein OS=Brassica napus OX=3708 GN=HID58_069571 PE=4 SV=1 |
| Nutrient uptake.nitrogen assimilation.ammonium assimilation.glutamine synthetase activities.cytosolic glutamine synthetase *(GLN1) | BnaCnng23850D | O04852 | Glutamine synthetase OS=Brassica napus OX=3708 GN=BnaCnng23850D PE=3 SV=1 |
| Nutrient uptake.nitrogen assimilation.aspartate aminotransferase *(ASP) | HID58_088682 | A0ABQ7XX08 | Aspartate aminotransferase, Uncharacterized protein OS=Brassica napus OX=3708 GN=HID58_088682 PE=4 SV=1 |
| Photosynthesis.calvin cycle.fructose-1,6-bisphosphate aldolase | BnaC02g33660D | A0A078HKF7 | Fructose-bisphosphate aldolase OS=Brassica napus OX=3708 GN=BnaC02g33660D PE=3 SV=1 |
| Photosynthesis.calvin cycle.ribulose-1,5-bisphosphat carboxylase/oxygenase (RuBisCo) activity.RuBisCo assembly.CPN60 chaperonin heterodimer.subunit alpha | BnaA04g16190D | A0A078GZH9 | Chaperonin (HSP60) (rape) hypothetical protein OS=Brassica napus OX=3708 GN=BnaA04g16190D PE=3 SV=1 |
| Photosynthesis.calvin cycle.ribulose-1,5-bisphosphat carboxylase/oxygenase (RuBisCo) activity.RuBisCo heterodimer.large subunit | BnaCnng12440D | A0A078I3R9 | Ribulose bisphosphate carboxylase large chain, BnaCnng12440D protein OS=Brassica napus OX=3708 GN=BnaCnng12440D PE=4 SV=1 |
| Photosynthesis.calvin cycle.ribulose-1,5-bisphosphat carboxylase/oxygenase (RuBisCo) activity.RuBisCo heterodimer.small subunit | BnaCnng55860D | A0A078JQI2 | Ribulose bisphosphate carboxylase small subunit, chloroplastic OS=Brassica napus OX=3708 GN=BnaCnng55860D PE=3 SV=1 |
| Photosynthesis.calvin cycle.ribulose-1,5-bisphosphat carboxylase/oxygenase (RuBisCo) activity.RuBisCo heterodimer.small subunit | RBCS | A0A816K122 | Ribulose bisphosphate carboxylase small subunit, chloroplastic OS=Brassica napus OX=3708 GN=RBCS PE=3 SV=1 |
| Photosynthesis.CAM/C4 photosynthesis.NAD-dependent malate dehydrogenase | BnaA09g16400D | A0A078H7S5 | Malate dehydrogenase OS=Brassica napus OX=3708 GN=BnaA09g16400D PE=3 SV=1 |
| Photosynthesis.photophosphorylation.chlororespiration.NADH dehydrogenase-like (NDH) complex.assembly and maintenance.Cpn60 chaperonin heterodimer.subunit alpha | BnaA04g16190D | A0A078GZH9 | Chaperonin (HSP60) (rape) hypothetical protein OS=Brassica napus OX=3708 GN=BnaA04g16190D PE=3 SV=1 |
| Photosynthesis.photophosphorylation.linear electron flow.ferredoxin-NADP reductase activity.ferredoxin-NADP oxidoreductase *(FNR1/2) | HID58_030700 | A0ABQ8CGP7 | Uncharacterized protein OS=Brassica napus OX=3708 GN=HID58_030700 PE=4 SV=1 |
| Photosynthesis.photophosphorylation.photosystem I.assembly and maintenance | BnaA07g07570D | A0A078HZ79 | Chlorophyll a-b binding protein, chloroplastic OS=Brassica napus OX=3708 GN=BnaA07g07570D PE=3 SV=1 |
| Photosynthesis.photophosphorylation.photosystem I.assembly and maintenance.assembly chaperone *(PSA2) | HID58_062910 | A0ABQ8A2S7 | Uncharacterized protein OS=Brassica napus OX=3708 GN=HID58_062910 PE=4 SV=1 |
| Photosynthesis.photophosphorylation.photosystem I.PS-I complex.component *(PsaE) | DARMORV10_C01P10750.1 | A0A816R943 | Photosystem I reaction center subunit IV A, chloroplastic, (rape) hypothetical protein OS=Brassica napus OX=3708 GN=DARMORV10_C01P10750.1 PE=3 SV=1 |
| Photosynthesis.photophosphorylation.photosystem II.assembly.assembly factor *(TLP7-6) | BnaA06g15210D | A0A078ILM3 | Uncharacterized LOC106347363, (rape) hypothetical protein OS=Brassica napus OX=3708 GN=BnaA06g15210D PE=4 SV=1 |
| Photosynthesis.photophosphorylation.photosystem II.photosynthetic acclimation.thylakoid grana stacking.regulatory factor *(CURT1) | DARMORV10_C03P38930.1 | A0A816I7Y6 | Protein CURVATURE THYLAKOID 1A, chloroplastic, (rape) hypothetical protein OS=Brassica napus OX=3708 GN=DARMORV10_C03P38930.1 PE=4 SV=1 |
| Photosynthesis.photophosphorylation.photosystem II.PS-II complex.component *(PsbL) | psbL | A0A1B1XZH5 | Photosystem II reaction center protein L OS=Brassica napus var. napus OX=138011 GN=psbL PE=3 SV=1 |
| Photosynthesis.photophosphorylation.photosystem II.PS-II complex.component *(PsbR) | HID58_093684 | A0ABQ7XCG7 | Photosystem II 10 kDa polypeptide, chloroplastic, Uncharacterized protein OS=Brassica napus OX=3708 GN=HID58_093684 PE=4 SV=1 |
| Photosynthesis.photorespiration.glycolate oxidase *(GOX1/2/3) | BnaA03g58240D | A0A078IYV5 | (S)-2-Hydroxy-acid oxidase OS=Brassica napus OX=3708 GN=BnaA03g58240D PE=3 SV=1 |
| Phytohormone action.abscisic acid.perception and signalling.regulatory protein *(MIW1/XIW1) | HID58_018225 | A0ABQ8D9A6 | Uncharacterized protein OS=Brassica napus OX=3708 GN=HID58_018225 PE=4 SV=1 |
| Plant organogenesis.vascular system formation.phloem differentiation.coordination.regulatory protein *(OCTOPUS) | DARMORV10_C09P74550.1 | A0A816J847 | Protein OCTOPUS-like, (rape) hypothetical protein OS=Brassica napus OX=3708 GN=DARMORV10_C09P74550.1 PE=4 SV=1 |
| Protein biosynthesis.organellar translation machinery.aminoacyl-tRNA formation.tyrosine-tRNA ligase | HID58_044743 | A0ABQ8BK95 | Tyrosine-tRNA ligase, Uncharacterized protein OS=Brassica napus OX=3708 GN=HID58_044743 PE=4 SV=1 |
| Protein biosynthesis.organellar translation machinery.plastidial ribosome biogenesis.large ribosomal subunit proteome.component *(bL9c) | BnaC01g24010D | A0A078G4M4 | Large ribosomal subunit protein bL9c OS=Brassica napus OX=3708 GN=BnaC01g24010D PE=3 SV=1 |
| Protein biosynthesis.organellar translation machinery.plastidial ribosome biogenesis.large ribosomal subunit proteome.component *(uL24c) | BnaA02g09540D | A0A078GUK1 | Universal ribosomal protein uL24 family, (rape) hypothetical protein OS=Brassica napus OX=3708 GN=BnaA02g09540D PE=3 SV=1 |
| Protein biosynthesis.organellar translation machinery.plastidial ribosome biogenesis.large ribosomal subunit proteome.component *(uL29c) | HID58_024504 | A0ABQ7XJ37 | Uncharacterized protein OS=Brassica napus OX=3708 GN=HID58_024504 PE=4 SV=1 |
| Protein biosynthesis.organellar translation machinery.plastidial ribosome biogenesis.small ribosomal subunit proteome.component *(cS22) | HID58_035227 | A0ABQ8C4D6 | Uncharacterized protein OS=Brassica napus OX=3708 GN=HID58_035227 PE=4 SV=1 |
| Protein biosynthesis.organellar translation machinery.plastidial ribosome biogenesis.small ribosomal subunit proteome.component *(uS9c) | BnaAnng19390D | A0A078JCZ3 | Small ribosomal subunit protein uS9c OS=Brassica napus OX=3708 GN=BnaAnng19390D PE=3 SV=1 |
| Protein biosynthesis.ribosome biogenesis.large ribosomal subunit (LSU).LSU proteome.component *(eL21) | HID58_031080 | A0ABQ8CHS6 | Uncharacterized protein (Fragment) OS=Brassica napus OX=3708 GN=HID58_031080 PE=4 SV=1 |
| Protein biosynthesis.ribosome biogenesis.large ribosomal subunit (LSU).LSU proteome.component *(eL6) | BnaAnng22110D | A0A078JKN0 | Large subunit ribosomal protein L6e, (rape) hypothetical protein OS=Brassica napus OX=3708 GN=BnaAnng22110D PE=3 SV=1 |
| Protein biosynthesis.ribosome biogenesis.large ribosomal subunit (LSU).LSU proteome.component *(uL11) | BnaA02g10390D | A0A078GP99 | Universal ribosomal protein uL11 family, BnaA02g10390D protein OS=Brassica napus OX=3708 GN=BnaA02g10390D PE=3 SV=1 |
| Protein biosynthesis.ribosome biogenesis.rRNA maturation.rRNA methylation.C/D small nucleolar ribonucleoprotein (snoRNP) rRNA methylation complex.methyltransferase component *(Nop1/fibrillarin) | BnaA03g47570D | A0A078IJL7 | rRNA 2'-O-methyltransferase fibrillarin 2, BnaA03g47570D protein OS=Brassica napus OX=3708 GN=BnaA03g47570D PE=3 SV=1 |
| Protein biosynthesis.ribosome biogenesis.small ribosomal subunit (SSU).SSU proteome.component *(uS11) | HID58_063002 | A0ABQ8A2Z8 | Uncharacterized protein OS=Brassica napus OX=3708 GN=HID58_063002 PE=4 SV=1 |
| Protein biosynthesis.translation elongation.eEF1 aminoacyl-tRNA binding factor activity.eEF1B eEF1A-GDP-recycling complex.component *(eEF1B-gamma) | HID58_064414 | A0ABQ7Z9W3 | Elongation factor 1-gamma 2, Uncharacterized protein (Fragment) OS=Brassica napus OX=3708 GN=HID58_064414 PE=4 SV=1 |
| Protein biosynthesis.translation initiation.Pre-Initiation Complex (PIC) module.eIF1 PIC assembly factor activity.assembly factor *(eIF1A) | HID58_061446 | A0ABQ7ZYL1 | Eukaryotic translation initiation factor 4C, Uncharacterized protein OS=Brassica napus OX=3708 GN=HID58_061446 PE=4 SV=1 |
| Protein homeostasis.protein storage.seed storage protein activities.2S albumin seed storage protein *(SESA) | HID58_050857 | A0ABQ8A862 | Uncharacterized protein OS=Brassica napus OX=3708 GN=HID58_050857 PE=4 SV=1 |
| Protein homeostasis.proteolysis.aspartic peptidase activities.Pepsin-type protease | BnaC08g15160D | A0A078H0E3 | Phytepsin, (rape) hypothetical protein OS=Brassica napus OX=3708 GN=BnaC08g15160D PE=3 SV=1 |
| Protein homeostasis.proteolysis.cysteine peptidase activities.Papain-type protease activities.subclass ALP thiol protease | BnaA10g13210D | A0A078GYM1 | Cathepsin H OS=Brassica napus OX=3708 GN=BnaA10g13210D PE=3 SV=1 |
| Protein homeostasis.proteolysis.serine peptidase activities.chloroplast Clp-type protease complex.substrate adaptor component *(ClpF) | HID58_076599 | A0ABQ7YPL7 | Hemimethylated DNA-binding domain-containing protein, Uncharacterized protein OS=Brassica napus OX=3708 GN=HID58_076599 PE=4 SV=1 |
| Protein homeostasis.proteolysis.serine peptidase activities.SCPL-type carboxypeptidase activities.carboxypeptidase *(SCPL) | HID58_029906 | A0ABQ8CGH1 | Carboxypeptidase, Uncharacterized protein OS=Brassica napus OX=3708 GN=HID58_029906 PE=4 SV=1 |
| Protein homeostasis.ubiquitin-proteasome system.26S proteasome.19S regulatory particle.base subcomplex.regulatory component *(RPN1) | HID58_000823 | A0ABQ8EHX7 | 26S proteasome non-ATPase regulatory subunit 2 homolog, Uncharacterized protein OS=Brassica napus OX=3708 GN=HID58_000823 PE=4 SV=1 |
| Protein homeostasis.ubiquitin-proteasome system.ubiquitin-fold protein conjugation.CULLIN-based E3 ubiquitin ligase activities.CUL1-based/SCF E3 ubiquitin ligase complexes.F-BOX regulatory substrate adaptor activities.substrate adaptor *(SKIP24) | DARMORV10_A06P05650.1 | A0A816S7X4 | Myosin, (rape) hypothetical protein OS=Brassica napus OX=3708 GN=DARMORV10_A06P05650.1 PE=3 SV=1 |
| Protein homeostasis.ubiquitin-proteasome system.ubiquitin-fold protein conjugation.CULLIN-based E3 ubiquitin ligase activities.CUL1-based/SCF E3 ubiquitin ligase complexes.F-BOX regulatory substrate adaptor activities.substrate adaptor *(SKIP5) | HID58_082204 | A0ABQ7Y9V6 | Uncharacterized protein OS=Brassica napus OX=3708 GN=HID58_082204 PE=4 SV=1 |
| Protein homeostasis.ubiquitin-proteasome system.ubiquitin-fold protein conjugation.RUB conjugation (neddylation).E1 RUB ubiquitin-activating enzyme heterodimer.small subunit *(AXR1/AXL) | HID58_036927 | A0ABQ8C958 | Uncharacterized protein OS=Brassica napus OX=3708 GN=HID58_036927 PE=4 SV=1 |
| Protein modification.acetylation.NatB N-terminal acetylase complex.catalytic component *(NAA20) | HID58_063886 | A0ABQ7Z8E8 | Uncharacterized protein OS=Brassica napus OX=3708 GN=HID58_063886 PE=4 SV=1 |
| Protein modification.cysteine disulfide formation.endoplasmic reticulum.PDI-L subfamily protein disulfide isomerase activities.protein disulfide isomerase *(PDI-L1) | BnaA06g15500D | A0A078F495 | Protein disulfide-isomerase OS=Brassica napus OX=3708 GN=BnaA06g15500D PE=3 SV=1 |
| Protein modification.dephosphorylation.protein serine/threonine protein phosphatase activities.PPM Mn/Mg-dependent protein phosphatase activities.clade F protein phosphatase | DARMORV10_C01P07180.1 | A0A816R2X7 | Protein-serine/threonine phosphatase OS=Brassica napus OX=3708 GN=DARMORV10_C01P07180.1 PE=3 SV=1 |
| Protein modification.glycosylation.N-linked glycosylation.dolichol-phosphate biosynthesis.cis-prenyltransferase *(LEW1) | HID58_030984 | A0ABQ8CJ07 | Uncharacterized protein OS=Brassica napus OX=3708 GN=HID58_030984 PE=4 SV=1 |
| Protein modification.peptide cyclisation.Plant BURP-domain peptide cyclase *(RD22) | BnaA06g39340D | A0A078JCL4 | Dehydration-responsive protein RD22-like, (rape) hypothetical protein OS=Brassica napus OX=3708 GN=BnaA06g39340D PE=4 SV=1 |
| Protein modification.phosphorylation.CMGC protein kinase activities.CDK protein kinase activities.CDK9 protein kinase | HID58_031080 | A0ABQ8CHS6 | Uncharacterized protein (Fragment) OS=Brassica napus OX=3708 GN=HID58_031080 PE=4 SV=1 |
| Protein physical control.endoplasmic reticulum protein translocation and insertion | DARMORV10_C02P37650.1 | A0A816K9F9 | Calreticulin, (rape) hypothetical protein OS=Brassica napus OX=3708 GN=DARMORV10_C02P37650.1 PE=3 SV=1 |
| Protein physical control.endoplasmic reticulum protein translocation and insertion.Sec co-translocation system.Signal Recognition Particle (SRP) receptor complex.component *(SR-alpha) | HID58_040683 | A0ABQ8BA75 | SRP54-type proteins GTP-binding domain-containing protein, Uncharacterized protein OS=Brassica napus OX=3708 GN=HID58_040683 PE=4 SV=1 |
| Protein physical control.mitochondrial protein translocation and insertion.inner mitochondrion membrane TIM translocation system.channel-forming component *(Tim17) | DARMORV10_C03P05810.1 | A0A816HVR2 | Malic enzyme OS=Brassica napus OX=3708 GN=DARMORV10_C03P05810.1 PE=3 SV=1 |
| Protein physical control.nucleocytoplasmic transport.cargo adaptor activities.cargo adaptor protein *(IMP-alpha) | DARMORV10_C08P12580.1 | A0A816UDU9 | Importin subunit alpha OS=Brassica napus OX=3708 GN=DARMORV10_C08P12580.1 PE=3 SV=1 |
| Protein physical control.nucleocytoplasmic transport.RAN GTPase cycle.Ran-activation accessory protein *(RanBP1) | DARMORV10_A09P66600.1 | A0A816PTZ9 | 6-phosphofructo-2-kinase/fructose-2, 6-bisphosphatase, (rape) hypothetical protein OS=Brassica napus OX=3708 GN=DARMORV10_A09P66600.1 PE=4 SV=1 |
| Protein physical control.nucleocytoplasmic transport.RAN GTPase cycle.Ran-activation accessory protein *(RanBP1) | HID58_037655 | A0ABQ8BNC0 | RanBD1 domain-containing protein, Uncharacterized protein OS=Brassica napus OX=3708 GN=HID58_037655 PE=4 SV=1 |
| Protein physical control.protein chaperone activities.cytosolic Hsp70 chaperone system.Hsp40 ATPase-stimulating co-chaperone activities.DnaJ type-I molecular co-chaperone activities.molecular co-chaperone *(DjA1/2) | DARMORV10_A03P10570.1 | A0A816VBH8 | Chaperone protein dnaJ 3-like, (rape) hypothetical protein OS=Brassica napus OX=3708 GN=DARMORV10_A03P10570.1 PE=3 SV=1 |
| Protein physical control.protein chaperone activities.cytosolic Hsp70 chaperone system.Hsp70 chaperone activities.molecular chaperone *(Hsp110) | DARMORV10_A04P04090.1 | A0A817AFS0 | Heat shock 70 kDa protein 15 (rape) hypothetical protein OS=Brassica napus OX=3708 GN=DARMORV10_A04P04090.1 PE=3 SV=1 |
| Protein physical control.protein chaperone activities.ER Quality Control (ERQC) machinery.calnexin/calreticulin chaperone system.CNX-CRT cycle.ER luminal lectin chaperone *(CRT) | DARMORV10_C05P07200.1 | A0A816KF65 | Small monomeric GTPase OS=Brassica napus OX=3708 GN=DARMORV10_C05P07200.1 PE=3 SV=1 |
| Protein physical control.protein chaperone activities.Hsp90 chaperone system.molecular co-chaperone *(HIP1/2) | DARMORV10_C05P48420.1 | A0A816LK77 | TPR repeat-containing thioredoxin TDX OS=Brassica napus OX=3708 GN=DARMORV10_C05P48420.1 PE=3 SV=1 |
| Protein physical control.protein chaperone activities.Hsp90 chaperone system.molecular co-chaperone *(HIP1/2) | HID58_029640 | A0ABQ8CDP1 | STI1 domain-containing protein, Uncharacterized protein OS=Brassica napus OX=3708 GN=HID58_029640 PE=4 SV=1 |
| Protein physical control.protein chaperone activities.Hsp90 chaperone system.molecular co-chaperone *(P23) | DARMORV10_C09P01990.1 | A0A816IJ81 | Co-chaperone protein p23-1 OS=Brassica napus OX=3708 GN=DARMORV10_C09P01990.1 PE=3 SV=1 |
| Protein physical control.protein chaperone activities.mitochondrial chaperonin system.molecular chaperone *(mtHsp60) | DARMORV10_C03P53460.1 | A0A816IAK2 | Chaperonin CPN60, mitochondrial, (rape) hypothetical protein OS=Brassica napus OX=3708 GN=DARMORV10_C03P53460.1 PE=3 SV=1 |
| Protein physical control.protein chaperone activities.plastidial chaperonin system.Cpn60 chaperone complex.component-A *(CPN60A) | BnaA04g16190D | A0A078GZH9 | Chaperonin (HSP60) (rape) hypothetical protein OS=Brassica napus OX=3708 GN=BnaA04g16190D PE=3 SV=1 |
| Protein physical control.protein chaperone activities.plastidial Hsp70 chaperone system.molecular chaperone *(cpHsc70) | BnaC08g11440D | A0A078F7A7 | Heat shock protein 70 family, chloroplast, BnaC08g11440D protein OS=Brassica napus OX=3708 GN=BnaC08g11440D PE=3 SV=1 |
| Protein physical control.protein folding.Cyclophilin-type chaperone activities.peptidyl-prolyl cis-trans isomerase *(ROC7) | DARMORV10_A10P15550.1 | A0A817BD13 | Peptidyl-prolyl cis-trans isomerase (Fragment) OS=Brassica napus OX=3708 GN=DARMORV10_A10P15550.1 PE=3 SV=1 |
| Protein physical control.protein folding.FKBP-type chaperone activities.peptidyl-prolyl cis-trans isomerase *(FKBP15-1/2) | HID58_054381 | A0ABQ8AI34 | Peptidylprolyl isomerase, Uncharacterized protein OS=Brassica napus OX=3708 GN=HID58_054381 PE=4 SV=1 |
| Redox homeostasis.ascorbate-based redox regulation.dehydroascorbate reductase *(DHAR) | HID58_004476 | A0ABQ8E8Q4 | Uncharacterized protein OS=Brassica napus OX=3708 GN=HID58_004476 PE=4 SV=1 |
| Redox homeostasis.glutathione-based redox regulation.glutathione S-transferase activities.class phi glutathione S-transferase *(GSTF1/2/++) | HID58_037284 | A0ABQ8BKU7 | Uncharacterized protein OS=Brassica napus OX=3708 GN=HID58_037284 PE=4 SV=1 |
| Redox homeostasis.reactive oxygen scavenging | DARMORV10_C03P34140.1 | A0A816I7N5 | Peroxidase OS=Brassica napus OX=3708 GN=DARMORV10_C03P34140.1 PE=3 SV=1 |
| Redox homeostasis.reactive oxygen scavenging.catalase *(CAT1/2/3) | DARMORV10_C07P60920.1 | A0A816NI65 | Catalase (Fragment) OS=Brassica napus OX=3708 GN=DARMORV10_C07P60920.1 PE=3 SV=1 |
| Redox homeostasis.thiol-based redox regulation.methionine sulfoxide reductase activities.methionine S-enantiomer sulfoxide reductase *(MsrA) | BnaA03g47160D | A0A078FDK2 | Peptide-methionine (S)-S-oxide reductase OS=Brassica napus OX=3708 GN=BnaA03g47160D PE=3 SV=1 |
| RNA biosynthesis.DNA-binding transcriptional regulation.basic DNA-binding domain.basic helix-loop-helix (bHLH) domain.bHLH class-IVc transcription factor | HID58_066592 | A0ABQ7ZG33 | Uncharacterized protein OS=Brassica napus OX=3708 GN=HID58_066592 PE=4 SV=1 |
| RNA biosynthesis.DNA-binding transcriptional regulation.basic DNA-binding domain.basic leucine zipper (bZIP) domain.bZIP class-D transcription factor activity.TGA-type transcription factor | HID58_024504 | A0ABQ7XJ37 | Uncharacterized protein OS=Brassica napus OX=3708 GN=HID58_024504 PE=4 SV=1 |
| RNA biosynthesis.DNA-binding transcriptional regulation.beta-barrel DNA-binding domain.REM family.subgroup-C transcription factor | DARMORV10_A09P56140.1 | A0A816PJ92 | Transcriptional factor B3 family protein 1, (rape) hypothetical protein OS=Brassica napus OX=3708 GN=DARMORV10_A09P56140.1 PE=4 SV=1 |
| RNA biosynthesis.DNA-binding transcriptional regulation.helix-turn-helix DNA-binding domain.HSF transcription factor | DARMORV10_C04P46660.1 | A0A816JSF1 | Ferredoxin-thioredoxin reductase catalytic chain, chloroplastic OS=Brassica napus OX=3708 GN=DARMORV10_C04P46660.1 PE=3 SV=1 |
| RNA biosynthesis.DNA-binding transcriptional regulation.zinc-coordinating DNA-binding domain.STY/SRS transcription factor | HID58_082204 | A0ABQ7Y9V6 | Uncharacterized protein OS=Brassica napus OX=3708 GN=HID58_082204 PE=4 SV=1 |
| RNA biosynthesis.RNA polymerase II-dependent transcription.transcription co-activation.MEDIATOR complex.tail module.component *(MED2/MED29/MED32) | HID58_030984 | A0ABQ8CJ07 | Uncharacterized protein OS=Brassica napus OX=3708 GN=HID58_030984 PE=4 SV=1 |
| RNA biosynthesis.RNA polymerase II-dependent transcription.transcription initiation.TFIIh basal transcription factor complex.CAK kinase module.scaffold component *(TFB3) | HID58_029906 | A0ABQ8CGH1 | Carboxypeptidase, Uncharacterized protein OS=Brassica napus OX=3708 GN=HID58_029906 PE=4 SV=1 |
| RNA biosynthesis.RNA polymerase III-dependent transcription.TFIIIc transcription factor complex.multifunctional component *(TFC2/YY1) | HID58_051625 | A0ABQ8A9J9 | RRM domain-containing protein, Uncharacterized protein OS=Brassica napus OX=3708 GN=HID58_051625 PE=4 SV=1 |
| RNA homeostasis.plastidial mRNA homeostasis.ribonucleoprotein *(CP29) | HID58_035317 | A0ABQ8C4M1 | Uncharacterized protein (Fragment) OS=Brassica napus OX=3708 GN=HID58_035317 PE=4 SV=1 |
| RNA homeostasis.RNA transport.cell-to-cell transport.mRNA transport protein *(ROC) | HID58_085166 | A0ABQ7XPQ2 | Uncharacterized protein OS=Brassica napus OX=3708 GN=HID58_085166 PE=4 SV=1 |
| RNA homeostasis.RNA transport.cell-to-cell transport.mRNA transport protein *(ROC) | HID58_000332 | A0ABQ8EGF8 | Peptidyl-prolyl cis-trans isomerase, Uncharacterized protein OS=Brassica napus OX=3708 GN=HID58_000332 PE=4 SV=1 |
| RNA homeostasis.RNA transport.RNA nuclear import.cargo adapter *(IMPA4) | DARMORV10_C08P12580.1 | A0A816UDU9 | Importin subunit alpha OS=Brassica napus OX=3708 GN=DARMORV10_C08P12580.1 PE=3 SV=1 |
| RNA processing.mRNA modification.Cleavage and Polyadenylation Specificity Factor (CPSF) complex.E3 ubiquitin ligase component *(PQT3/MPE1) | BnaC09g20200D | A0A078GLC7 | E3 ubiquitin-protein ligase RBBP6, (rape) hypothetical protein OS=Brassica napus OX=3708 GN=BnaC09g20200D PE=4 SV=1 |
| RNA processing.mRNA modification.poly(A) RNA polymerase activity.regulatory protein *(PABN1/2/3) | HID58_051625 | A0ABQ8A9J9 | RRM domain-containing protein, Uncharacterized protein OS=Brassica napus OX=3708 GN=HID58_051625 PE=4 SV=1 |
| RNA processing.organelle RNA modification.C-to-U RNA editing.ORRM-type RNA editing factor activities.RNA editing factor *(ORRM3) | DARMORV10_C07P44770.1 | A0A816N162 | Exocyst subunit Exo70 family protein OS=Brassica napus OX=3708 GN=DARMORV10_C07P44770.1 PE=3 SV=1 |
| RNA processing.spliceosome-mediated pre-mRNA splicing.Exon Junction Complex (EJC).core components.component *(Y14) | DARMORV10_C06P13130.1 | A0A816Q4L2 | RNA-binding protein 8A (Fragment) OS=Brassica napus OX=3708 GN=DARMORV10_C06P13130.1 PE=3 SV=1 |
| RNA processing.tRNA modification.tRNA thiolation.mercaptopyruvate sulfurtransferase *(MST/RDH) | HID58_095721 | A0ABQ7X2K8 | Uncharacterized protein (Fragment) OS=Brassica napus OX=3708 GN=HID58_095721 PE=4 SV=1 |
| Secondary metabolism.phenolics biosynthesis.flavonoid biosynthesis.flavones | DARMORV10_A09P34160.1 | A0A816P5U2 | Dihydroflavonol reductase 1, (rape) hypothetical protein (Fragment) OS=Brassica napus OX=3708 GN=DARMORV10_A09P34160.1 PE=3 SV=1 |
| Solutetransport.carrier-mediatedtransport.CPA-typetransportersuperfamily.CPAgroup-2transporteractivities.potassiumcation:protonantiporter*(KEA) | DARMORV10_C05P00270.1 | A0A816KNP2 | K(+) efflux antiporter 1, chloroplastic-like, (rape) hypothetical protein OS=Brassica napus OX=3708 GN=DARMORV10_C05P00270.1 PE=3 SV=1 |
| Solutetransport.carrier-mediatedtransport.OPT-typetransporteractivities.ironchelatortransporter*(YSL1/2/++) | HID58_087622 | A0ABQ7XWC2 | Uncharacterized protein OS=Brassica napus OX=3708 GN=HID58_087622 PE=4 SV=1 |
| Solutetransport.primaryactivetransport.P-typeATPasesuperfamily.group-P3ATPaseactivities.P3A-typeproton-translocatingATPase*(AHA) | DARMORV10_C08P17790.1 | A0A816UDQ8 | Plasma membrane ATPase OS=Brassica napus OX=3708 GN=DARMORV10_C08P17790.1 PE=3 SV=1 |
| Uncharacterisedcontext.enzymaticactivities.EC_1oxidoreductases.EC_1-1oxidoreductaseactingonCH-OHgroupofdonor | HID58_077940 | A0ABQ7YRS2 | Uncharacterized protein OS=Brassica napus OX=3708 GN=HID58_077940 PE=4 SV=1 |
| Uncharacterisedcontext.putativeactivities.epimeraseactivities.putativeNAD-dependentepimerase/dehydratase | DARMORV10_C04P66100.1 | A0A816JWT6 | Uncharacterized protein At2g37660, chloroplastic, (rape) hypothetical protein OS=Brassica napus OX=3708 GN=DARMORV10_C04P66100.1 PE=3 SV=1 |
| Uncharacterisedcontext.unknownactivities.membraneproteinofunknownfunction | BnaA07g04430D | A0A078H697 | Uncharacterized LOC106345859, BnaA07g04430D protein OS=Brassica napus OX=3708 GN=BnaA07g04430D PE=4 SV=1 |
| Uncharacterisedcontext.unknownactivities.proteinofunknownfunction | BnaC01g35980D | A0A078G5W3 | Trichohyalin-like, (rape) hypothetical protein OS=Brassica napus OX=3708 GN=BnaC01g35980D PE=4 SV=1 |
| Uncharacterisedcontext.unknownactivities.proteinofunknownfunction | DARMORV10_C05P40720.1 | A0A816LDM1 | DUF7081 domain-containing protein, (rape) hypothetical protein OS=Brassica napus OX=3708 GN=DARMORV10_C05P40720.1 PE=4 SV=1 |
| Uncharacterisedcontext.unknownactivities.proteinofunknownfunction | DARMORV10_A06P07410.1 | A0A816S1D1 | Uncharacterized protein At4g28440, (rape) hypothetical protein OS=Brassica napus OX=3708 GN=DARMORV10_A06P07410.1 PE=4 SV=1 |
| Uncharacterisedcontext.unknownactivities.proteinofunknownfunction | HID58_063926 | A0ABQ7Z8Y3 | Single-stranded DNA binding protein Ssb-like OB fold domain-containing protein, Uncharacterized protein (Fragment) OS=Brassica napus OX=3708 GN=HID58_063926 PE=4 SV=1 |
| Uncharacterisedcontext.unknownactivities.proteinofunknownfunction | HID58_014990 | A0ABQ8DIX1 | Uncharacterized protein OS=Brassica napus OX=3708 GN=HID58_014990 PE=4 SV=1 |
| Uncharacterisedcontext.unknownactivities.proteinofunknownfunction | HID58_013121 | A0ABQ8E309 | Uncharacterized protein OS=Brassica napus OX=3708 GN=HID58_013121 PE=4 SV=1 |
| Vesicletrafficking.anterogradetrafficking.CoatproteinII(COPII)coatomermachinery.coatproteinrecruiting.smallGTPase*(Sar1) | DARMORV10_C05P07200.1 | A0A816KF65 | Small monomeric GTPase OS=Brassica napus OX=3708 GN=DARMORV10_C05P07200.1 PE=3 SV=1 |
| Vesicletrafficking.anterogradetrafficking.proteincargoreceptoractivities.P24GPI-anchorproteincargoreceptorcomplex.component*(p24-beta | BnaA01g24430D | A0A078H8W8 | Transmembrane emp24 domain-containing protein p24 beta3, (rape) hypothetical protein OS=Brassica napus OX=3708 GN=BnaA01g24430D PE=3 SV=1 |
| Vesicletrafficking.exocytictrafficking.Exocystcomplex.component*(EXO70) | DARMORV10_C07P44770.1 | A0A816N162 | Exocyst subunit Exo70 family protein OS=Brassica napus OX=3708 GN=DARMORV10_C07P44770.1 PE=3 SV=1 |
| Vesicletrafficking.retrogradetrafficking.CoatproteinI(COPI)coatomermachinery.coatproteincomplex.subunitepsilon | HID58_062910 | A0ABQ8A2S7 | Uncharacterized protein OS=Brassica napus OX=3708 GN=HID58_062910 PE=4 SV=1 |
| Vesicletrafficking.retrogradetrafficking.vesicletethering.COGtetheringcomplex.component*(COG2) | HID58_041698 | A0ABQ8BBJ8 | Uncharacterized protein OS=Brassica napus OX=3708 GN=HID58_041698 PE=4 SV=1 |
| Vesicletrafficking.vesicletethering.GARP/EARPcomplexes.component*(VPS52/POK) | HID58_073017 | A0ABQ7Z624 | Vacuolar protein sorting-associated protein 52 A, Uncharacterized protein OS=Brassica napus OX=3708 GN=HID58_073017 PE=4 SV=1 |
| Vesicletrafficking.vesicletethering.RAB-GTPaseactivities.A-classRABGTPase | DARMORV10_C09P67060.1 | A0A816JD42 | Ras-related protein RABA4a, (rape) hypothetical protein OS=Brassica napus OX=3708 GN=DARMORV10_C09P67060.1 PE=3 SV=1 |
| Vesicletrafficking.vesicletethering.RAB-GTPaseactivities.A-classRABGTPase | HID58_032276 | A0ABQ8BVW9 | Ras-related protein Rab11D, Uncharacterized protein (Fragment) OS=Brassica napus OX=3708 GN=HID58_032276 PE=4 SV=1 |

**Supplementary Table S3** – Mercator4 functional BIN classification of down-represented proteins, listing functional category, gene ID, accession and protein description.

| **NAME** | **Gene Name** | **Accession** | **Description** |
| --- | --- | --- | --- |
| Amino acid metabolism.amino acid degradation | BnaC06g01480D | A0A078G8E2 | 3-hydroxyisobutyratedehydrogenase-like1,mitochondrial,BnaC06g01480DproteinOS=BrassicanapusOX=3708GN=BnaC06g01480DPE=4SV=1 |
| Amino acid metabolism.amino acid degradation.arginine degradation.cyanase *(CYN) | CYN | A0A816MES8 | CyanatehydrataseOS=BrassicanapusOX=3708GN=CYNPE=3SV=1 |
| Amino acid metabolism.amino acid degradation.aromatic amino acids.tyrosine degradation.fumarylacetoacetate hydrolase *(FAH) | HID58_021452 | A0ABQ8CWH4 | Fumarylacetoacetase protein OS=Brassica napus OX=3708 GN=HID58_021452 PE=4 SV=1 |
| Amino acid metabolism.amino acid degradation.aromatic amino acids.tyrosine degradation.tyrosine aminotransferase *(TAT1/2/++) | HID58_041568 | A0ABQ8BCT4 | AminotransferaseclassI/classIIlargedomain-containingproteinOS=BrassicanapusOX=3708GN=HID58_041568PE=4SV=1 |
| Amino acid metabolism.amino acid degradation.gamma-aminobutyrate (GABA).gamma-aminobutyric acid pyruvate transaminase *(GABAT1) | DARMORV10_C01P42030.1 | A0A816RXC9 | Gamma-aminobutyricacidtransaminase,(rape)hypotheticalproteinOS=BrassicanapusOX=3708GN=DARMORV10_C01P42030.1PE=3SV=1 |
| Amino acid metabolism.amino acid degradation.lysine degradation.bifunctional lysine ketoglutarate reductase and saccharopine dehydrogenase | DARMORV10_C01P05470.1 | A0A816R4J7 | Alpha-aminoadipicsemialdehydesynthase,(rape)hypotheticalproteinOS=BrassicanapusOX=3708GN=DARMORV10_C01P05470.1PE=3SV=1 |
| Amino acid metabolism.aspartate group amino acid biosynthesis.asparagine biosynthesis.glutamine-dependent asparagine synthetase *(ASN1) | DARMORV10_A06P27160.1 | A0A816SRJ3 | Asparaginesynthetase[glutamine-hydrolyzing]OS=BrassicanapusOX=3708GN=DARMORV10_A06P27160.1PE=4SV=1 |
| Amino acid metabolism.aspartate group amino acid biosynthesis.aspartate-derived amino acids.bifunctional aspartate kinase and homoserine dehydrogenase *(AK-HSDH1/2) | BnaCnng21170D | A0A078IKQ3 | Bifunctionalaspartokinase/homoserinedehydrogenase1,chloroplastic,(rape)hypotheticalproteinOS=BrassicanapusOX=3708GN=BnaCnng21170DPE=3SV=1 |
| Amino acid metabolism.aspartate group amino acid biosynthesis.aspartate-derived amino acids.isoleucine biosynthesis.acetolactate synthase complex.regulatory subunit *(AIP1/3) | BnaC02g06500D | A0A078HMT6 | AcetolactatesynthaseI/IIIsmallsubunit,(rape)hypotheticalproteinOS=BrassicanapusOX=3708GN=BnaC02g06500DPE=3SV=1 |
| Amino acid metabolism.aspartate group amino acid biosynthesis.aspartate-derived amino acids.lysine biosynthesis.diaminopimelate epimerase *(DapF) | DARMORV10_A09P46600.1 | A0A816PDC5 | DiaminopimelateepimeraseOS=BrassicanapusOX=3708GN=DARMORV10_A09P46600.1PE=3SV=1 |
| Amino acid metabolism.aspartate group amino acid biosynthesis.aspartate-derived amino acids.lysine biosynthesis.hydroxy-tetrahydrodihydrodipicolinate synthase *(DHDPS) | HID58_058470 | A0ABQ7ZQ40 | 4-Hydroxy-tetrahydrodipicolinate synthase protein (Fragment) OS=Brassica napus OX=3708 GN=HID58_058470 PE=4 SV=1 |
| Amino acid metabolism.aspartate group amino acid biosynthesis.aspartate-derived amino acids.lysine biosynthesis.LL-diaminopimelate aminotransferase *(DapAT) | DARMORV10_A01P04640.1 | A0A068FAY3 | LL-diaminopimelateaminotransferase,(rape)hypotheticalproteinOS=BrassicanapusOX=3708GN=DARMORV10_A01P04640.1PE=3SV=1 |
| Amino acid metabolism.aspartate group amino acid biosynthesis.aspartate-derived amino acids.methionine biosynthesis.salvage pathway.methylthioadenosine nucleosidase *(MTN) | HID58_056278 | A0ABQ8AMS0 | Nucleosidephosphorylasedomain-containingproteinOS=BrassicanapusOX=3708GN=HID58_056278PE=4SV=1 |
| Amino acid metabolism.aspartate group amino acid biosynthesis.aspartate-derived amino acids.methionine biosynthesis.S-methylmethionine cycle.homocysteine S-methyltransferase *(HMT1/2/3) | DARMORV10_C08P37950.1 | A0A816UMB6 | Non-specificserine/threonineproteinkinaseOS=BrassicanapusOX=3708GN=DARMORV10_C08P37950.1PE=4SV=1 |
| Amino acid metabolism.aspartate group amino acid biosynthesis.aspartate-derived amino acids.threonine biosynthesis.threonine synthase *(TS1/2) | HID58_000716 | A0ABQ8EHI4 | ThreoninesynthaseproteinOS=BrassicanapusOX=3708GN=HID58_000716PE=4SV=1 |
| Amino acid metabolism.glutamate group amino acid biosynthesis.glutamate-derived amino acids.ornithine biosynthesis.N-acetylglutamate kinase complex.N-acetylglutamate kinase *(NAGK) | DARMORV10_C08P33110.1 | A0A816ULC5 | AcetylglutamatekinaseOS=BrassicanapusOX=3708GN=DARMORV10_C08P33110.1PE=3SV=1 |
| Amino acid metabolism.glutamate group amino acid biosynthesis.histidine biosynthesis.bifunctional phosphoribosyl-ATP diphosphatase and phosphoribosyl-AMP cyclohydrolase | BnaA09g24740D | A0A078FGZ3 | HistidinebiosynthesisbifunctionalproteinhisIE,chloroplastic,BnaA09g24740DproteinOS=BrassicanapusOX=3708GN=BnaA09g24740DPE=3SV=1 |
| Amino acid metabolism.glutamate group amino acid biosynthesis.histidine biosynthesis.imidazoleglycerol-phosphate synthase | DARMORV10_A01P18110.1 | A0A816XWQ7 | Imidazoleglycerol-phosphatesynthase,(rape)hypotheticalproteinOS=BrassicanapusOX=3708GN=DARMORV10_A01P18110.1PE=3SV=1 |
| Amino acid metabolism.glutamate group amino acid biosynthesis.histidine biosynthesis.ribose-phosphate diphosphokinase | DARMORV10_A05P03440.1 | A0A816SXD7 | Ribose-phosphatediphosphokinaseOS=BrassicanapusOX=3708GN=DARMORV10_A05P03440.1PE=3SV=1 |
| Amino acid metabolism.lysine biosynthesis | DARMORV10_A04P12100.1 | A0A817AY91 | Mitochondrialsaccharopinedehydrogenase-likeoxidoreductaseAt5g39410,(rape)hypotheticalproteinOS=BrassicanapusOX=3708GN=DARMORV10_A04P12100.1PE=3SV=1 |
| Amino acid metabolism.polyamine metabolism.polyamine degradation.copper-containing amine oxidase *(CuAO) | HID58_060330 | A0ABQ7ZVE1 | Multifunctional fusion protein OS=Brassica napus OX=3708 GN=HID58_060330 PE=4 SV=1 |
| Amino acid metabolism.polyamine metabolism.polyamine degradation.FAD-dependent polyamine oxidase activities.polyamine oxidase *(PAO5) | DARMORV10_C07P57070.1 | A0A816NHJ6 | Polyamineoxidase5,(rape)hypotheticalproteinOS=BrassicanapusOX=3708GN=DARMORV10_C07P57070.1PE=3SV=1 |
| Amino acid metabolism.polyamine metabolism.spermidine biosynthesis.spermidine synthase *(SPDS1/2) | BnaA08g20290D | A0A078IYK4 | Spermidine/sperminesynthasefamily,(rape)hypotheticalproteinOS=BrassicanapusOX=3708GN=BnaA08g20290DPE=3SV=1 |
| Amino acid metabolism.pyruvate group amino acid biosynthesis.pyruvate-derived amino acids.acetolactate synthase complex.regulatory subunit *(AIP1/3) | BnaC02g06500D | A0A078HMT6 | AcetolactatesynthaseI/IIIsmallsubunit,(rape)hypotheticalproteinOS=BrassicanapusOX=3708GN=BnaC02g06500DPE=3SV=1 |
| Amino acid metabolism.pyruvate group amino acid biosynthesis.pyruvate-derived amino acids.leucine biosynthesis.3-isopropylmalate dehydrogenase | HID58_028158 | A0ABQ8CV72 | 3-isopropylmalatedehydrogenase,chloroplasticOS=BrassicanapusOX=3708GN=HID58_028158PE=4SV=1 |
| Amino acid metabolism.serine group amino acid biosynthesis.cysteine biosynthesis.O-acetylserine sulfydrylase *(OASTL-a/b/c) | DARMORV10_C04P70750.1 | A0A816JK99 | CysteinesynthaseOS=BrassicanapusOX=3708GN=DARMORV10_C04P70750.1PE=3SV=1 |
| Amino acid metabolism.serine group amino acid biosynthesis.glycine biosynthesis.plastidial serine hydroxymethyltransferase *(SHMT3) | HID58_040515 | A0ABQ8B944 | SerinehydroxymethyltransferaseproteinOS=BrassicanapusOX=3708GN=HID58_040515PE=4SV=1 |
| Amino acid metabolism.serine group amino acid biosynthesis.non-photorespiratory serine.phosphoglycerate dehydrogenase | BnaA03g50990D | A0A078IH14 | D-3-phosphoglyceratedehydrogenaseOS=BrassicanapusOX=3708GN=BnaA03g50990DPE=3SV=1 |
| Amino acid metabolism.serine group amino acid biosynthesis.non-photorespiratory serine.phosphoserine aminotransferase | BnaA06g25710D | A0A078GUH2 | (rape)hypotheticalproteinOS=BrassicanapusOX=3708GN=BnaA06g25710DPE=4SV=1 |
| Amino acid metabolism.shikimate group amino acid biosynthesis.tryptophan biosynthesis.indole-3-glycerol phosphate synthase | BnaA03g57920D | A0A078JLJ1 | Indole-3-glycerol-phosphatesynthaseOS=BrassicanapusOX=3708GN=BnaA03g57920DPE=3SV=1 |
| Carbohydrate metabolism.fermentation.acetic acid biosynthesis.pyruvate decarboxylase *(PDC) | BnaAnng13920D | A0A078IZC5 | PyruvatedecarboxylaseOS=BrassicanapusOX=3708GN=BnaAnng13920DPE=3SV=1 |
| Carbohydrate metabolism.gluconeogenesis | BnaAnng15110D | A0A078J5L4 | Fructose-bisphosphataseOS=BrassicanapusOX=3708GN=BnaAnng15110DPE=3SV=1 |
| Carbohydrate metabolism.gluconeogenesis.phosphoenolpyruvate carboxykinase | BnaC02g43920D | A0A078IRF5 | Phosphoenolpyruvatecarboxykinase(ATP)OS=BrassicanapusOX=3708GN=BnaC02g43920DPE=3SV=1 |
| Carbohydrate metabolism.gluconeogenesis.pyruvate orthophosphate dikinase activity.pyruvate orthophosphate dikinase | HID58_001966 | A0ABQ8EKW9 | Pyruvate,phosphatedikinaseOS=BrassicanapusOX=3708GN=HID58_001966PE=4SV=1 |
| Carbohydrate metabolism.glycolysis | BnaA10g11380D | A0A078HII6 | Glucose-6-phosphate1-epimeraseOS=BrassicanapusOX=3708GN=BnaA10g11380DPE=3SV=1 |
| Carbohydrate metabolism.glycolysis | HID58_012572 | A0ABQ8E424 | Glucose-6-phosphate1-epimeraseproteinOS=BrassicanapusOX=3708GN=HID58_012572PE=4SV=1 |
| Carbohydrate metabolism.nucleotide sugar biosynthesis.GDP-L-fucose biosynthesis.de novo biosynthesis.GDP-D-mannose 4,6-dehydratase *(MUR1) | DARMORV10_A07P17560.1 | A0A816YTP4 | GDP-mannose4,6-dehydrataseOS=BrassicanapusOX=3708GN=DARMORV10_A07P17560.1PE=3SV=1 |
| Carbohydrate metabolism.nucleotide sugar biosynthesis.UDP-D-glucose 4-epimerase | BnaA01g13540D | A0A078ICM7 | UDP-glucose4-epimeraseOS=BrassicanapusOX=3708GN=BnaA01g13540DPE=3SV=1 |
| Carbohydrate metabolism.nucleotide sugar biosynthesis.UDP-D-glucuronic acid biosynthesis.UDP-D-glucose 6-dehydrogenase | DARMORV10_C05P24470.1 | A0A816L2D0 | UDP-glucose6-dehydrogenaseOS=BrassicanapusOX=3708GN=DARMORV10_C05P24470.1PE=3SV=1 |
| Carbohydrate metabolism.nucleotide sugar biosynthesis.UDP-L-arabinose biosynthesis.UDP-L-arabinose mutase | DARMORV10_C02P07880.1 | A0A816K1G9 | UDP-arabinopyranosemutaseOS=BrassicanapusOX=3708GN=DARMORV10_C02P07880.1PE=3SV=1 |
| Carbohydrate metabolism.oligosaccharide metabolism.alkaline alpha-galactosidase *(RFS2) | HID58_082460 | A0ABQ7YAN3 | Galactinol--sucrosegalactosyltransferaseproteinOS=BrassicanapusOX=3708GN=HID58_082460PE=4SV=1 |
| Carbohydrate metabolism.oxidative pentose phosphate pathway.non-oxidative phase.ribose 5-phosphate isomerase | BnaA01g33410D | A0A078G3Q8 | Ribose-5-phosphateisomeraseOS=BrassicanapusOX=3708GN=BnaA01g33410DPE=3SV=1 |
| Carbohydrate metabolism.oxidative pentose phosphate pathway.oxidative phase.6-phosphogluconate dehydrogenase | BnaA04g11280D | A0A078HMH2 | 6-Phosphogluconatedehydrogenase,decarboxylatingOS=BrassicanapusOX=3708GN=BnaA04g11280DPE=3SV=1 |
| Carbohydrate metabolism.oxidative pentose phosphate pathway.oxidative phase.6-phosphogluconolactonase | DARMORV10_A01P24800.1 | A0A816Y1W8 | 6-phosphogluconolactonaseOS=BrassicanapusOX=3708GN=DARMORV10_A01P24800.1PE=3SV=1 |
| Carbohydrate metabolism.oxidative pentose phosphate pathway.oxidative phase.6-phosphogluconolactonase | HID58_023369 | A0ABQ8D233 | Glucosamine/galactosamine-6-phosphate isomerase domain-containing protein OS=Brassica napus OX=3708 GN=HID58_023369 PE=4 SV=1 |
| Carbohydrate metabolism.oxidative pentose phosphate pathway.oxidative phase.glucose-6-phosphate dehydrogenase | HID58_031092 | A0ABQ8CHV8 | Glucose-6-phosphate1-dehydrogenaseprotein(Fragment)OS=BrassicanapusOX=3708GN=HID58_031092PE=4SV=1 |
| Carbohydrate metabolism.plastidial glycolysis.pyruvate kinase *(PKP1) | BnaCnng32880D | A0A078J5Z2 | Pyruvatekinase,OS=BrassicanapusOX=3708GN=BnaCnng32880DPE=3SV=1 |
| Carbohydrate metabolism.plastidial glycolysis.pyruvate kinase *(PKP1) | BnaC05g28630D | A0A078H0Q1 | PyruvatekinaseOS=BrassicanapusOX=3708GN=BnaC05g28630DPE=3SV=1 |
| Carbohydrate metabolism.starch metabolism | DARMORV10_C03P05920.1 | A0A816I295 | Alpha-D-xylosidexylohydrolaseOS=BrassicanapusOX=3708GN=DARMORV10_C03P05920.1PE=3SV=1 |
| Carbohydrate metabolism.starch metabolism.biosynthesis.ADP-glucose pyrophosphorylase complex.large subunit *(APL) | HID58_078865 | A0ABQ7YVD1 | Glucose-1-phosphateadenylyltransferaseproteinOS=BrassicanapusOX=3708GN=HID58_078865PE=4SV=1 |
| Carbohydrate metabolism.starch metabolism.biosynthesis.starch synthase activities.starch synthase *(SS2) | HID58_020302 | A0ABQ8DFC9 | StarchsynthaseproteinOS=BrassicanapusOX=3708GN=HID58_020302PE=4SV=1 |
| Carbohydrate metabolism.starch metabolism.biosynthesis.starch synthase activities.starch synthase *(SS4) | BnaC08g11320D | A0A078F736 | StarchsynthaseOS=BrassicanapusOX=3708GN=BnaC08g11320DPE=3SV=1 |
| Carbohydrate metabolism.starch metabolism.degradation.dephosphorylation.phosphoglucan phosphatase *(SEX4) | DARMORV10_A09P45250.1 | A0A816PCI0 | Dualspecificityproteinphosphatase4OS=BrassicanapusOX=3708GN=DARMORV10_A09P45250.1PE=4SV=1 |
| Carbohydrate metabolism.starch metabolism.degradation.hydrolysis and phosphorolysis.amylase activities.alpha amylase *(AMY1/2/3) | DARMORV10_A07P36260.1 | A0A816ZAB2 | Alpha-AmylaseOS=BrassicanapusOX=3708GN=DARMORV10_A07P36260.1PE=3SV=1 |
| Carbohydrate metabolism.sucrose metabolism.biosynthesis.cytosolic UDP-glucose pyrophosphorylase | HID58_020258 | A0ABQ8DF70 | UTP--glucose-1-phosphateuridylyltransferaseproteinOS=BrassicanapusOX=3708GN=HID58_020258PE=4SV=1 |
| Carbohydrate metabolism.sucrose metabolism.biosynthesis.sucrose-phosphate synthase | HID58_095211 | A0ABQ7X6Z8 | Sucrose-phosphate synthase protein OS=Brassica napus OX=3708 GN=HID58_095211 PE=4 SV=1 |
| Carbohydrate metabolism.sucrose metabolism.degradation.hexokinase | HID58_030222 | A0ABQ8CFA8 | PhosphotransferaseproteinOS=BrassicanapusOX=3708GN=HID58_030222PE=4SV=1 |
| Carbohydrate metabolism.sucrose metabolism.degradation.invertase activities.alkaline sucrose-specific invertase *(CIN) | HID58_038750 | A0ABQ8BRB7 | Alkaline/neutralinvertaseproteinOS=BrassicanapusOX=3708GN=HID58_038750PE=4SV=1 |
| Cell division.cell cycle organisation.cell cycle control.CYCLIN-dependent protein kinase complex.catalytic component *(CDKA) | DARMORV10_C08P23780.1 | A0A816UHF7 | Cyclin-dependentkinaseOS=BrassicanapusOX=3708GN=DARMORV10_C08P23780.1PE=3SV=1 |
| Cell division.cell cycle organisation.cell cycle control.mobile transcription factor *(MYB3R1) | HID58_039346 | A0ABQ8BRS4 | UncharacterizedproteinOS=BrassicanapusOX=3708GN=HID58_039346PE=4SV=1 |
| Cell division.cytokinesis.cell-plate formation.membrane remodelling activity.Phragmoplastin dynamin-like protein *(DRP1) | HID58_016547 | A0ABQ8DQM7 | Dynamin-typeGdomain-containingproteinOS=BrassicanapusOX=3708GN=HID58_016547PE=4SV=1 |
| Cell division.cytokinesis.cell-plate formation.SNARE cell-plate vesicle fusion complex.R-type SNARE component *(VAMP721/VAMP722) | HID58_079121 | A0ABQ7Y132 | Longin domain-containing protein OS=Brassica napus OX=3708 GN=HID58_079121 PE=4 SV=1 |
| Cell division.cytokinesis.endoplasmic reticulum (ER) reorganisation.ER-tubule curvature-inducing protein *(Reticulon) | HID58_019818 | A0ABQ7ZM08 | DNAgyrasesubunitBOS=BrassicanapusOX=3708GN=HID58_019818PE=4SV=1 |
| Cell division.cytokinesis.endoplasmic reticulum (ER) reorganisation.ER-tubule formation factor *(RHD3/RL) | HID58_068293 | A0ABQ7ZL02 | ProteinROOTHAIRDEFECTIVE3homologOS=BrassicanapusOX=3708GN=HID58_068293PE=4SV=1 |
| Cell division.cytokinesis.preprophase microtubule organization.regulatory protein of microtubule orientation *(CLASP) | HID58_032639 | A0ABQ8BX32 | TOGdomain-containingproteinOS=BrassicanapusOX=3708GN=HID58_032639PE=4SV=1 |
| Cell division.cytokinesis.preprophase microtubule organization.TON1-TRM-PP2A (TTP) preprophase band formation complex.PP2A protein phosphatase subcomplex.catalytic component *(PP2A-C) | BnaA05g34330D | A0A078IYJ1 | Serine/threonine-proteinphosphataseOS=BrassicanapusOX=3708GN=BnaA05g34330DPE=3SV=1 |
| Cell division.cytokinesis.preprophase microtubule organization.TON1-TRM-PP2A (TTP) preprophase band formation complex.PP2A protein phosphatase subcomplex.regulatory component *(TON2/FASS) | HID58_050878 | A0ABQ8A7D1 | EF-hand domain-containing protein OS=Brassica napus OX=3708 GN=HID58_050878 PE=4 SV=1 |
| Cell division.DNA replication.elongation.DNA polymerase delta complex.component *(POLD3) | HID58_073559 | A0ABQ7Z7L6 | Folate gamma-glutamyl hydrolase protein (Fragment) OS=Brassica napus OX=3708 GN=HID58_073559 PE=4 SV=1 |
| Cell division.DNA replication.organelle machinery.DNA gyrase complex.subunit A | DARMORV10_A03P36700.1 | A0A816W1Z3 | DNAgyrasesubunitA,chloroplastic/mitochondrialOS=BrassicanapusOX=3708GN=DARMORV10_A03P36700.1PE=3SV=1 |
| Cell division.DNA replication.organelle machinery.DNA gyrase complex.subunit B | HID58_019818 | A0ABQ7ZM08 | DNAgyrasesubunitBOS=BrassicanapusOX=3708GN=HID58_019818PE=4SV=1 |
| Cell division.plastid division.plastid separation.plastid-nucleoid partitioning factor *(YLMG1) | HID58_019114 | A0ABQ8DDN7 | DhaL domain-containing protein OS=Brassica napus OX=3708 GN=HID58_019114 PE=4 SV=1 |
| Cell division.plastid division.thylakoid organisation.regulatory protein *(THF1) | BnaA09g43540D | A0A078FLI1 | ProteinTHYLAKOIDFORMATION1,chloroplastic,BnaA09g43540DproteinOS=BrassicanapusOX=3708GN=BnaA09g43540DPE=3SV=1 |
| Cell division.plastid division.thylakoid organisation.thylakoid membrane-remodeling GTPase *(FZL) | HID58_079033 | A0ABQ7Y2K6 | G domain-containing protein (Fragment) OS=Brassica napus OX=3708 GN=HID58_079033 PE=4 SV=1 |
| Cell wall organisation.callose.degradation.beta-1,3 glucanase *(BG10/11) | DARMORV10_A07P21140.1 | A0A816YZ70 | Glucanendo-1,3-beta-D-glucosidaseOS=BrassicanapusOX=3708GN=DARMORV10_A07P21140.1PE=3SV=1 |
| Cell wall organisation.callose.degradation.beta-1,3 glucanase *(BG10/11) | HID58_038579 | A0ABQ8BPQ0 | Glucanendo-1,3-beta-D-glucosidaseproteinOS=BrassicanapusOX=3708GN=HID58_038579PE=4SV=1 |
| Cell wall organisation.cell wall proteins.expansin activities.alpha-class expansin | DARMORV10_C02P51120.1 | A0A816KDK9 | ExpansinOS=BrassicanapusOX=3708GN=DARMORV10_C02P51120.1PE=3SV=1 |
| Cell wall organisation.cell wall proteins.expansin activities.alpha-class expansin | DARMORV10_C05P24190.1 | A0A816KXY7 | ExpansinOS=BrassicanapusOX=3708GN=DARMORV10_C05P24190.1PE=3SV=1 |
| Cell wall organisation.cell wall proteins.expansin activities.alpha-class expansin | HID58_059730 | A0ABQ7ZTQ4 | ExpansinOS=BrassicanapusOX=3708GN=HID58_059730PE=4SV=1 |
| Cell wall organisation.cell wall proteins.expansin activities.alpha-class expansin | HID58_089998 | A0ABQ7Y0K9 | ExpansinproteinOS=BrassicanapusOX=3708GN=HID58_089998PE=4SV=1 |
| Cell wall organisation.cell wall proteins.hydroxyproline-rich glycoprotein activities.arabinogalactan-protein activities.arabinogalactan protein glycosylation.hydroxyproline-O-galactosyltransferase *(HPGT) | DARMORV10_A01P06180.1 | A0A816XIR4 | (rape)hypotheticalproteinOS=BrassicanapusOX=3708GN=DARMORV10_A01P06180.1PE=3SV=1 |
| Cell wall organisation.cell wall proteins.hydroxyproline-rich glycoprotein activities.arabinogalactan-protein activities.Fasciclin-type arabinogalactan protein activities.Fasciclin-type arabinogalactan protein *(FLA11) | BnaC05g02150D | A0A078FZJ3 | Fasciclin-likearabinogalactanprotein9,BnaC05g02150DproteinOS=BrassicanapusOX=3708GN=BnaC05g02150DPE=3SV=1 |
| Cell wall organisation.cell wall proteins.hydroxyproline-rich glycoprotein activities.arabinogalactan-protein activities.Fasciclin-type arabinogalactan protein activities.Fasciclin-type arabinogalactan protein *(FLA15-FLA18) | HID58_008307 | A0ABQ8DPB3 | FAS1domain-containingproteinOS=BrassicanapusOX=3708GN=HID58_008307PE=4SV=1 |
| Cell wall organisation.cell wall proteins.hydroxyproline-rich glycoprotein activities.extensin activities.extensin glycosylation.hydroxyproline beta-1,4-arabinosyltransferase *(HPAT) | DARMORV10_C09P63600.1 | A0A816JAU4 | HydroxyprolineO-arabinosyltransferase-likedomain-containingprotein,(rape)hypotheticalproteinOS=BrassicanapusOX=3708GN=DARMORV10_C09P63600.1PE=3SV=1 |
| Cell wall organisation.cellulose.cellulose-hemicellulose network assembly.regulatory protein *(COBL1/2/++) | DARMORV10_C05P07850.1 | A0A816L5G1 | COBRA C-terminal domain-containing protein OS=Brassica napus OX=3708 GN=DARMORV10_C05P07850.1 PE=3 SV=1 |
| Cell wall organisation.cutin and suberin.cuticular lipid formation.epoxidation.epoxide hydrolase *(EH) | HID58_035136 | A0ABQ8C5S8 | Thioredoxin domain-containing protein OS=Brassica napus OX=3708 GN=HID58_035136 PE=4 SV=1 |
| Cell wall organisation.cutin and suberin.cuticular lipid formation.very-long-chain fatty alcohol oxidation pathway.fatty aldehyde dehydrogenase *(FADH) | HID58_093628 | A0ABQ7XA93 | Aldehyde dehydrogenase protein OS=Brassica napus OX=3708 GN=HID58_093628 PE=4 SV=1 |
| Cell wall organisation.cutin and suberin.cutin polyester biosynthesis.cutin synthase *(CD) | BnaC05g46600D | A0A078HX36 | GDSLesterase/lipase,BnaC05g46600DproteinOS=BrassicanapusOX=3708GN=BnaC05g46600DPE=3SV=1 |
| Cell wall organisation.cutin and suberin.cutin polyester biosynthesis.cutin synthase *(CD) | DARMORV10_C03P40760.1 | A0A816I6U6 | GDSLesterase/lipaseLTL1-like,(rape)hypotheticalproteinOS=BrassicanapusOX=3708GN=DARMORV10_C03P40760.1PE=3SV=1 |
| Cell wall organisation.lignin.monolignol biosynthesis | HID58_023908 | A0ABQ8D5Y0 | Cytochrome P450 protein OS=Brassica napus OX=3708 GN=HID58_023908 PE=4 SV=1 |
| Cell wall organisation.lignin.monolignol biosynthesis | HID58_063562 | A0ABQ8A5Q5 | Cytochrome P450 protein OS=Brassica napus OX=3708 GN=HID58_063562 PE=4 SV=1 |
| Cell wall organisation.lignin.monolignol biosynthesis.caffeic acid O-methyltransferase *(COMT) | BnaA10g07270D | A0A078FPR3 | CaffeicO-methyltransferase1-5,BnaA10g07270DproteinOS=BrassicanapusOX=3708GN=BnaA10g07270DPE=4SV=1 |
| Cell wall organisation.lignin.monolignol biosynthesis.caffeoyl shikimate esterase *(CSE) | DARMORV10_A06P01640.1 | A0A816RXT9 | SerineaminopeptidaseS33domain-containingprotein,(rape)hypotheticalprotein(Fragment)OS=BrassicanapusOX=3708GN=DARMORV10_A06P01640.1PE=4SV=1 |
| Cell wall organisation.lignin.monolignol biosynthesis.ferulate 5-hydroxylase *(F5H) | HID58_000231 | A0ABQ8EFX4 | Cytochrome P450 84A1 OS=Brassica napus OX=3708 GN=HID58_000231 PE=4 SV=1 |
| Cell wall organisation.pectin.homogalacturonan.biosynthesis.methyltransferase *(CGR2/3) | BnaA06g15610D | A0A078F117 | (rape)hypotheticalproteinOS=BrassicanapusOX=3708GN=BnaA06g15610DPE=4SV=1 |
| Cell wall organisation.pectin.homogalacturonan.modification and degradation.pectin methylesterase | HID58_022552 | A0ABQ8CZP4 | PectinesteraseproteinOS=BrassicanapusOX=3708GN=HID58_022552PE=4SV=1 |
| Cell wall organisation.pectin.homogalacturonan.modification and degradation.pectin methylesterase inhibitor | HID58_067976 | A0ABQ7ZKJ6 | Pectinesterase inhibitor domain-containing protein OS=Brassica napus OX=3708 GN=HID58_067976 PE=4 SV=1 |
| Cell wall organisation.pectin.modification and degradation.pectate lyase | BnaA01g14130D | A0A078H6W1 | PectatelyaseOS=BrassicanapusOX=3708GN=BnaA01g14130DPE=3SV=1 |
| Cell wall organisation.pectin.modification and degradation.pectate lyase | DARMORV10_C08P45750.1 | A0A816UW50 | PectatelyaseOS=BrassicanapusOX=3708GN=DARMORV10_C08P45750.1PE=3SV=1 |
| Cell wall organisation.pectin.rhamnogalacturonan I.modification and degradation.alpha-L-arabinofuranosidase activities.bifunctional alpha-L-arabinofuranosidase and beta-D-xylosidase *(BXL) | DARMORV10_A06P38380.1 | A0A816SYL0 | Beta-D-xylosidase1,(rape)hypotheticalproteinOS=BrassicanapusOX=3708GN=DARMORV10_A06P38380.1PE=3SV=1 |
| Cell wall organisation.pectin.rhamnogalacturonan I.modification and degradation.alpha-L-arabinofuranosidase activities.bifunctional alpha-L-arabinofuranosidase and beta-D-xylosidase *(BXL) | HID58_091849 | A0ABQ7WYF3 | Fibronectin type III-like domain-containing protein OS=Brassica napus OX=3708 GN=HID58_091849 PE=4 SV=1 |
| Cell wall organisation.pectin.rhamnogalacturonan I.modification and degradation.alpha-L-arabinofuranosidase activities.bifunctional alpha-L-arabinofuranosidase and beta-D-xylosidase *(BXL) | HID58_073517 | A0ABQ7Z7F7 | FibronectintypeIII-likedomain-containingprotein(Fragment)OS=BrassicanapusOX=3708GN=HID58_073517PE=4SV=1 |
| Cell wall organisation.pectin.rhamnogalacturonan I.modification and degradation.beta-galactosidase activities.beta-galactosidase *(BGAL1) | BnaA02g08480D | A0A078G7U2 | Beta-galactosidaseOS=BrassicanapusOX=3708GN=BnaA02g08480DPE=3SV=1 |
| Cell wall organisation.pectin.rhamnogalacturonan I.modification and degradation.beta-galactosidase activities.beta-galactosidase *(BGAL7) | HID58_026674 | A0ABQ8CPL0 | Beta-galactosidaseproteinOS=BrassicanapusOX=3708GN=HID58_026674PE=4SV=1 |
| Cellular respiration.glycolysis.cytosolic glycolysis.glyceraldehyde 3-phosphate dehydrogenase activities.NAD-dependent glyceraldehyde 3-phosphate dehydrogenase | DARMORV10_A08P32290.1 | A0A817AKH6 | Glyceraldehyde-3-phosphatedehydrogenase(phosphorylating)OS=BrassicanapusOX=3708GN=DARMORV10_A08P32290.1PE=3SV=1 |
| Cellular respiration.glycolysis.cytosolic glycolysis.phosphoglycerate kinase *(PGK3) | DARMORV10_A07P27080.1 | A0A816Z4P8 | PhosphoglyceratekinaseOS=BrassicanapusOX=3708GN=DARMORV10_A07P27080.1PE=3SV=1 |
| Cellular respiration.glycolysis.cytosolic glycolysis.phosphoglycerate mutase | BnaA05g31980D | A0A078GRF3 | Phosphoglyceratemutase-likeproteinAT74,BnaA05g31980DproteinOS=BrassicanapusOX=3708GN=BnaA05g31980DPE=4SV=1 |
| Cellular respiration.glycolysis.cytosolic glycolysis.pyruvate kinase *(cPK) | DARMORV10_C03P15820.1 | A0A816I555 | PyruvatekinaseOS=BrassicanapusOX=3708GN=DARMORV10_C03P15820.1PE=3SV=1 |
| Cellular respiration.glycolysis.methylglyoxal/glyoxal degradation.D-lactate dehydrogenase *(D-LDH) | HID58_089688 | A0ABQ7XZR0 | D-lactatedehydrogenase(cytochrome)proteinOS=BrassicanapusOX=3708GN=HID58_089688PE=4SV=1 |
| Cellular respiration.glycolysis.methylglyoxal/glyoxal degradation.Viridiplantae-shared glyoxalase system.nickel-dependent lactoyl-glutathione lyase *(GLX-I1/2) | DARMORV10_A07P33890.1 | A0A816ZCQ1 | LactoylglutathionelyaseOS=BrassicanapusOX=3708GN=DARMORV10_A07P33890.1PE=3SV=1 |
| Cellular respiration.oxidative phosphorylation.alternative NAD(P)H dehydrogenase activities.alternative oxidase *(AOx) | HID58_043462 | A0ABQ8BGN1 | Ubiquinol oxidase protein OS=Brassica napus OX=3708 GN=HID58_043462 PE=4 SV=1 |
| Cellular respiration.oxidative phosphorylation.ATP synthase complex.membrane MF0 subcomplex.component *(6kDa/ATP6) | HID58_051146 | A0ABQ8A844 | UncharacterizedproteinOS=BrassicanapusOX=3708GN=HID58_051146PE=4SV=1 |
| Cellular respiration.oxidative phosphorylation.ATP synthase complex.peripheral MF1 subcomplex.subunit alpha | DARMORV10_C04P19640.1 | A0A816JTM6 | ATPsynthasesubunitalphaOS=BrassicanapusOX=3708GN=DARMORV10_C04P19640.1PE=3SV=1 |
| Cellular respiration.oxidative phosphorylation.cytochrome c oxidase complex.core components.component *(COX6a) | HID58_030222 | A0ABQ8CFA8 | PhosphotransferaseproteinOS=BrassicanapusOX=3708GN=HID58_030222PE=4SV=1 |
| Cellular respiration.oxidative phosphorylation.cytochrome c reductase complex.component *(QCR6) | HID58_006988 | A0ABQ8ED10 | ComplexIIIsubunitVI(Fragment)OS=BrassicanapusOX=3708GN=HID58_006988PE=4SV=1 |
| Cellular respiration.oxidative phosphorylation.cytochrome c reductase complex.core subcomplex.subunit beta *(QCR1) | BnaC05g48550D | A0A078HZF7 | MitochondrialprocessingpeptidaseOS=BrassicanapusOX=3708GN=BnaC05g48550DPE=3SV=1 |
| Cellular respiration.oxidative phosphorylation.NADH dehydrogenase complex | HID58_017066 | A0ABQ8D626 | NAD(P)H dehydrogenase (quinone) protein (Fragment) OS=Brassica napus OX=3708 GN=HID58_017066 PE=4 SV=1 |
| Cellular respiration.oxidative phosphorylation.NADH dehydrogenase complex.module N (electron input).component *(NQO1/51kDa) | DARMORV10_C03P04400.1 | A0A816I4Z1 | NADHdehydrogenase[ubiquinone]flavoprotein1,mitochondrialOS=BrassicanapusOX=3708GN=DARMORV10_C03P04400.1PE=3SV=1 |
| Cellular respiration.oxidative phosphorylation.NADH dehydrogenase complex.module P (proton translocation).component *(ND1/NQO8) | nad1 | A0A7T7BWD1 | NADH-ubiquinoneoxidoreductasechain1OS=BrassicanapusOX=3708GN=nad1PE=3SV=1 |
| Cellular respiration.oxidative phosphorylation.NADH dehydrogenase complex.module Q (electron output).component *(NQO6/PSST) | HID58_050500 | A0ABQ8A6D7 | OTU domain-containing protein OS=Brassica napus OX=3708 GN=HID58_050500 PE=4 SV=1 |
| Cellular respiration.oxidative phosphorylation.NADH dehydrogenase complex.non-core modules.alpha subcomplex.component *(NDUFA13/B16-6) | BnaA08g27970D | A0A078FTA9 | NADHdehydrogenase[ubiquinone]1alphasubcomplexsubunit13OS=BrassicanapusOX=3708GN=BnaA08g27970DPE=3SV=1 |
| Cellular respiration.oxidative phosphorylation.NADH dehydrogenase complex.non-core modules.carbonic anhydrase module.component *(CA) | HID58_018451 | A0ABQ8D9X0 | UncharacterizedproteinOS=BrassicanapusOX=3708GN=HID58_018451PE=4SV=1 |
| Cellular respiration.oxidative phosphorylation.NADH dehydrogenase complex.non-core modules.carbonic anhydrase module.component *(CAL) | HID58_018451 | A0ABQ8D9X0 | UncharacterizedproteinOS=BrassicanapusOX=3708GN=HID58_018451PE=4SV=1 |
| Cellular respiration.pyruvate oxidation.mitochondrial pyruvate dehydrogenase complex.regulation.pyruvate dehydrogenase kinase *(PDHK) | DARMORV10_A03P34710.1 | A0A816W090 | Protein-serine/threoninekinase(Fragment)OS=BrassicanapusOX=3708GN=DARMORV10_A03P34710.1PE=3SV=1 |
| Cellular respiration.tricarboxylic acid cycle.2-oxoglutarate dehydrogenase complex.E1 component *(OGDH-E1) | DARMORV10_C03P59910.1 | A0A816I9N6 | 2-Oxoglutaratedehydrogenase,mitochondrialOS=BrassicanapusOX=3708GN=DARMORV10_C03P59910.1PE=3SV=1 |
| Cellular respiration.tricarboxylic acid cycle.succinyl-CoA ligase heterodimer.subunit alpha | DARMORV10_A03P46310.1 | A0A816VY53 | Succinate--CoAligase[ADP-forming]subunitalpha,mitochondrialOS=BrassicanapusOX=3708GN=DARMORV10_A03P46310.1PE=3SV=1 |
| Chromatin organisation.chromatin structure.structure modulation.chromatin architectural modulator *(DEK) | HID58_030056 | A0ABQ8CGW5 | DEK-Cdomain-containingproteinOS=BrassicanapusOX=3708GN=HID58_030056PE=4SV=1 |
| Chromatin organisation.chromatin structure.structure modulation.chromatin architectural modulator *(HMGB1/2/3) | DARMORV10_C05P19300.1 | A0A816KW83 | ENTHdomain-containingprotein(rape)hypotheticalproteinOS=BrassicanapusOX=3708GN=DARMORV10_C05P19300.1PE=4SV=1 |
| Chromatin organisation.epigenetic state regulation.RNA-directed DNA methylation (RdDM) pathway.RNA polymerase-IV branch (siRNA generation).regulatory factor *(GDE1) | HID58_044594 | A0ABQ8BLL9 | Ribosomal RNA-processing protein 42 OS=Brassica napus OX=3708 GN=HID58_044594 PE=4 SV=1 |
| Chromatin organisation.histone chaperone activities.CAF1 histone chaperone complex.component *(CAF1c/MSI) | HID58_088187 | A0ABQ7XVN8 | Histone-binding protein RBBP4-like N-terminal domain-containing protein (Fragment) OS=Brassica napus OX=3708 GN=HID58_088187 PE=4 SV=1 |
| Chromatin organisation.nucleosome remodeling.ISWI chromatin remodeling complexes.shared ATPase component *(CHR11/CHR17) | DARMORV10_C05P60840.1 | A0A816LHQ9 | (rape)hypotheticalproteinOS=BrassicanapusOX=3708GN=DARMORV10_C05P60840.1PE=3SV=1 |
| Chromatin organisation.nucleosome remodeling.SWI/SNF chromatin remodeling complexes.shared BRM/SYD/MINU-associated module.SMARCN component *(ARP7) | BnaA09g38930D | A0A078ISB1 | Actin-relatedprotein7OS=BrassicanapusOX=3708GN=BnaA09g38930DPE=3SV=1 |
| Chromatin organisation.post-translational histone modification.histone acetylation.chaperone of histone deacetylase activity *(MSI4/5) | HID58_032611 | A0ABQ8BWW7 | Histone-bindingproteinRBBP4-likeN-terminaldomain-containingproteinOS=BrassicanapusOX=3708GN=HID58_032611PE=4SV=1 |
| Chromatin organisation.post-translational histone modification.histone acetylation.HD1-type histone deacetylase activities.class-I histone deacetylase activities.HDA19 histone deacetylase complex.deacetylase component *(HDA19) | BnaA01g33500D | A0A078G6T8 | HistonedeacetylaseOS=BrassicanapusOX=3708GN=BnaA01g33500DPE=3SV=1 |
| Chromatin organisation.post-translational histone modification.histone acetylation.HD2-type histone deacetylase *(HDT1/2/++) | BnaA04g16080D | A0A078GVN7 | Nucleoplasmin-likedomain-containingprotein,(rape)hypotheticalproteinOS=BrassicanapusOX=3708GN=BnaA04g16080DPE=4SV=1 |
| Chromatin organisation.post-translational histone modification.histone acetylation.HD2-type histone deacetylase *(HDT1/2/++) | BnaA02g05840D | A0A078FBB6 | Nucleoplasmin-likedomain-containingprotein,BnaA02g05840DproteinOS=BrassicanapusOX=3708GN=BnaA02g05840DPE=3SV=1 |
| Chromatin organisation.post-translational histone modification.histone methylation.arginine methylation.histone methylase *(PRMT4) | HID58_069219 | A0ABQ7XET9 | TypeIproteinargininemethyltransferaseOS=BrassicanapusOX=3708GN=HID58_069219PE=4SV=1 |
| Chromatin organisation.post-translational histone modification.histone methylation.lysine methylation.class-I histone methyltransferase activities.PRC2 histone methylation complex.VRN/FIS/EMF core subcomplexes.component *(MSI1) | HID58_088187 | A0ABQ7XVN8 | Histone-binding protein RBBP4-like N-terminal domain-containing protein (Fragment) OS=Brassica napus OX=3708 GN=HID58_088187 PE=4 SV=1 |
| Clade-specific metabolism.Brassicaceae.glucosinolate biosynthesis.methylthioalkylmalate dehydrogenase *(IPMDH) | HID58_028158 | A0ABQ8CV72 | 3-isopropylmalatedehydrogenase,chloroplasticOS=BrassicanapusOX=3708GN=HID58_028158PE=4SV=1 |
| Coenzyme metabolism.chlorophyll metabolism.chlorophyll biosynthesis.Mg-protoporphyrin IX monomethylester cyclase complex.catalytic component *(CRD1) | HID58_082407 | A0ABQ7YDB2 | Magnesium-protoporphyrin IX monomethyl ester (oxidative) cyclase protein OS=Brassica napus OX=3708 GN=HID58_082407 PE=4 SV=1 |
| Coenzyme metabolism.chlorophyll metabolism.chlorophyll biosynthesis.Mg-protoporphyrin IX monomethylester cyclase complex.scaffold component *(LCAA) | HID58_088187 | A0ABQ7XVN8 | Histone-binding protein RBBP4-like N-terminal domain-containing protein (Fragment) OS=Brassica napus OX=3708 GN=HID58_088187 PE=4 SV=1 |
| Coenzyme metabolism.iron-sulfur cluster assembly machinery.cytosolic CIA system.assembly phase.glutaredoxin maturation factor *(GRXS17) | HID58_052972 | A0ABQ8AE70 | Thioredoxindomain-containingproteinOS=BrassicanapusOX=3708GN=HID58_052972PE=4SV=1 |
| Coenzyme metabolism.phosphoadenosine phosphosulfate (PAPS) homeostasis.ATP sulfurylase *(APS) | HID58_076011 | A0ABQ7YL95 | SulfateadenylyltransferaseOS=BrassicanapusOX=3708GN=HID58_076011PE=4SV=1 |
| Coenzyme metabolism.S-adenosyl methionine (SAM) cycle.S-adenosyl methionine synthetase *(MAT) | DARMORV10_A04P28170.1 | A0A817AWD8 | S-adenosylmethioninesynthaseOS=BrassicanapusOX=3708GN=DARMORV10_A04P28170.1PE=3SV=1 |
| Coenzyme metabolism.tetrahydrofolate metabolism.dihydrofolate synthetase | BnaA06g36040D | A0A078H7J7 | Dihydrofolatesynthetase,(rape)hypotheticalproteinOS=BrassicanapusOX=3708GN=BnaA06g36040DPE=3SV=1 |
| Coenzyme metabolism.tetrahydrofolate metabolism.folyl-polyglutamate synthetase | HID58_004058 | A0ABQ8E4Q1 | FolylpolyglutamatesynthaseproteinOS=BrassicanapusOX=3708GN=HID58_004058PE=4SV=1 |
| Coenzyme metabolism.tetrahydrofolate metabolism.gamma-glutamyl hydrolase *(GGH) | HID58_073559 | A0ABQ7Z7L6 | Folate gamma-glutamyl hydrolase protein (Fragment) OS=Brassica napus OX=3708 GN=HID58_073559 PE=4 SV=1 |
| Coenzyme metabolism.tetrahydrofolate metabolism.pterin regeneration.pterin-4-alpha-carbinolamine dehydratase | BnaA09g26630D | A0A078GM84 | 4a-HydroxytetrahydrobiopterindehydrataseOS=BrassicanapusOX=3708GN=BnaA09g26630DPE=3SV=1 |
| Coenzyme metabolism.tetrapyrrole metabolism.heme oxygenase activities.alpha-helical heme oxygenase *(HO1) | BnaC06g17920D | A0A078GF16 | Hemeoxygenase(biliverdin-producing)OS=BrassicanapusOX=3708GN=BnaC06g17920DPE=3SV=1 |
| Coenzyme metabolism.tetrapyrrole metabolism.heme oxygenase activities.plastidial heme-binding protein *(HBP3) | DARMORV10_A05P38960.1 | A0A816TUJ8 | Heme-binding-likeproteinAt3g10130,chloroplastic,(rape)hypotheticalproteinOS=BrassicanapusOX=3708GN=DARMORV10_A05P38960.1PE=3SV=1 |
| Coenzyme metabolism.tetrapyrrole metabolism.protoporphyrin IX formation.protoporphyrinogen IX oxidase activities.plastidial protoporphyrinogen IX oxidase *(HemG1) | DARMORV10_C05P01130.1 | A0A816KUU9 | ProtoporphyrinogenoxidaseOS=BrassicanapusOX=3708GN=DARMORV10_C05P01130.1PE=3SV=1 |
| Coenzyme metabolism.tetrapyrrole metabolism.siroheme biosynthesis.sirohydrochlorin ferrochelatase *(SirB) | HID58_057383 | A0ABQ8AQY6 | Diseaseresistanceprotein(Fragment)OS=BrassicanapusOX=3708GN=HID58_057383PE=4SV=1 |
| Coenzyme metabolism.thiamine pyrophosphate metabolism.pyrimidine moiety.hydroxymethylpyrimidine phosphate synthase *(ThiC) | HID58_059657 | A0ABQ7ZTK5 | Phosphomethylpyrimidine synthase, chloroplastic protein OS=Brassica napus OX=3708 GN=HID58_059657 PE=4 SV=1 |
| Cytoskeleton organisation.actin and tubulin folding.post-CCT Tubulin folding pathway.tubulin folding cofactor | HID58_071419 | A0ABQ7Z1P0 | TBCCdomain-containingprotein1OS=BrassicanapusOX=3708GN=HID58_071419PE=4SV=1 |
| Cytoskeleton organisation.actin and tubulin folding.post-CCT Tubulin folding pathway.tubulin folding cofactor *(PLP3) | HID58_035136 | A0ABQ8C5S8 | Thioredoxin domain-containing protein OS=Brassica napus OX=3708 GN=HID58_035136 PE=4 SV=1 |
| Cytoskeleton organisation.actin and tubulin folding.TRiC/CCT chaperonin folding complex.subunit zeta *(CCT6) | HID58_088999 | A0ABQ7XXS5 | UncharacterizedproteinOS=BrassicanapusOX=3708GN=HID58_088999PE=4SV=1 |
| Cytoskeleton organisation.endoplasmic reticulum (ER) - cell/organelle membrane contact.ER - plasma membrane interface.contact site modulator *(VST) | HID58_056857 | A0ABQ8AQG1 | MSPdomain-containingproteinOS=BrassicanapusOX=3708GN=HID58_056857PE=4SV=1 |
| Cytoskeleton organisation.microfilament network.myosin microfilament-based motor protein activities.class-VIII myosin motor protein activity.myosin motor protein *(ATM1/2) | BnaA05g19230D | A0A078GIZ4 | TRAFACclassmyosin-kinesinATPasesuperfamily,BnaA05g19230DproteinOS=BrassicanapusOX=3708GN=BnaA05g19230DPE=3SV=1 |
| Cytoskeleton organisation.microfilament network.myosin microfilament-based motor protein activities.myosin receptor MyoB activities.MyoB class-IIb myosin receptor | HID58_003515 | A0ABQ8EQY2 | Ubiquitinyl hydrolase 1 protein OS=Brassica napus OX=3708 GN=HID58_003515 PE=4 SV=1 |
| Cytoskeleton organisation.microtubular network.Kinesin microtubule-based motor protein activities.Kinesin-14-type motor protein activities.kinesin motor protein *(KAC) | DARMORV10_C03P05120.1 | A0A816I220 | Kinesinmotordomain-containingprotein,(rape)hypotheticalproteinOS=BrassicanapusOX=3708GN=DARMORV10_C03P05120.1PE=3SV=1 |
| Cytoskeleton organisation.microtubular network.Kinesin microtubule-based motor protein activities.Kinesin-7-type motor protein | HID58_036211 | A0ABQ8C736 | Kinesin-like protein OS=Brassica napus OX=3708 GN=HID58_036211 PE=4 SV=1 |
| Cytoskeleton organisation.microtubular network.microtubule dynamics.microtubule plus-end control.microtubule orientation regulator *(CLASP) | HID58_032639 | A0ABQ8BX32 | TOGdomain-containingproteinOS=BrassicanapusOX=3708GN=HID58_032639PE=4SV=1 |
| Cytoskeleton organisation.microtubular network.microtubule dynamics.microtubule plus-end control.regulatory protein *(SPR1) | HID58_023971 | A0ABQ8D3M2 | Glutamine amidotransferase type-2 domain-containing protein OS=Brassica napus OX=3708 GN=HID58_023971 PE=4 SV=1 |
| Cytoskeleton organisation.microtubular network.microtubule dynamics.microtubule-stabilizing factor *(WDL1/2/++) | BnaC03g29720D | A0A078G971 | ProteinWVD2-like2,(rape)hypotheticalproteinOS=BrassicanapusOX=3708GN=BnaC03g29720DPE=3SV=1 |
| Cytoskeleton organisation.microtubular network.microtubule formation.alpha-beta-Tubulin heterodimer.beta-Tubulin component *(TUB) | BnaA05g12490D | A0A078I5S9 | TubulinbetachainOS=BrassicanapusOX=3708GN=BnaA05g12490DPE=3SV=1 |
| Cytoskeleton organisation.microtubular network.microtubule formation.alpha-beta-Tubulin heterodimer.beta-Tubulin component *(TUB) | DARMORV10_C09P64620.1 | A0A816J613 | TubulinbetachainOS=BrassicanapusOX=3708GN=DARMORV10_C09P64620.1PE=3SV=1 |
| Cytoskeleton organisation.microtubular network.microtubule formation.alpha-beta-Tubulin heterodimer.beta-Tubulin component *(TUB) |  | #N/D | #N/D |
| Cytoskeleton organisation.nuclear dynamics.nuclear basket to nucleoskeleton bridging.regulatory GTPase protein *(GBPL) | BnaA09g17790D | A0A078HBL4 | Guanylate-bindingprotein2,(rape)hypotheticalproteinOS=BrassicanapusOX=3708GN=BnaA09g17790DPE=3SV=1 |
| Cytoskeleton organisation.nuclear dynamics.nuclear shape determination.lamin-like protein *(CRWN) | HID58_054762 | A0ABQ8AIR4 | UncharacterizedproteinOS=BrassicanapusOX=3708GN=HID58_054762PE=4SV=1 |
| Cytoskeleton organisation.plastid movement.accessory motility factor *(KAC1/2) | DARMORV10_C03P05120.1 | A0A816I220 | Kinesinmotordomain-containingprotein,(rape)hypotheticalproteinOS=BrassicanapusOX=3708GN=DARMORV10_C03P05120.1PE=3SV=1 |
| Cytoskeleton organisation.plastid movement.actin filament reorganisation.stability factor *(PMI1) | DARMORV10_C08P09250.1 | A0A816TYJ5 | C2NT-typedomain-containingprotein,(rape)hypotheticalproteinOS=BrassicanapusOX=3708GN=DARMORV10_C08P09250.1PE=4SV=1 |
| DNA damage response.base excision repair (BER).gap filling and nick sealing.single-strand DNA repair protein *(XRCC1) | HID58_006230 | A0ABQ8EAX0 | BRCTdomain-containingproteinOS=BrassicanapusOX=3708GN=HID58_006230PE=4SV=1 |
| DNA damage response.homologous recombination repair (HR).BRCC regulatory holo-complex.component *(BRCC45) | BnaA06g37130D | A0A078GAH7 | BRISCandBRCA1-Acomplexmember2OS=BrassicanapusOX=3708GN=BnaA06g37130DPE=3SV=1 |
| DNA damage response.mismatch repair (MMR).lesion recognition.MSH2-x mismatch repair heterodimers.component *(MSH3) | DARMORV10_C07P54390.1 | A0A816N0S2 | DNAmismatchrepairproteinMSH3OS=BrassicanapusOX=3708GN=DARMORV10_C07P54390.1PE=3SV=1 |
| DNA damage response.nucleotide excision repair (NER).damage recognition.transcription-coupled nucleotide excision repair (TC-NER).deubiquitinase *(UBP12/13) | HID58_003515 | A0ABQ8EQY2 | Ubiquitinyl hydrolase 1 protein OS=Brassica napus OX=3708 GN=HID58_003515 PE=4 SV=1 |
| DNA damage response.nucleotide excision repair (NER).damage recognition.transcription-coupled nucleotide excision repair (TC-NER).deubiquitinase *(UBP12/13) | HID58_004075 | A0ABQ8E4T5 | Ubiquitinylhydrolase1proteinOS=BrassicanapusOX=3708GN=HID58_004075PE=4SV=1 |
| External stimuli response | HID58_043873 | A0ABQ8BHT2 | Stress-response A/B barrel domain-containing protein OS=Brassica napus OX=3708 GN=HID58_043873 PE=4 SV=1 |
| External stimuli response | BnaC09g36390D | A0A078GNY7 | Stress-responseA/Bbarreldomain-containingproteinAt5g22580,BnaC09g36390DproteinOS=BrassicanapusOX=3708GN=BnaC09g36390DPE=4SV=1 |
| External stimuli response | BnaA05g27200D | A0A078F604 | UniversalstressproteinYxiE,BnaA05g27200DproteinOS=BrassicanapusOX=3708GN=BnaA05g27200DPE=4SV=1 |
| External stimuli response.gravity.gravitropism.regulatory protein *(WEEP) | HID58_044594 | A0ABQ8BLL9 | Ribosomal RNA-processing protein 42 OS=Brassica napus OX=3708 GN=HID58_044594 PE=4 SV=1 |
| External stimuli response.light.multiple photoreceptor signalling.substrate adaptor module of CUL4-DDB1 ubiquitin ligase complex.COP1 E3 ubiquitin ligase activity.regulatory factor *(CSU2) | HID58_063833 | A0ABQ7Z888 | Cupin type-1 domain-containing protein (Fragment) OS=Brassica napus OX=3708 GN=HID58_063833 PE=4 SV=1 |
| External stimuli response.light.multiple photoreceptor signalling.substrate adaptor module of CUL4-DDB1 ubiquitin ligase complex.regulatory component *(SPA) | HID58_070590 | A0ABQ7YZ90 | Proteinkinasedomain-containingproteinOS=BrassicanapusOX=3708GN=HID58_070590PE=4SV=1 |
| External stimuli response.light.red/far red light.phytochrome signalling.regulatory protein *(LAF1) | HID58_012677 | A0ABQ8E4D6 | Uncharacterizedprotein(Fragment)OS=BrassicanapusOX=3708GN=HID58_012677PE=4SV=1 |
| External stimuli response.light.red/far red light.phytochrome signalling.transcriptional regulator *(PAT1) | DARMORV10_A06P03020.1 | A0A816SAR4 | Scarecrow-likeprotein5,(rape)hypotheticalproteinOS=BrassicanapusOX=3708GN=DARMORV10_A06P03020.1PE=3SV=1 |
| External stimuli response.light.red/far red light.phytochrome-mediated photoperception.phytochrome biosynthesis.apophytochrome *(PHY-A) | DARMORV10_C05P49390.1 | A0A816L9H0 | PhytochromeB-like,(rape)hypotheticalproteinOS=BrassicanapusOX=3708GN=DARMORV10_C05P49390.1PE=3SV=1 |
| External stimuli response.light.UV-A/blue light.cryptochrome-mediated photoperception.cryptochrome photoreceptor *(CRY) | BnaAnng13370D | A0A078IZQ8 | Bluelightphotoreceptor,OS=BrassicanapusOX=3708GN=BnaAnng13370DPE=3SV=1 |
| External stimuli response.light.UV-A/blue light.phototropin-mediated photoperception.phototropin photoreceptor *(PHOT1/2) | BnaA02g07840D | A0A078H5I3 | Non-specificserine/threonineproteinkinaseOS=BrassicanapusOX=3708GN=BnaA02g07840DPE=3SV=1 |
| External stimuli response.pathogen.defense mechanisms.defense enzyme activities.pathogen polygalacturonase inhibitor *(PGIP1/2) | HID58_019676 | A0ABQ8DDG1 | Leucine-rich repeat-containing N-terminal plant-type domain-containing protein (Fragment) OS=Brassica napus OX=3708 GN=HID58_019676 PE=4 SV=1 |
| External stimuli response.pathogen.defense mechanisms.defense enzyme activities.pathogen polygalacturonase inhibitor *(PGIP1/2) | HID58_050288 | A0ABQ8A5W0 | Leucine-rich repeat-containing N-terminal plant-type domain-containing protein OS=Brassica napus OX=3708 GN=HID58_050288 PE=4 SV=1 |
| External stimuli response.pathogen.defense mechanisms.systemic acquired resistance (SAR).pipecolic acid metabolism.pipecolate oxidase *(SOX) | BnaC04g36290D | A0A078G5U4 | Sarcosineoxidase,(rape)hypotheticalproteinOS=BrassicanapusOX=3708GN=BnaC04g36290DPE=3SV=1 |
| External stimuli response.pathogen.effector-triggered immunity (ETI) machinery.TNL-mediated effector-triggered immunity.EDS1-PAD4/SAG101 signalling heterodimers.component *(SAG101) | HID58_050672 | A0ABQ8A6U5 | UncharacterizedproteinOS=BrassicanapusOX=3708GN=HID58_050672PE=4SV=1 |
| External stimuli response.pathogen.virus infection.tobamovirus multiplication.replication host factor *(TOM1) | HID58_041350 | A0ABQ8BAK3 | THH1/TOM1/TOM3 domain-containing protein (Fragment) OS=Brassica napus OX=3708 GN=HID58_041350 PE=4 SV=1 |
| External stimuli response.pathogen.virus infection.tobamovirus multiplication.small GTPase *(ARL8) | BnaC07g31560D | A0A078GCG6 | SmallGTPasesuperfamily.Arffamily,BnaC07g31560DproteinOS=BrassicanapusOX=3708GN=BnaC07g31560DPE=3SV=1 |
| External stimuli response.temperature.cold response.cold-responsive mRNA chaperone *(CSD) | BnaC03g57650D | A0A078FI85 | Coldshockprotein2,(rape)hypotheticalproteinOS=BrassicanapusOX=3708GN=BnaC03g57650DPE=3SV=1 |
| External stimuli response.temperature.heat response.monogalactosyldiacylglycerol lipase *(HIL1) | HID58_061366 | A0ABQ7ZYD6 | C2domain-containingproteinOS=BrassicanapusOX=3708GN=HID58_061366PE=4SV=1 |
| External stimuli response.toxic compounds.reactive aldehyde detoxification.aldo-keto reductase *(AKR4C8/9) | HID58_069055 | A0ABQ7ZNP5 | 2-alkenalreductase[NAD(P)(+)](Fragment)OS=BrassicanapusOX=3708GN=HID58_069055PE=4SV=1 |
| External stimuli response.toxic compounds.reactive aldehyde detoxification.aldo-keto reductase *(AKR4C8/9) | HID58_082162 | A0ABQ7Y9R7 | NADP-dependentoxidoreductasedomain-containingproteinOS=BrassicanapusOX=3708GN=HID58_082162PE=4SV=1 |
| External stimuli response.toxic compounds.reactive aldehyde detoxification.formaldehyde.S-formylglutathione hydrolase *(SFGH) | HID58_010134 | A0ABQ8DUE0 | S-formylglutathionehydrolaseproteinOS=BrassicanapusOX=3708GN=HID58_010134PE=4SV=1 |
| External stimuli response.water and drought.osmosensing.condensable transcriptional regulator *(SEU/SLK) | DARMORV10_C07P54390.1 | A0A816N0S2 | DNAmismatchrepairproteinMSH3OS=BrassicanapusOX=3708GN=DARMORV10_C07P54390.1PE=3SV=1 |
| External stimuli response.wounding | DARMORV10_A06P20480.1 | A0A816SMV0 | Beta-glucosidase,(rape)hypotheticalproteinOS=BrassicanapusOX=3708GN=DARMORV10_A06P20480.1PE=3SV=1 |
| External stimuli response.wounding | BnaCnng39140D | A0A078J6L2 | Thioglucosidase,OS=BrassicanapusOX=3708GN=BnaCnng39140DPE=3SV=1 |
| External stimuli response.wounding | HID58_091934 | A0ABQ7WY43 | Thioglucosidase,OS=BrassicanapusOX=3708GN=HID58_091934PE=4SV=1 |
| External stimuli response.wounding | BnaA02g17600D | A0A078HNC1 | ThioglucosidaseOS=BrassicanapusOX=3708GN=BnaA02g17600DPE=3SV=1 |
| External stimuli response.wounding | BnaC01g43700D | A0A078J5J3 | ThioglucosidaseOS=BrassicanapusOX=3708GN=BnaC01g43700DPE=3SV=1 |
| Lipid metabolism.cytoplasmic lipid droplet-associated activities.Caleosin-type peroxygenase *(CLO/PXG) | DARMORV10_C04P62820.1 | A0A816JVZ8 | peroxygenase3(rape)hypotheticalproteinOS=BrassicanapusOX=3708GN=DARMORV10_C04P62820.1PE=3SV=1 |
| Lipid metabolism.cytoplasmic lipid droplet-associated activities.Caleosin-type peroxygenase *(CLO/PXG) | BnaA05g10200D | A0A078FXS4 | Peroxygenase3,BnaA05g10200DproteinOS=BrassicanapusOX=3708GN=BnaA05g10200DPE=3SV=1 |
| Lipid metabolism.fatty acid metabolism | HID58_028387 | A0ABQ8CC64 | 3-oxoacyl-[acyl-carrier-protein]reductaseproteinOS=BrassicanapusOX=3708GN=HID58_028387PE=4SV=1 |
| Lipid metabolism.fatty acid metabolism | DARMORV10_C05P19130.1 | A0A816KW56 | butanoate--CoAligaseAAE1,(rape)hypotheticalproteinOS=BrassicanapusOX=3708GN=DARMORV10_C05P19130.1PE=3SV=1 |
| Lipid metabolism.fatty acid metabolism.acetyl-CoA generation.plastidial pyruvate dehydrogenase complex.E2 dihydrolipoamide acetyltransferase component | DARMORV10_C06P11500.1 | A0A816Q7E3 | DihydrolipoamideacetyltransferasecomponentofpyruvatedehydrogenasecomplexOS=BrassicanapusOX=3708GN=DARMORV10_C06P11500.1PE=3SV=1 |
| Lipid metabolism.fatty acid metabolism.acetyl-CoA generation.plastidial pyruvate kinase | BnaC05g28630D | A0A078H0Q1 | PyruvatekinaseOS=BrassicanapusOX=3708GN=BnaC05g28630DPE=3SV=1 |
| Lipid metabolism.fatty acid metabolism.citrate shuttle.mitochondrial dicarboxylate transporter | BnaA02g04450D | A0A078I266 | Mitochondrialcarrier(TC2.A.29)family,(rape)hypotheticalproteinOS=BrassicanapusOX=3708GN=BnaA02g04450DPE=3SV=1 |
| Lipid metabolism.fatty acid metabolism.fatty acid degradation.alpha-oxidation.alpha dioxygenase | HID58_020315 | A0ABQ8DFA4 | GPI ethanolamine phosphate transferase 1 protein OS=Brassica napus OX=3708 GN=HID58_020315 PE=4 SV=1 |
| Lipid metabolism.fatty acid metabolism.fatty acid degradation.glyoxylate cycle.isocitrate lyase | HID58_067334 | A0ABQ7ZIU3 | UncharacterizedproteinOS=BrassicanapusOX=3708GN=HID58_067334PE=4SV=1 |
| Lipid metabolism.fatty acid metabolism.fatty acid degradation.glyoxylate cycle.peroxisomal NAD-dependent malate dehydrogenase | HID58_004182 | A0ABQ8E513 | Malate dehydrogenase protein (Fragment) OS=Brassica napus OX=3708 GN=HID58_004182 PE=4 SV=1 |
| Lipid metabolism.fatty acid metabolism.fatty acid degradation.glyoxylate cycle.peroxisomal NAD-dependent malate dehydrogenase | BnaA04g13230D | A0A078GJU5 | MalatedehydrogenaseOS=BrassicanapusOX=3708GN=BnaA04g13230DPE=3SV=1 |
| Lipid metabolism.fatty acid metabolism.fatty acid degradation.peroxisomal beta-oxidation.long-chain acyl-CoA oxidase *(ACX1/5) | BnaA01g18160D | A0A078I1I6 | Acyl-coenzymeAoxidaseOS=BrassicanapusOX=3708GN=BnaA01g18160DPE=3SV=1 |
| Lipid metabolism.fatty acid metabolism.fatty acid degradation.peroxisomal beta-oxidation.multifunctional enoyl-CoA hydratase & 3-hydroxyacyl-CoA dehydrogenase *(AIM1/MFP2) | HID58_011049 | A0ABQ8DX31 | Glyoxysomalfattyacidbeta-oxidationmultifunctionalproteinMFP-a(Fragment)OS=BrassicanapusOX=3708GN=HID58_011049PE=4SV=1 |
| Lipid metabolism.fatty acid metabolism.fatty acid degradation.peroxisomal beta-oxidation.short-chain acyl-CoA oxidase *(ACX4) | DARMORV10_C04P41140.1 | A0A816K0C0 | Acyl-coenzymeAoxidase4,peroxisomal(rape)hypotheticalproteinOS=BrassicanapusOX=3708GN=DARMORV10_C04P41140.1PE=3SV=1 |
| Lipid metabolism.fatty acid metabolism.plastidial fatty acid synthase (ptFAS) system.ketoacyl-ACP synthase (ptKAS) activities.ketoacyl-ACP synthase II | HID58_026944 | A0ABQ8CQD4 | Beta-ketoacyl-[acyl-carrier-protein]synthaseIOS=BrassicanapusOX=3708GN=HID58_026944PE=4SV=1 |
| Lipid metabolism.glycerolipid metabolism.glycerolipid degradation.phospholipase activities.phospholipase A1 activities.phospholipase-A1 *(PC-PLA1) | DARMORV10_C04P69860.1 | A0A816JYJ5 | PhospholipaseA1OS=BrassicanapusOX=3708GN=DARMORV10_C04P69860.1PE=3SV=1 |
| Lipid metabolism.glycerolipid metabolism.glycerolipid degradation.phospholipase activities.phospholipase C activities.phospholipase-C *(nPLC) | BnaC05g05030D | A0A078F6W4 | PhospholipaseC,(rape)hypotheticalproteinOS=BrassicanapusOX=3708GN=BnaC05g05030DPE=3SV=1 |
| Lipid metabolism.glycerolipid metabolism.glycerolipid degradation.triacylglycerol lipase activities.monoacylglycerol lipase | HID58_016313 | A0ABQ8DMK0 | Serine aminopeptidase S33 domain-containing protein OS=Brassica napus OX=3708 GN=HID58_016313 PE=4 SV=1 |
| Lipid metabolism.glycerolipid metabolism.phosphatidylcholine biosynthesis.choline homeostasis.choline kinase *(CEK) | DARMORV10_C06P45740.1 | A0A816QPB2 | Cholinekinase1,(rape)hypotheticalproteinOS=BrassicanapusOX=3708GN=DARMORV10_C06P45740.1PE=3SV=1 |
| Lipid metabolism.glycerolipid metabolism.phosphatidylethanolamine metabolism.CDP-ethanolamine pathway.CTP:phosphorylethanolamine cytidylyltransferase *(PECT1) | BnaA03g18010D | A0A078F013 | Ethanolamine-phosphatecytidylyltransferaseOS=BrassicanapusOX=3708GN=BnaA03g18010DPE=3SV=1 |
| Lipid metabolism.glycerolipid metabolism.phosphatidylethanolamine metabolism.N-acylethanolamine metabolism.lyso-glycerophospholipid acyltransferase *(NAPES) | HID58_073559 | A0ABQ7Z7L6 | Folate gamma-glutamyl hydrolase protein (Fragment) OS=Brassica napus OX=3708 GN=HID58_073559 PE=4 SV=1 |
| Lipid metabolism.phytosterol metabolism.plant sterol pathway.sterol delta24 reductase *(DWF1) | BnaA05g19350D | A0A078GJA7 | Delta(24)-sterolreductaseOS=BrassicanapusOX=3708GN=BnaA05g19350DPE=4SV=1 |
| Lipid metabolism.plastoglobule-associated activities.Fibrillin plastoglobule core protein *(FBN1/2) | HID58_010629 | A0ABQ8DVT6 | Plastidlipid-associatedprotein1,chloroplasticOS=BrassicanapusOX=3708GN=HID58_010629PE=4SV=1 |
| Lipid metabolism.plastoglobule-associated activities.Fibrillin plastoglobule core protein *(FBN1/2) | BnaC09g37690D | A0A078HDD3 | plastid-lipid-associatedprotein8,chloroplasticBnaC09g37690DproteinOS=BrassicanapusOX=3708GN=BnaC09g37690DPE=3SV=1 |
| Lipid metabolism.plastoglobule-associated activities.Fibrillin plastoglobule core protein *(FBN8) | DARMORV10_C04P01080.1 | A0A816J9K0 | Plastidlipid-associatedprotein/fibrillinconserveddomain-containingprotein,(rape)hypotheticalproteinOS=BrassicanapusOX=3708GN=DARMORV10_C04P01080.1PE=3SV=1 |
| Multi-process regulation.calcium homeostasis.Ca2+-dependent signalling.calcium sensor and kinase *(CPK1/2/++) | DARMORV10_A07P41530.1 | A0A816ZBX6 | Non-specificserine/threonineproteinkinaseOS=BrassicanapusOX=3708GN=DARMORV10_A07P41530.1PE=3SV=1 |
| Multi-process regulation.calcium homeostasis.Ca2+-dependent signalling.calcium sensor and kinase *(CPK1/2/++) | DARMORV10_C01P43710.1 | A0A816RME9 | Non-specificserine/threonineproteinkinaseOS=BrassicanapusOX=3708GN=DARMORV10_C01P43710.1PE=3SV=1 |
| Multi-process regulation.calcium homeostasis.Ca2+-dependent signalling.calcium sensor and kinase *(CPK1/2/++) | HID58_050529 | A0ABQ8A7S2 | Non-specificserine/threonineproteinkinaseOS=BrassicanapusOX=3708GN=HID58_050529PE=4SV=1 |
| Multi-process regulation.circadian clock system.morning element regulation.regulatory protein *(LWD1/2) | HID58_036604 | A0ABQ8C9P0 | Exportin-1/Importin-beta-like domain-containing protein OS=Brassica napus OX=3708 GN=HID58_036604 PE=4 SV=1 |
| Multi-process regulation.G-protein-dependent signalling.heterotrimeric G-protein complex.canonical subunit alpha *(GPA) | DARMORV10_C08P38230.1 | A0A816UGE8 | Guaninenucleotide-bindingproteinalphasubunitOS=BrassicanapusOX=3708GN=DARMORV10_C08P38230.1PE=3SV=1 |
| Multi-process regulation.MAP kinase cascade signalling.MAP2K protein kinase activities.MAP2K protein kinase *(MKK4/5/++) | DARMORV10_C03P51530.1 | A0A816IBF3 | Calcium-transportingATPaseOS=BrassicanapusOX=3708GN=DARMORV10_C03P51530.1PE=3SV=1 |
| Multi-process regulation.MAP kinase cascade signalling.MAP3K-RAF/subgroup-C protein kinase activities.subgroup-C1 protein kinase *(ILK1/2/++) | HID58_083475 | A0ABQ7YEX5 | Non-specificserine/threonineproteinkinaseproteinOS=BrassicanapusOX=3708GN=HID58_083475PE=4SV=1 |
| Multi-process regulation.MAP kinase cascade signalling.MAP3K-RAF/subgroup-C protein kinase activities.subgroup-C1 protein kinase *(ILK1/2/++) | HID58_000978 | A0ABQ8EI45 | Protein kinase domain-containing protein OS=Brassica napus OX=3708 GN=HID58_000978 PE=4 SV=1 |
| Multi-process regulation.MAP kinase cascade signalling.MAP3K-RAF/subgroup-C protein kinase activities.subgroup-C7 protein kinase *(RAF33/38/++) | DARMORV10_C08P37950.1 | A0A816UMB6 | Non-specificserine/threonineproteinkinaseOS=BrassicanapusOX=3708GN=DARMORV10_C08P37950.1PE=4SV=1 |
| Multi-process regulation.MAP kinase cascade signalling.MAPK protein kinase *(MPK1/2/++) | HID58_026626 | A0ABQ8CRN5 | Mitogen-activatedproteinkinaseOS=BrassicanapusOX=3708GN=HID58_026626PE=4SV=1 |
| Multi-process regulation.MAP kinase cascade signalling.YDA-MAP kinase signalling module.MAP2K protein kinase *(MKK4/5) | DARMORV10_C03P51530.1 | A0A816IBF3 | Calcium-transportingATPaseOS=BrassicanapusOX=3708GN=DARMORV10_C03P51530.1PE=3SV=1 |
| Multi-process regulation.nitric oxide signalling.homeostasis.class-1/2 phytoglobin *(PGB1/2) | HID58_044481 | A0ABQ8BJL2 | Globin domain-containing protein OS=Brassica napus OX=3708 GN=HID58_044481 PE=4 SV=1 |
| Multi-process regulation.phosphoinositide-dependent signalling.inositol phosphate homeostasis.inositol hexakisphosphate metabolism.regulatory protein kinase *(IPCK) | HID58_004616 | A0ABQ8E6A0 | Receptor-like serine/threonine-protein kinase OS=Brassica napus OX=3708 GN=HID58_004616 PE=4 SV=1 |
| Multi-process regulation.phosphoinositide-dependent signalling.inositol phosphate homeostasis.inositol monophosphate phosphatase activities.myo-inositol-1-phosphate phosphatase *(VTC4) | DARMORV10_A05P44240.1 | A0A816U1P6 | Inositol-1-monophosphataseOS=BrassicanapusOX=3708GN=DARMORV10_A05P44240.1PE=3SV=1 |
| Multi-process regulation.phosphoinositide-dependent signalling.inositol phosphate homeostasis.lipid-dependent inositol phosphate biosynthesis.phosphatidylinositol phospholipase *(PI-PLC) | HID58_019921 | A0ABQ8DE51 | PhosphoinositidephospholipaseCproteinOS=BrassicanapusOX=3708GN=HID58_019921PE=4SV=1 |
| Multi-process regulation.phosphoinositide-dependent signalling.phosphoinositide homeostasis.phosphatidylinositol 4-kinase activities.phosphatidylinositol 4-kinase (PI4K-alpha) complex.component *(EFOP) | BnaC08g44520D | A0A078FAF5 | ProteinSEMI-ROLLEDLEAF2,(rape)hypotheticalproteinOS=BrassicanapusOX=3708GN=BnaC08g44520DPE=4SV=1 |
| Multi-process regulation.phosphoinositide-dependent signalling.phosphoinositide homeostasis.phosphatidylinositol 5-kinase activities.PIKfyve lipid kinase complex.scaffold protein component *(VAC14) | HID58_076493 | A0ABQ7YQR9 | Vacuolarprotein14C-terminalFig4-bindingdomain-containingproteinOS=BrassicanapusOX=3708GN=HID58_076493PE=4SV=1 |
| Multi-process regulation.Programmed Cell Death (PCD) system.vacuole-mediated cell death.cysteine protease *(XCP1/2) | DARMORV10_A08P29200.1 | A0A817AI73 | CysteineproteaseXCP2,(rape)hypotheticalproteinOS=BrassicanapusOX=3708GN=DARMORV10_A08P29200.1PE=3SV=1 |
| Multi-process regulation.Programmed Cell Death (PCD) system.vacuole-mediated cell death.cysteine proteinase *(VPE) | HID58_040444 | A0ABQ8B833 | LegumainproteinOS=BrassicanapusOX=3708GN=HID58_040444PE=4SV=1 |
| Multi-process regulation.pyrophosphate homeostasis.cytosolic pyrophosphatase | HID58_074233 | A0ABQ7YGC9 | Inorganic diphosphatase protein OS=Brassica napus OX=3708 GN=HID58_074233 PE=4 SV=1 |
| Multi-process regulation.pyrophosphate homeostasis.cytosolic pyrophosphatase | BnaC04g27080D | A0A078H1Z4 | InorganicdiphosphataseOS=BrassicanapusOX=3708GN=BnaC04g27080DPE=3SV=1 |
| Multi-process regulation.TOR (Target Of Rapamycin) signalling.regulatory protein *(TCTP1/TCTP2) | DARMORV10_C03P48370.1 | A0A816I965 | Translationally-controlledtumorproteinhomolog,(rape)hypotheticalproteinOS=BrassicanapusOX=3708GN=DARMORV10_C03P48370.1PE=3SV=1 |
| Multi-process regulation.TOR (Target Of Rapamycin) signalling.TOR kinase substrates.TOR-dependent regulatory protein *(MRF) of protein translation | HID58_041695 | A0ABQ8BBJ9 | MI domain-containing protein OS=Brassica napus OX=3708 GN=HID58_041695 PE=4 SV=1 |
| Multi-process regulation.UPR (Unfolded Protein Response) signalling.IRE1-bZIP60 pathway.regulatory mediator *(SVB) | DARMORV10_A01P27560.1 | A0A816Y294 | (rape)hypotheticalproteinOS=BrassicanapusOX=3708GN=DARMORV10_A01P27560.1PE=4SV=1 |
| Multi-process regulation.UPR (Unfolded Protein Response) signalling.IRE1-bZIP60 pathway.regulatory protein *(PDI9) | HID58_051828 | A0ABQ8AA27 | Protein disulfide-isomerase OS=Brassica napus OX=3708 GN=HID58_051828 PE=4 SV=1 |
| not assigned.annotated | DARMORV10_A01P38740.1 | A0A816Y6T0 | (rape)hypotheticalproteinOS=BrassicanapusOX=3708GN=DARMORV10_A01P38740.1PE=4SV=1 |
| not assigned.annotated | DARMORV10_A09P17610.1 | A0A816NUK8 | (rape)hypotheticalproteinOS=BrassicanapusOX=3708GN=DARMORV10_A09P17610.1PE=4SV=1 |
| not assigned.annotated | BnaA03g01260D | A0A078FA30 | Acyl-CoA-bindingdomain-containingprotein,(rape)hypotheticalproteinOS=BrassicanapusOX=3708GN=BnaA03g01260DPE=4SV=1 |
| not assigned.annotated | HID58_080431 | A0ABQ7Y807 | Agenet domain-containing protein (Fragment) OS=Brassica napus OX=3708 GN=HID58_080431 PE=4 SV=1 |
| not assigned.annotated | HID58_072186 | A0ABQ7Z3W1 | ATP-dependentRNAhelicaseOS=BrassicanapusOX=3708GN=HID58_072186PE=4SV=1 |
| not assigned.annotated | DARMORV10_C03P42830.1 | A0A816I942 | Auxin-inducedinrootculturesprotein12-like,(rape)hypotheticalproteinOS=BrassicanapusOX=3708GN=DARMORV10_C03P42830.1PE=4SV=1 |
| not assigned.annotated | BnaA03g11110D | A0A078HEI6 | BnaA03g11110DproteinOS=BrassicanapusOX=3708GN=BnaA03g11110DPE=3SV=1 |
| not assigned.annotated | HID58_081745 | A0ABQ7Y8M4 | Coiled-coil domain-containing protein SCD2 protein OS=Brassica napus OX=3708 GN=HID58_081745 PE=4 |
| not assigned.annotated | HID58_022023 | A0ABQ8CY15 | DehydrinproteinOS=BrassicanapusOX=3708GN=HID58_022023PE=4SV=1 |
| not assigned.annotated | HID58_040355 | A0ABQ8B7S2 | DJ-1/PfpIdomain-containingproteinOS=BrassicanapusOX=3708GN=HID58_040355PE=4SV=1 |
| not assigned.annotated | DARMORV10_A01P05910.1 | A0A816XKQ3 | DUF642domain-containingprotein,(rape)hypotheticalproteinOS=BrassicanapusOX=3708GN=DARMORV10_A01P05910.1PE=4SV=1 |
| not assigned.annotated | DARMORV10_A02P42180.1 | A0A816XC64 | Embryo-specificproteinATS3B-like,(rape)hypotheticalproteinOS=BrassicanapusOX=3708GN=DARMORV10_A02P42180.1PE=4SV=1 |
| not assigned.annotated | DARMORV10_C06P52730.1 | A0A816QU66 | EP1-likeglycoprotein2,(rape)hypotheticalproteinOS=BrassicanapusOX=3708GN=DARMORV10_C06P52730.1PE=4SV=1 |
| not assigned.annotated | HID58_082340 | A0ABQ7YAA3 | Glucose-methanol-choline oxidoreductase N-terminal domain-containing protein OS=Brassica napus OX=3708 GN=HID58_082340 PE=4 SV=1 |
| not assigned.annotated | DARMORV10_A10P28690.1 | A0A817BF91 | Heparanase-likeprotein1,(rape)hypotheticalproteinOS=BrassicanapusOX=3708GN=DARMORV10_A10P28690.1PE=3SV=1 |
| not assigned.annotated | HID58_020681 | A0ABQ8CVP8 | Legume lectin domain-containing protein OS=Brassica napus OX=3708 GN=HID58_020681 PE=4 SV=1 |
| not assigned.annotated | HID58_000988 | A0ABQ8EID7 | MethyltransferaseproteinOS=BrassicanapusOX=3708GN=HID58_000988PE=4SV=1 |
| not assigned.annotated | BnaA01g29080D | A0A078G3X4 | Peptide-N4-(N-acetyl-beta-glucosaminyl)asparagineamidaseA-like,(rape)hypotheticalproteinOS=BrassicanapusOX=3708GN=BnaA01g29080DPE=4SV=1 |
| not assigned.annotated | DARMORV10_C03P57040.1 | A0A816IE78 | PurpleacidphosphataseOS=BrassicanapusOX=3708GN=DARMORV10_C03P57040.1PE=3SV=1 |
| not assigned.annotated | rbcL | A0A482K1T8 | Ribulosebisphosphatecarboxylaselargechain(Fragment)OS=BrassicanapusOX=3708GN=rbcLPE=3SV=1 |
| not assigned.annotated | HID58_012881 | A0ABQ8E2C1 | S1 motif domain-containing protein OS=Brassica napus OX=3708 GN=HID58_012881 PE=4 SV=1 |
| not assigned.annotated | DARMORV10_C04P51900.1 | A0A816JSD3 | Uncharacterizedprotein(rape)hypotheticalproteinOS=BrassicanapusOX=3708GN=DARMORV10_C04P51900.1PE=4SV=1 |
| not assigned.annotated | HID58_004297 | A0ABQ8E5D1 | UncharacterizedproteinOS=BrassicanapusOX=3708GN=HID58_004297PE=4SV=1 |
| not assigned.not annotated | DARMORV10_C04P64000.1 | A0A816JW56 | (rape)hypotheticalproteinOS=BrassicanapusOX=3708GN=DARMORV10_C04P64000.1PE=4SV=1 |
| not assigned.not annotated | DARMORV10_C08P17640.1 | A0A816U3Z1 | Appledomain-containingprotein,(rape)hypotheticalproteinOS=BrassicanapusOX=3708GN=DARMORV10_C08P17640.1PE=4SV=1 |
| not assigned.not annotated | HID58_046245 | A0ABQ8AWH0 | Bifunctionalinhibitor/plantlipidtransferprotein/seedstoragehelicaldomain-containingprotein(Fragment)OS=BrassicanapusOX=3708GN=HID58_046245PE=4SV=1 |
| not assigned.not annotated | HID58_039811 | A0ABQ8BT57 | GSTN-terminaldomain-containingproteinOS=BrassicanapusOX=3708GN=HID58_039811PE=4SV=1 |
| not assigned.not annotated | BnaCnng57090D | A0A078JRA0 | Helitronhelicase-likedomain-containingprotein,BnaCnng57090DproteinOS=BrassicanapusOX=3708GN=BnaCnng57090DPE=4SV=1 |
| not assigned.not annotated | DARMORV10_C03P46600.1 | A0A816IH85 | PutativehydrolaseC777.06c,(rape)hypotheticalproteinOS=BrassicanapusOX=3708GN=DARMORV10_C03P46600.1PE=4SV=1 |
| not assigned.not annotated | HID58_044731 | A0ABQ8BLL2 | RCC1-like domain-containing protein OS=Brassica napus OX=3708 GN=HID58_044755 PE=4 SV=1 |
| not assigned.not annotated | HID58_024203 | A0ABQ8D481 | RimMN-terminaldomain-containingproteinOS=BrassicanapusOX=3708GN=HID58_024203PE=4SV=1 |
| not assigned.not annotated | HID58_032300 | A0ABQ8BVZ4 | RuvB-like helicase protein OS=Brassica napus OX=3708 GN=HID58_032300 PE=4 SV=1 |
| not assigned.not annotated | HID58_065823 | A0ABQ7ZDY6 | Uncharacterizedprotein(Fragment)OS=BrassicanapusOX=3708GN=HID58_065823PE=4SV=1 |
| not assigned.not annotated | DARMORV10_C09P36020.1 | A0A816IZ13 | Uncharacterizedprotein(rape)hypotheticalproteinOS=BrassicanapusOX=3708GN=DARMORV10_C09P36020.1PE=4SV=1 |
| not assigned.not annotated | DARMORV10_C09P45650.1 | A0A816IZT2 | Uncharacterizedprotein(rape)hypotheticalproteinOS=BrassicanapusOX=3708GN=DARMORV10_C09P45650.1PE=4SV=1 |
| not assigned.not annotated | HID58_018577 | A0ABQ8DAD9 | UncharacterizedproteinOS=BrassicanapusOX=3708GN=HID58_018577PE=4SV=1 |
| not assigned.not annotated | HID58_025123 | A0ABQ8CKA8 | UncharacterizedproteinOS=BrassicanapusOX=3708GN=HID58_025123PE=4SV=1 |
| not assigned.not annotated | HID58_029245 | A0ABQ8CCJ5 | UncharacterizedproteinOS=BrassicanapusOX=3708GN=HID58_029245PE=4SV=1 |
| not assigned.not annotated | HID58_075901 | A0ABQ7YKZ5 | UncharacterizedproteinOS=BrassicanapusOX=3708GN=HID58_075901PE=4SV=1 |
| Nucleotide metabolism.non-canonical nucleotide metabolism.nucleoside monophosphoramidate hydrolase *(HINT1/2) | HID58_025493 | A0ABQ8CLA4 | HITdomain-containingproteinOS=BrassicanapusOX=3708GN=HID58_025493PE=4SV=1 |
| Nucleotide metabolism.purines.catabolism.S-allantoin synthase *(TTL) | HID58_046011 | A0ABQ8AVC4 | Hydroxyisourate hydrolase (Fragment) OS=Brassica napus OX=3708 GN=HID58_046011 PE=4 SV=1 |
| Nucleotide metabolism.purines.ribonucleotide (RN) anabolism.adenylosuccinate lyase *(PUR8) | DARMORV10_C01P12350.1 | A0A816R3B4 | AdenylosuccinatelyaseOS=BrassicanapusOX=3708GN=DARMORV10_C01P12350.1PE=3SV=1 |
| Nucleotide metabolism.purines.ribonucleotide (RN) anabolism.adenylosuccinate synthetase *(PUR11) | BnaCnng07310D | A0A078HAE4 | Adenylosuccinatesynthetase,chloroplasticOS=BrassicanapusOX=3708GN=BnaCnng07310DPE=3SV=1 |
| Nucleotide metabolism.purines.ribonucleotide (RN) anabolism.adenylosuccinate synthetase *(PUR11) | PURA | A0A816ULE3 | Adenylosuccinatesynthetase,chloroplasticOS=BrassicanapusOX=3708GN=PURAPE=3SV=1 |
| Nucleotide metabolism.purines.ribonucleotide (RN) anabolism.formylglycinamidine RN synthase *(PUR4) | HID58_073223 | A0ABQ7Z6R8 | PhosphoribosylformylglycinamidinesynthaseOS=BrassicanapusOX=3708GN=HID58_073223PE=4SV=1 |
| Nucleotide metabolism.pyrimidines.catabolism.beta-ureidopropionase *(PYD3) | BnaAnng15120D | A0A078J5Y0 | Beta-ureidopropionase,OS=BrassicanapusOX=3708GN=BnaAnng15120DPE=3SV=1 |
| Nutrient uptake.nitrogen assimilation.ammonium assimilation.glutamine synthetase activities.cytosolic glutamine synthetase *(GLN1) | HID58_088966 | A0ABQ7XXM9 | Glutamine synthetase protein OS=Brassica napus OX=3708 GN=HID58_088966 PE=4 SV=1 |
| Nutrient uptake.nitrogen assimilation.ammonium assimilation.glutamine synthetase activities.cytosolic glutamine synthetase *(GLN1) | gln | Q42623 | GlutaminesynthetaseOS=BrassicanapusOX=3708GN=glnPE=2SV=1 |
| Nutrient uptake.nitrogen assimilation.ammonium assimilation.glutamine synthetase activities.cytosolic glutamine synthetase *(GLN1) | HID58_053968 | A0ABQ8AG61 | GlutaminesynthetaseproteinOS=BrassicanapusOX=3708GN=HID58_053968PE=4SV=1 |
| Nutrient uptake.nitrogen assimilation.aspartate aminotransferase *(ASP) | HID58_060330 | A0ABQ7ZVE1 | Multifunctional fusion protein OS=Brassica napus OX=3708 GN=HID58_060330 PE=4 SV=1 |
| Nutrient uptake.nitrogen assimilation.nitrate uptake system.regulatory factor *(NRT3) | HID58_041689 | A0ABQ8BBK7 | Manganese-dependentADP-ribose/CDP-alcoholdiphosphataseproteinOS=BrassicanapusOX=3708GN=HID58_041689PE=4SV=1 |
| Nutrient uptake.other micronutrient homeostasis.metallochaperone activities.nucleotide-dependent metallochaperone *(ZNG1) | HID58_065907 | A0ABQ7ZEJ1 | CobWC-terminaldomain-containingprotein(Fragment)OS=BrassicanapusOX=3708GN=HID58_065907PE=4SV=1 |
| Nutrient uptake.phosphorus assimilation.phosphate uptake.phosphate transporter *(PHT1) | HID58_073396 | A0ABQ7Z734 | Major facilitator superfamily (MFS) profile domain-containing protein (Fragment) OS=Brassica napus OX=3708 GN=HID58_073396 PE=4 SV=1 |
| Nutrient uptake.phosphorus assimilation.phosphate uptake.phosphate transporter trafficking factor *(PHF1) | DARMORV10_A09P45250.1 | A0A816PCI0 | Dualspecificityproteinphosphatase4OS=BrassicanapusOX=3708GN=DARMORV10_A09P45250.1PE=4SV=1 |
| Photosynthesis.calvin cycle.phosphoribulokinase *(PRK) | BnaA08g07000D | A0A078HT68 | PhosphoribulokinaseOS=BrassicanapusOX=3708GN=BnaA08g07000DPE=3SV=1 |
| Photosynthesis.calvin cycle.phosphoribulokinase *(PRK) | HID58_067145 | A0ABQ7ZHQ8 | PhosphoribulokinaseproteinOS=BrassicanapusOX=3708GN=HID58_067145PE=4SV=1 |
| Photosynthesis.calvin cycle.ribose 5-phosphate isomerase | BnaA01g33410D | A0A078G3Q8 | Ribose-5-phosphateisomeraseOS=BrassicanapusOX=3708GN=BnaA01g33410DPE=3SV=1 |
| Photosynthesis.calvin cycle.ribulose-1,5-bisphosphat carboxylase/oxygenase (RuBisCo) activity.RuBisCo assembly.assembly factor *(RAF2) | DARMORV10_C03P19210.1 | A0A816HYZ4 | 4a-HydroxytetrahydrobiopterindehydrataseOS=BrassicanapusOX=3708GN=DARMORV10_C03P19210.1PE=3SV=1 |
| Photosynthesis.calvin cycle.ribulose-1,5-bisphosphat carboxylase/oxygenase (RuBisCo) activity.RuBisCo assembly.auxiliary co-chaperone *(CPN20) | HID58_008941 | A0ABQ8DR46 | Trichome birefringence-like N-terminal domain-containing protein OS=Brassica napus OX=3708 GN=HID58_008941 PE=4 SV=1 |
| Photosynthesis.calvin cycle.ribulose-1,5-bisphosphat carboxylase/oxygenase (RuBisCo) activity.RuBisCo assembly.CPN60 chaperonin heterodimer.subunit alpha | DARMORV10_C04P23540.1 | A0A816JUZ4 | RuBisCOlargesubunit-bindingproteinsubunitalpha,chloroplastic,(rape)hypotheticalproteinOS=BrassicanapusOX=3708GN=DARMORV10_C04P23540.1PE=3SV=1 |
| Photosynthesis.calvin cycle.ribulose-1,5-bisphosphat carboxylase/oxygenase (RuBisCo) activity.RuBisCo regulation.lysine N-methyltransferase of small RuBisCo subunit *(SSMT) | HID58_044594 | A0ABQ8BLL9 | Ribosomal RNA-processing protein 42 OS=Brassica napus OX=3708 GN=HID58_044594 PE=4 SV=1 |
| Photosynthesis.calvin cycle.ribulose-1,5-bisphosphat carboxylase/oxygenase (RuBisCo) activity.RuBisCo regulation.RuBisCo inhibitor dephosphorylation.xylulose-1,5-bisphosphate phosphatase *(CbbYa) | HID58_022728 | A0ABQ8D036 | UncharacterizedproteinOS=BrassicanapusOX=3708GN=HID58_022728PE=4SV=1 |
| Photosynthesis.calvin cycle.sedoheptulose-1,7-bisphosphatase | HID58_026476 | A0ABQ8CR66 | Sedoheptulose-1,7-bisphosphatase,chloroplasticOS=BrassicanapusOX=3708GN=HID58_026476PE=4SV=1 |
| Photosynthesis.CAM/C4 photosynthesis.phosphoenolpyruvate (PEP) carboxylase activity.PEP carboxylase *(PPC) | DARMORV10_C05P52230.1 | A0A816L981 | PhosphoenolpyruvatecarboxylaseOS=BrassicanapusOX=3708GN=DARMORV10_C05P52230.1PE=3SV=1 |
| Photosynthesis.CAM/C4 photosynthesis.phosphoenolpyruvate (PEP) carboxylase activity.PEP carboxylase *(PPC) | PE3-PEPCase | Q42634 | PhosphoenolpyruvatecarboxylaseOS=BrassicanapusOX=3708GN=PE3-PEPCasePE=3SV=1 |
| Photosynthesis.photophosphorylation.ATP synthase complex.assembly.assembly factor *(BFA3) | HID58_036211 | A0ABQ8C736 | Kinesin-like protein OS=Brassica napus OX=3708 GN=HID58_036211 PE=4 SV=1 |
| Photosynthesis.photophosphorylation.ATP synthase complex.membrane CF0 subcomplex.subunit a | atpI | A0A1B1Y0D5 | ATPsynthasesubunita,chloroplasticOS=Brassicanapusvar.napusOX=138011GN=atpIPE=3SV=1 |
| Photosynthesis.photophosphorylation.chlororespiration.NADH dehydrogenase-like (NDH) complex.assembly and maintenance.assembly factor *(CRR1) | DARMORV10_C03P18520.1 | A0A816I5Y0 | DihydrodipicolinatereductaseN-terminaldomain-containingprotein(rape)hypotheticalproteinOS=BrassicanapusOX=3708GN=DARMORV10_C03P18520.1PE=3SV=1 |
| Photosynthesis.photophosphorylation.chlororespiration.NADH dehydrogenase-like (NDH) complex.assembly and maintenance.assembly factor *(CRR41) | BnaC06g04300D | A0A078GFE6 | Chlororespiratoryreduction41,BnaC06g04300DproteinOS=BrassicanapusOX=3708GN=BnaC06g04300DPE=4SV=1 |
| Photosynthesis.photophosphorylation.chlororespiration.NADH dehydrogenase-like (NDH) complex.assembly and maintenance.Cpn60 chaperonin heterodimer.subunit alpha | DARMORV10_C04P23540.1 | A0A816JUZ4 | RuBisCOlargesubunit-bindingproteinsubunitalpha,chloroplastic,(rape)hypotheticalproteinOS=BrassicanapusOX=3708GN=DARMORV10_C04P23540.1PE=3SV=1 |
| Photosynthesis.photophosphorylation.chlororespiration.NADH dehydrogenase-like (NDH) complex.subcomplex A.component *(NdhM) | BnaC03g61160D | A0A078IKT4 | NAD(P)H-quinoneoxidoreductasesubunitM,chloroplasticOS=BrassicanapusOX=3708GN=BnaC03g61160DPE=3SV=1 |
| Photosynthesis.photophosphorylation.chlororespiration.NADH dehydrogenase-like (NDH) complex.subcomplex B.component *(PnsB5/NDH18) | BnaA02g22320D | A0A078FKB8 | PhotosyntheticNDHsubunitofsubcomplexB5,chloroplastic,BnaA02g22320DproteinOS=BrassicanapusOX=3708GN=BnaA02g22320DPE=4SV=1 |
| Photosynthesis.photophosphorylation.Cytb6/f to PS-I electron carriers.plastocyanin copper-protein *(PetE) | DARMORV10_A07P41660.1 | A0A816ZC43 | PlastocyaninOS=BrassicanapusOX=3708GN=DARMORV10_A07P41660.1PE=3SV=1 |
| Photosynthesis.photophosphorylation.Cytb6/f to PS-I electron carriers.plastocyanin copper-protein *(PetE) | DARMORV10_C06P50040.1 | A0A816QW44 | PlastocyaninOS=BrassicanapusOX=3708GN=DARMORV10_C06P50040.1PE=3SV=1 |
| Photosynthesis.photophosphorylation.cytochrome b6/f complex.assembly.CCB cytochrome b6 maturation system (system IV).assembly factor *(CCB1) | HID58_007238 | A0ABQ8EDM9 | Protein kinase domain-containing protein OS=Brassica napus OX=3708 GN=HID58_007238 PE=4 SV=1 |
| Photosynthesis.photophosphorylation.cytochrome b6/f complex.component *(PetD/IV) | petD | A0A1B1Y0C1 | Cytochromeb6-fcomplexsubunit4OS=Brassicanapusvar.napusOX=138011GN=petDPE=3SV=1 |
| Photosynthesis.photophosphorylation.cytochrome b6/f complex.component *(PetM/VII) | BnaA09g40450D | A0A078FEV0 | (rape)hypotheticalproteinOS=BrassicanapusOX=3708GN=BnaA09g40450DPE=3SV=1 |
| Photosynthesis.photophosphorylation.linear electron flow.ferredoxin-NADP reductase activity.ferredoxin-NADP reductase membrane-tethering.recruitment protein *(TROL) | DARMORV10_C09P00870.1 | A0A816IRH3 | Rhodanese-likedomain-containingprotein4,chloroplastic(rape)hypotheticalproteinOS=BrassicanapusOX=3708GN=DARMORV10_C09P00870.1PE=4SV=1 |
| Photosynthesis.photophosphorylation.linear electron flow.ferredoxin-NADP reductase activity.regulatory protein *(LIR1) | HID58_007238 | A0ABQ8EDM9 | Protein kinase domain-containing protein OS=Brassica napus OX=3708 GN=HID58_007238 PE=4 SV=1 |
| Photosynthesis.photophosphorylation.photosystem I | LHB1B2 | Q2I0E4 | Chlorophylla-bbindingprotein,chloroplastic(Fragment)OS=BrassicanapusOX=3708GN=LHB1B2PE=2SV=1 |
| Photosynthesis.photophosphorylation.photosystem I | BnaC05g37470D | A0A078GUF2 | ProteinPOST-ILLUMINATIONCHLOROPHYLLFLUORESCENCEINCREASE,chloroplastic,(rape)hypotheticalproteinOS=BrassicanapusOX=3708GN=BnaC05g37470DPE=4SV=1 |
| Photosynthesis.photophosphorylation.photosystem I | DARMORV10_C01P20210.1 | A0A816RAG3 | Thylakoidlumenal17.9kDaprotein,chloroplastic,(rape)hypotheticalproteinOS=BrassicanapusOX=3708GN=DARMORV10_C01P20210.1PE=4SV=1 |
| Photosynthesis.photophosphorylation.photosystem I.LHC-I complex.component *(LHCa2) | HID58_026674 | A0ABQ8CPL0 | Beta-galactosidaseproteinOS=BrassicanapusOX=3708GN=HID58_026674PE=4SV=1 |
| Photosynthesis.photophosphorylation.photosystem I.LHC-I complex.component *(LHCa3) | BnaA01g22670D | A0A078ICK1 | Chlorophylla-bbindingprotein,chloroplasticOS=BrassicanapusOX=3708GN=BnaA01g22670DPE=3SV=1 |
| Photosynthesis.photophosphorylation.photosystem I.PS-I complex.apoprotein component *(PsaA) | psaA | A0A1B1Y0E3 | PhotosystemIP700chlorophyllaapoproteinA1OS=Brassicanapusvar.napusOX=138011GN=psaAPE=3SV=1 |
| Photosynthesis.photophosphorylation.photosystem I.PS-I complex.component *(PsaO) | DARMORV10_C05P06520.1 | A0A816KQY7 | PhotosystemIsubunitO,(rape)hypotheticalproteinOS=BrassicanapusOX=3708GN=DARMORV10_C05P06520.1PE=4SV=1 |
| Photosynthesis.photophosphorylation.photosystem II.assembly.assembly factor *(LPA1) | HID58_079029 | A0ABQ7Y0V4 | Glutathione transferase protein OS=Brassica napus OX=3708 GN=HID58_079029 PE=4 SV=1 |
| Photosynthesis.photophosphorylation.photosystem II.assembly.LHCII-stabilizing factor *(SEP3) | HID58_024158 | A0ABQ8D438 | CaseinkinaseIIsubunitbetaproteinOS=BrassicanapusOX=3708GN=HID58_024158PE=4SV=1 |
| Photosynthesis.photophosphorylation.photosystem II.assembly.psbA-translation activator *(HCF173) | BnaA06g11240D | A0A078HCC1 | proteinHIGHCHLOROPHYLLFLUORESCENCEPHENOTYPE173,chloroplastic,(rape)hypotheticalproteinOS=BrassicanapusOX=3708GN=BnaA06g11240DPE=4SV=1 |
| Photosynthesis.photophosphorylation.photosystem II.LHC-II complex.component *(LHCb1/2/3) | BnaA09g26570D | A0A078GLT1 | Chlorophylla-bbindingprotein,chloroplasticOS=BrassicanapusOX=3708GN=BnaA09g26570DPE=3SV=1 |
| Photosynthesis.photophosphorylation.photosystem II.photosynthetic acclimation.acclimation factor activities.acclimation factor *(MPH2) | HID58_048015 | A0ABQ8B1T5 | Glutathione transferase protein OS=Brassica napus OX=3708 GN=HID58_048015 PE=4 SV=1 |
| Photosynthesis.photophosphorylation.photosystem II.photosynthetic acclimation.non-photochemical quenching (NPQ).qE/qZ-type quenching.regulatory protein *(FLAP1) | DARMORV10_A01P18990.1 | A0A816XSM9 | Uncharacterizedprotein(rape)hypotheticalproteinOS=BrassicanapusOX=3708GN=DARMORV10_A01P18990.1PE=4SV=1 |
| Photosynthesis.photophosphorylation.photosystem II.photosynthetic acclimation.non-photochemical quenching (NPQ).qE/qZ-type quenching.xanthophyll cycle.zeaxanthin epoxidase *(ZEP) | DARMORV10_C09P11330.1 | A0A816J0G2 | Zeaxanthinepoxidase,chloroplasticOS=BrassicanapusOX=3708GN=DARMORV10_C09P11330.1PE=4SV=1 |
| Photosynthesis.photophosphorylation.photosystem II.photosynthetic acclimation.regulatory protein modification.lysine acetyltransferase *(NSI) | DARMORV10_A05P25290.1 | A0A816TP41 | HistoneacetyltransferaseOS=BrassicanapusOX=3708GN=DARMORV10_A05P25290.1PE=3SV=1 |
| Photosynthesis.photophosphorylation.photosystem II.PS-II complex.oxygen-evolving center (OEC) extrinsic components.component *(OEC33/PsbO) | HID58_042447 | A0ABQ8BDP2 | CASP-like protein OS=Brassica napus OX=3708 GN=HID58_042447 PE=4 SV=1 |
| Photosynthesis.photophosphorylation.photosystem II.PS-II complex.reaction center complex.component *(D2/PsbD) | DARMORV10_C09P40430.1 | A0A816JB71 | PhotosystemIID2proteinOS=BrassicanapusOX=3708GN=DARMORV10_C09P40430.1PE=3SV=1 |
| Photosynthesis.photophosphorylation.photosystem II.repair and reassembly cycle.assembly factor *(HHL1) | HID58_072465 | A0ABQ7Z4J6 | Peptidase M3A/M3B catalytic domain-containing protein (Fragment) OS=Brassica napus OX=3708 GN=HID58_072465 PE=4 SV=1 |
| Photosynthesis.photophosphorylation.photosystem II.repair and reassembly cycle.assembly factor *(MET1) | BnaA05g13750D | A0A078F5A7 | ProteinMET1,chloroplastic,(rape)hypotheticalproteinOS=BrassicanapusOX=3708GN=BnaA05g13750DPE=4SV=1 |
| Photosynthesis.photophosphorylation.photosystem II.repair and reassembly cycle.photoprotective factor *(MPH1) | DARMORV10_C09P69150.1 | A0A816J6H1 | ProteinMAINTENANCEOFPSIIUNDERHIGHLIGHT1-like(rape)hypotheticalproteinOS=BrassicanapusOX=3708GN=DARMORV10_C09P69150.1PE=4SV=1 |
| Photosynthesis.photorespiration.glycerate:glycolate transporter *(PLGG1) | DARMORV10_A05P25290.1 | A0A816TP41 | HistoneacetyltransferaseOS=BrassicanapusOX=3708GN=DARMORV10_A05P25290.1PE=3SV=1 |
| Phytohormone action.abscisic acid.biosynthesis.zeaxanthin epoxidase *(ABA1) | DARMORV10_C09P11330.1 | A0A816J0G2 | Zeaxanthinepoxidase,chloroplasticOS=BrassicanapusOX=3708GN=DARMORV10_C09P11330.1PE=4SV=1 |
| Phytohormone action.abscisic acid.transport.abscisic acid transporter *(AIT) | HID58_072875 | A0ABQ7Z5U2 | Band 7 domain-containing protein OS=Brassica napus OX=3708 GN=HID58_072875 PE=4 SV=1 |
| Phytohormone action.auxin.perception and signal transduction.substrate adaptor *(TIR1/AFB) of SCF E3 ubiquitin ligase activity | DARMORV10_A03P27600.1 | A0A816VAQ7 | Transportinhibitorresponse1-likeprotein,(rape)hypotheticalproteinOS=BrassicanapusOX=3708GN=DARMORV10_A03P27600.1PE=4SV=1 |
| Phytohormone action.auxin.transport.auxin efflux.ABCB-type auxin transporter *(ABCB28) | BnaCnng49290D | A0A078JH18 | ABCtransporterBfamilymember28,BnaCnng49290DproteinOS=BrassicanapusOX=3708GN=BnaCnng49290DPE=4SV=1 |
| Phytohormone action.jasmonic acid.biosynthesis.acyl-CoA thioesterase *(ACH) | HID58_047896 | A0ABQ8B1L1 | Cyclic nucleotide-binding domain-containing protein OS=Brassica napus OX=3708 GN=HID58_047896 PE=4 SV=1 |
| Phytohormone action.jasmonic acid.biosynthesis.OPC-8:CoA oxidase *(ACX1/5) | BnaA01g18160D | A0A078I1I6 | Acyl-coenzymeAoxidaseOS=BrassicanapusOX=3708GN=BnaA01g18160DPE=3SV=1 |
| Phytohormone action.signalling peptides.CRP (cysteine-rich-peptide) category.GASA/GAST-peptide activity.GASA-precursor polypeptide | HID58_050672 | A0ABQ8A6U5 | UncharacterizedproteinOS=BrassicanapusOX=3708GN=HID58_050672PE=4SV=1 |
| Phytohormone action.signalling peptides.CRP (cysteine-rich-peptide) category.RALF/RALFL-peptide activity.RALF/RALFL-precursor polypeptide | BnaAnng39730D | A0A078K063 | ProteinRALF-like34,(rape)hypotheticalproteinOS=BrassicanapusOX=3708GN=BnaAnng39730DPE=3SV=1 |
| Plant organogenesis.flower formation.autonomous floral-promotion pathway.component of histone deacetylase complex *(FVE) | HID58_032611 | A0ABQ8BWW7 | Histone-bindingproteinRBBP4-likeN-terminaldomain-containingproteinOS=BrassicanapusOX=3708GN=HID58_032611PE=4SV=1 |
| Plant organogenesis.flower formation.floral transition regulation.FRI-C transcription effector complex.stabilizing component *(FRL1/2) | DARMORV10_C09P60740.1 | A0A816J6W7 | ConservedoligomericGolgicomplexsubunit1OS=BrassicanapusOX=3708GN=DARMORV10_C09P60740.1PE=3SV=1 |
| Plant organogenesis.vascular system formation.phloem differentiation.sieve element formation.phloem filament.regulatory protein *(SEOR1/2/3) | HID58_020561 | A0ABQ8CVC4 | Sieve element occlusion N-terminal domain-containing protein OS=Brassica napus OX=3708 GN=HID58_020561 PE=4 SV=1 |
| Plant reproduction.gametogenesis.female gametophyt.germ cell differentiation factor *(RKD) | DARMORV10_C09P11330.1 | A0A816J0G2 | Zeaxanthinepoxidase,chloroplasticOS=BrassicanapusOX=3708GN=DARMORV10_C09P11330.1PE=4SV=1 |
| Plant reproduction.gametogenesis.male gametophyt.regulatory protein *(DUO3) | HID58_032830 | A0ABQ8BXP3 | ABC transporter domain-containing protein OS=Brassica napus OX=3708 GN=HID58_032830 PE=4 SV=1 |
| Plant reproduction.pollination and fertilization.gamete viability factor *(DEM1/2) | HID58_020209 | A0ABQ8DGR3 | Vacuolar import/degradation Vid27 C-terminal domain-containing protein OS=Brassica napus OX=3708 GN=HID58_020209 PE=4 SV=1 |
| Protein biosynthesis.aminoacyl-tRNA formation.isoleucine-tRNA ligase | HID58_077996 | A0ABQ7YT00 | Isoleucine--tRNA ligase protein (Fragment) OS=Brassica napus OX=3708 GN=HID58_077996 PE=4 SV=1 |
| Protein biosynthesis.aminoacyl-tRNA formation.lysine-tRNA ligase | HID58_011223 | A0ABQ8DXN5 | Lysine--tRNA ligase protein OS=Brassica napus OX=3708 GN=HID58_011223 PE=4 SV=1 |
| Protein biosynthesis.aminoacyl-tRNA formation.phenylalanine-tRNA ligase heterodimer.subunit alpha *(PheRSa) | DARMORV10_C08P41200.1 | A0A816UX43 | Phenylalanine-tRNAligaseOS=BrassicanapusOX=3708GN=DARMORV10_C08P41200.1PE=3SV=1 |
| Protein biosynthesis.aminoacyl-tRNA formation.tryptophan-tRNA ligase | HID58_010945 | A0ABQ8DX70 | Tryptophan--tRNAligase,cytoplasmicprotein(Fragment)OS=BrassicanapusOX=3708GN=HID58_010945PE=4SV=1 |
| Protein biosynthesis.aminoacyl-tRNA formation.valine-tRNA ligase | DARMORV10_A08P27330.1 | A0A817A5K5 | Valine-tRNAligaseOS=BrassicanapusOX=3708GN=DARMORV10_A08P27330.1PE=3SV=1 |
| Protein biosynthesis.organellar translation machinery.aminoacyl-tRNA formation.glycine-tRNA ligase *(EDD1) | DARMORV10_C06P32790.1 | A0A816QGY5 | Glycine-tRNAligaseOS=BrassicanapusOX=3708GN=DARMORV10_C06P32790.1PE=3SV=1 |
| Protein biosynthesis.organellar translation machinery.aminoacyl-tRNA formation.methionine-tRNA ligase *(OVA1) | HID58_014229 | A0ABQ8DGQ0 | Methionine--tRNAligaseproteinOS=BrassicanapusOX=3708GN=HID58_014229PE=4SV=1 |
| Protein biosynthesis.organellar translation machinery.aminoacyl-tRNA formation.serine-tRNA ligase *(OVA7) | HID58_083608 | A0ABQ7YFN9 | Serine--tRNAligaseproteinOS=BrassicanapusOX=3708GN=HID58_083608PE=4SV=1 |
| Protein biosynthesis.organellar translation machinery.mitochondrial ribosome biogenesis.large ribosomal subunit proteome.component *(mL105) | HID58_035136 | A0ABQ8C5S8 | Thioredoxin domain-containing protein OS=Brassica napus OX=3708 GN=HID58_035136 PE=4 SV=1 |
| Protein biosynthesis.organellar translation machinery.mitochondrial ribosome biogenesis.large ribosomal subunit proteome.component *(uL29m) | HID58_064260 | A0ABQ7Z9G2 | Sas10C-terminaldomain-containingproteinOS=BrassicanapusOX=3708GN=HID58_064260PE=4SV=1 |
| Protein biosynthesis.organellar translation machinery.mitochondrial ribosome biogenesis.large ribosomal subunit proteome.component *(uL4m) | DARMORV10_A09P59050.1 | A0A816PL51 | LargeribosomalsubunitproteinuL4mOS=BrassicanapusOX=3708GN=DARMORV10_A09P59050.1PE=3SV=1 |
| Protein biosynthesis.organellar translation machinery.mitochondrial ribosome biogenesis.large ribosomal subunit proteome.component *(uL4m) | HID58_073862 | A0ABQ7YF46 | LargeribosomalsubunitproteinuL4mOS=BrassicanapusOX=3708GN=HID58_073862PE=4SV=1 |
| Protein biosynthesis.organellar translation machinery.mitochondrial ribosome biogenesis.mitochondrial tRNA import system.import factor *(TRIC1/2) | DARMORV10_C08P24540.1 | A0A816V877 | ChloroplasticimportinnermembranetranslocasesubunitHP30-1,(rape)hypotheticalproteinOS=BrassicanapusOX=3708GN=DARMORV10_C08P24540.1PE=3SV=1 |
| Protein biosynthesis.organellar translation machinery.mitochondrial ribosome biogenesis.small ribosomal subunit proteome.component *(bS16m) | HID58_040307 | A0ABQ8B7M0 | UncharacterizedproteinOS=BrassicanapusOX=3708GN=HID58_040307PE=4SV=1 |
| Protein biosynthesis.organellar translation machinery.plastidial ribosome biogenesis.large ribosomal subunit proteome | HID58_072661 | A0ABQ7Z517 | 50SribosomalproteinL22,chloroplasticproteinOS=BrassicanapusOX=3708GN=HID58_072661PE=4SV=1 |
| Protein biosynthesis.organellar translation machinery.plastidial ribosome biogenesis.large ribosomal subunit proteome.component *(uL13c) | HID58_073559 | A0ABQ7Z7L6 | Folate gamma-glutamyl hydrolase protein (Fragment) OS=Brassica napus OX=3708 GN=HID58_073559 PE=4 SV=1 |
| Protein biosynthesis.organellar translation machinery.plastidial ribosome biogenesis.large ribosomal subunit proteome.component *(uL23c) | rpl23 | A0A1B1XZB1 | 50SribosomalproteinL23,chloroplasticOS=Brassicanapusvar.napusOX=138011GN=rpl23PE=3SV=1 |
| Protein biosynthesis.organellar translation machinery.plastidial ribosome biogenesis.large ribosomal subunit proteome.component *(uL4c) | BnaC05g05110D | A0A078FDV7 | LargeribosomalsubunitproteinuL4cOS=BrassicanapusOX=3708GN=BnaC05g05110DPE=3SV=1 |
| Protein biosynthesis.organellar translation machinery.plastidial ribosome biogenesis.plastidial ribosome assembly.ribosome biogenesis factor *(CGL20) | BnaA07g02500D | A0A078GCW3 | HistonechaperoneASF1-like,(rape)hypotheticalproteinOS=BrassicanapusOX=3708GN=BnaA07g02500DPE=4SV=1 |
| Protein biosynthesis.organellar translation machinery.plastidial ribosome biogenesis.plastidial ribosome assembly.ribosome biogenesis factor *(CRASS) | HID58_050672 | A0ABQ8A6U5 | UncharacterizedproteinOS=BrassicanapusOX=3708GN=HID58_050672PE=4SV=1 |
| Protein biosynthesis.organellar translation machinery.plastidial ribosome biogenesis.small ribosomal subunit proteome.component *(bS18c) | rps18 | A0A1B1XZQ3 | SmallribosomalsubunitproteinbS18cOS=Brassicanapusvar.napusOX=138011GN=rps18PE=3SV=1 |
| Protein biosynthesis.organellar translation machinery.plastidial ribosome biogenesis.small ribosomal subunit proteome.component *(cS23) | HID58_072397 | A0ABQ7Z4J3 | 30S ribosomal protein 3, chloroplastic protein (Fragment) OS=Brassica napus OX=3708 GN=HID58_072397 |
| Protein biosynthesis.ribosome biogenesis.large ribosomal subunit (LSU).LSU processome.pre-60S ribosomal subunit nuclear assembly.OPNR assembly complex.ATP-dependent chaperone component *(CDC48d) | HID58_023971 | A0ABQ8D3M2 | Glutamine amidotransferase type-2 domain-containing protein OS=Brassica napus OX=3708 GN=HID58_023971 PE=4 SV=1 |
| Protein biosynthesis.ribosome biogenesis.large ribosomal subunit (LSU).LSU proteome.component *(eL14) | HID58_074050 | A0ABQ7YIA5 | Uncharacterizedprotein(Fragment)OS=BrassicanapusOX=3708GN=HID58_074050PE=4SV=1 |
| Protein biosynthesis.ribosome biogenesis.large ribosomal subunit (LSU).LSU proteome.component *(eL28) | BnaA09g44030D | A0A078G9F7 | 60SribosomalproteinL28-1,BnaA09g44030DproteinOS=BrassicanapusOX=3708GN=BnaA09g44030DPE=3SV=1 |
| Protein biosynthesis.ribosome biogenesis.large ribosomal subunit (LSU).LSU proteome.component *(eL33) | HID58_073223 | A0ABQ7Z6R8 | PhosphoribosylformylglycinamidinesynthaseOS=BrassicanapusOX=3708GN=HID58_073223PE=4SV=1 |
| Protein biosynthesis.ribosome biogenesis.large ribosomal subunit (LSU).LSU proteome.component *(eL43) | BnaA09g38330D | A0A078HTJ8 | BnaA09g38330DproteinOS=BrassicanapusOX=3708GN=BnaA09g38330DPE=3SV=1 |
| Protein biosynthesis.ribosome biogenesis.large ribosomal subunit (LSU).LSU proteome.component *(RPP1) | HID58_031542 | A0ABQ8BTQ9 | 60SacidicribosomalproteinP1(Fragment)OS=BrassicanapusOX=3708GN=HID58_031542PE=4SV=1 |
| Protein biosynthesis.ribosome biogenesis.small ribosomal subunit (SSU).SSU processome.SSU processome assembly.assembly factor *(LCP5) | HID58_064260 | A0ABQ7Z9G2 | Sas10C-terminaldomain-containingproteinOS=BrassicanapusOX=3708GN=HID58_064260PE=4SV=1 |
| Protein biosynthesis.ribosome biogenesis.small ribosomal subunit (SSU).SSU proteome.component *(eS1) | HID58_029707 | A0ABQ8CG13 | SmallribosomalsubunitproteineS1OS=BrassicanapusOX=3708GN=HID58_029707PE=4SV=1 |
| Protein biosynthesis.ribosome biogenesis.small ribosomal subunit (SSU).SSU proteome.component *(eS10) | BnaA08g30820D | A0A078JHM6 | Plectin/eS10N-terminaldomain-containingprotein(rape)hypotheticalproteinOS=BrassicanapusOX=3708GN=BnaA08g30820DPE=3SV=1 |
| Protein biosynthesis.ribosome biogenesis.small ribosomal subunit (SSU).SSU proteome.component *(eS19) | HID58_007684 | A0ABQ8EHJ5 | 40S ribosomal protein S19 OS=Brassica napus OX=3708 GN=HID58_007684 PE=4 SV=1 |
| Protein biosynthesis.ribosome biogenesis.small ribosomal subunit (SSU).SSU proteome.component *(eS19) | HID58_053239 | A0ABQ8AE57 | 40SribosomalproteinS19OS=BrassicanapusOX=3708GN=HID58_053239PE=4SV=1 |
| Protein biosynthesis.ribosome biogenesis.small ribosomal subunit (SSU).SSU proteome.component *(eS6) | HID58_057241 | A0ABQ8AQJ3 | 40SribosomalproteinS6(Fragment)OS=BrassicanapusOX=3708GN=HID58_057241PE=4SV=1 |
| Protein biosynthesis.ribosome biogenesis.small ribosomal subunit (SSU).SSU proteome.component *(RACK1) | DARMORV10_C06P03270.1 | A0A816Q204 | ReceptorforactivatedCkinase1B,(rape)hypotheticalproteinOS=BrassicanapusOX=3708GN=DARMORV10_C06P03270.1PE=3SV=1 |
| Protein biosynthesis.ribosome biogenesis.small ribosomal subunit (SSU).SSU proteome.component *(uS5) | BnaC01g28170D | A0A078I7M4 | UniversalribosomalproteinuS5family,(rape)hypotheticalproteinOS=BrassicanapusOX=3708GN=BnaC01g28170DPE=3SV=1 |
| Protein biosynthesis.translation elongation.eEF5 poly-P/G elongation factor activity.poly-P/G elongation factor *(eEF5/eIF5A) |  | #N/D | #N/D |
| Protein biosynthesis.translation elongation.eEF5 poly-P/G elongation factor activity.regulatory eEF5 hypusination.deoxyhypusine hydroxylase | HID58_082509 | A0ABQ7YAR3 | Deoxyhypusinehydroxylaseprotein(Fragment)OS=BrassicanapusOX=3708GN=HID58_082509PE=4SV=1 |
| Protein biosynthesis.translation elongation.eEF5 poly-P/G elongation factor activity.regulatory eEF5 hypusination.deoxyhypusine synthase | HID58_004058 | A0ABQ8E4Q1 | FolylpolyglutamatesynthaseproteinOS=BrassicanapusOX=3708GN=HID58_004058PE=4SV=1 |
| Protein biosynthesis.translation initiation.eIF2A-eIF5B initiator tRNA carrier complex.component *(eIF2A) | HID58_027010 | A0ABQ8CQM1 | UncharacterizedproteinOS=BrassicanapusOX=3708GN=HID58_027010PE=4SV=1 |
| Protein biosynthesis.translation initiation.mRNA loading.mRNA unwinding factor *(eIF4A) | HID58_027685 | A0ABQ8CSG4 | RNAhelicaseproteinOS=BrassicanapusOX=3708GN=HID58_027685PE=4SV=1 |
| Protein biosynthesis.translation initiation.mRNA loading.mRNA unwinding factor *(eIF4A) | HID58_057592 | A0ABQ8ASE2 | RNAhelicaseproteinOS=BrassicanapusOX=3708GN=HID58_057592PE=4SV=1 |
| Protein biosynthesis.translation initiation.mRNA loading.RNA-cap-binding factor *(4EHP) | DARMORV10_C03P10090.1 | A0A816I526 | EukaryotictranslationinitiationfactorNCBPOS=BrassicanapusOX=3708GN=DARMORV10_C03P10090.1PE=3SV=1 |
| Protein biosynthesis.translation initiation.Pre-Initiation Complex (PIC) module.eIF1 PIC assembly factor activity.assembly factor *(eIF1) | HID58_018161 | A0ABQ8D944 | SUI1 domain-containing protein (Fragment) OS=Brassica napus OX=3708 GN=HID58_018161 PE=4 SV=1 |
| Protein biosynthesis.translation initiation.Pre-Initiation Complex (PIC) module.eIF2 Met-tRNA binding factor activity.eIF2 Met-tRNA binding factor complex.eIF2-alpha component activity.regulatory protein *(GCN20) of eIF2-alpha kinase activity | HID58_032830 | A0ABQ8BXP3 | ABC transporter domain-containing protein OS=Brassica napus OX=3708 GN=HID58_032830 PE=4 SV=1 |
| Protein biosynthesis.translation initiation.Pre-Initiation Complex (PIC) module.eIF2 Met-tRNA binding factor activity.regulatory factor *(eIF5C) | HID58_005419 | A0ABQ8EAY0 | W2 domain-containing protein OS=Brassica napus OX=3708 GN=HID58_005419 PE=4 SV=1 |
| Protein biosynthesis.translation initiation.Pre-Initiation Complex (PIC) module.eIF2 Met-tRNA binding factor activity.regulatory factor *(eIF5C) | BnaC06g42190D | A0A078IJ78 | W2domain-containingprotein,BnaC06g42190DproteinOS=BrassicanapusOX=3708GN=BnaC06g42190DPE=3SV=1 |
| Protein biosynthesis.translation initiation.Pre-Initiation Complex (PIC) module.eIF3 mRNA-to-PIC binding complex.component *(eIF3d) | BnaA09g17080D | A0A078FJF5 | Eukaryotictranslationinitiationfactor3subunitDOS=BrassicanapusOX=3708GN=BnaA09g17080DPE=3SV=1 |
| Protein biosynthesis.translation initiation.Pre-Initiation Complex (PIC) module.eIF3 mRNA-to-PIC binding complex.component *(eIF3e) | BnaC04g24310D | A0A078H2A9 | Eukaryotictranslationinitiationfactor3subunitEOS=BrassicanapusOX=3708GN=BnaC04g24310DPE=3SV=1 |
| Protein biosynthesis.translation initiation.Pre-Initiation Complex (PIC) module.eIF3 mRNA-to-PIC binding complex.component *(eIF3j) | DARMORV10_C04P49430.1 | A0A816JT56 | Eukaryotictranslationinitiationfactor3subunitJOS=BrassicanapusOX=3708GN=DARMORV10_C04P49430.1PE=3SV=1 |
| Protein homeostasis.protein repair.protein-L-isoaspartate methyltransferase *(PIMT1/2) | DARMORV10_C03P66480.1 | A0A816II30 | Protein-L-isoaspartateO-methyltransferase(Fragment)OS=BrassicanapusOX=3708GN=DARMORV10_C03P66480.1PE=3SV=1 |
| Protein homeostasis.protein storage.seed storage protein activities.11S globulin family.Globulin-type seed storage protein *(CR) | HID58_036973 | A0ABQ8C9A8 | 11S globulin protein OS=Brassica napus OX=3708 GN=HID58_036973 PE=4 SV=1 |
| Protein homeostasis.protein storage.seed storage protein activities.11S globulin family.Globulin-type seed storage protein *(CR) | DARMORV10_A01P09420.1 | A0A816XM00 | 11SglobulinOS=BrassicanapusOX=3708GN=DARMORV10_A01P09420.1PE=3SV=1 |
| Protein homeostasis.proteolysis | DARMORV10_A06P03180.1 | A0A816S5P1 | ProlylendopeptidaseOS=BrassicanapusOX=3708GN=DARMORV10_A06P03180.1PE=3SV=1 |
| Protein homeostasis.proteolysis.aspartic peptidase activities.Pepsin-type protease | HID58_059908 | A0ABQ7ZU83 | Peptidase A1 domain-containing protein OS=Brassica napus OX=3708 GN=HID58_059908 PE=4 SV=1 |
| Protein homeostasis.proteolysis.aspartic peptidase activities.Pepsin-type protease | BnaA03g55560D | A0A078J712 | PeptidaseA1family,(rape)hypotheticalproteinOS=BrassicanapusOX=3708GN=BnaA03g55560DPE=3SV=1 |
| Protein homeostasis.proteolysis.cysteine peptidase activities.Legumain-type asparaginyl endopeptidase *(AEP) | HID58_040444 | A0ABQ8B833 | LegumainproteinOS=BrassicanapusOX=3708GN=HID58_040444PE=4SV=1 |
| Protein homeostasis.proteolysis.cysteine peptidase activities.Papain-type protease activities.subclass CTB cysteine protease | DARMORV10_C04P03330.1 | A0A816JCK2 | CathepsinB-likeprotease2,(rape)hypotheticalproteinOS=BrassicanapusOX=3708GN=DARMORV10_C04P03330.1PE=3SV=1 |
| Protein homeostasis.proteolysis.cysteine peptidase activities.Papain-type protease activities.subclass RD19 cysteine protease | BnaA01g05320D | A0A078HTU1 | PeptidaseC1family,(rape)hypotheticalproteinOS=BrassicanapusOX=3708GN=BnaA01g05320DPE=3SV=1 |
| Protein homeostasis.proteolysis.cysteine peptidase activities.Papain-type protease activities.subclass RD21/XCP thiol protease | DARMORV10_A08P29200.1 | A0A817AI73 | CysteineproteaseXCP2,(rape)hypotheticalproteinOS=BrassicanapusOX=3708GN=DARMORV10_A08P29200.1PE=3SV=1 |
| Protein homeostasis.proteolysis.metallopeptidase activities.FtsH endopeptidase activities.FtsH plastidial protease complexes.component *(FtsH7/9) | DARMORV10_C03P71290.1 | A0A816IME9 | ATP-dependentzincmetalloproteaseFTSH7,chloroplastic(rape)hypotheticalproteinOS=BrassicanapusOX=3708GN=DARMORV10_C03P71290.1PE=3SV=1 |
| Protein homeostasis.proteolysis.metallopeptidase activities.M1-class aminopeptidase activities.broad substrate-specificity aminopeptidase *(MPA1) | HID58_096058 | A0ABQ7X1J6 | UncharacterizedproteinOS=BrassicanapusOX=3708GN=HID58_096058PE=4SV=1 |
| Protein homeostasis.proteolysis.metallopeptidase activities.M1-class aminopeptidase activities.leucyl aminopeptidase *(LKHA4) | BnaC09g43560D | A0A078F5T2 | LeucineaminopeptidaseOS=BrassicanapusOX=3708GN=BnaC09g43560DPE=3SV=1 |
| Protein homeostasis.proteolysis.metallopeptidase activities.M24-class aminopeptidase activities.aminopeptidase *(APP) | HID58_068901 | A0ABQ7ZMP2 | Xaa-Pro aminopeptidase P protein OS=Brassica napus OX=3708 GN=HID58_068901 PE=4 SV=1 |
| Protein homeostasis.proteolysis.metallopeptidase activities.M3-class metallopeptidase activities.Thimet-type metallopeptidase *(TOP1/2) | DARMORV10_A06P32460.1 | A0A816SPT8 | OligopeptidaseAOS=BrassicanapusOX=3708GN=DARMORV10_A06P32460.1PE=3SV=1 |
| Protein homeostasis.proteolysis.metallopeptidase activities.M3-class metallopeptidase activities.Thimet-type metalloprotease *(PSPM3) | HID58_072465 | A0ABQ7Z4J6 | Peptidase M3A/M3B catalytic domain-containing protein (Fragment) OS=Brassica napus OX=3708 GN=HID58_072465 PE=4 SV=1 |
| Protein homeostasis.proteolysis.protease inhibitor activities.Kunitz protease inhibitor | HID58_027706 | A0ABQ8CUV5 | Uncharacterizedprotein(Fragment)OS=BrassicanapusOX=3708GN=HID58_027706PE=4SV=1 |
| Protein homeostasis.proteolysis.serine peptidase activities.chloroplast Clp-type protease complex.chaperone component *(ClpC1/2) | DARMORV10_C08P23840.1 | A0A816UPL9 | ChaperoneproteinClpC2,chloroplastic,(rape)hypotheticalproteinOS=BrassicanapusOX=3708GN=DARMORV10_C08P23840.1PE=3SV=1 |
| Protein homeostasis.proteolysis.serine peptidase activities.chloroplast Clp-type protease complex.ClpP proteolytic core activities.proteolytic component *(ClpP3) | BnaC06g27560D | A0A078HEJ2 | ATP-dependentClpproteaseproteolyticsubunitOS=BrassicanapusOX=3708GN=BnaC06g27560DPE=3SV=1 |
| Protein homeostasis.proteolysis.serine peptidase activities.chloroplast Clp-type protease complex.plant-specific stabilizing component *(ClpT1/2) | DARMORV10_A03P55940.1 | A0A816WCD4 | ATP-dependentClpproteaseATP-bindingsubunitCLPT1,chloroplastic-like,(rape)hypotheticalprotein(Fragment)OS=BrassicanapusOX=3708GN=DARMORV10_A03P55940.1PE=4SV=1 |
| Protein homeostasis.proteolysis.serine peptidase activities.mitochondrion Clp-type protease complex.proteolytic component *(ClpP2) | HID58_077446 | A0ABQ7YQD0 | ATP-dependent Clp protease proteolytic subunit protein OS=Brassica napus OX=3708 GN=HID58_077446 PE=4 SV=1 |
| Protein homeostasis.proteolysis.serine peptidase activities.SBT-type protease activities.protease *(SBT1) | DARMORV10_C09P41180.1 | A0A816JAL7 | Subtilisin-likeproteaseSBT1.3,(rape)hypotheticalproteinOS=BrassicanapusOX=3708GN=DARMORV10_C09P41180.1PE=3SV=1 |
| Protein homeostasis.proteolysis.serine peptidase activities.SBT-type protease activities.protease *(SBT5) | BnaA02g06800D | A0A078JDI8 | PeptidaseS8family,BnaA02g06800DproteinOS=BrassicanapusOX=3708GN=BnaA02g06800DPE=3SV=1 |
| Protein homeostasis.proteolysis.serine peptidase activities.SCPL-type carboxypeptidase activities.carboxypeptidase | HID58_054762 | A0ABQ8AIR4 | UncharacterizedproteinOS=BrassicanapusOX=3708GN=HID58_054762PE=4SV=1 |
| Protein homeostasis.proteolysis.serine peptidase activities.SCPL-type carboxypeptidase activities.carboxypeptidase *(SCPL) | HID58_040664 | A0ABQ8BA80 | Carboxypeptidase protein OS=Brassica napus OX=3708 GN=HID58_040664 PE=4 SV=1 |
| Protein homeostasis.proteolysis.serine peptidase activities.SCPL-type carboxypeptidase activities.carboxypeptidase *(SCPL) | HID58_017738 | A0ABQ8D7Z8 | Carboxypeptidaseprotein(Fragment)OS=BrassicanapusOX=3708GN=HID58_017738PE=4SV=1 |
| Protein homeostasis.ubiquitin-proteasome system.26S proteasome.19S regulatory particle.base subcomplex.regulatory component *(RPN13) | HID58_010389 | A0ABQ8DV52 | Regulatoryparticlenon-ATPase13protein(Fragment)OS=BrassicanapusOX=3708GN=HID58_010389PE=4SV=1 |
| Protein homeostasis.ubiquitin-proteasome system.26S proteasome.19S regulatory particle.base subcomplex.regulatory component *(RPT2) | HID58_080739 | A0ABQ7Y5R3 | AAA+ ATPase domain-containing protein OS=Brassica napus OX=3708 GN=HID58_080739 PE=4 SV=1 |
| Protein homeostasis.ubiquitin-proteasome system.26S proteasome.19S regulatory particle.lid subcomplex.regulatory component *(RPN9) | BnaAnng05690D | A0A078HRQ0 | 26Sproteasomenon-ATPaseregulatorysubunit13homologB-like,(rape)hypotheticalproteinOS=BrassicanapusOX=3708GN=BnaAnng05690DPE=3SV=1 |
| Protein homeostasis.ubiquitin-proteasome system.26S proteasome.20S core particle.alpha-type components.alpha-type-3 component *(PAC) | DARMORV10_C03P52170.1 | A0A816IA79 | Proteasomesubunitalphatype-4-A-like,(rape)hypotheticalproteinOS=BrassicanapusOX=3708GN=DARMORV10_C03P52170.1PE=3SV=1 |
| Protein homeostasis.ubiquitin-proteasome system.26S proteasome.20S core particle.assembly.assembly factor *(PA200) | HID58_053739 | A0ABQ8AH24 | Proteasome activator subunit 4 OS=Brassica napus OX=3708 GN=HID58_053739 PE=4 SV=1 |
| Protein homeostasis.ubiquitin-proteasome system.26S proteasome.20S core particle.beta-type components.beta-type-3 component *(PBC) | DARMORV10_C02P34590.1 | A0A816KKL8 | Proteasomesubunitbeta(Fragment)OS=BrassicanapusOX=3708GN=DARMORV10_C02P34590.1PE=3SV=1 |
| Protein homeostasis.ubiquitin-proteasome system.26S proteasome.20S core particle.beta-type components.beta-type-3 component *(PBC) | HID58_006114 | A0ABQ8EAH0 | ProteasomesubunitbetaproteinOS=BrassicanapusOX=3708GN=HID58_006114PE=4SV=1 |
| Protein homeostasis.ubiquitin-proteasome system.26S proteasome.20S core particle.beta-type components.beta-type-5 component *(PBE) | HID58_036596 | A0ABQ8C8C1 | Proteasome endopeptidase complex OS=Brassica napus OX=3708 GN=HID58_036596 PE=4 SV=1 |
| Protein homeostasis.ubiquitin-proteasome system.membrane-associated protein degradation.chloroplast/mitochondrion-associated protein degradation.substrate-to-CDC48 bridging factor *(PUX10) | DARMORV10_A03P29290.1 | A0A816WFF6 | PlantUBXdomain-containingprotein10-like,(rape)hypotheticalproteinOS=BrassicanapusOX=3708GN=DARMORV10_A03P29290.1PE=4SV=1 |
| Protein homeostasis.ubiquitin-proteasome system.membrane-associated protein degradation.ER-associated protein degradation (ERAD).substrate processing.deglycosylating peptide:N-glycanase *(PNG1) | HID58_077098 | A0ABQ7YPG5 | Peptide-N(4)-(N-acetyl-beta-glucosaminyl)asparagineamidaseOS=BrassicanapusOX=3708GN=HID58_077098PE=4SV=1 |
| Protein homeostasis.ubiquitin-proteasome system.membrane-associated protein degradation.ER-associated protein degradation (ERAD).substrate processing.ubiquitin-binding adaptor *(UFD3) | HID58_092025 | A0ABQ7X000 | PhospholipaseA-2-activatingproteinOS=BrassicanapusOX=3708GN=HID58_092025PE=4SV=1 |
| Protein homeostasis.ubiquitin-proteasome system.ubiquitin-fold protein conjugation.CULLIN-based E3 ubiquitin ligase activities.co-factor of RBX1 activity *(DCN1) | BnaA05g26410D | A0A078F1R5 | DefectiveincullinneddylationproteinOS=BrassicanapusOX=3708GN=BnaA05g26410DPE=4SV=1 |
| Protein homeostasis.ubiquitin-proteasome system.ubiquitin-fold protein conjugation.CULLIN-based E3 ubiquitin ligase activities.CUL1-based/SCF E3 ubiquitin ligase complexes.linker component *(ASK1/2/3) | HID58_007992 | A0ABQ7XHK5 | SKP1-likeproteinOS=BrassicanapusOX=3708GN=HID58_007992PE=4SV=1 |
| Protein homeostasis.ubiquitin-proteasome system.ubiquitin-fold protein conjugation.CULLIN-based E3 ubiquitin ligase activities.CUL1-based/SCF E3 ubiquitin ligase complexes.regulatory protein *(CAND1) | DARMORV10_C07P33270.1 | A0A816MTW1 | Cullin-associatedNEDD8-dissociatedprotein1OS=BrassicanapusOX=3708GN=DARMORV10_C07P33270.1PE=3SV=1 |
| Protein homeostasis.ubiquitin-proteasome system.ubiquitin-fold protein conjugation.CULLIN-based E3 ubiquitin ligase activities.CUL4-based E3 ubiquitin ligase complexes.DCAF substrate adaptor component activities.COP1-SPA substrate adaptor module.regulator component *(SPA) | HID58_070590 | A0ABQ7YZ90 | Proteinkinasedomain-containingproteinOS=BrassicanapusOX=3708GN=HID58_070590PE=4SV=1 |
| Protein homeostasis.ubiquitin-proteasome system.ubiquitin-fold protein conjugation.CULLIN-based E3 ubiquitin ligase activities.CUL4-based E3 ubiquitin ligase complexes.DCAF substrate adaptor component activities.substrate adaptor | HID58_030056 | A0ABQ8CGW5 | DEK-Cdomain-containingproteinOS=BrassicanapusOX=3708GN=HID58_030056PE=4SV=1 |
| Protein homeostasis.ubiquitin-proteasome system.ubiquitin-fold protein conjugation.CULLIN-based E3 ubiquitin ligase activities.CUL4-based E3 ubiquitin ligase complexes.DCAF substrate adaptor component activities.substrate adaptor *(DCAF1) | BnaC04g11150D | A0A078G1Y5 | Pre-mRNA-processingfactor19OS=BrassicanapusOX=3708GN=BnaC04g11150DPE=3SV=1 |
| Protein homeostasis.ubiquitin-proteasome system.ubiquitin-fold protein conjugation.CULLIN-based E3 ubiquitin ligase activities.CUL4-based E3 ubiquitin ligase complexes.DCAF substrate adaptor component activities.substrate adaptor *(DWA2) | DARMORV10_C06P50230.1 | A0A816QSX1 | WDrepeat-containingproteinDWA2,(rape)hypotheticalproteinOS=BrassicanapusOX=3708GN=DARMORV10_C06P50230.1PE=4SV=1 |
| Protein homeostasis.ubiquitin-proteasome system.ubiquitin-fold protein conjugation.SUMO conjugation (sumoylation).SUMO ubiquitin-fold protein | DARMORV10_C09P46830.1 | A0A816JD15 | Ubiquitin-like domain-containing protein OS=Brassica napus OX=3708 GN=DARMORV10_C09P46830.1 PE=4 |
| Protein homeostasis.ubiquitin-proteasome system.ubiquitin-fold protein conjugation.U-Box E3 ubiquitin ligase activities.group-II.E3 ubiquitin ligase *(PUB12/13) | DARMORV10_C01P33340.1 | A0A816RLF7 | RING-typeE3ubiquitintransferaseOS=BrassicanapusOX=3708GN=DARMORV10_C01P33340.1PE=4SV=1 |
| Protein homeostasis.ubiquitin-proteasome system.ubiquitin-fold protein conjugation.U-Box E3 ubiquitin ligase activities.group-VI.E3 ubiquitin protein ligase *(PUB60) | BnaC04g11150D | A0A078G1Y5 | Pre-mRNA-processingfactor19OS=BrassicanapusOX=3708GN=BnaC04g11150DPE=3SV=1 |
| Protein homeostasis.ubiquitin-proteasome system.ubiquitin-fold protein conjugation.UBQ conjugation (ubiquitylation).E2 ubiquitin-conjugating enzyme activities.E2 ubiquitin-conjugating enzyme *(UBC1/2/3) | DARMORV10_C08P45750.1 | A0A816UW50 | PectatelyaseOS=BrassicanapusOX=3708GN=DARMORV10_C08P45750.1PE=3SV=1 |
| Protein homeostasis.ubiquitin-proteasome system.ubiquitin-fold protein conjugation.UFM conjugation.UFM ubiquitin-fold protein *(UFM1) | DARMORV10_A07P27870.1 | A0A816QFX6 | Ubiquitin-foldmodifier1OS=BrassicanapusOX=3708GN=DARMORV10_A07P27870.1PE=3SV=1 |
| Protein homeostasis.ubiquitin-proteasome system.ubiquitin-fold protein deconjugation.RUB deconjugation.COP9 signalosome complex.component *(CSN2) | DARMORV10_A03P26810.1 | A0A816WDV3 | COP9signalosomecomplexsubunit2OS=BrassicanapusOX=3708GN=DARMORV10_A03P26810.1PE=3SV=1 |
| Protein homeostasis.ubiquitin-proteasome system.ubiquitin-fold protein deconjugation.RUB deconjugation.COP9 signalosome complex.component *(CSN3) | HID58_089144 | A0ABQ7XY62 | COP9signalosomecomplexsubunit3(Fragment)OS=BrassicanapusOX=3708GN=HID58_089144PE=4SV=1 |
| Protein homeostasis.ubiquitin-proteasome system.ubiquitin-fold protein deconjugation.RUB deconjugation.COP9 signalosome complex.component *(CSN5) | BnaA09g30270D | A0A078G4L8 | COP9signalosomecomplexsubunit5°,BnaA09g30270DproteinOS=BrassicanapusOX=3708GN=BnaA09g30270DPE=3SV=1 |
| Protein homeostasis.ubiquitin-proteasome system.ubiquitin-fold protein deconjugation.UBQ deconjugation.OTU deubiquitinase activities.deubiquitinase *(OTU6-12) | HID58_050500 | A0ABQ8A6D7 | OTU domain-containing protein OS=Brassica napus OX=3708 GN=HID58_050500 PE=4 SV=1 |
| Protein homeostasis.ubiquitin-proteasome system.ubiquitin-fold protein deconjugation.UBQ deconjugation.UBP deubiquitinase activities.deubiquitinase *(UBP12-13) | HID58_003515 | A0ABQ8EQY2 | Ubiquitinyl hydrolase 1 protein OS=Brassica napus OX=3708 GN=HID58_003515 PE=4 SV=1 |
| Protein homeostasis.ubiquitin-proteasome system.ubiquitin-fold protein deconjugation.UBQ deconjugation.UBP deubiquitinase activities.deubiquitinase *(UBP12-13) | HID58_004075 | A0ABQ8E4T5 | Ubiquitinylhydrolase1proteinOS=BrassicanapusOX=3708GN=HID58_004075PE=4SV=1 |
| Protein modification.acetylation.bifunctional lysine and N-terminal acetyltransferase activities.bifunctional lysine and N-terminal acetyltransferase *(GNAT2) | DARMORV10_A05P25290.1 | A0A816TP41 | HistoneacetyltransferaseOS=BrassicanapusOX=3708GN=DARMORV10_A05P25290.1PE=3SV=1 |
| Protein modification.acetylation.bifunctional lysine and N-terminal acetyltransferase activities.bifunctional lysine and N-terminal acetyltransferase *(GNAT3) | HID58_041197 | A0ABQ8BA54 | N-acetyltransferasedomain-containingproteinOS=BrassicanapusOX=3708GN=HID58_041197PE=4SV=1 |
| Protein modification.acetylation.NatA N-terminal acetylase complex.auxiliary component *(NAA15) | HID58_006230 | A0ABQ8EAX0 | BRCTdomain-containingproteinOS=BrassicanapusOX=3708GN=HID58_006230PE=4SV=1 |
| Protein modification.cysteine disulfide formation.endoplasmic reticulum.protein disulfide isomerase *(PDI-M) | HID58_051828 | A0ABQ8AA27 | Protein disulfide-isomerase OS=Brassica napus OX=3708 GN=HID58_051828 PE=4 SV=1 |
| Protein modification.dephosphorylation.protein serine/threonine protein phosphatase activities.PPM Mn/Mg-dependent protein phosphatase activities.clade E protein phosphatase | HID58_094631 | A0ABQ7X6P1 | Protein kinase domain-containing protein OS=Brassica napus OX=3708 GN=HID58_094631 PE=4 SV=1 |
| Protein modification.dephosphorylation.protein serine/threonine protein phosphatase activities.PPM Mn/Mg-dependent protein phosphatase activities.clade F protein phosphatase | HID58_049629 | A0ABQ8B5I2 | Protein-serine/threonine phosphatase OS=Brassica napus OX=3708 GN=HID58_049629 PE=4 SV=1 |
| Protein modification.dephosphorylation.protein serine/threonine protein phosphatase activities.PPP Fe/Zn-dependent protein phosphatase activities.PP1-class protein phosphatase activity.inhibitor protein | DARMORV10_C07P54390.1 | A0A816N0S2 | DNAmismatchrepairproteinMSH3OS=BrassicanapusOX=3708GN=DARMORV10_C07P54390.1PE=3SV=1 |
| Protein modification.dephosphorylation.protein serine/threonine protein phosphatase activities.PPP Fe/Zn-dependent protein phosphatase activities.PP1-class protein phosphatase activity.PP1-class protein phosphatase complex.catalytic component | HID58_058583 | A0ABQ7ZR20 | Serine/threonine-proteinphosphatase(Fragment)OS=BrassicanapusOX=3708GN=HID58_058583PE=4SV=1 |
| Protein modification.dephosphorylation.protein serine/threonine protein phosphatase activities.PPP Fe/Zn-dependent protein phosphatase activities.PP2A-class protein phosphatase complexes.catalytic component C | BnaA05g34330D | A0A078IYJ1 | Serine/threonine-proteinphosphataseOS=BrassicanapusOX=3708GN=BnaA05g34330DPE=3SV=1 |
| Protein modification.dephosphorylation.protein serine/threonine protein phosphatase activities.PPP Fe/Zn-dependent protein phosphatase activities.PP2A-class protein phosphatase complexes.regulatory component B2 | HID58_050878 | A0ABQ8A7D1 | EF-hand domain-containing protein OS=Brassica napus OX=3708 GN=HID58_050878 PE=4 SV=1 |
| Protein modification.dephosphorylation.protein serine/threonine protein phosphatase activities.PPP Fe/Zn-dependent protein phosphatase activities.PP2A-class protein phosphatase complexes.regulatory component B2 | HID58_023588 | A0ABQ8D2H7 | EF-handdomain-containingproteinOS=BrassicanapusOX=3708GN=HID58_023588PE=4SV=1 |
| Protein modification.glycosylation.N-linked glycosylation.complex N-glycan maturation.class-II glucosidase II heterodimer.subunit alpha | DARMORV10_C09P09530.1 | A0A816IZV9 | Glucan1,3-alpha-glucosidaseOS=BrassicanapusOX=3708GN=DARMORV10_C09P09530.1PE=3SV=1 |
| Protein modification.glycosylation.O-linked glycosylation.hydroxyproline O-linked glycosylation.hydroxyproline-O-arabinosyltransferase *(HPAT) | DARMORV10_C09P63600.1 | A0A816JAU4 | HydroxyprolineO-arabinosyltransferase-likedomain-containingprotein,(rape)hypotheticalproteinOS=BrassicanapusOX=3708GN=DARMORV10_C09P63600.1PE=3SV=1 |
| Protein modification.glycosylation.O-linked glycosylation.hydroxyproline O-linked glycosylation.hydroxyproline-O-galactosyltransferase *(HPGT) | DARMORV10_A01P06180.1 | A0A816XIR4 | (rape)hypotheticalproteinOS=BrassicanapusOX=3708GN=DARMORV10_A01P06180.1PE=3SV=1 |
| Protein modification.hydroxylation.prolyl hydroxylase | BnaC03g47960D | A0A078F366 | Procollagen-proline4-dioxygenaseOS=BrassicanapusOX=3708GN=BnaC03g47960DPE=3SV=1 |
| Protein modification.lipidation.Cys-linked prenylation.type-I protein prenyltransferase heterodimer.subunit alpha | BnaA01g18430D | A0A078G901 | Proteinfarnesyltransferase/geranylgeranyltransferasetype-1subunitalphaOS=BrassicanapusOX=3708GN=BnaA01g18430DPE=3SV=1 |
| Protein modification.lipidation.glycophosphatidylinositol (GPI) anchor addition.GPI pre-assembly.phosphoethanolamine transferase-I *(PIG-N) | HID58_020315 | A0ABQ8DFA4 | GPI ethanolamine phosphate transferase 1 protein OS=Brassica napus OX=3708 GN=HID58_020315 PE=4 SV=1 |
| Protein modification.lipidation.protein S-acylation.group-A protein S-acyltransferase activities.protein S-acyltransferase *(PAT1-9) | HID58_050200 | A0ABQ8A5K1 | Coatomersubunitdelta(Fragment)OS=BrassicanapusOX=3708GN=HID58_050200PE=4SV=1 |
| Protein modification.lipidation.protein S-acylation.group-A protein S-acyltransferase activities.protein S-acyltransferase *(PAT1-9) | HID58_082407 | A0ABQ7YDB2 | Magnesium-protoporphyrin IX monomethyl ester (oxidative) cyclase protein OS=Brassica napus OX=3708 GN=HID58_082407 PE=4 SV=1 |
| Protein modification.phosphorylation.ABC1K protein kinase activities.ancestral clade.subgroup-9 protein kinase | HID58_008258 | A0ABQ8DP47 | ABC1atypicalkinase-likedomain-containingproteinOS=BrassicanapusOX=3708GN=HID58_008258PE=4SV=1 |
| Protein modification.phosphorylation.ABC1K protein kinase activities.photosynthetic-specific clade.subgroup-2 protein kinase | HID58_049629 | A0ABQ8B5I2 | Protein-serine/threonine phosphatase OS=Brassica napus OX=3708 GN=HID58_049629 PE=4 SV=1 |
| Protein modification.phosphorylation.ABC1K protein kinase activities.photosynthetic-specific clade.subgroup-5 protein kinase | HID58_008258 | A0ABQ8DP47 | ABC1atypicalkinase-likedomain-containingproteinOS=BrassicanapusOX=3708GN=HID58_008258PE=4SV=1 |
| Protein modification.phosphorylation.ABC1K protein kinase activities.photosynthetic-specific clade.subgroup-5 protein kinase | HID58_094631 | A0ABQ7X6P1 | Protein kinase domain-containing protein OS=Brassica napus OX=3708 GN=HID58_094631 PE=4 SV=1 |
| Protein modification.phosphorylation.ABC1K protein kinase activities.photosynthetic-specific clade.subgroup-5 protein kinase | HID58_049629 | A0ABQ8B5I2 | Protein-serine/threonine phosphatase OS=Brassica napus OX=3708 GN=HID58_049629 PE=4 SV=1 |
| Protein modification.phosphorylation.AGC protein kinase activities.AGC-VIIIb/plant-AGC4 protein kinase *(PHOT1/2) | BnaA02g07840D | A0A078H5I3 | Non-specificserine/threonineproteinkinaseOS=BrassicanapusOX=3708GN=BnaA02g07840DPE=3SV=1 |
| Protein modification.phosphorylation.CAMK protein kinase activities.CDPK protein kinase | DARMORV10_A07P41530.1 | A0A816ZBX6 | Non-specificserine/threonineproteinkinaseOS=BrassicanapusOX=3708GN=DARMORV10_A07P41530.1PE=3SV=1 |
| Protein modification.phosphorylation.CAMK protein kinase activities.CDPK protein kinase | DARMORV10_C01P43710.1 | A0A816RME9 | Non-specificserine/threonineproteinkinaseOS=BrassicanapusOX=3708GN=DARMORV10_C01P43710.1PE=3SV=1 |
| Protein modification.phosphorylation.CAMK protein kinase activities.CDPK protein kinase | HID58_050529 | A0ABQ8A7S2 | Non-specificserine/threonineproteinkinaseOS=BrassicanapusOX=3708GN=HID58_050529PE=4SV=1 |
| Protein modification.phosphorylation.CMGC protein kinase activities.CDK protein kinase activities.CDKA protein kinase | DARMORV10_C08P23780.1 | A0A816UHF7 | Cyclin-dependentkinaseOS=BrassicanapusOX=3708GN=DARMORV10_C08P23780.1PE=3SV=1 |
| Protein modification.phosphorylation.CMGC protein kinase activities.CK-II protein kinase heterodimer.catalytic subunit alpha *(CKA1/2/++) | BnaA09g42220D | A0A078IEW0 | CaseinkinaseIIsubunitalphaOS=BrassicanapusOX=3708GN=BnaA09g42220DPE=3SV=1 |
| Protein modification.phosphorylation.CMGC protein kinase activities.CK-II protein kinase heterodimer.regulatory subunit beta *(CKB1/2/++) | HID58_024158 | A0ABQ8D438 | CaseinkinaseIIsubunitbetaproteinOS=BrassicanapusOX=3708GN=HID58_024158PE=4SV=1 |
| Protein modification.phosphorylation.CMGC protein kinase activities.CLK/LAMMER protein kinase | HID58_041689 | A0ABQ8BBK7 | Manganese-dependentADP-ribose/CDP-alcoholdiphosphataseproteinOS=BrassicanapusOX=3708GN=HID58_041689PE=4SV=1 |
| Protein modification.phosphorylation.TKL protein kinase activities.Extensin-type protein kinase activities.protein kinase | DARMORV10_C08P34010.1 | A0A816ULY3 | HTHcro/C1-typedomain-containingprotein,(rape)hypotheticalproteinOS=BrassicanapusOX=3708GN=DARMORV10_C08P34010.1PE=3SV=1 |
| Protein modification.phosphorylation.TKL protein kinase activities.LRR-VIII protein kinase activities.LRR-VIII-1 protein kinase | HID58_094631 | A0ABQ7X6P1 | Protein kinase domain-containing protein OS=Brassica napus OX=3708 GN=HID58_094631 PE=4 SV=1 |
| Protein modification.phosphorylation.TKL protein kinase activities.RLCK-IX receptor-like protein kinase activities.RLCK-IXa receptor-like protein kinase | HID58_007238 | A0ABQ8EDM9 | Protein kinase domain-containing protein OS=Brassica napus OX=3708 GN=HID58_007238 PE=4 SV=1 |
| Protein modification.phosphorylation.TKL protein kinase activities.RLCK-V receptor-like protein kinase | HID58_004616 | A0ABQ8E6A0 | Receptor-like serine/threonine-protein kinase OS=Brassica napus OX=3708 GN=HID58_004616 PE=4 SV=1 |
| Protein modification.phosphorylation.TKL protein kinase activities.RLCK-VIII receptor-like protein kinase activities.RLCK-VIII receptor-like protein kinase | HID58_057950 | A0ABQ7ZNS7 | Protein kinase domain-containing protein protein OS=Brassica napus OX=3708 GN=HID58_057950 PE=4 SV=1 |
| Protein modification.targeting peptide maturation.endomembrane system.SPC endoplasmic signal peptidase complex.component *(SPCs2) | HID58_017286 | A0ABQ8D969 | Signal peptidase complex subunit 2 protein OS=Brassica napus OX=3708 GN=HID58_017286 PE=4 SV=1 |
| Protein modification.targeting peptide maturation.mitochondrion.MPP mitochondrial signal peptidase heterodimer.subunit beta | BnaC05g48550D | A0A078HZF7 | MitochondrialprocessingpeptidaseOS=BrassicanapusOX=3708GN=BnaC05g48550DPE=3SV=1 |
| Protein modification.targeting peptide maturation.mitochondrion.preprotein processing peptidase *(ICP55) | BnaA01g07350D | A0A078I1N1 | Xaa-ProdipeptidaseOS=BrassicanapusOX=3708GN=BnaA01g07350DPE=3SV=1 |
| Protein physical control.endoplasmic reticulum protein translocation and insertion.EMC membrane protein insertion complex.component *(EMC3) | HID58_041429 | A0ABQ8BCE5 | ER membrane protein complex subunit 3 OS=Brassica napus OX=3708 GN=HID58_041429 PE=4 SV=1 |
| Protein physical control.endoplasmic reticulum protein translocation and insertion.EMC membrane protein insertion complex.component *(EMC7) | DARMORV10_A01P06180.1 | A0A816XIR4 | (rape)hypotheticalproteinOS=BrassicanapusOX=3708GN=DARMORV10_A01P06180.1PE=3SV=1 |
| Protein physical control.endoplasmic reticulum protein translocation and insertion.EMC membrane protein insertion complex.component *(EMC7) | HID58_082880 | A0ABQ7YBT9 | ER membrane protein complex subunit 7 beta-sandwich domain-containing protein (Fragment) OS=Brassica napus OX=3708 GN=HID58_082880 PE=4 SV=1 |
| Protein physical control.endoplasmic reticulum protein translocation and insertion.GET post-translational insertion system.GET4-GET5 scaffold subcomplex.GET3-recruitment component *(GET4) | HID58_022937 | A0ABQ8D0M9 | GolgitoERtrafficprotein4homologproteinOS=BrassicanapusOX=3708GN=HID58_022937PE=4SV=1 |
| Protein physical control.endoplasmic reticulum protein translocation and insertion.multi-pass translocon.BOS scaffolding complex.component *(NCLN) | BnaC03g54000D | A0A078G743 | NicalinOS=BrassicanapusOX=3708GN=BnaC03g54000DPE=3SV=1 |
| Protein physical control.endoplasmic reticulum protein translocation and insertion.multi-pass translocon.PAT chaperone complex.component *(CCDC47) | HID58_012613 | A0ABQ8E492 | DUF1682domain-containingproteinOS=BrassicanapusOX=3708GN=HID58_012613PE=4SV=1 |
| Protein physical control.endoplasmic reticulum protein translocation and insertion.Sec co-translocation system.Signal Recognition Particle (SRP) complex.component *(SRP54) | HID58_028607 | A0ABQ8CAQ9 | Signalrecognitionparticle54kDaproteinUncharacterizedprotein(Fragment)OS=BrassicanapusOX=3708GN=HID58_028607PE=4SV=1 |
| Protein physical control.endoplasmic reticulum protein translocation and insertion.Sec61 translocation complex.subunit gamma | HID58_078150 | A0ABQ7YSD4 | Polysaccharide biosynthesis domain-containing protein OS=Brassica napus OX=3708 GN=HID58_078150 PE=4 SV=1 |
| Protein physical control.nucleocytoplasmic transport.cargo adaptor activities.cargo adaptor protein *(IMP-alpha) | HID58_029340 | A0ABQ8CCW0 | ImportinsubunitalphaproteinOS=BrassicanapusOX=3708GN=HID58_029340PE=4SV=1 |
| Protein physical control.nucleocytoplasmic transport.nuclear pore complex (NPC).nuclear basket.nucleoporin *(NUA) | HID58_071822 | A0ABQ7Z2P0 | NucleoproteinTPR/MLP1-2domain-containingproteinOS=BrassicanapusOX=3708GN=HID58_071822PE=4SV=1 |
| Protein physical control.nucleocytoplasmic transport.nuclear pore complex (NPC).outer ring.scaffold nucleoporin *(ALADIN) | HID58_082405 | A0ABQ7YAI0 | Anaphase-promotingcomplexsubunit4WD40domain-containingproteinOS=BrassicanapusOX=3708GN=HID58_082405PE=4SV=1 |
| Protein physical control.nucleocytoplasmic transport.nuclear pore complex (NPC).outer ring.scaffold nucleoporin *(SEH1) | HID58_032844 | A0ABQ8BXJ8 | Protein SEH1 OS=Brassica napus OX=3708 GN=HID58_032844 PE=4 SV=1 |
| Protein physical control.nucleocytoplasmic transport.nuclear transport receptor activities.import karyopherin *(IMB1) | BnaA05g29380D | A0A078I6C5 | Importinsubunitbeta-1,(rape)hypotheticalproteinOS=BrassicanapusOX=3708GN=BnaA05g29380DPE=3SV=1 |
| Protein physical control.nucleocytoplasmic transport.nuclear transport receptor activities.import karyopherin *(IMB3) | DARMORV10_C03P11210.1 | A0A816HX21 | Importin-5,(rape)hypotheticalproteinOS=BrassicanapusOX=3708GN=DARMORV10_C03P11210.1PE=4SV=1 |
| Protein physical control.nucleocytoplasmic transport.nuclear transport receptor activities.transport karyopherin *(MOS14) | HID58_055113 | A0ABQ8AJI9 | ImportinN-terminaldomain-containingprotein(Fragment)OS=BrassicanapusOX=3708GN=HID58_055113PE=4SV=1 |
| Protein physical control.nucleocytoplasmic transport.nuclear transport receptor activities.transport karyopherin *(TNPO3) | HID58_036604 | A0ABQ8C9P0 | Exportin-1/Importin-beta-like domain-containing protein OS=Brassica napus OX=3708 GN=HID58_036604 PE=4 SV=1 |
| Protein physical control.nucleocytoplasmic transport.RAN GTPase cycle.RanGDP-specific nuclear import factor *(NTF2) | HID58_056002 | A0ABQ8AMX9 | NTF2-related export protein (Fragment) OS=Brassica napus OX=3708 GN=HID58_056002 PE=4 SV=1 |
| Protein physical control.plastidial protein translocation and insertion.inner envelope TIC translocation system.AAA-ATPase motor complex.FtsH12-FtsHi subcomplex.component *(FtsHi1) | HID58_041613 | A0ABQ8BCX8 | SUNdomain-containingproteinOS=BrassicanapusOX=3708GN=HID58_041613PE=4SV=1 |
| Protein physical control.plastidial protein translocation and insertion.inner envelope TIC translocation system.TIC-20 complex.component *(Tic56) | BnaA03g00280D | A0A078FDW8 | ProteinTIC56,chloroplastic,(rape)hypotheticalproteinOS=BrassicanapusOX=3708GN=BnaA03g00280DPE=4SV=1 |
| Protein physical control.plastidial protein translocation and insertion.inner envelope TIC translocation system.translocation channel *(Tic110) | BnaC05g04790D | A0A078FA32 | ProteinTIC110,chloroplastic,BnaC05g04790DproteinOS=BrassicanapusOX=3708GN=BnaC05g04790DPE=4SV=1 |
| Protein physical control.plastidial protein translocation and insertion.outer envelope TOC translocation system.receptor GTPase *(Toc33/Toc34) | BnaA03g55090D | A0A078IM29 | TranslocaseofchloroplastOS=BrassicanapusOX=3708GN=BnaA03g55090DPE=3SV=1 |
| Protein physical control.protein chaperone activities | HID58_008232 | A0ABQ8DP32 | SHSP domain-containing protein OS=Brassica napus OX=3708 GN=HID58_008232 PE=4 SV=1 |
| Protein physical control.protein chaperone activities | DARMORV10_A10P05120.1 | A0A817B1J5 | SHSPdomain-containingprotein(rape)hypotheticalproteinOS=BrassicanapusOX=3708GN=DARMORV10_A10P05120.1PE=3SV=1 |
| Protein physical control.protein chaperone activities.cytosolic Hsp70 chaperone system.Hsp70 chaperone activities.molecular chaperone *(Hsp110) | HID58_021306 | A0ABQ8CW79 | Heatshock70kDaprotein16OS=BrassicanapusOX=3708GN=HID58_021306PE=4SV=1 |
| Protein physical control.protein chaperone activities.ER Quality Control (ERQC) machinery.calnexin/calreticulin chaperone system.CNX-CRT cycle.POD1-SUN-CRT3 ER luminal chaperone complex.regulatory component *(SUN3/4/5) | HID58_041613 | A0ABQ8BCX8 | SUNdomain-containingproteinOS=BrassicanapusOX=3708GN=HID58_041613PE=4SV=1 |
| Protein physical control.protein chaperone activities.Hsp90 chaperone system.Hsp90-SGT1-RAR1 chaperone complex.molecular co-chaperone *(SGT1) | HID58_086928 | A0ABQ7XRY7 | UncharacterizedproteinOS=BrassicanapusOX=3708GN=HID58_086928PE=4SV=1 |
| Protein physical control.protein chaperone activities.plastidial chaperonin system.Cpn60 chaperone complex.component-A *(CPN60A) | DARMORV10_C04P23540.1 | A0A816JUZ4 | RuBisCOlargesubunit-bindingproteinsubunitalpha,chloroplastic,(rape)hypotheticalproteinOS=BrassicanapusOX=3708GN=DARMORV10_C04P23540.1PE=3SV=1 |
| Protein physical control.protein chaperone activities.plastidial chaperonin system.molecular co-chaperone *(Cpn20) | HID58_008941 | A0ABQ8DR46 | Trichome birefringence-like N-terminal domain-containing protein OS=Brassica napus OX=3708 GN=HID58_008941 PE=4 SV=1 |
| Protein physical control.protein chaperone activities.plastidial Hsp70 chaperone system.ATPase-stimulating co-chaperone *(DjA4/5/6/7) | HID58_062018 | A0ABQ8A0U7 | J domain-containing protein (Fragment) OS=Brassica napus OX=3708 GN=HID58_062018 PE=4 SV=1 |
| Protein physical control.protein chaperone activities.ribosome-associated chaperone activities.NAC chaperone heterodimer.subunit alpha *(NACa1) | DARMORV10_A03P37610.1 | A0A816WJK6 | Nascentpolypeptide-associatedcomplexsubunitalpha-likeprotein1,(rape)hypotheticalproteinOS=BrassicanapusOX=3708GN=DARMORV10_A03P37610.1PE=4SV=1 |
| Protein physical control.protein chaperone activities.ribosome-associated chaperone activities.NAC chaperone heterodimer.subunit alpha *(NACa1) | DARMORV10_A10P24500.1 | A0A817BLH5 | Nascentpolypeptide-associatedcomplexsubunitalpha-likeprotein3,(rape)hypotheticalprotein(Fragment)OS=BrassicanapusOX=3708GN=DARMORV10_A10P24500.1PE=4SV=1 |
| Protein physical control.protein chaperone activities.ribosome-associated chaperone activities.NAC chaperone heterodimer.subunit beta *(NACb1) | BnaAnng20100D | A0A078JHB7 | Nascentpolypeptide-associatedcomplexsubunitbetaOS=BrassicanapusOX=3708GN=BnaAnng20100DPE=3SV=1 |
| Protein physical control.protein folding.Cyclophilin-type chaperone activities.peptidyl-prolyl cis-trans isomerase *(CYP37/CYP38) | HID58_068122 | A0ABQ7ZKN4 | PPIase cyclophilin-type domain-containing protein OS=Brassica napus OX=3708 GN=HID58_068122 PE=4 SV=1 |
| Protein physical control.protein folding.nascent polypeptide chaperone *(TIG) | HID58_046262 | A0ABQ8AVZ6 | peptidylprolylisomeraseproteinOS=BrassicanapusOX=3708GN=HID58_046262PE=4SV=1 |
| Protein physical control.protein folding.nascent polypeptide chaperone *(TIG) | HID58_054087 | A0ABQ8AGU5 | PeptidylprolylisomeraseproteinOS=BrassicanapusOX=3708GN=HID58_054087PE=4SV=1 |
| Redox homeostasis.ascorbate-based redox regulation.ascorbate metabolism.L-galactose pathway.L-galactose-1-phosphate phosphatase *(VTC4) | DARMORV10_A05P44240.1 | A0A816U1P6 | Inositol-1-monophosphataseOS=BrassicanapusOX=3708GN=DARMORV10_A05P44240.1PE=3SV=1 |
| Redox homeostasis.ascorbate-based redox regulation.ascorbate metabolism.myo-inositol pathway.L-gulono-1,4-lactone oxidase *(GULLO1/2/++) | HID58_038437 | A0ABQ8BQS8 | L-gulonolactoneoxidaseproteinOS=BrassicanapusOX=3708GN=HID58_038437PE=4SV=1 |
| Redox homeostasis.ascorbate-based redox regulation.cytosolic ascorbate peroxidase *(APX) | APX | P93657 | L-ascorbateperoxidaseOS=BrassicanapusOX=3708GN=APXPE=2SV=1 |
| Redox homeostasis.ascorbate-based redox regulation.glutathione reductase *(GR) | DARMORV10_C08P29810.1 | A0A816UQ20 | GlutathionereductaseOS=BrassicanapusOX=3708GN=DARMORV10_C08P29810.1PE=3SV=1 |
| Redox homeostasis.ascorbate-based redox regulation.monodehydroascorbate reductase *(MDAR) | HID58_035277 | A0ABQ8C4F8 | Monodehydroascorbate reductase (NADH) protein OS=Brassica napus OX=3708 GN=HID58_035277 PE=4 SV=1 |
| Redox homeostasis.ascorbate-based redox regulation.monodehydroascorbate reductase *(MDAR) | BnaA03g00990D | A0A078FGS0 | Monodehydroascorbatereductase(NADH)OS=BrassicanapusOX=3708GN=BnaA03g00990DPE=3SV=1 |
| Redox homeostasis.glutathione-based redox regulation | HID58_093120 | A0ABQ7XC24 | Putative gamma-glutamylcyclotransferase protein OS=Brassica napus OX=3708 GN=HID58_093120 PE=4 SV=1 |
| Redox homeostasis.glutathione-based redox regulation | HID58_078514 | A0ABQ7YU82 | Putativegamma-glutamylcyclotransferaseproteinOS=BrassicanapusOX=3708GN=HID58_078514PE=4SV=1 |
| Redox homeostasis.glutathione-based redox regulation.glutathione S-transferase activities | HID58_024544 | A0ABQ7XIY7 | GST C-terminal domain-containing protein OS=Brassica napus OX=3708 GN=HID58_024544 PE=4 SV=1 |
| Redox homeostasis.glutathione-based redox regulation.glutathione S-transferase activities | HID58_006723 | A0ABQ8ED27 | GSTC-terminaldomain-containingprotein(Fragment)OS=BrassicanapusOX=3708GN=HID58_006723PE=4SV=1 |
| Redox homeostasis.glutathione-based redox regulation.glutathione S-transferase activities.class phi glutathione S-transferase *(GSTF1/2/++) | HID58_048015 | A0ABQ8B1T5 | Glutathione transferase protein OS=Brassica napus OX=3708 GN=HID58_048015 PE=4 SV=1 |
| Redox homeostasis.glutathione-based redox regulation.glutathione S-transferase activities.class phi glutathione S-transferase *(GSTF1/2/++) | DARMORV10_C02P08840.1 | A0A816K281 | Glutathionetransferase(Fragment)OS=BrassicanapusOX=3708GN=DARMORV10_C02P08840.1PE=3SV=1 |
| Redox homeostasis.glutathione-based redox regulation.glutathione S-transferase activities.class phi glutathione S-transferase *(GSTF1/2/++) | BnaAnng37730D | A0A078JVB3 | GlutathionetransferaseOS=BrassicanapusOX=3708GN=BnaAnng37730DPE=3SV=1 |
| Redox homeostasis.reactive electrophilic lipid homeostasis.oxylipin generation.alpha dioxygenase *(DOX1/2) | HID58_020315 | A0ABQ8DFA4 | GPI ethanolamine phosphate transferase 1 protein OS=Brassica napus OX=3708 GN=HID58_020315 PE=4 SV=1 |
| Redox homeostasis.reactive oxygen scavenging.catalase | HID58_051208 | A0ABQ8A8C9 | Peroxidase protein (Fragment) OS=Brassica napus OX=3708 GN=HID58_051208 PE=4 SV=1 |
| Redox homeostasis.reactive oxygen scavenging.catalase | BnaC08g44140D | A0A078FAL2 | PeroxidaseOS=BrassicanapusOX=3708GN=BnaC08g44140DPE=3SV=1 |
| Redox homeostasis.reactive oxygen scavenging.catalase | DARMORV10_C08P24120.1 | Q4PJU0 | PeroxidaseOS=BrassicanapusOX=3708GN=DARMORV10_C08P24120.1PE=2SV=1 |
| Redox homeostasis.reactive oxygen scavenging.catalase | HID58_041374 | A0ABQ8BAP4 | PeroxidaseproteinOS=BrassicanapusOX=3708GN=HID58_041374PE=4SV=1 |
| Redox homeostasis.reactive oxygen scavenging.catalase *(CAT1/2/3) | DARMORV10_C01P03770.1 | A0A816R4K3 | CatalaseOS=BrassicanapusOX=3708GN=DARMORV10_C01P03770.1PE=3SV=1 |
| Redox homeostasis.reactive oxygen scavenging.catalase *(CAT1/2/3) | HID58_025933 | A0ABQ8CMJ5 | CatalaseOS=BrassicanapusOX=3708GN=HID58_025933PE=4SV=1 |
| Redox homeostasis.reactive oxygen scavenging.catalase *(CAT1/2/3) | HID58_000313 | A0ABQ8EGF2 | CatalaseproteinOS=BrassicanapusOX=3708GN=HID58_000313PE=4SV=1 |
| Redox homeostasis.reactive oxygen scavenging.catalase *(CAT1/2/3) | HID58_040264 | A0ABQ8B7K2 | CatalaseproteinOS=BrassicanapusOX=3708GN=HID58_040264PE=4SV=1 |
| Redox homeostasis.reactive oxygen scavenging.superoxide dismutase activities.copper/zinc superoxide dismutase *(CSD1/2/3) | DARMORV10_C09P58880.1 | A0A816JH76 | SuperoxidedismutaseOS=BrassicanapusOX=3708GN=DARMORV10_C09P58880.1PE=3SV=1 |
| Redox homeostasis.reactive oxygen scavenging.superoxide dismutase activities.manganese superoxide dismutase *(MSD1/2) | BnaA01g31350D | A0A078HHJ9 | SuperoxidedismutaseOS=BrassicanapusOX=3708GN=BnaA01g31350DPE=3SV=1 |
| Redox homeostasis.reactive oxygen-induced signalling | HID58_077824 | A0ABQ7YRR1 | RRM domain-containing protein (Fragment) OS=Brassica napus OX=3708 GN=HID58_077824 PE=4 SV=1 |
| Redox homeostasis.reactive oxygen-induced signalling.hydrogen peroxide receptor kinase *(HPCAL1/2/++) | HID58_094631 | A0ABQ7X6P1 | Protein kinase domain-containing protein OS=Brassica napus OX=3708 GN=HID58_094631 PE=4 SV=1 |
| Redox homeostasis.reactive oxygen-induced signalling.singlet oxygen-induced signalling.protein lysine N-methyltransferase *(SAFE1) | HID58_089144 | A0ABQ7XY62 | COP9signalosomecomplexsubunit3(Fragment)OS=BrassicanapusOX=3708GN=HID58_089144PE=4SV=1 |
| Redox homeostasis.thiol-based redox regulation.methionine sulfoxide reductase activities.methionine R-enantiomer sulfoxide reductase *(MsrB) | BnaA06g00780D | A0A078GC86 | Peptide-methionine(R)-S-oxidereductaseOS=BrassicanapusOX=3708GN=BnaA06g00780DPE=3SV=1 |
| Redox homeostasis.thiol-based redox regulation.peroxiredoxin activities.type-2 peroxiredoxin *(PrxII) | DARMORV10_A04P06550.1 | A0A817APY9 | Glutaredoxin-dependentperoxiredoxinOS=BrassicanapusOX=3708GN=DARMORV10_A04P06550.1PE=3SV=1 |
| Redox homeostasis.thiol-based redox regulation.peroxiredoxin activities.type-2 peroxiredoxin *(PrxII) | HID58_005456 | A0ABQ8E8P8 | Glutaredoxin-dependentperoxiredoxinproteinOS=BrassicanapusOX=3708GN=HID58_005456PE=4SV=1 |
| Redox homeostasis.thiol-based redox regulation.peroxiredoxin activities.typical 2-Cys peroxiredoxin *(PrxA/B) | BnaC03g73430D | A0A078J0Q2 | Thioredoxin-dependentperoxiredoxinOS=BrassicanapusOX=3708GN=BnaC03g73430DPE=3SV=1 |
| Redox homeostasis.thiol-based redox regulation.regulatory protein *(LEJ1/2) | HID58_078794 | A0ABQ7YV06 | CBSdomain-containingproteinOS=BrassicanapusOX=3708GN=HID58_078794PE=4SV=1 |
| Redox homeostasis.thiol-based redox regulation.thioredoxin activities.nucleoredoxin *(NRX) | HID58_094155 | A0ABQ7X8F3 | Protein-disulfide reductase protein (Fragment) OS=Brassica napus OX=3708 GN=HID58_094155 PE=4 SV=1 |
| Redox homeostasis.tocopherol biosynthesis.regulatory protein kinase *(ABC1K3) | HID58_094631 | A0ABQ7X6P1 | Protein kinase domain-containing protein OS=Brassica napus OX=3708 GN=HID58_094631 PE=4 SV=1 |
| RNA biosynthesis.DNA-binding transcriptional regulation.beta-barrel DNA-binding domain.REM family.subgroup-E transcription factor | DARMORV10_A07P44620.1 | A0A816ZIL8 | (rape)hypotheticalproteinOS=BrassicanapusOX=3708GN=DARMORV10_A07P44620.1PE=3SV=1 |
| RNA biosynthesis.DNA-binding transcriptional regulation.beta-sheet DNA-binding domain.AHL family.clade-A transcription factor | DARMORV10_C03P33150.1 | A0A816IE71 | AT-hookmotifnuclear-localizedproteinOS=BrassicanapusOX=3708GN=DARMORV10_C03P33150.1PE=4SV=1 |
| RNA biosynthesis.DNA-binding transcriptional regulation.helix-turn-helix DNA-binding domain.MYB transcription factor family.R1R2R3-MYB transcription factor | HID58_039346 | A0ABQ8BRS4 | UncharacterizedproteinOS=BrassicanapusOX=3708GN=HID58_039346PE=4SV=1 |
| RNA biosynthesis.DNA-binding transcriptional regulation.helix-turn-helix DNA-binding domain.MYB transcription factor family.R2R3-MYB transcription factor family.subgroup-16 transcription factor | HID58_012677 | A0ABQ8E4D6 | Uncharacterizedprotein(Fragment)OS=BrassicanapusOX=3708GN=HID58_012677PE=4SV=1 |
| RNA biosynthesis.DNA-binding transcriptional regulation.helix-turn-helix DNA-binding domain.MYB transcription factor family.R2R3-MYB transcription factor family.subgroup-18 transcription factor | DARMORV10_A03P26810.1 | A0A816WDV3 | COP9signalosomecomplexsubunit2OS=BrassicanapusOX=3708GN=DARMORV10_A03P26810.1PE=3SV=1 |
| RNA biosynthesis.DNA-binding transcriptional regulation.helix-turn-helix DNA-binding domain.TRIHELIX family.transcription factor *(GT2) | DARMORV10_A07P42480.1 | A0A816ZFK7 | TrihelixtranscriptionfactorGT-2,(rape)hypotheticalproteinOS=BrassicanapusOX=3708GN=DARMORV10_A07P42480.1PE=4SV=1 |
| RNA biosynthesis.DNA-binding transcriptional regulation.helix-turn-helix DNA-binding domain.tryptophan-cluster structure.MYBR-R-type transcription factor | HID58_027798 | A0ABQ8CU80 | Smrdomain-containingproteinOS=BrassicanapusOX=3708GN=HID58_027798PE=4SV=1 |
| RNA biosynthesis.DNA-binding transcriptional regulation.undefined DNA-binding domain.ALOG transcription factor | HID58_004898 | A0ABQ8E738 | ALOG domain-containing protein OS=Brassica napus OX=3708 GN=HID58_004898 PE=4 SV=1 |
| RNA biosynthesis.DNA-binding transcriptional regulation.undefined DNA-binding domain.GRAS transcription factor | DARMORV10_A06P03020.1 | A0A816SAR4 | Scarecrow-likeprotein5,(rape)hypotheticalproteinOS=BrassicanapusOX=3708GN=DARMORV10_A06P03020.1PE=3SV=1 |
| RNA biosynthesis.DNA-binding transcriptional regulation.undefined DNA-binding domain.NIN family.RKD transcription factor | DARMORV10_C09P11330.1 | A0A816J0G2 | Zeaxanthinepoxidase,chloroplasticOS=BrassicanapusOX=3708GN=DARMORV10_C09P11330.1PE=4SV=1 |
| RNA biosynthesis.DNA-binding transcriptional regulation.zinc-coordinating DNA-binding domain.AS2/LOB transcription factor | HID58_023890 | A0ABQ8D4G0 | LOB domain-containing protein OS=Brassica napus OX=3708 GN=HID58_023890 PE=4 SV=1 |
| RNA biosynthesis.organellar gene expression machinery.plastid-encoded RNA polymerase (PEP) complex.core components.core component *(RpoA) | rpoA | A0A1B1XZH7 | DNA-directedRNApolymerasesubunitalphaOS=Brassicanapusvar.napusOX=138011GN=rpoAPE=3SV=1 |
| RNA biosynthesis.organellar gene expression machinery.plastid-encoded RNA polymerase (PEP) complex.core components.core component *(RpoB) | rpoB | A0A076VK12 | DNA-directedRNApolymerasesubunitbetaOS=BrassicanapusOX=3708GN=rpoBPE=3SV=1 |
| RNA biosynthesis.organellar gene expression machinery.plastid-encoded RNA polymerase (PEP) complex.essential co-factor components.component *(PAP1/TAC3) | BnaA01g33620D | A0A078G6R5 | SAPdomain-containingprotein,BnaA01g33620DproteinOS=BrassicanapusOX=3708GN=BnaA01g33620DPE=4SV=1 |
| RNA biosynthesis.organellar gene expression machinery.plastid-encoded RNA polymerase (PEP) complex.essential co-factor components.component *(PAP2/TAC2) | HID58_027798 | A0ABQ8CU80 | Smrdomain-containingproteinOS=BrassicanapusOX=3708GN=HID58_027798PE=4SV=1 |
| RNA biosynthesis.organellar gene expression machinery.plastid-encoded RNA polymerase (PEP) complex.essential co-factor components.lysine N-methyltransferase component *(PAP7/TAC14) | HID58_029548 | A0ABQ8CDE1 | SETdomain-containingproteinOS=BrassicanapusOX=3708GN=HID58_029548PE=4SV=1 |
| RNA biosynthesis.organellar gene expression machinery.plastid-encoded RNA polymerase (PEP) complex.regulatory co-factor components.component *(TAC18) | HID58_017857 | A0ABQ8D8D2 | TFIIS N-terminal domain-containing protein OS=Brassica napus OX=3708 GN=HID58_017857 PE=4 SV=1 |
| RNA biosynthesis.RNA polymerase II-dependent transcription.RNA polymerase-II regulation.nuclear import.nuclear import factor *(GPN1/QQT2) | HID58_041350 | A0ABQ8BAK3 | THH1/TOM1/TOM3 domain-containing protein (Fragment) OS=Brassica napus OX=3708 GN=HID58_041350 PE=4 SV=1 |
| RNA biosynthesis.RNA polymerase II-dependent transcription.transcription co-activation.MEDIATOR complex.middle module.component *(MED9) | HID58_018161 | A0ABQ8D944 | SUI1 domain-containing protein (Fragment) OS=Brassica napus OX=3708 GN=HID58_018161 PE=4 SV=1 |
| RNA biosynthesis.RNA polymerase II-dependent transcription.transcription elongation.PAF1C transcription initiation and elongation complex.component *(VIP6/CTR9) | HID58_011818 | A0ABQ8E205 | UDP-N-acetylglucosamine--peptide N-acetylglucosaminyltransferase SPINDLY protein OS=Brassica napus OX=3708 GN=HID58_011818 PE=4 SV=1 |
| RNA biosynthesis.RNA polymerase II-dependent transcription.transcription initiation.TATA box-binding protein (TBP) regulation.NC2 regulator heterodimer.component alpha | DARMORV10_C03P45620.1 | A0A816IDT1 | Dr1-associatedcorepressor,(rape)hypotheticalprotein(Fragment)OS=BrassicanapusOX=3708GN=DARMORV10_C03P45620.1PE=4SV=1 |
| RNA biosynthesis.transcriptional co-regulation.LEUNIG-SEUSS transcriptional repressor complex.adapter component *(SEU/SLK) | DARMORV10_C07P54390.1 | A0A816N0S2 | DNAmismatchrepairproteinMSH3OS=BrassicanapusOX=3708GN=DARMORV10_C07P54390.1PE=3SV=1 |
| RNA biosynthesis.transcriptional co-regulation.transcriptional co-regulator *(MBF1) | DARMORV10_C08P34010.1 | A0A816ULY3 | HTHcro/C1-typedomain-containingprotein,(rape)hypotheticalproteinOS=BrassicanapusOX=3708GN=DARMORV10_C08P34010.1PE=3SV=1 |
| RNA homeostasis.mRNA degradation.Exosome RNA surveillance complex.EXO9 core complex.component *(RRP42) | HID58_044594 | A0ABQ8BLL9 | Ribosomal RNA-processing protein 42 OS=Brassica napus OX=3708 GN=HID58_044594 PE=4 SV=1 |
| RNA homeostasis.mRNA degradation.mRNA deadenylation.CCR4-NOT complex.scaffold component *(NOT1) | DARMORV10_A07P21180.1 | A0A816YPV8 | CCR4-NOTtranscriptioncomplexsubunit1,(rape)hypotheticalproteinOS=BrassicanapusOX=3708GN=DARMORV10_A07P21180.1PE=4SV=1 |
| RNA homeostasis.mRNA degradation.mRNA decapping-dependent degradation.DDX6-type mRNA helicase *(RH6/8/12) | HID58_026665 | A0ABQ8CPL1 | RNAhelicaseproteinOS=BrassicanapusOX=3708GN=HID58_026665PE=4SV=1 |
| RNA homeostasis.mRNA degradation.mRNA decapping-dependent degradation.mRNA decapping machinery.regulatory LSM1-LSM7 complex.component *(LSm6) | HID58_026626 | A0ABQ8CRN5 | Mitogen-activatedproteinkinaseOS=BrassicanapusOX=3708GN=HID58_026626PE=4SV=1 |
| RNA homeostasis.mRNA silencing.miRNA pathway.RNA-induced silencing complex (RISC) assembly and export.assembly factor *(SAD2/EMA1) | HID58_062685 | A0ABQ8A247 | ImportinN-terminaldomain-containingprotein(Fragment)OS=BrassicanapusOX=3708GN=HID58_062685PE=4SV=1 |
| RNA homeostasis.mRNA stress granule formation.assembly factor activity.granule assembly factor *(UBP1) | HID58_070181 | A0ABQ7YY04 | RRMdomain-containingproteinOS=BrassicanapusOX=3708GN=HID58_070181PE=4SV=1 |
| RNA homeostasis.mRNA stress granule formation.mRNA endoribonuclease activities.mRNA endoribonuclease *(G3BP) | DARMORV10_C09P08020.1 | A0A816IRI9 | Nucleartransportfactor2,(rape)hypotheticalproteinOS=BrassicanapusOX=3708GN=DARMORV10_C09P08020.1PE=4SV=1 |
| RNA homeostasis.mRNA stress granule formation.regulatory factor *(RBP47) | BnaA01g26250D | A0A078FVZ3 | Polyadenylate-bindingproteinRBP47B-like,(rape)hypotheticalproteinOS=BrassicanapusOX=3708GN=BnaA01g26250DPE=3SV=1 |
| RNA homeostasis.mRNA stress granule formation.regulatory protein *(AN) | HID58_063782 | A0ABQ7Z8D6 | D-isomerspecific2-hydroxyaciddehydrogenaseNAD-bindingdomain-containingproteinOS=BrassicanapusOX=3708GN=HID58_063782PE=4SV=1 |
| RNA homeostasis.mRNA surveillance pathways.No-Go Decay (NGD) pathway.Ribosome-associated Quality Control (RQC) complex.component *(RQC1) | DARMORV10_C04P01080.1 | A0A816J9K0 | Plastidlipid-associatedprotein/fibrillinconserveddomain-containingprotein,(rape)hypotheticalproteinOS=BrassicanapusOX=3708GN=DARMORV10_C04P01080.1PE=3SV=1 |
| RNA homeostasis.mRNA surveillance pathways.Nonsense-Mediated Decay (NMD) pathway.co-effector protein *(UPF2) | HID58_052186 | A0ABQ8AB60 | MIF4Gdomain-containingproteinOS=BrassicanapusOX=3708GN=HID58_052186PE=4SV=1 |
| RNA homeostasis.post-transcriptional regulator activities.C3H-ZF-type post-transcriptional regulator activity.regulatory protein *(C3H32) | HID58_051828 | A0ABQ8AA27 | Protein disulfide-isomerase OS=Brassica napus OX=3708 GN=HID58_051828 PE=4 SV=1 |
| RNA homeostasis.RNA transport.RNA nuclear export.nuclear side machinery.TREX/THO ribonucleoparticle (RNP) trafficking complex.mRNA export adaptor activities.mRNA-binding adaptor *(ALY/Tho4) | BnaA10g12880D | A0A078H436 | THOcomplexsubunit4A,BnaA10g12880DproteinOS=BrassicanapusOX=3708GN=BnaA10g12880DPE=4SV=1 |
| RNA processing.mRNA modification.Cleavage Factor I (CF-Im) complex.component *(CPFS5/CFIm25) | HID58_012677 | A0ABQ8E4D6 | Uncharacterizedprotein(Fragment)OS=BrassicanapusOX=3708GN=HID58_012677PE=4SV=1 |
| RNA processing.organelle RNA modification.C-to-U RNA editing.ORRM-type RNA editing factor activities.RNA editing factor *(ORRM3) | HID58_005945 | A0ABQ8EA23 | RRM domain-containing protein (Fragment) OS=Brassica napus OX=3708 GN=HID58_005945 PE=4 SV=1 |
| RNA processing.organelle RNA modification.C-to-U RNA editing.RNA editing factor *(MORF) | HID58_064591 | A0ABQ7ZAF7 | MORF/ORRM1/DAG-like MORF domain-containing protein OS=Brassica napus OX=3708 GN=HID58_064591 |
| RNA processing.organelle RNA modification.C-to-U RNA editing.RNA editing factor *(MORF) | HID58_090784 | A0ABQ7XCP9 | MORF/ORRM1/DAG-like MORF domain-containing protein OS=Brassica napus OX=3708 GN=HID58_090784 PE=4 SV=1 |
| RNA processing.pre-RNA group-I/-II intron splicing.helicase activities.group-II intron RNA splicing helicase *(PMH1/2) | HID58_043462 | A0ABQ8BGN1 | Ubiquinol oxidase protein OS=Brassica napus OX=3708 GN=HID58_043462 PE=4 SV=1 |
| RNA processing.RNA pseudouridylation.H/ACA snoRNP RNA pseudouridylation complex.core component *(GAR1) | BnaA01g33870D | A0A078G6U3 | H/ACAribonucleoproteincomplexsubunitOS=BrassicanapusOX=3708GN=BnaA01g33870DPE=3SV=1 |
| RNA processing.RNA pseudouridylation.H/ACA snoRNP RNA pseudouridylation complex.pseudouridine synthase component *(Nap57/CBF5) | HID58_071353 | A0ABQ7Z1C8 | Uncharacterizedprotein(Fragment)OS=BrassicanapusOX=3708GN=HID58_071353PE=4SV=1 |
| RNA processing.spliceosome-mediated pre-mRNA splicing.MAC/NTC spliceosome-associated complex.core components.component *(CDC5/MAC1) | HID58_064428 | A0ABQ7Z9Y6 | Cell division cycle 5-like protein OS=Brassica napus OX=3708 GN=HID58_064428 PE=4 SV=1 |
| RNA processing.spliceosome-mediated pre-mRNA splicing.MAC/NTC spliceosome-associated complex.core components.component *(MAC3) | BnaC04g11150D | A0A078G1Y5 | Pre-mRNA-processingfactor19OS=BrassicanapusOX=3708GN=BnaC04g11150DPE=3SV=1 |
| RNA processing.spliceosome-mediated pre-mRNA splicing.MAC/NTC spliceosome-associated complex.core components.component *(MAC7) | HID58_017368 | A0ABQ8D6X3 | Intron-bindingproteinaquariusOS=BrassicanapusOX=3708GN=HID58_017368PE=4SV=1 |
| RNA processing.spliceosome-mediated pre-mRNA splicing.MAC/NTC spliceosome-associated complex.core components.regulatory component *(MAC12) | HID58_021232 | A0ABQ8CW06 | UncharacterizedproteinOS=BrassicanapusOX=3708GN=HID58_021232PE=4SV=1 |
| RNA processing.spliceosome-mediated pre-mRNA splicing.MAC/NTC spliceosome-associated complex.core components.RNA helicase component *(MAC15/Prp2) | DARMORV10_C05P38490.1 | A0A816L3K6 | RNAhelicaseOS=BrassicanapusOX=3708GN=DARMORV10_C05P38490.1PE=3SV=1 |
| RNA processing.spliceosome-mediated pre-mRNA splicing.U2-type major spliceosome.U1/U2/U4/U5 snRNP-associated Sm accessory RNP complex.component *(Sm-F) | DARMORV10_A03P59660.1 | A0A816WDI1 | SmproteinFOS=BrassicanapusOX=3708GN=DARMORV10_A03P59660.1PE=3SV=1 |
| RNA processing.spliceosome-mediated pre-mRNA splicing.U2-type major spliceosome.U2 snRNP complex.RNA splicing factor 3B subcomplex.component *(SF3B1/SAP155) | DARMORV10_A09P08590.1 | A0A816NP02 | Splicingfactor3Bsubunit1-like,(rape)hypotheticalproteinOS=BrassicanapusOX=3708GN=DARMORV10_A09P08590.1PE=3SV=1 |
| RNA processing.spliceosome-mediated pre-mRNA splicing.U2-type major spliceosome.U4-U6-U5 tri-snRNP complex.component *(USP39) | DARMORV10_C07P51810.1 | A0A816MYX1 | USPdomain-containingprotein,(rape)hypotheticalproteinOS=BrassicanapusOX=3708GN=DARMORV10_C07P51810.1PE=3SV=1 |
| RNA processing.spliceosome-mediated pre-mRNA splicing.U2-type major spliceosome.U6 snRNP-associated LSM2-LSM8 chaperone complex.component *(LSm6) | HID58_026626 | A0ABQ8CRN5 | Mitogen-activatedproteinkinaseOS=BrassicanapusOX=3708GN=HID58_026626PE=4SV=1 |
| RNA processing.tRNA modification.RNase-P-dependent modification.protein-only ribonuclease *(RNase P) | BnaA05g10710D | A0A078IGJ3 | RibonucleasePOS=BrassicanapusOX=3708GN=BnaA05g10710DPE=3SV=1 |
| RNA processing.tRNA modification.tRNA editing.uridylation.tRNA dihydrouridine synthase activities.tRNA dihydrouridine synthase | BnaC01g00040D | A0A078HXM7 | tRNA-dihydrouridine(47)synthase[NAD(P)(+)]OS=BrassicanapusOX=3708GN=BnaC01g00040DPE=3SV=1 |
| Secondary metabolism.alkaloids biosynthesis | DARMORV10_C07P14920.1 | A0A816MFY0 | Berberinebridgeenzyme-like13,(rape)hypotheticalproteinOS=BrassicanapusOX=3708GN=DARMORV10_C07P14920.1PE=3SV=1 |
| Secondary metabolism.alkaloids biosynthesis | HID58_035590 | A0ABQ8C5C4 | Strictosidine synthase conserved region domain-containing protein (Fragment) OS=Brassica napus OX=3708 GN=HID58_035590 PE=4 SV=1 |
| Secondary metabolism.alkaloids biosynthesis | HID58_065460 | A0ABQ7ZCV3 | Strictosidine synthase conserved region domain-containing protein OS=Brassica napus OX=3708 GN=HID58_065460 PE=4 SV=1 |
| Secondary metabolism.phenolics biosynthesis | BnaA10g11850D | A0A078H649 | Cinnamoyl-CoAreductase1,(rape)hypotheticalproteinOS=BrassicanapusOX=3708GN=BnaA10g11850DPE=4SV=1 |
| Secondary metabolism.phenolics biosynthesis.flavonoid biosynthesis.anthocyanidins.anthocyanin glutathione S-transferase *(TT19) | DARMORV10_C02P08840.1 | A0A816K281 | Glutathionetransferase(Fragment)OS=BrassicanapusOX=3708GN=DARMORV10_C02P08840.1PE=3SV=1 |
| Secondary metabolism.phenolics biosynthesis.flavonoid biosynthesis.flavones | BnaC06g06390D | A0A078I7K6 | Chalcone-flavononeisomerasefamilyproteinOS=BrassicanapusOX=3708GN=BnaC06g06390DPE=3SV=1 |
| Secondary metabolism.phenolics biosynthesis.flavonoid biosynthesis.flavones.type-I flavone synthase | DARMORV10_C02P60220.1 | A0A816KXJ7 | Fe2OGdioxygenasedomain-containingprotein(rape)hypotheticalproteinOS=BrassicanapusOX=3708GN=DARMORV10_C02P60220.1PE=3SV=1 |
| Secondary metabolism.terpenoid biosynthesis.carotenoid biosynthesis.xanthophylls.zeaxanthin epoxidase *(ZEP) | DARMORV10_C09P11330.1 | A0A816J0G2 | Zeaxanthinepoxidase,chloroplasticOS=BrassicanapusOX=3708GN=DARMORV10_C09P11330.1PE=4SV=1 |
| Secondary metabolism.terpenoid biosynthesis.cycloartenol biosynthesis.cycloartenol synthase | HID58_054460 | A0ABQ8AHJ6 | Terpenecyclase/mutasefamilymemberproteinOS=BrassicanapusOX=3708GN=HID58_054460PE=4SV=1 |
| Secondary metabolism.terpenoid biosynthesis.methylerythritol phosphate (MEP) pathway.1-deoxy-d-xylulose 5-phosphate import.D-xylulose kinase | HID58_022382 | A0ABQ8CZ51 | Xylulose kinase protein OS=Brassica napus OX=3708 GN=HID58_022382 PE=4 SV=1 |
| Secondary metabolism.terpenoid biosynthesis.methylerythritol phosphate (MEP) pathway.2-C-methyl-D-erythritol 2,4-cyclodiphosphate synthase | HID58_032768 | A0ABQ8BX98 | 2-C-methyl-D-erythritol 2,4-cyclodiphosphate synthase protein OS=Brassica napus OX=3708 GN=HID58_032768 PE=4 SV=1 |
| Secondary metabolism.terpenoid biosynthesis.mevalonate (MVA) pathway.mevalonate diphosphate decarboxylase *(MVD1/2) | DARMORV10_C08P29490.1 | A0A816VBP6 | DiphosphomevalonatedecarboxylaseOS=BrassicanapusOX=3708GN=DARMORV10_C08P29490.1PE=3SV=1 |
| Secondary metabolism.terpenoid biosynthesis.terpene biosynthesis.triterpenoid synthase | HID58_054460 | A0ABQ8AHJ6 | Terpenecyclase/mutasefamilymemberproteinOS=BrassicanapusOX=3708GN=HID58_054460PE=4SV=1 |
| Solute transport.carrier-mediated transport.APC-type transporter superfamily.borate transporter *(BOR) | HID58_040515 | A0ABQ8B944 | SerinehydroxymethyltransferaseproteinOS=BrassicanapusOX=3708GN=HID58_040515PE=4SV=1 |
| Solute transport.carrier-mediated transport.DMT-type transporter superfamily.group-NST/TPT transporter activities.nucleotide sugar transporter *(UUAT) | HID58_019818 | A0ABQ7ZM08 | DNAgyrasesubunitBOS=BrassicanapusOX=3708GN=HID58_019818PE=4SV=1 |
| Solute transport.carrier-mediated transport.glycerate:glycolate transporter *(PLGG1) | DARMORV10_A05P25290.1 | A0A816TP41 | HistoneacetyltransferaseOS=BrassicanapusOX=3708GN=DARMORV10_A05P25290.1PE=3SV=1 |
| Solute transport.carrier-mediated transport.MATE-type transporter activities.metal-citrate complex transporter *(FRD3) | DARMORV10_C03P77090.1 | A0A816ING8 | ProteinDETOXIFICATIONOS=BrassicanapusOX=3708GN=DARMORV10_C03P77090.1PE=3SV=1 |
| Solute transport.carrier-mediated transport.MFS-type transporter superfamily.anion transporter *(NRT1/PTR) | HID58_072875 | A0ABQ7Z5U2 | Band 7 domain-containing protein OS=Brassica napus OX=3708 GN=HID58_072875 PE=4 SV=1 |
| Solute transport.carrier-mediated transport.MFS-type transporter superfamily.phosphate transporter *(PHT1) | HID58_073396 | A0ABQ7Z734 | Major facilitator superfamily (MFS) profile domain-containing protein (Fragment) OS=Brassica napus OX=3708 GN=HID58_073396 PE=4 SV=1 |
| Solute transport.carrier-mediated transport.MTCC-type transporter activities.adenine nucleotide transporter *(ANT) | DARMORV10_C09P63600.1 | A0A816JAU4 | HydroxyprolineO-arabinosyltransferase-likedomain-containingprotein,(rape)hypotheticalproteinOS=BrassicanapusOX=3708GN=DARMORV10_C09P63600.1PE=3SV=1 |
| Solute transport.carrier-mediated transport.MTCC-type transporter activities.mitochondrial dicarboxylate carrier *(DTC) | BnaA02g04450D | A0A078I266 | Mitochondrialcarrier(TC2.A.29)family,(rape)hypotheticalproteinOS=BrassicanapusOX=3708GN=BnaA02g04450DPE=3SV=1 |
| Solute transport.carrier-mediated transport.MTCC-type transporter activities.S-adenosylmethionine transporter *(SAMTL) | HID58_051955 | A0ABQ8AAG5 | Non-specific serine/threonine protein kinase OS=Brassica napus OX=3708 GN=HID58_051955 PE=4 SV=1 |
| Solute transport.channel-mediated transport.MIP-type intrinsic protein activities.tonoplast intrinsic protein *(TIP) | BnaA01g28120D | A0A078G0V8 | MIP/aquaporin(TC1.A.8)family,(rape)hypotheticalproteinOS=BrassicanapusOX=3708GN=BnaA01g28120DPE=3SV=1 |
| Solute transport.porin-mediated transport.outer membrane porin *(OEP37) | BnaC04g49380D | A0A078GAE5 | Outerenvelopeporeprotein37,chloroplastic-like,(rape)hypotheticalproteinOS=BrassicanapusOX=3708GN=BnaC04g49380DPE=4SV=1 |
| Solute transport.primary active transport.ABC-type transporter superfamily.ABC group-1 transporter activities.ABC1 subfamily-B transporter activities.ABCB-type transporter *(ABCB28) | BnaCnng49290D | A0A078JH18 | ABCtransporterBfamilymember28,BnaCnng49290DproteinOS=BrassicanapusOX=3708GN=BnaCnng49290DPE=4SV=1 |
| Solute transport.primary active transport.ABC-type transporter superfamily.ABC group-2 transporter activities.ABC2 subfamily-G transporter activities.ABC2 subfamily-G full-size transporter *(PDR1/2/3) | HID58_064933 | A0ABQ7ZBN3 | ABCtransporterdomain-containingproteinOS=BrassicanapusOX=3708GN=HID58_064933PE=4SV=1 |
| Solute transport.primary active transport.P-type ATPase superfamily.group-P2 ATPase activities.P2B-type calcium cation-transporting ATPase *(ACA) | BnaC02g11860D | A0A078H631 | Calcium-transportingATPaseOS=BrassicanapusOX=3708GN=BnaC02g11860DPE=3SV=1 |
| Solute transport.primary active transport.P-type ATPase superfamily.group-P2 ATPase activities.P2B-type calcium cation-transporting ATPase *(ACA) | DARMORV10_C03P51530.1 | A0A816IBF3 | Calcium-transportingATPaseOS=BrassicanapusOX=3708GN=DARMORV10_C03P51530.1PE=3SV=1 |
| Solute transport.primary active transport.P-type ATPase superfamily.group-P3 ATPase activities.P3A-type proton-translocating ATPase *(AHA) | DARMORV10_C03P88830.1 | A0A816IM64 | PlasmamembraneATPaseOS=BrassicanapusOX=3708GN=DARMORV10_C03P88830.1PE=3SV=1 |
| Solute transport.primary active transport.V-type ATPase complex.membrane V0 subcomplex.subunit d | HID58_049282 | A0ABQ8B4I4 | V-type proton ATPase subunit protein OS=Brassica napus OX=3708 GN=HID58_049282 PE=4 SV=1 |
| Solute transport.primary active transport.V-type ATPase complex.peripheral V1 subcomplex.subunit A | HID58_028072 | A0ABQ8CTJ7 | V-typeprotonATPasecatalyticsubunitAproteinOS=BrassicanapusOX=3708GN=HID58_028072PE=4SV=1 |
| Solute transport.primary active transport.V-type ATPase complex.peripheral V1 subcomplex.subunit C | DARMORV10_A08P32590.1 | A0A817AGF7 | V-typeprotonATPasesubunitCOS=BrassicanapusOX=3708GN=DARMORV10_A08P32590.1PE=3SV=1 |
| Solute transport.primary active transport.V-type ATPase complex.peripheral V1 subcomplex.subunit G | HID58_020315 | A0ABQ8DFA4 | GPI ethanolamine phosphate transferase 1 protein OS=Brassica napus OX=3708 GN=HID58_020315 PE=4 SV=1 |
| Uncharacterised context.enzymatic activities.EC_1 oxidoreductases.EC_1-10 oxidoreductase acting on diphenol or related substance as donor | HID58_078903 | A0ABQ7YVH3 | UncharacterizedproteinOS=BrassicanapusOX=3708GN=HID58_078903PE=4SV=1 |
| Uncharacterised context.enzymatic activities.EC_2 transferases.EC_2-3 acyltransferase | HID58_021843 | A0ABQ8CXI3 | 2-isopropylmalate synthase protein OS=Brassica napus OX=3708 GN=HID58_021843 PE=4 SV=1 |
| Uncharacterised context.enzymatic activities.EC_2 transferases.EC_2-3 acyltransferase | HID58_075879 | A0ABQ7YMN3 | Acetyltransferase protein (Fragment) OS=Brassica napus OX=3708 GN=HID58_075879 PE=4 SV=1 |
| Uncharacterised context.enzymatic activities.EC_2 transferases.EC_2-3 acyltransferase | BnaAnng11500D | A0A078ITY5 | ProteinECERIFERUM26-like,BnaAnng11500DproteinOS=BrassicanapusOX=3708GN=BnaAnng11500DPE=3SV=1 |
| Uncharacterised context.enzymatic activities.EC_2 transferases.EC_2-4 glycosyltransferase | HID58_050671 | A0ABQ8A6T3 | Glycosyltransferase(Fragment)OS=BrassicanapusOX=3708GN=HID58_050671PE=4SV=1 |
| Uncharacterised context.enzymatic activities.EC_2 transferases.EC_2-4 glycosyltransferase | HID58_010216 | A0ABQ8DUT8 | UDP-glycosyltransferases domain-containing protein (Fragment) OS=Brassica napus OX=3708 GN=HID58_010216 PE=4 SV=1 |
| Uncharacterised context.enzymatic activities.EC_2 transferases.EC_2-7 transferase transferring phosphorus-containing group | BnaA09g30470D | A0A078G4J9 | tRNAnucleotidyltransferase/poly(A)polymerasefamily,BnaA09g30470DproteinOS=BrassicanapusOX=3708GN=BnaA09g30470DPE=3SV=1 |
| Uncharacterised context.enzymatic activities.EC_3 hydrolases.EC_3-1 hydrolase acting on ester bond | DARMORV10_A04P22280.1 | A0A817B7F2 | 3-Hydroxyisobutyryl-CoAhydrolaseOS=BrassicanapusOX=3708GN=DARMORV10_A04P22280.1PE=3SV=1 |
| Uncharacterised context.enzymatic activities.EC_3 hydrolases.EC_3-4 hydrolase acting on peptide bond (peptidase) | HID58_044789 | A0ABQ8BM46 | Carboxypeptidase protein OS=Brassica napus OX=3708 GN=HID58_044789 PE=4 SV=1 |
| Uncharacterised context.enzymatic activities.EC_3 hydrolases.EC_3-5 hydrolase acting on carbon-nitrogen bond, other than peptide bond | HID58_083049 | A0ABQ7YCD2 | 3,4-Dihydroxy-2-butanone-4-phosphate synthase protein OS=Brassica napus OX=3708 GN=HID58_083049 PE=4 SV=1 |
[truncated: 21,396 more chars]
